# The special role of B(C_6_F_5_)_3_ in the single electron reduction of quinones by radicals†
†Electronic supplementary information (ESI) available: additional experimental details, further spectral and crystallographic data, additional data from the cyclic voltammetry and ESR studies. CCDC 1822878 to 1822888. For ESI and crystallographic data in CIF or other electronic format see DOI: 10.1039/c8sc03005g


**DOI:** 10.1039/c8sc03005g

**Published:** 2018-08-20

**Authors:** Xin Tao, Constantin G. Daniliuc, Robert Knitsch, Michael Ryan Hansen, Hellmut Eckert, Maximilian Lübbesmeyer, Armido Studer, Gerald Kehr, Gerhard Erker

**Affiliations:** a Organisch-Chemisches Institut , Westfälische Wilhelms-Universität Münster , Corrensstraße 40 , 48149 Münster , Germany; b Institut für Physikalische Chemie der Universität Münster , Corrensstraße 28-30 , 48149 Münster , Germany; c Instituto de Fisica, Sáo Carlos , Universidade de Sáo Paulo , CP 369, 13566-590 , Sáo Carlos , S.P. , Brazil

## Abstract

In the presence of two molar equiv. of B(C_6_F_5_)_3_*p*-benzoquinone reacts with persistent radicals TEMPO, trityl or decamethylferrocene by single electron transfer to give doubly *O*-borylated benzosemiquinone radical anions with TEMPO^+^, trityl or  ferrocenium counter cations. All three [(C_6_F_5_)_3_B]_2_–semiquinone radical anion salts were characterized by X-ray diffraction. The addition of donor reagent THF or DMSO induced rapid back electron transfer, in the case of the [(C_6_F_5_)_3_B]_2_–semiquinone radical anion oxoammonium salt giving rise to the formation of the (C_6_F_5_)_3_B–DMSO (or THF) Lewis adduct, *p*-benzoquinone and the TEMPO radical. The reaction of 9,10-anthraquinone or acenaphthenequinone with either the Gomberg dimer or  in 1 : 1 stoichiometry in the presence of two molar equiv. of B(C_6_F_5_)_3_ gave the respective two-fold O–B(C_6_F_5_)_3_ containing 9,10-anthrasemiquinone or acenaphthene-semiquinone radical anion salts with either Ph_3_C^+^ or  counter cations. These products were also characterized by X-ray diffraction. The  salts showed analogous back electron shuttling behavior upon treatment with DMSO. 9,10-Phenanthrenequinone reacted analogously with B(C_6_F_5_)_3_ and the electron rich ferrocene. The  [(C_6_F_5_)_3_B]_2_–9,10-phenanthrene–semiquinone salt was characterized by X-ray diffraction. The radical anions were characterized by ESR spectroscopy.

## Abstract 2

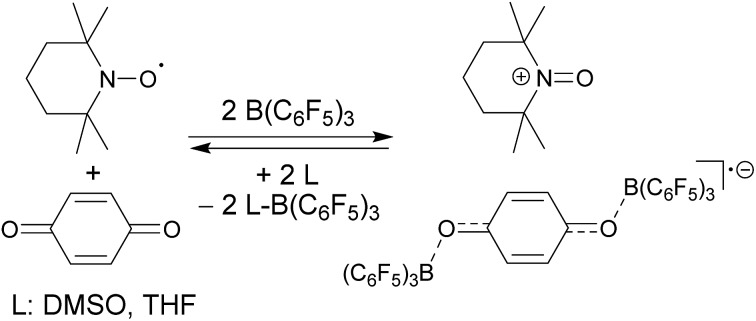
In the presence of two molar equiv. of B(C_6_F_5_)_3_*p*-benzoquinone reacts with persistent radicals TEMPO, trityl or decamethylferrocene by single electron transfer to give the doubly *O*-borylated benzosemiquinone radical anion with TEMPO^+^, trityl cation or 
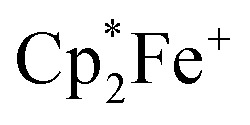
 ferrocenium counter cations.

## Introduction

Quinone/semiquinone/hydroquinone redox triads play important roles in biology.^1^ They serve as redox mediators or active electron shuttles in a variety of processes involving intra- or intercellular electron transport, including *e.g.* the respiratory chain and photosynthesis.^2^ Semiquinone radicals are important in superoxide and peroxide formation.1*e* Some examples serve as chemical redox reagents in organic synthesis.^3^ Anthraquinone/anthrahydroquinone derivatives are involved as active agents in technical H_2_O_2_ production.^4^ In many of these redox examples sequential electron transfer plays a role, proceeding through intermediate semiquinone steps. Some such semiquinones are rather stable and have substantial half-lives. Biological systems containing hydro-quinone/semiquinone/quinone triads were structurally characterized at different redox stages.^5^ Several X-ray crystal structure analyses of semiquinones were published.^6^ They usually show short C–O bonds, which are only slightly elongated as compared to their parent quinone systems (*e.g.* anthraquinone: 1.222 Å).^7^ Examples include *p*-benzosemiquinone [C=O distance: 1.295 Å], tetrabromo- and tetrachlorobenzosemiquinone [*d*(C=O): 1.25(2) Å and 1.248(2) Å, depending slightly on the countercation]. The caesium salt of 2,3-dicyano-5,6-dichlorobenzosemiquinone shows a carbon oxygen bond length of 1.243 (1) Å (at 120 K).^8^ In contrast quinhydrone shows two rather different C=O bond lengths of its pair of subunits, namely 1.234 Å and 1.385/1.409 Å, respectively.^9^

Alkali metal semiquinones are not too easy to make; often they are generated by exposure of the respective quinones to alkali metal mirrors. We regarded it desirable to search for a method that would easily and reliably allow for using kinetically favourable pathways for the preparation of suitable semiquinone derivatives, which would then show similar structural (and chemical) features to free metal containing semiquinones as they were described in the literature. We found that this could be achieved in a rather convenient way by the reaction of a number of quinones with persistent radicals or a suitable organometallic electron transfer reagent in the presence of two molar equivalents of the strong boron Lewis acid tris(pentafluorophenyl)borane B(C_6_F_5_)_3_ (**1**). Representative examples will be described in this account and some properties of the resulting borane containing semiquinones will be discussed.

**Scheme 1:**
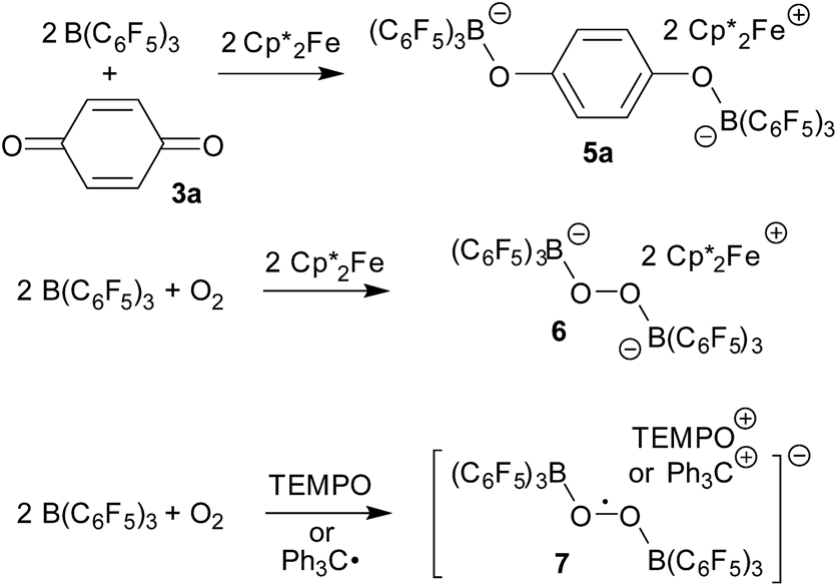


There have been a few previous reports about the use of B(C_6_F_5_)_3_ in radical and electron transfer chemistry. Norton *et al.*^10^,^11^ have shown that the [˙B(C_6_F_5_)_3_^–^] radical anion (**2**) can be formed *e.g.* upon treatment of **1** with 
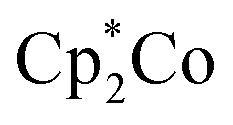
 serving as a single electron donor. The radical anion **2** was characterized by ESR spectroscopy. Stephan *et al.* recently proposed the involvement of the [˙B(C_6_F_5_)_3_^–^] radical anion in FLP chemistry.^12^ They have also shown that B(C_6_F_5_)_3_ reduces quinone derivatives in the presence of dihydrogen to give reactive paramagnetic semiquinone type boron containing radical products.^13^ It was shown in the course of that study that *p*-benzoquinone (**3a**) underwent two electron reduction by two molar equivalents of decamethylferrocene (
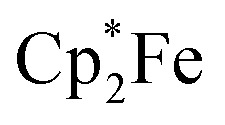
, **4**) in the presence of B(C_6_F_5_)_3_ to yield the *O*-borylated hydroquinone dianion product **5a** (with two 
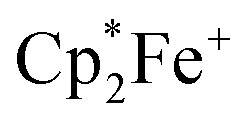
 cations).^14^ Agapie *et al.* have shown a conceptionally related two electron reduction of dioxygen with 
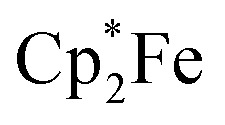
 and B(C_6_F_5_)_3_ to give borylated peroxide **6** (Scheme 1).^15^ We have recently shown that O_2_ could be reduced by suitable persistent radicals (TEMPO or the trityl radical) in the presence of B(C_6_F_5_)_3_ to yield paramagnetic superoxide products **7**.^16^ The sensitive superoxide radical anion systems **7** were characterized by X-ray diffraction, ESR spectroscopy and cyclic voltammetry.^14^ The treatment of the systems **7** with suitable donors L (THF and DMSO) resulted in back electron transfer with the formation of respective L–B(C_6_F_5_)_3_ adducts and re-formation of triplet dioxygen. With additional trityl radicals or a second equivalent of 
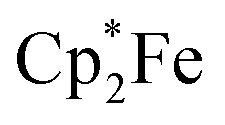
 the reduction of (superoxide)[B(C_6_F_5_)_3_]_2_^–^ to (peroxide)[B(C_6_F_5_)_3_]_2_^2–^ was achieved.^16^

## Results and discussion

**Scheme 2:**
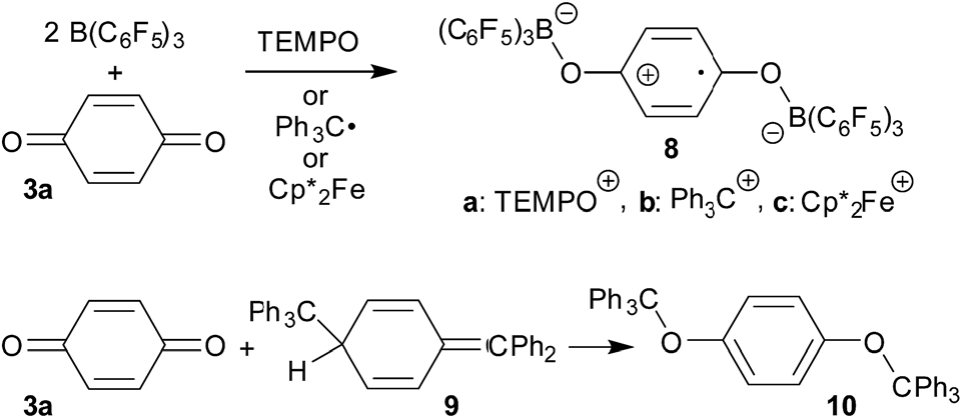


**Fig. 1:**
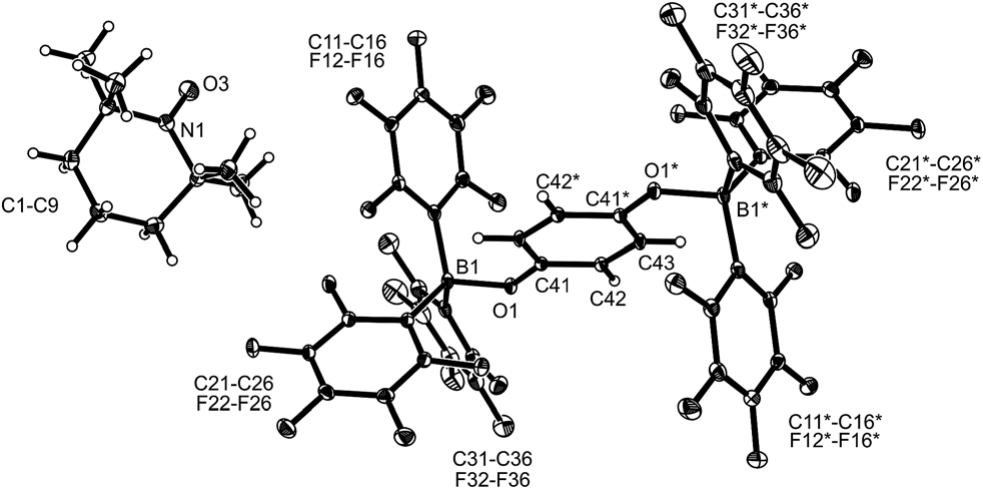
A view of the molecular structure of the doubly borylated *p*-benzosemiquinone salt **8a** (thermal ellipsoids are shown with 15% probability).

The TEMPO radical does not react with *p*-benzoquinone under our typical conditions (r.t., CD_2_Cl_2_). However, this changed in the presence of the Lewis acid B(C_6_F_5_)_3_. The mixing of *p*-benzoquinone with B(C_6_F_5_)_3_ leads to adduct formation.^14^ The inspection of the NMR spectra at low temperature (193 K) indicated the formation of a mono-adduct when we started from 1 : 1 stoichiometry. In 1 : 2 stoichiometry there seemed to be a dynamic equilibration between the two possible adducts and free *p*-benzoquinone and B(C_6_F_5_)_3_ which was so rapid that we could not “freeze” it out on the NMR time scale even at low temperature (see the ESI† for details). The addition of one molar equiv. of TEMPO to the resulting red solution (r.t., 10 min) gave a dark brown mixture. The NMR spectra indicated that a reaction with the apparent formation of a paramagnetic product had taken place. We monitored the broad ^1^H NMR signals due to the (diamagnetic) oxoammonium cation [*i.e.* TEMPO^+^] of the product **8a** (Scheme 2). The diffusion of pentane vapour into the dichloromethane solution at –35 °C (48 h) yielded crystalline compound **8a** (59% isolated). It was characterized by X-ray diffraction (Fig. 1 and Table 1).

**Table 1:** Selected structural data of the *O*-borylated p-benzosemiquinone radical ion salts **8a–c** and the *O*-borylated hydroquinone derivative **5a**^*a*^

Compound	**8a**	**8b**	**8c**	**5a** ^*b*^
Cation	TEMPO^+^	Ph_3_C^+^	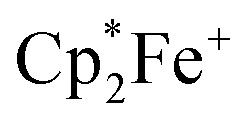	2 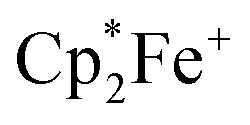
B1–O1	1.531(3)	1.523(5)	1.533(4)	1.471(5)
O1–C41	1.298(3)	1.308(4)	1.306(4)	1.367(4)
C41–C42	1.424(3)	1.427(5)	1.419(4)^*c*^	1.393(5)^*e*^
C42–C43	1.350(3)	1.353(5)	1.354(4)^*d*^	1.387(6)
B1–O1–C41	129.2(2)	127.7(3)	126.4(2)	124.8(3)
∑B1^CCC^	336.3	336.1	334.3	330.1

^*a*^Bond lengths in Å; angles in °.

^*b*^From ref. 14.

^*c*^C41–C43 1.429(5).

^*d*^C42–C43*.

^*e*^C42–C43′ 1.389(5).

The X-ray crystal structure analysis confirmed the formation of the doubly B(C_6_F_5_)_3_ bonded *p*-benzosemiquinone radical anion with one TMP oxoammonium countercation. It features a semiquinone core that has a B(C_6_F_5_)_3_ moiety attached to each of its oxygen atoms in a bent arrangement. The B1–O1 bonds are relatively long and the O1–C41 linkage of the central organic unit is only slightly elongated. The core has retained a long/short/long *p*-benzoquinone like carbon–carbon bond lengths sequence of the central *p*-phenylene moiety. The TEMPO^+^ oxoammonium counter cation shows a short N1–O3 bond length (1.191(3) Å) and a planar-trigonal coordination geometry at the nitrogen atom N1 (∑N1^OCC^ = 360°).

*p*-Benzoquinone reacts with the Gomberg dimer by two-fold addition of the trityl radical to the pair of carbonyl oxygen atoms to form the respective bis-trityl hydroquinone ether **10**.^17^ This typical reaction pathway becomes completely suppressed in the presence of B(C_6_F_5_)_3_. We reacted the red solution that was obtained by mixing *p*-benzoquinone with two molar equiv. of B(C_6_F_5_)_3_ in dichloromethane with half an equivalent of the Gomberg dimer **9** (Scheme 2). The workup of the dark red reaction mixture involving layering with pentane and crystallization at –35 °C gave the diborylated benzosemiquinone trityl salt **8b**, which we isolated in 65% yield as a dark red crystalline material. In CD_2_Cl_2_ solution we have monitored the ^1^H/^13^C NMR features of the trityl cation, but the semiquinone radical anion part of **8b** appears NMR silent. The doubly borylated semiquinone system **8b** was positively identified and structurally characterized by X-ray crystal structure analysis. This revealed bonding of a pair of (C_6_F_5_)_3_B-moieties to the oxygen atoms of the semiquinone nucleus. The structural parameters of the central core of **8b** are very similar to those of its oxoammonium congener **8a** (Table 1), but it shows a planar tricoordinate trityl cation, of course. The structure of compound **8b** is depicted in the ESI.†

The treatment of the *p*-benzoquinone/2 B(C_6_F_5_)_3_ mixture with one molar equiv. of 
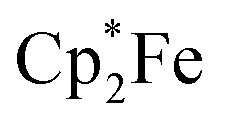
 also took place by one electron transfer and we isolated the decamethylferrocenium/semiquinone radical anion salt **8c** (Scheme 2), which also had a pair of stabilizing (C_6_F_5_)_3_B-moieties attached to the core oxygen atoms. Dark red crystals of compound **8c** were isolated in 63% yield. The compound was characterized by X-ray diffraction (the structure is depicted in the ESI,† see Table 1 for selected structural parameters). It features a broad paramagnetically shifted ^1^H NMR feature of the 
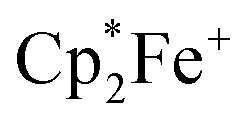
 ferrocenium cation at *δ* = –36.7. The semiquinone radical anion part of compound **8c** appears NMR silent.

Compounds **8a**, **8b** and **8c** in dichloromethane solution show a broad ESR signal at *g* = 2.0049. The best-resolved spectra are observed at low temperatures (180 K) whereas resolution is gradually lost when the measurement temperature is increased. We attribute the general lack in resolution observed here to the low solubility of the radical in organic solvents of low polarity probably leading to ion pair aggregation. Solvents offering increased polarity such as THF and DMSO either lead to the decomposition of the semiquinone derivative, due to their Lewis basicity, or do not allow ESR measurements at sufficiently low temperatures (for more information see the ESI†).

**Fig. 2:**
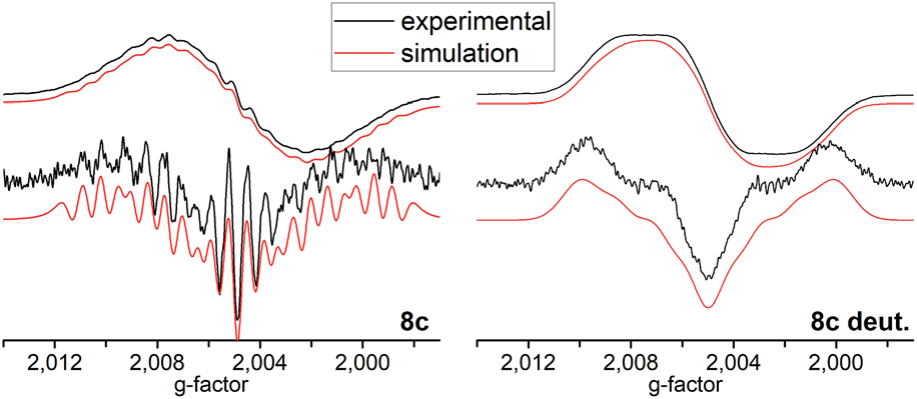
Top: Experimental (black) and simulated (red) cw-ESR spectra of **8c** and tetra-deuterated **8c** acquired at 180 K in ∼10^–4^ M DCM solution. Bottom: Second derivatives of the respective spectra.

The line shapes measured at 180 K in CH_2_Cl_2_ can be simulated in terms of magnetic hyperfine couplings to two equivalent boron nuclei and two pairs of equivalent protons (Fig. 2). The simulated spectra displayed are based on parameters which minimize deviations from the experimental data for both the first and the second derivative spectra. The inequivalence of the ring protons suggests that at 180 K conformational reorientations are slow on the ESR timescale. The ^11^B hyperfine coupling constant (6.4 MHz) was confirmed independently by measuring a fully deuterated sample of an identical radical in compound **8c** (Fig. 2). The further loss in resolution with increasing temperature suggests that the line-shape is additionally influenced by exchange phenomena due to conformational dynamics occurring on the ESR timescale.

**Scheme 3:**
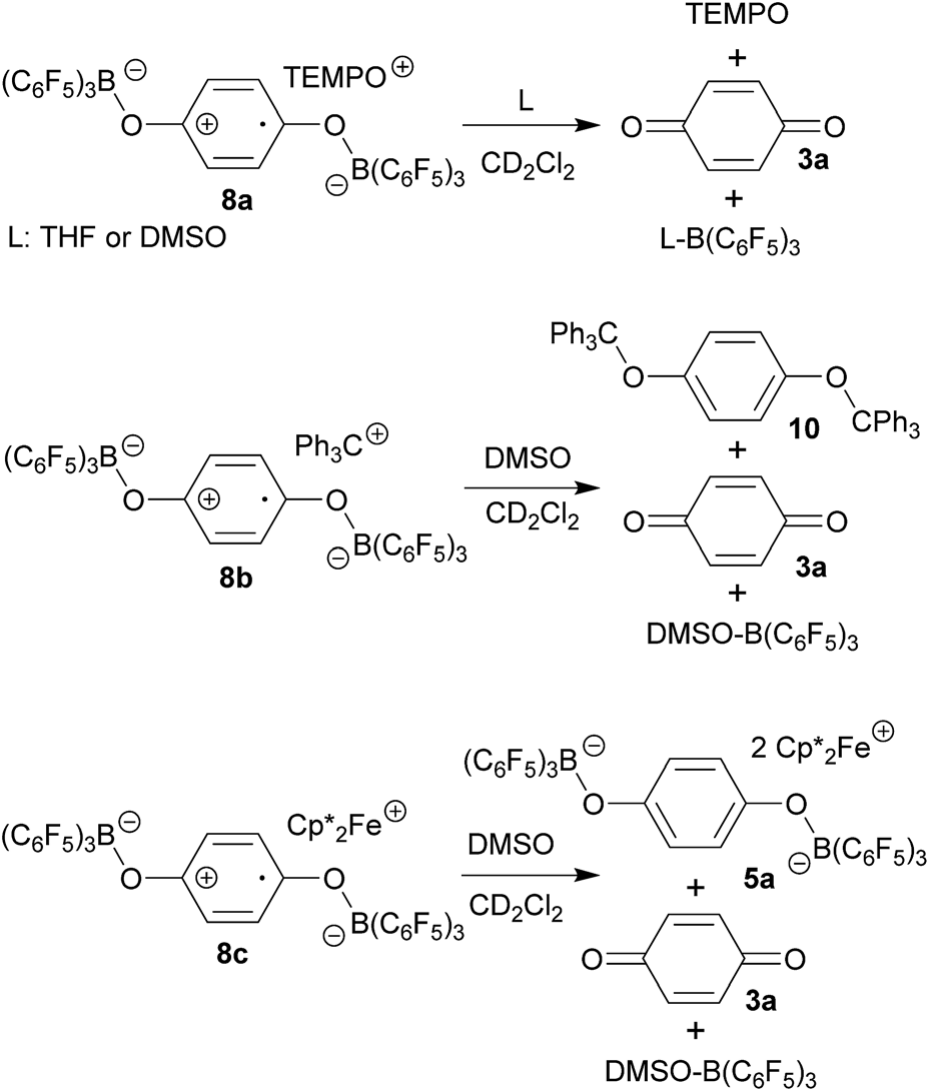


The compounds **8** are sensitive toward Lewis base donor ligands that typically add to the B(C_6_F_5_)_3_ Lewis acid forming the respective Lewis adducts.^18^ This leads to back electron transfer. In the case of the oxoammonium/benzosemiquinone[B(C_6_F_5_)_3_]_2_ salt **8a** the reaction with THF occurs readily and leads to the formation of the TEMPO radical (as shown by ESR spectroscopy), the (C_6_F_5_)_3_B–THF adduct and *p*-benzoquinone (both were identified by their NMR spectra from the reaction mixture). The treatment of **8a** with DMSO furnished a similar result (Scheme 3 and the ESI† for details).

The reaction of the trityl cation salt **8b** with DMSO takes a slightly different course. Here the exposure to the Lewis basic reagent leads to back electron transfer with the formation of the DMSO·B(C_6_F_5_)_3_ adduct. In this case the re-formation of the trityl radical apparently results in the redox reaction with re-formed *p*-benzoquinone to the known diamagnetic product **10**. We observed an approximately equal amount of **10** and *p*-benzoquinone **3a** in the product mixture (Scheme 3).

Back electron transfer also ensues apparently upon treatment of the decamethylferrocenium/benzosemiquinone–[B(C_6_F_5_)_3_]_2_ salt **8c** with DMSO. We observed the formation of the DMSO·B(C_6_F_5_)_3_ adduct, *p*-benzoquinone and the doubly borylated hydroquinone dianion. This indicates the single electron transfer of the *in situ* generated 
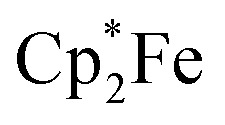
 to remaining **8c**, forming the bis-B(C_6_F_5_)_3_ adduct **5a** with the hydroquinone dianion (Scheme 3 and the ESI† for details).

**Scheme 4:**
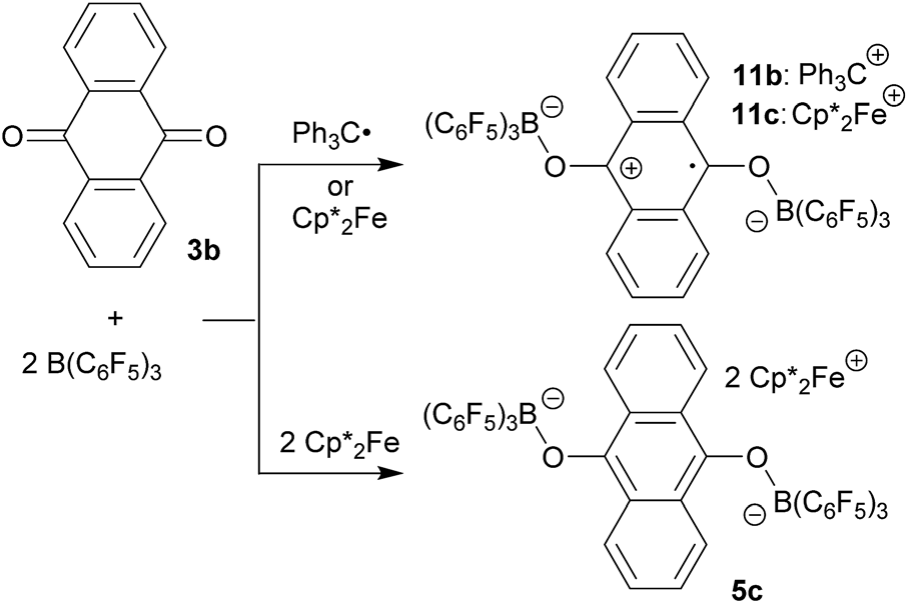


9,10-Anthraquinone (**3b**), acenaphthenequinone (**3c**) or 9,10-phenanthrenequinone (**3d**) were neither reduced with TEMPO nor with the Gomberg dimer or 
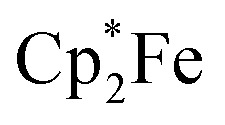
 under our typical conditions in the absence of the B(C_6_F_5_)_3_ Lewis acid. Even the anthraquinone/2 B(C_6_F_5_)_3_ mixture did not react with TEMPO. However, the trityl radical from its equilibrium with the Gomberg dimer reduced the quinone **3b** in a single electron transfer process to generate the trityl cation/anthrasemiquinone–[B(C_6_F_5_)_3_]_2_ radical anion salt **11b** (Scheme 4). We tried to isolate compound **11b** from the dark green reaction mixture by crystallization, but this product proved to be too sensitive. We only obtained a mixture of dark green compounds admixed with a colourless (apparently diamagnetic) material. The NMR spectra showed the presence of **3b**. However, we found some green single crystals that were suited for the characterization of compound **11b** by X-ray diffraction.

**Fig. 3:**
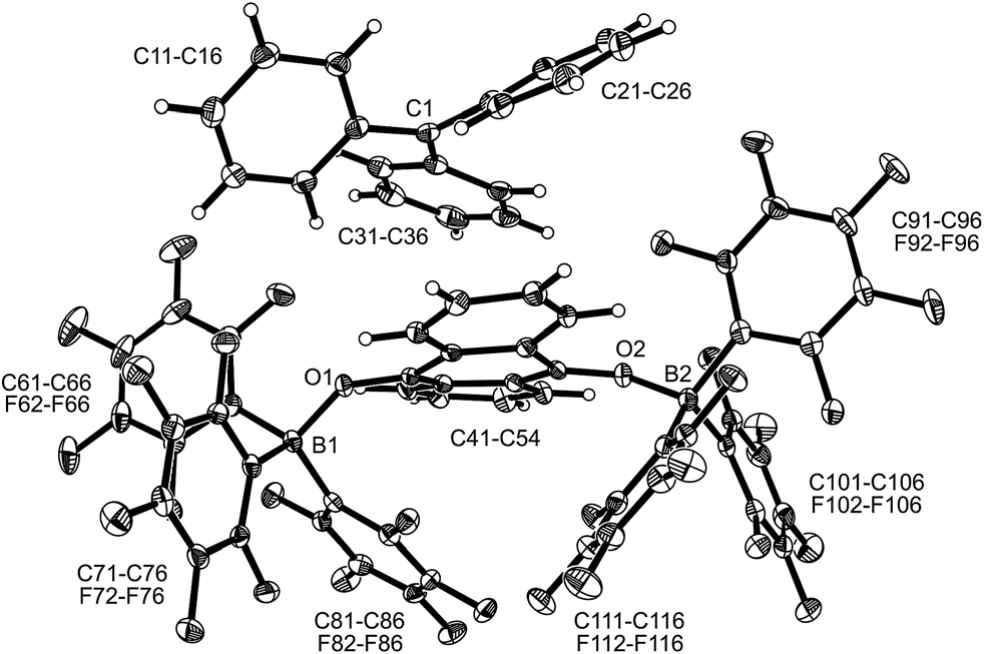
Molecular structure of compound **11b** (thermal ellipsoids are shown with 50% probability). Selected bond lengths (Å) and angles (°): B1–O1 1.524(3), B2–O2 1.524(3), O1–C41 1.301(2), O2–C48 1.303(2), C41–C42 1.432(3), C42–C47 1.421(3), C47–C48 1.441(3), C48–C49 1.425(3), C49–C54 1.423(3), C54–C41 1.445(3), B1–O1–C41 134.5(2), B2–O2–C48 136.0(2), C54–C41–O1–B1 –135.8(2), and C47–C48–O2–B2 –145.2(2).

The X-ray crystal structure analysis of compound **11b** (Fig. 3) shows an anthrasemiquinone core with B(C_6_F_5_)_3_ groups attached to the pair of oxygen atoms. Consequently, the carbon–oxygen bonds were found slightly elongated as are the carbon–carbon bonds proximal to the carbonyl carbon atom. The annulated pair of phenylene rings shows the slightly alternating π-system that is typical of quinoid aromatic structures.^19^ The system is bent at oxygen and the pair of boron–oxygen vectors is found oriented markedly outside of the central anthrasemiquinone plane. The overall structure is close to (but not crystallographically exactly) *C*_2_-symmetric.

We could also achieve the single electron reduction of 9,10-anthraquinone (**3b**) with 
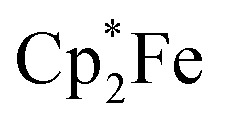
 in the presence of B(C_6_F_5_)_3_. The treatment of **3b** with one molar equiv. of the electron-rich ferrocene derivative in the presence of two molar equiv. of B(C_6_F_5_)_3_ gave a dark green reaction mixture. In this case we were able to isolate the respective product **11c** sufficiently pure. After crystallization it was isolated in 88% yield and characterized by C, H elemental analysis, by NMR spectroscopy [
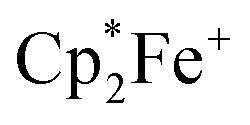
: *δ* = –36.5 (^1^H)], by ESR spectroscopy (broad unstructured signal in CD_2_Cl_2_ at *g* = 2.0043) and by X-ray diffraction. Compound **11c** shows the structural parameters of the anthrasemiquinone·[B(C_6_F_5_)_3_]_2_ radical anion core that are very similar to those of **11b** (see the ESI† about the details of the characterization of compound **11c**). Compound **11c** could be further reduced by treatment of 
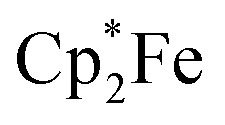
 to give the bis-borane adduct (**5c**) of the respective 9,10-anthracenediolate dianion. The compound was characterized by X-ray diffraction. Table 2 shows a comparison of the structural parameters of the radical anion system **11c** with the dianion system **5c**. The structure of compound **5c** is depicted in the ESI.†

**Table 2:** Comparison of selected structural parameters of compounds **11c** and **5c** (both with 
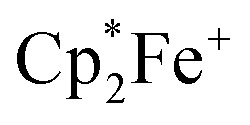
 countercations)^*a*^

Compounds	**11c**	**5c**
O1–B1	1.519 (3)	1.499 (7)
O1–C41	1.304 (3)	1.356 (6)
C41–C42	1.438 (3)	1.402 (7)
C42–C43	1.411 (3)	1.442 (7)
C42–C47	1.425 (3)	1.437 (7)
B1–O1–C41	137.6 (2)	128.5 (4)

^*a*^Bond lengths in Å; angles in °.

**Fig. 4:**
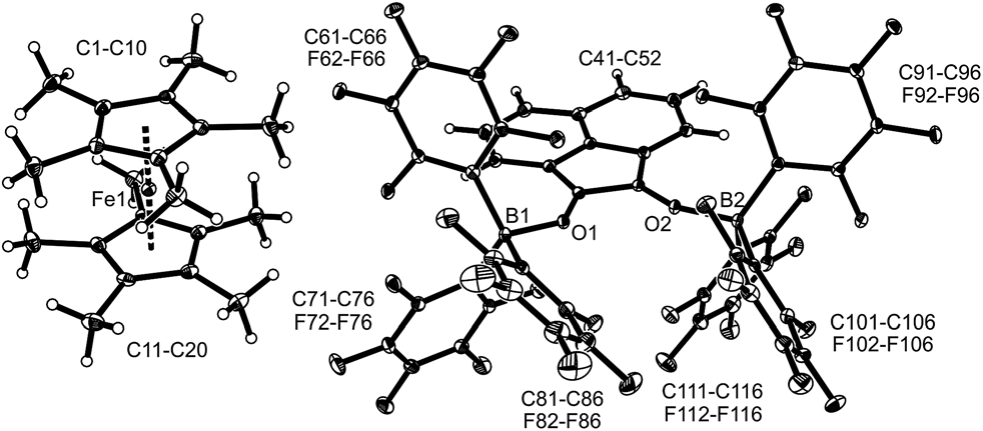
A view of the molecular structure of compound **12c** (thermal ellipsoids are shown with 15% probability). Selected bond lengths (Å) and angles (°): B1–O1 1.521(4), B2–O2 1.518 (4), O1–C41 1.285 (3), O2–C52 1.276 (3), C41–C52 1.453 (4), B1–O1–C41 130.1 (2), and B2–O2–C52 134.0 (2).

The reactions of acenaphthenequinone (**3c**) with the single electron donors used in this study are slightly more complicated. We made sure that **3c** did not react with TEMPO, the Gomberg dimer or 
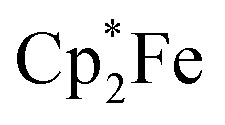
 under our typical conditions in the absence of the activating B(C_6_F_5_)_3_ Lewis acid. However, acenaphthenequinone was not reduced by the TEMPO radical even in the presence of two molar equiv. of B(C_6_F_5_)_3_. The trityl radical is a stronger electron donor than TEMPO.^16^,^20^ We found that the exposure of **3c** to the Gomberg dimer at r.t. (0.5 equiv. 1 h in CD_2_Cl_2_) in the presence of two molar equiv. of B(C_6_F_5_)_3_ led to the generation of the respective acenaphthene-semiquinone bis[B(C_6_F_5_)_3_] product **12b**. The dark green solution was investigated by NMR spectroscopy, which indicated the formation of a paramagnetic product. We could not isolate the very sensitive product **12b** in bulk as a clean solid but obtained a few dark green single crystals by low temperature (–35 °C, 7 d) crystallization from dichloromethane/pentane by the diffusion method. This allowed for the characterization of compound **12b** by X-ray diffraction (the structure is depicted in the ESI†). The compound features two strong boron–oxygen linkages [B1–O1: 1.522(5) Å, B2–O2: 1.514(5) Å, and adjacent O–C bonds: 1.282(4) Å (O1–C41), 1.279(4) Å (O2–C52)]; the connecting C–C bond is rather long [1.453(5) Å (C41–C52)] (atom numbering as for **12c**, see Fig. 4 below).

**Scheme 5:**
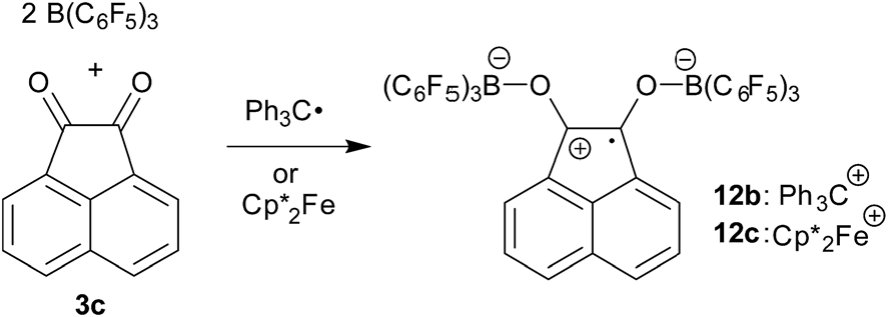


The reaction of a red acenaphthenequinone (**3c**)/2 B(C_6_F_5_)_3_ solution with one molar equiv. of 
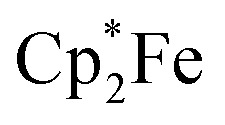
 in CD_2_Cl_2_ gave a dark green solution, from which the crystalline compound **12c** precipitated upon cooling to –35 °C (24 h). The 
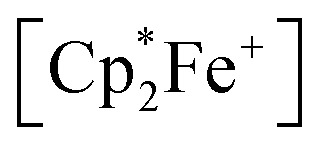
 acenaphthene-semiquinone·2 B(C_6_F_5_)_3_ product **12c** (Scheme 5) was characterized by C, H elemental analysis and ESR spectroscopy (broad signal at *g* = 2.0046) in solution (CH_2_Cl_2_). In solution the acenaphthene-semiquinone core of compound **12c** is NMR silent. We only observed a single broadened ^19^F NMR signal of the –B(C_6_F_5_)_3_ moieties and a typically paramagnetically shifted 
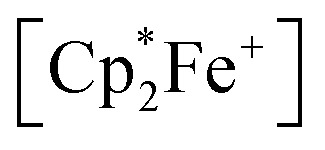
 ^1^H NMR resonance at *δ* = –36.7. The X-ray crystal structure analysis shows the presence of the 
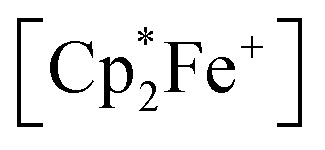
 cation. The acenaphthene-semiquinone radical anion has a pair of –B(C_6_F_5_)_3_ units attached to its oxygen atoms. The B–O bonds are oriented close to anti-periplanar at the framework. The bonding parameters of **12c** and **12b** (see above) are similar (see Fig. 4 and the ESI† for details).

The semiquinone salt **12c** reacted only slowly with tetrahydrofuran; after 72 h there was still some **12c** left. The reaction of **12c** with DMSO was much faster. Within 1 h at r.t. back electron transfer was complete to give acenaphthenequinone (**3c**), 
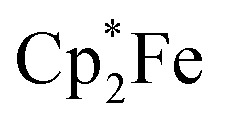
 and the (DMSO)B(C_6_F_5_)_3_ adduct (for details see the ESI†).

**Scheme 6:**
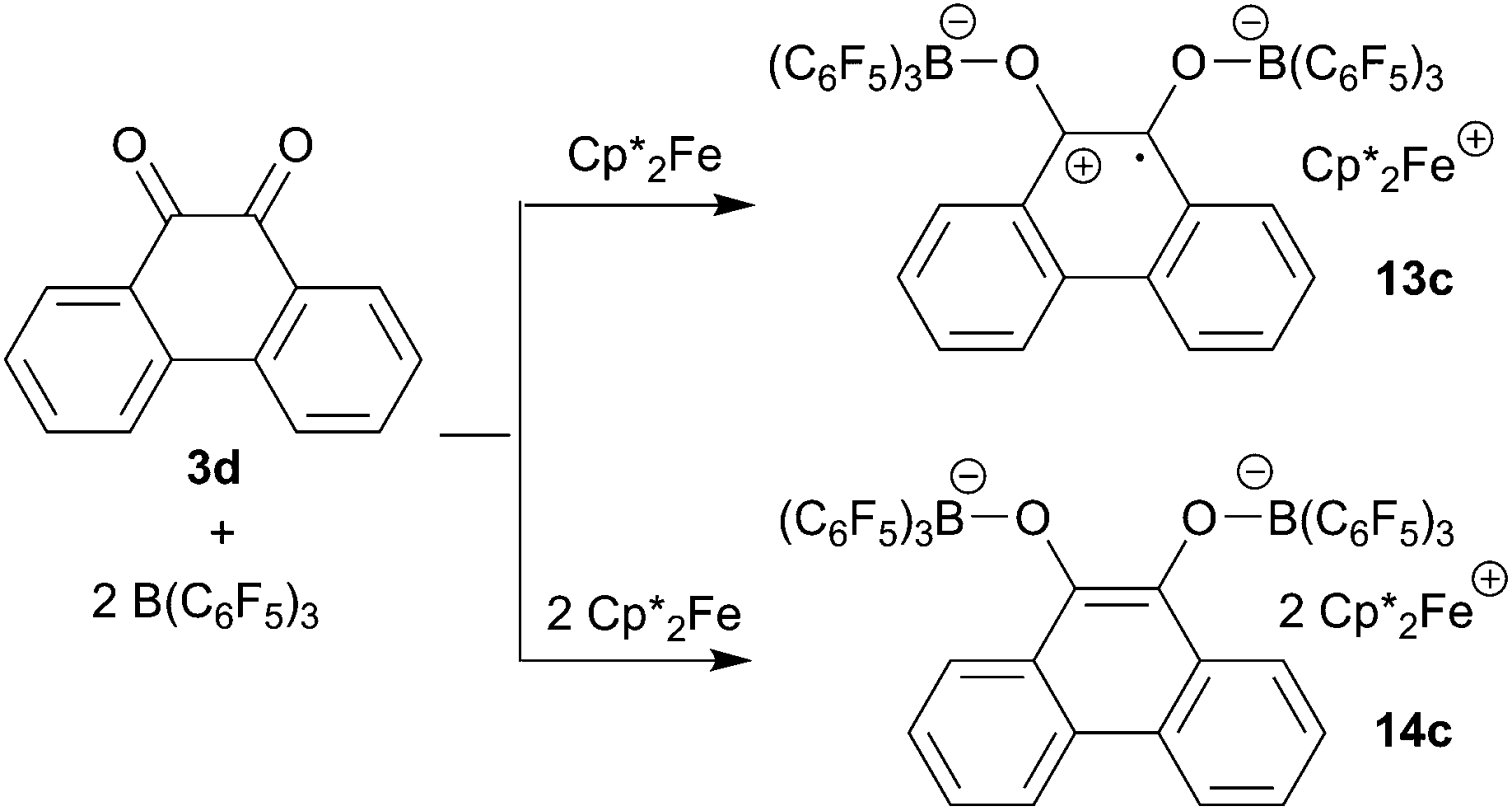


9,10-Phenanthrenequinone (**3d**) did not react with the electron transfer reagents in the absence of B(C_6_F_5_)_3_. However, the reaction of B(C_6_F_5_)_3_ with **3d** (2 : 1 molar ratio) in dichloromethane followed by the treatment of the resulting dark brown solution with 
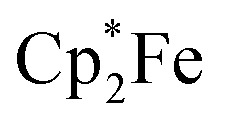
 (one molar equiv.)^21^ gave the respective [(C_6_F_5_)_3_B]_2_–9,10-phenanthrene–semiquinone radical anion, which was isolated as the 
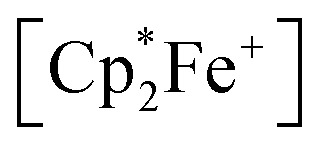
 salt **13c** as a brown crystalline material in 81% yield after workup involving crystallization at –35 °C (Scheme 6). Compound **13c** was characterized by C, H elemental analysis. In dichloromethane solution compound **13c** showed an ESR signal at 2.0047 *g* with a septet-like hyperfine coupling pattern. Our DFT-supported simulations suggest that this pattern is mainly caused by hyperfine interactions with the adjacent ^1^H nuclei (see the ESI† for details), whereas the nuclear hyperfine interactions with ^11^B and ^10^B are significantly weaker (A(^11^B) = 1.3 MHz). Similar results were previously obtained for a radical of a similar structure exhibiting only one B(C_6_F_5_)_2_ moiety.13*a*

We have also reacted the quinone **3d** with two molar equiv. of 
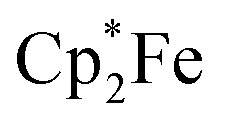
 in the presence of two molar equiv. of B(C_6_F_5_)_3_. This resulted in a reduction to the bis-alkoxide, isolated as the two-fold B(C_6_F_5_)_3_*O*-borylated dianion with two 
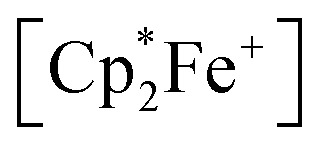
 countercations. The green crystalline product **14c** was isolated in 95% yield. It was characterized by C, H elemental analysis. The low temperature NMR spectra (233 K) show that the two parts of the dianion are symmetry-equivalent. We observed four separate equal intensity ^1^H NMR CH signals of the aromatic nucleus. The ^19^F NMR spectrum shows six *o*-F signals of the three boron bound –C_6_F_5_ groups of the symmetry equivalent pair of –B(C_6_F_5_)_3_ groups, three *p*-F and six *m*-F resonances. This indicates a frozen conformation of these groups at 233 K on the NMR time scale. The ^11^B NMR feature is at *δ* = –2.7 and there is a broad 
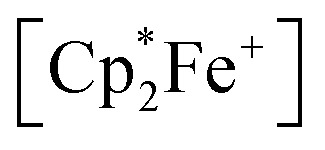
 methyl ^1^H NMR resonance at *δ* = –50.3 (see the ESI† for details).

**Fig. 5:**
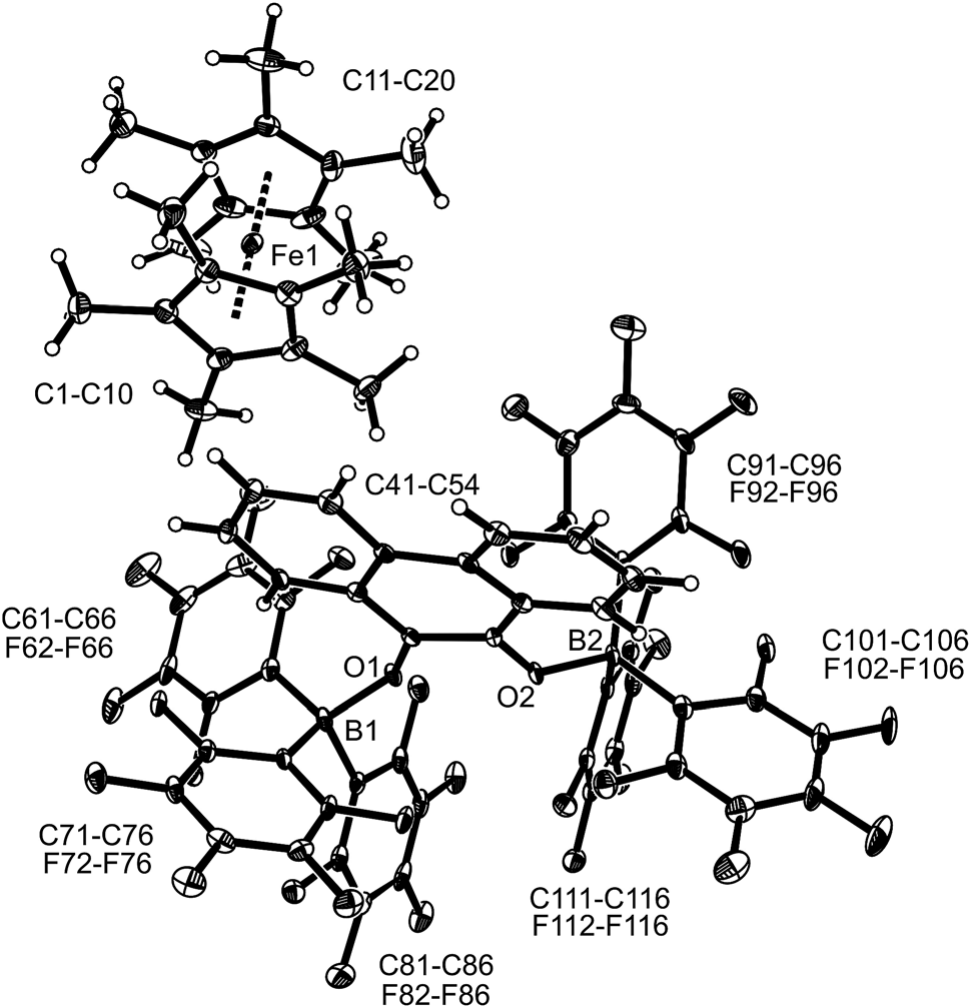
A projection of the molecular structure of 9,10-phenanthrene-semiquinone radical anion/
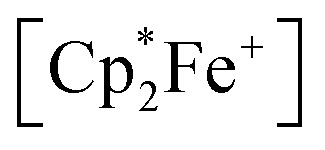
 salt **13c** (thermal ellipsoids are shown with 30% probability).

Both the semiquinone radical anion salt **13c** and the dianion salt **14c** were characterized by X-ray diffraction (see Table 3 for a structural comparison of the same core data). The semiquinone radical anion in **13c** shows rather long B–O bonds (Table 3) which probably indicates a marked structural quinone resemblance. In contrast, the B–O bonds of the diolate dianion in **14c** are much shorter. Consistently, the O–C bonds in **13c** are short, featuring a remaining partial carbon–oxygen double bond character. The C–O bonds in **14c** are markedly longer. The connecting C–C bond in **14c** shows a typical double bond character, while the connecting C–C bond in **13c** is much longer, almost with a typical value of a C(sp^2^)–C(sp^2^) single bond. The structure of the 9,10-phenanthrene-semiquinone salt is depicted in Fig. 5; see the ESI† for a projection of the structure of the dianion 
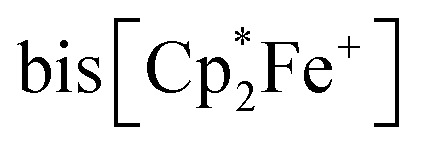
 salt **14c**.

**Table 3:** A comparison of selected structure parameters between compounds **13c** and **14c**^*a*^

Compounds	**13c**	**14c**
O1–B1	1.524 (7)	1.498 (6)
O2–B2	1.523 (7)	1.481 (6)
O1–C41	1.308 (6)	1.366 (5)
O2–C54	1.295 (6)	1.357 (5)
C41–C54	1.458 (7)	1.370 (6)
B1–O1–C41	131.9 (4)	124.9 (3)
B2–O2–C54	134.6 (4)	125.7 (3)

^*a*^Bond lengths in Å; angles in °.

The reduction of the semiquinone–[B(C_6_F_5_)_3_]_2_ Lewis acid adducts **8** to the borylated hydroquinone dianion systems was also investigated by cyclic voltammetry. For the parent *p*-benzosemiquinone–[B(C_6_F_5_)_3_]_2_ system cyclic voltammetry experiments revealed a redox potential of –0.03 V *vs.* the ferrocene/ferrocenium redox couple for the reduction of the semiquinone **8c** to the *O*-borylated hydroquinone dianion **5a** in dichloromethane (0.1 M Bu_4_NPF_6_). The reduction of *p*-benzoquinone **3a** to the semiquinone and dianion in dichloromethane (0.1 M Bu_4_NPF_6_) in the absence of B(C_6_F_5_)_3_ occurs at redox potentials of –0.99 V and approx. –1.8 V *vs.* Fc/Fc^+^, respectively (see the ESI† for the voltammograms and additional information). Hence, the adduct formation of **3a** with B(C_6_F_5_)_3_ leads to a significant shift (approx. 1.8 V) of the redox potentials which is just in accordance with the experimental observations.^22^

Unfortunately, the first reduction of the quinones to the semiquinone stage in the presence of tris(pentafluorophenyl)borane remained inconclusive in our cyclic voltammetry experiments under various conditions. Hence, for a qualitative assessment we looked at the redox potentials of the uncomplexed quinones instead. 9,10-Anthraquinone **3b** is reduced at –1.49 V and –2.0 V *vs.* Fc/Fc^+^, respectively, under our typical cyclic voltammetry conditions (in dichloromethane, 0.1 M Bu_4_NPF_6_). The reduction to the dianion stage could not be observed for acenaphthenequinone **3c** under the described experimental conditions and is, therefore, expected to occur at <–2.1 V *vs.* Fc/Fc^+^. The first reduction step takes place at –1.43 V *vs.* Fc/Fc^+^. The reduction of 9,10-phenanthrenequinone **3d** to the semiquinone and subsequently the dianion occurs at –1.13 V and –1.8 V *vs.* Fc/Fc^+^ in dichloromethane (0.1 M Bu_4_NPF_6_). The redox potentials of the reducing agents (decamethylferrocene: –0.59 V *vs.* Fc/Fc^+^; trityl radical: –0.18 V *vs.* Fc/Fc^+^; TEMPO: 0.24 V *vs.* Fc/Fc^+^) were determined under the same experimental conditions. This sequence in redox potentials might qualitatively explain why TEMPO, the weakest of the here utilized reductants, was only able to reduce *p*-benzoquinone to the borane stabilized semiquinone under our conditions in the presence of B(C_6_F_5_)_3_ but failed to do so for the benzannulated quinones. We could positively show that anthraquinone **3b** and acenaphthenquinone **3c** were reduced readily to the respective borylated semiquinones by trityl radical, and decamethylferrocene reduced all of the quinones investigated here in the presence of the B(C_6_F_5_)_3_ Lewis acid.

## Conclusions

We have found that the here studied quinones did not react with a stoichiometric amount of the members of the trio of electron donor reagents used in this study, namely TEMPO, trityl radical and decamethylferrocene, under our typical conditions in the absence of B(C_6_F_5_)_3_. The addition of two molar equiv. of the borane Lewis acid then resulted in a rapid and complete single electron transfer in the 1 : 1 stoichiometry experiments in these cases. In some examples the energetically least potent reductant, the TEMPO radical, was inert towards oxidation by some quinones, but mostly, single electron transfer was possible and rapidly realized. In some cases further reduction to the diamagnetic dianion could be realized.

The X-ray crystal structure analyses of the resulting semiquinone–[B(C_6_F_5_)_3_]_2_ products revealed some interesting structural effects: the C=O bonds in the various O–B(C_6_F_5_)_3_ borylated semiquinone systems investigated in our study are almost identical in lengths to the reported structurally characterized free semiquinone examples.^6^–^9^ In addition, the B–O lengths are rather long as compared to the respective borylated hydroquinone systems (which consequently show much longer C–O bonds). So, we conclude that the attachment of the boranes to the pairs of semiquinone oxygen atoms has only a marginal structural effect. Consequently, the essential role of the added B(C_6_F_5_)_3_ Lewis acid component seems to be kinetic quinone activation and thermodynamic semiquinone radical anion stabilization, but structurally, the attachment of the pair of B(C_6_F_5_)_3_ Lewis acid moieties seems to only marginally alter the structural features of the semiquinone radical anions.

The obtained systems showed another interesting feature of potentially general significance. The systems were prone to rapid back electron transfer. This became visible upon addition of suitable donor reagents, tetrahydrofuran or even better dimethyl sulfoxide, which trapped B(C_6_F_5_)_3_ from the borylated semiquinone radical anions to form the respective THF–B(C_6_F_5_)_3_ or DMSO–B(C_6_F_5_)_3_ Lewis adducts, and thereby opened the way to efficient back electron transfer. This re-formed the respective radicals, *e.g.* TEMPO from TEMPO^+^, and regenerated the quinone systems (or products derived from them). So, in addition to promoting the formation of the semiquinone radical anion stage, the B(C_6_F_5_)_3_ Lewis acid serves in a role that apparently makes electron-shuttling between semiquinone radical anions and quinone oxidation states easier. We will see if that may lead to a closer similarity of such well-characterized molecular electron-transfer models in their function close to the related systems in biology.

## Conflicts of interest

There are no conflicts to declare.

## Supplementary Material

Supplementary information

Crystal structure data
