# Supplementary material for: The special role of B(C6F5)3 in the single electron reduction of quinones by radicals
Source: Chem Sci. 2018 Aug 20;9(41):8011–8. doi: 10.1039/c8sc03005g (PMC6202917; doi:10.1039/c8sc03005g)
Supplement: Supplementary file 1 [file SC-009-C8SC03005G-s001.pdf]

Electronic Supplementary Material (ESI) for Chemical Science.
This journal is © The Royal Society of Chemistry 2018

Supporting Information

The Special Role of $B(C_6F_5)_3$ in the Single Electron Reduction of Quinones by Radicals

Xin Tao,[a] Constantin G. Daniliuc,[a] Robert Knitsch,[b] Michael Ryan Hansen,[b] Hellmut Eckert,[b,c] Maximilian Lübbesmeyer,[a] Armido Studer,[a] Gerald Kehr[a] and Gerhard Erker[a]*

[a] Organisch-Chemisches Institut, Westfälische Wilhelm-Universität Münster, Corrensstraße 40, 48149 Münster, Germany,

[b] Institut für Physikalische Chemie der Universität Münster, Corrensstraße 28, 48149 Münster, Germany,

[c] Instituto de Fisica, Universidade de Sáo Paulo, CP 369, 13560-970, Sáo Carlos, S.P. Brasil.

Contents

Supporting Information: analytical and experimental details S3

General Information S3

A) Complexation of p-benzoquinone with $B(C_6F_5)_3$ S5

B) Synthesis of compound **8a** (**8a-D**) S10

C) Synthesis of compound **8b** S17

D) Synthesis of compound **8c** (**8c-D**) S25

E) Reaction of 9,10-anthraquinone, 2 $B(C_6F_5)_3$ and TEMPO S29

F) Generation of compound **11b** S31

G) Synthesis of compound **11c** S36

H) Reaction of acenaphthenequinone, 2 $B(C_6F_5)_3$ and TEMPO S41

I) Generation of compound **12b** S43

J) Synthesis of compound **12c** S48

K) Reaction of 9,10-phenanthrenequinone, 2 $B(C_6F_5)_3$ and TEMPO S52

L) Synthesis of compound **13c** S54

M) Reaction of compound **8a** with Cp^*_2Fe S59

N) Reaction of compound **11c** with Cp^*_2Fe S61

O) Reaction of compound **12c** with Cp^*_2Fe S64

P) Reaction of compound **13c** with Cp^*_2Fe S66

Q) Reaction of compound **8a** with THF and DMSO, respectively S74

R) Reaction of compound **8b** with THF and DMSO, respectively S78

S) Reaction of compound **8c** with THF and DMSO, respectively S82

T) Reaction of compound **11c** with THF and DMSO, respectively S86

U) Reaction of compound **12c** with THF and DMSO, respectively S90

Cyclic voltammetry S95

EPR spectroscopy on tris(pentafluorophenyl)borane (BCF) stabilized semiquinones S103

Supporting Information: experimental and analytical details

Xin Tao, Constantin G. Daniliuc, Gerald Kehr and Gerhard Erker

Organisch-Chemisches Institut, Westfälische Wilhelm-Universität Münster, Corrensstraße 40, 48149 Münster, Germany

General Information. All reactions involving air- or moisture-sensitive compounds were carried out under an inert gas atmosphere (Argon) by using Schlenk-type glassware or in a glovebox. All solvents were dried and degassed before use, if necessary for the respective reaction. Chemicals: Unless otherwise noted all chemicals were used as purchased. The following instruments were used for physical characterization of the compounds: melting points: TA-instrument DSC Q-20; elemental analyses: Foss-Heraeus CHNO-Rapid; NMR: Varian UNITY plus NMR spectrometer (^{1}H, 600 MHz; ^{13}C, 151 MHz; ^{11}B, 192 MHz; ^{19}F, 564 MHz; ^{31}P, 243 MHz). NMR chemical shifts are given relative to $SiMe_4$ and referenced to the respective solvent signals (^{1}H and ^{13}C) or external standard [δ($BF_3{\cdot}OEt_2$) = 0 for ^{11}B NMR, δ($CFCl_3{\cdot}OEt_2$) = 0 for ^{19}F NMR].

X-Ray diffraction: For compounds **5c**, **8a**, **8c** und **12c** sets were collected with a Nonius Kappa CCD diffractometer. Programs used: data collection, COLLECT (R. W. W. Hooft, Bruker AXS, **2008**, Delft, The Netherlands); data reduction Denzo-SMN (Z. Otwinowski, W. Minor, *Methods Enzymol.* **1997**, *276*, 307-326); absorption correction, Denzo (Z. Otwinowski, D. Borek, W. Majewski, W. Minor, *Acta Crystallogr*. **2003**, *A59*, 228-234); structure solution SHELXS-97 (G. M. Sheldrick, *Acta Crystallogr*. **1990**, *A46*, 467-473); structure refinement SHELXL-97 (G. M. Sheldrick, *Acta Crystallogr*. **2008**, *A64*, 112-122). For compound **11b**, **12b**, **13c**, **14c** data sets were collected with a Bruker APEX II CCD diffractometer. Data sets for compounds **8a(4D)**, **8b** and **11c** were collected with a D8 Venture CMOS diffractometer. Programs used: data collection: APEX3 V2016.1-0 (Bruker AXS Inc., **2016**); cell refinement: SAINT V8.37A (Bruker AXS Inc., **2015**); data reduction: SAINT V8.37A (Bruker AXS Inc., **2015**); absorption correction, SADABS V2014/7 (Bruker AXS Inc., **2014**); structure solution SHELXT-2015 (Sheldrick, **2015**); structure refinement SHELXL-2015 (Sheldrick, **2015**) and graphics, XP (Bruker AXS Inc., **2015**). *R*-values are given for observed reflections, and wR^2 values are given for all reflections. *Exceptions and special features*: For compounds **11b** and **12b** two dichloromethane molecules, for compounds **11c** and **13c** one dichloromethane molecule and for compound **14c** one Cp* group were found disordered over two positions in the asymmetric unit. Several restraints (SADI, SAME, ISOR and SIMU) were used in order to improve refinement stability. For compounds **8a** and **14c** two dichloromethane molecules and for compounds **12c** and **13c** one dichloromethane molecule were found in the asymmetrical unit and could not be satisfactorily refined. The program SQUEEZE (A. L. Spek (**2015**) Acta Cryst., C71, 9-18) was therefore used to remove mathematically the effect of the solvent. The quoted formula and derived parameters are not included the squeezed solvent molecules.

Materials. $B(C_6F_5)_3$ was obtained from Boulder Scientific and used after crystallization from pentane. Decamethylferrocene, *p*-benzoquinone (PBQ), 9,10-anthraquinone, acenaphthenequinone, 9,10-phenanthrenequinone and TEMPO were purchased from Sigma-Adrich and used as received. Gomberg dimer [Heurich, T.; Nesterov, V.; Schnakenburg, G.; Qu, Z.-W.; Grimme, S.; Hazin, K.; Gates, D. P.; Engeser, M.; Streubel, R. *Angew. Chem. Int. Ed.* **2016**, *55*, 14439.] was synthesized according to the procedures described in the literature.

A) Complexation of p-benzoquinone with $B(C_6F_5)_3$

Scheme S1.

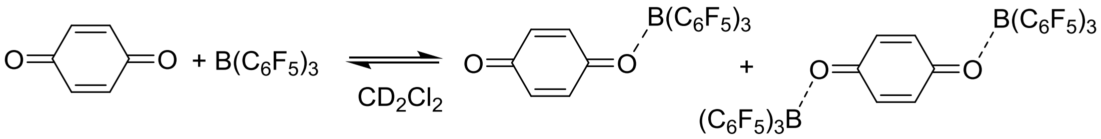

Experiment 1: (mixture of p-benzoquinone and $B(C_6F_5)_3$ in molar ratio 2:1)
p-Benzoquinone (5.4 mg, 0.05 mmol) and $B(C_6F_5)_3$ (12.8 mg, 0.025 mmol) were dissolved in CD_2Cl_2 (0.5 mL) at room temperature. Subsequently, the resulting reaction mixture was characterized by NMR experiments.
[NMR spectra see Figure S5-7]

Experiment 2: (mixture of p-benzoquinone and $B(C_6F_5)_3$ in molar ratio 1:1)
p-Benzoquinone (5.4 mg, 0.05 mmol) and $B(C_6F_5)_3$ (25.6 mg, 0.05 mmol) were dissolved in CD_2Cl_2 (0.5 mL) at room temperature. Subsequently, the resulting reaction mixture was characterized by NMR experiments.

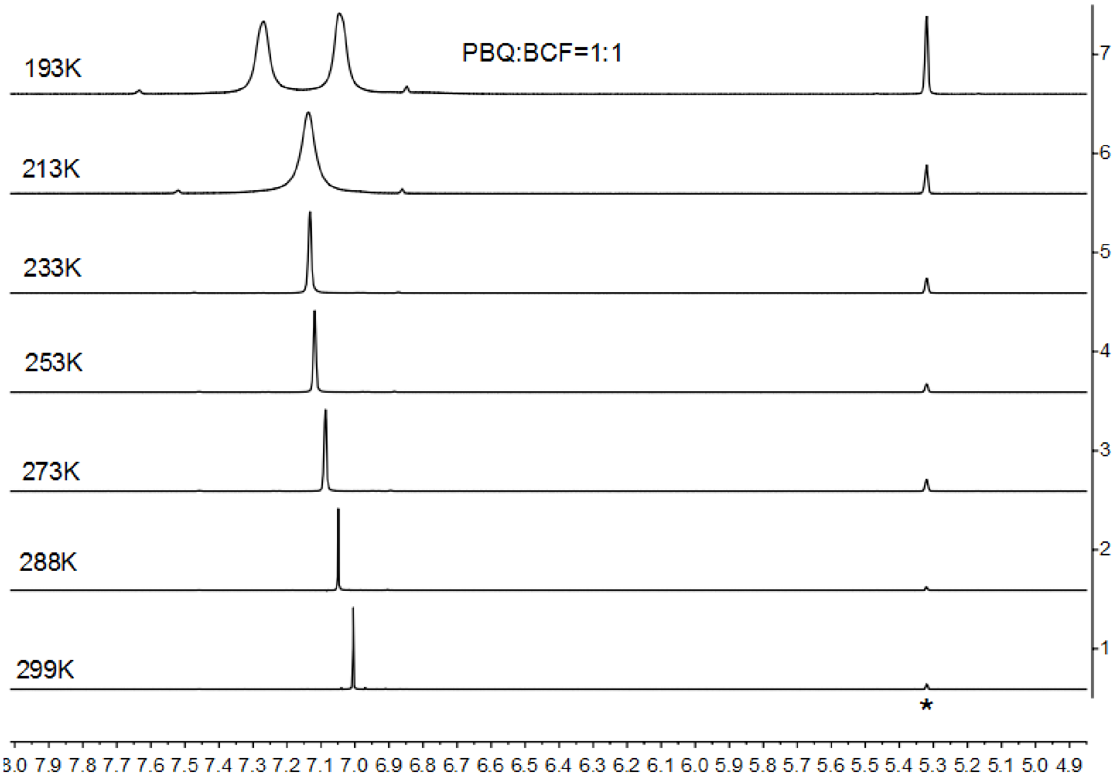

Figure S1. ^1H NMR (600 MHz, CD_2Cl_2*) spectra of reaction mixture by Experiment 2 at variable temperature. [PBQ: p-benzoquinone; BCF: $B(C_6F_5)_3$]

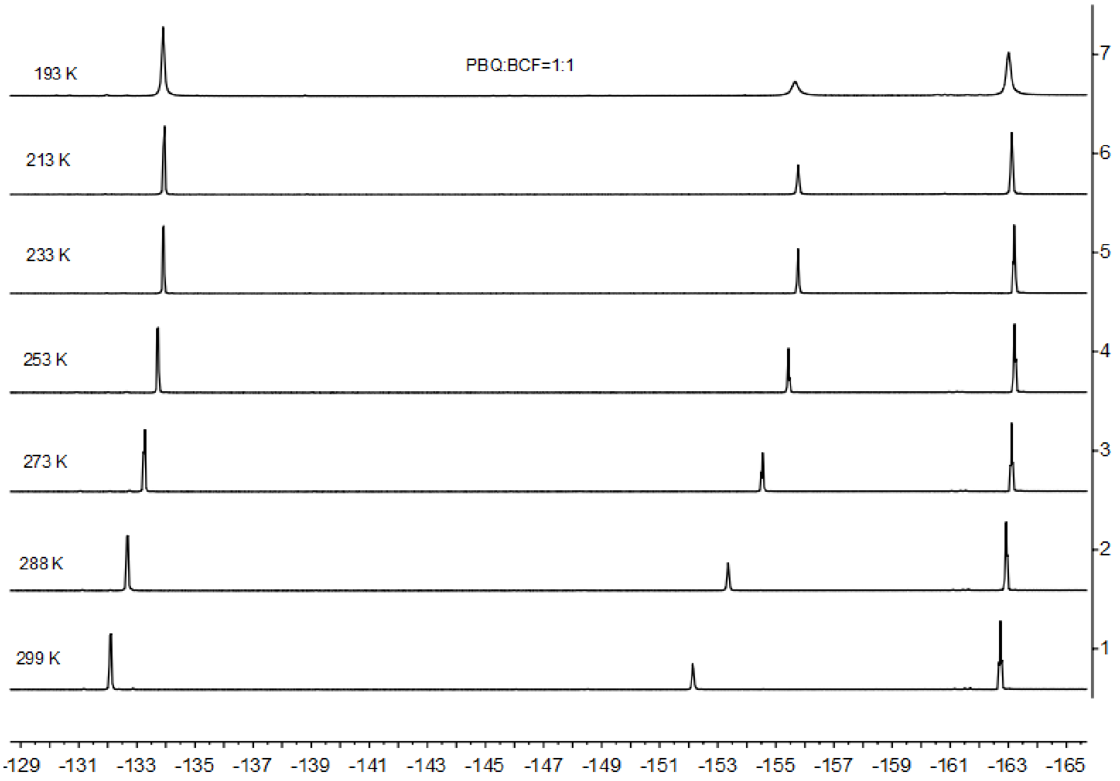

Figure S2. ^{19}F NMR (564 MHz, CD_2Cl_2) spectra of reaction mixture by Experiment 2 at variable temperature. [PBQ: p-benzoquinone, BCF: $B(C_6F_5)_3$]

Experiment 3: (mixture of p-benzoquinone and $B(C_6F_5)_3$ in molar ratio 1:1.5)

p-Benzoquinone (5.4 mg, 0.05 mmol) and $B(C_6F_5)_3$ (38.4 mg, 0.075 mmol) were dissolved in CD_2Cl_2 (0.5 mL) at room temperature. Subsequently, the resulting reaction mixture was characterized by NMR experiments.

[NMR spectra see Figure S5-7]

Experiment 4: (mixture of p-benzoquinone and $B(C_6F_5)_3$ in molar ratio 1:2)

p-Benzoquinone (5.4 mg, 0.05 mmol) and $B(C_6F_5)_3$ (51.2 mg, 0.10 mmol) were dissolved in CD_2Cl_2 (0.5 mL) at room temperature. Subsequently, the resulting reaction mixture was characterized by NMR experiments.

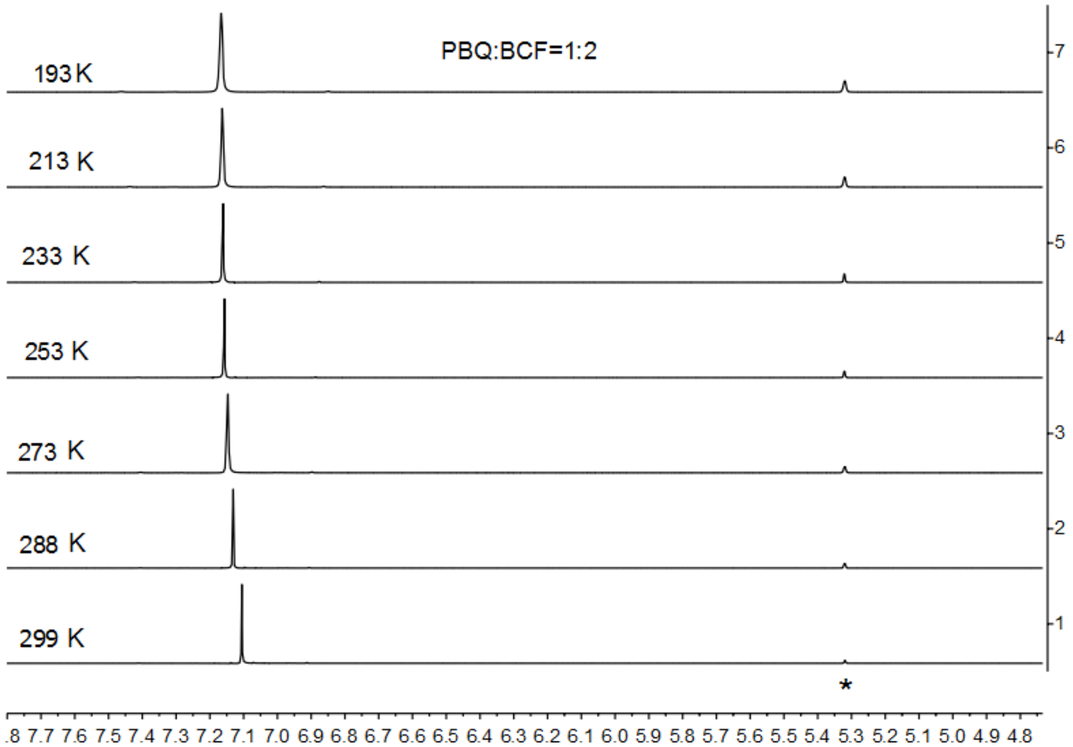

Figure S3. ^{1}H NMR (600 MHz, CD_2Cl_2*) spectra of reaction mixture by Experiment 4 at variable temperature. [PBQ: p-benzoquinone, BCF: $B(C_6F_5)_3$]

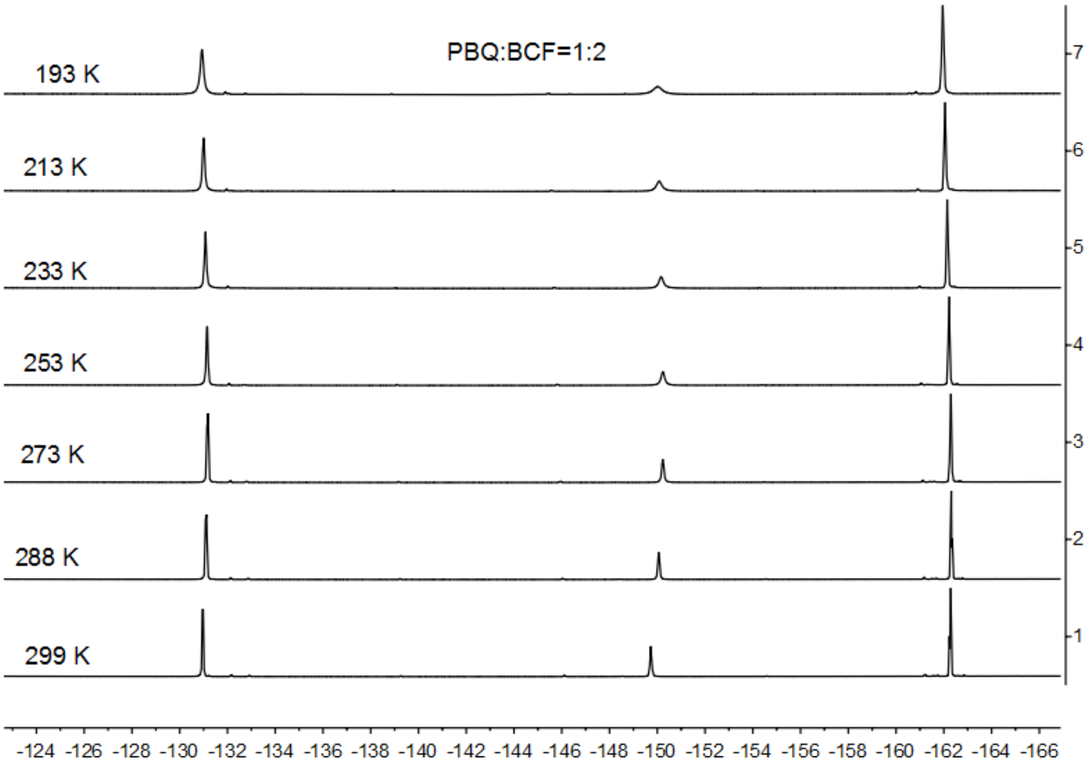

Figure S4. ^{19}F NMR (564 MHz, CD_2Cl_2) spectra of reaction mixture by Experiment 4 at variable temperature. [PBQ: p-benzoquinone, BCF: $B(C_6F_5)_3$]

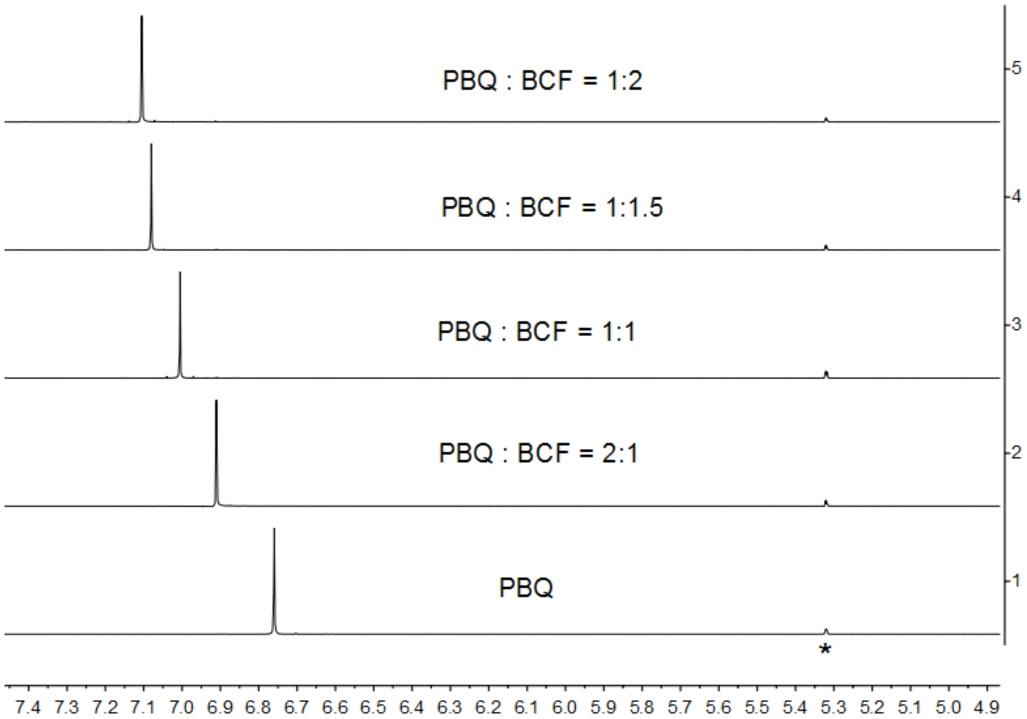

Figure S5. ^{1}H NMR (600 MHz, CD_2Cl_2*, 299 K) spectra of p-benzoquinone (spectrum 1) and reaction mixtures of p-benzoquinone and $B(C_6F_5)_3$ in ratios of 2:1 (spectrum 2, Experiment1), 1:1 (spectrum 3, Experiment 2), 1:1.5 (spectrum 4, Experiment 3) and 1:2 (spectrum 5, Experiment 4). [PBQ: p-benzoquinone, BCF: $B(C_6F_5)_3$]

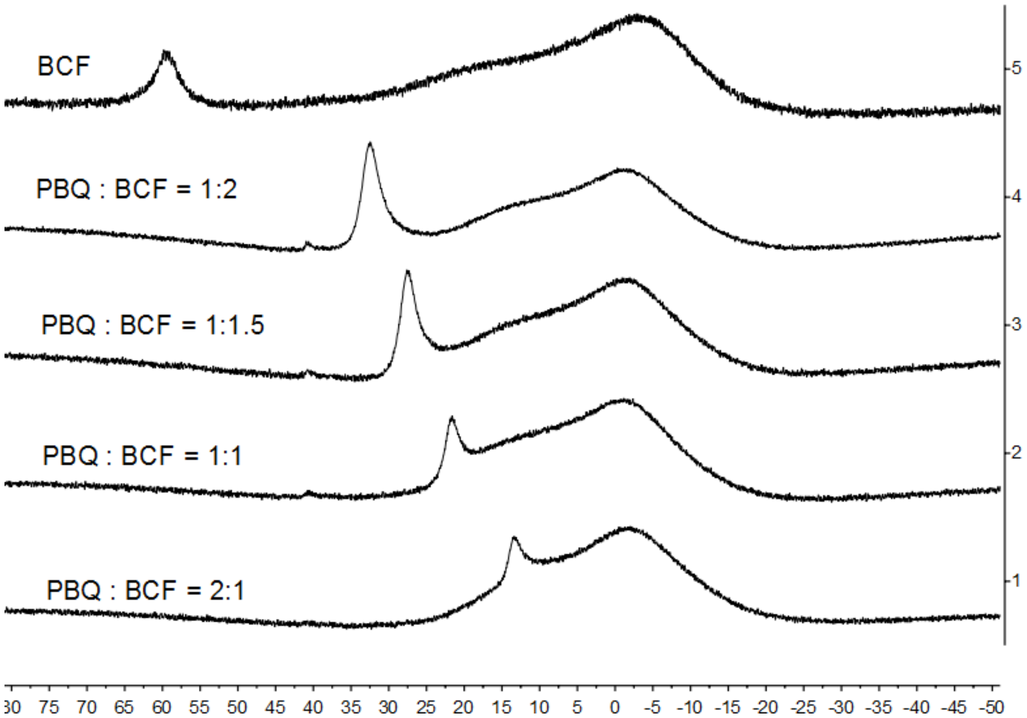

Figure S6. ^{11}B NMR (192 MHz, CD_2Cl_2, 299 K) spectra of reaction mixtures of p-benzoquinone and $B(C_6F_5)_3$ in ratios of 2:1 (spectrum 1, Experiment1), 1:1 (spectrum 2, Experiment 2), 1:1.5 (spectrum 3, Experiment 3), 1:2 (spectrum 4, Experiment 4) and $B(C_6F_5)_3$ (spectrum 5). [PBQ: p-benzoquinone, BCF: $B(C_6F_5)_3$]

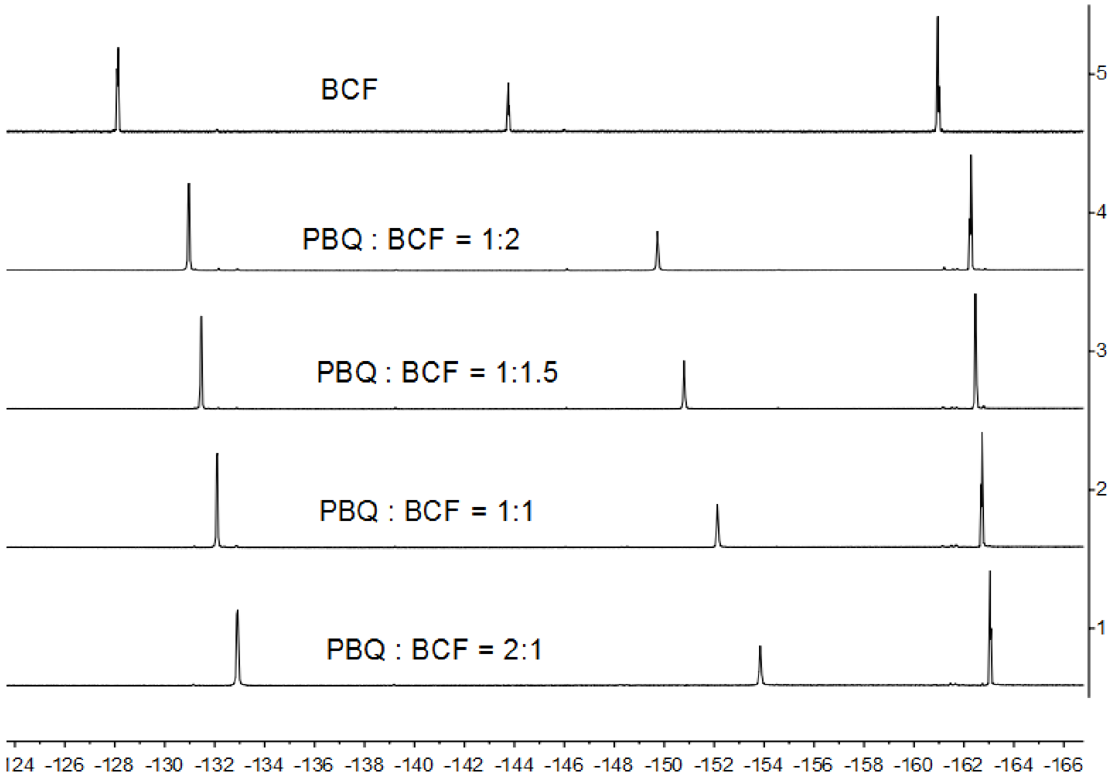

Figure S7. ^{19}F NMR (564 MHz, CD_2Cl_2, 299 K) spectra of reaction mixtures of p-benzoquinone and $B(C_6F_5)_3$ in ratios of 2:1 (spectrum 1, Experiment1), 1:1 (spectrum 2, Experiment 2), 1:1.5 (spectrum 3, Experiment 3), 1:2 (spectrum 4, Experiment 4) and $B(C_6F_5)_3$ (spectrum 5). [PBQ: p-benzoquinone, BCF: $B(C_6F_5)_3$]

B) Synthesis of compound **8a** (**8a-D**)

Experiment 1: (reaction of TEMPO with PBQ, NMR scale)

Scheme S2

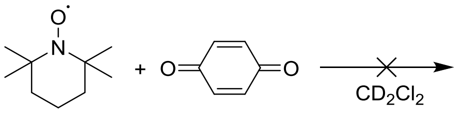

p-Benzoquinone (10.8 mg, 0.10 mmol) and TEMPO (15.6 mg, 0.10 mmol) were dissolved in CD_2Cl_2 (0.5 mL) at room temperature. The resulting reaction mixture was characterized by NMR experiments.

[Comment: no reaction was observed].

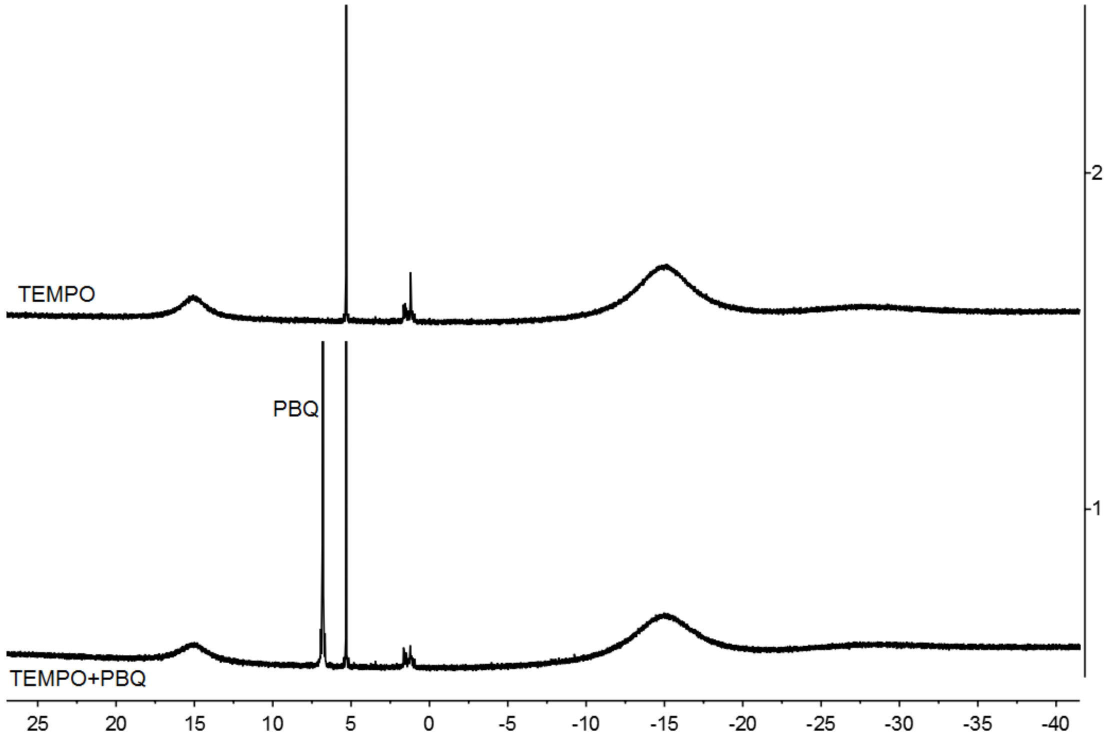

Figure S8. ^{1}H NMR (600 MHz, 299 K, CD_2Cl_2) spectra of (1) reaction mixture and (2) TEMPO. [PBQ: p-benzoquinone]

Experiment 2: (isolation of compound **8a**)

Scheme S3

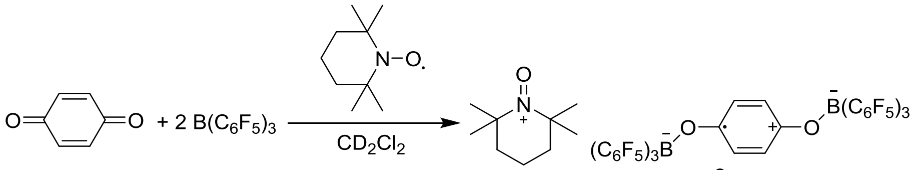

8a

p-Benzoquinone (21.6 mg, 0.20 mmol) and $B(C_6F_5)_3$ (204 mg, 0.40 mmol) were dissolved in CD_2Cl_2 (1 mL) at room temperature and the resulting red solution was characterized by NMR experiments. A solution of TEMPO (31.2 mg, 0.20 mmol) in CD_2Cl_2 (1 mL) was added to the reaction mixture at room temperature. After stirring for 10 min at room temperature, the resulting a dark brown solution was characterized by NMR experiments. Diffusion of pentane vapor to the resulting reaction mixture at –35 ^oC during 48 h yielded the dark brown crystalline material. Removal of the supernatant solution by decantation and drying of the remaining solid in vacuo gave compound **8a** (152 mg, 0.37 mmol, 59%) as brown crystalline material.

Decomp.: 155 ^oC

Anal. Calc. for $C_{51}H_{22}B_2F_{30}NO_3$: C, 47.55; H, 1.72; N, 1.09. Found: C, 47.45; H, 2.07; N, 1.11.

NMR data of compound **8a** in the reaction mixture:

[TMP: 2,2,6,6-tetramethylpiperidino]

^{1}H NMR (600 MHz, 299 K, CD_2Cl_2): δ ^{1}H: 2.54 (br, 6H, CH_2^{TMP}), 1.73 (br, 12H, CH_3^{TMP}). See Figure S9.

^{19}F NMR (564 MHz, 299 K, CD_2Cl_2): δ ^{19}F: see Figure S11.

^{11}B{^{1}H} NMR (192 MHz, 299 K, CD_2Cl_2): δ ^{11}B: see Figure S10.

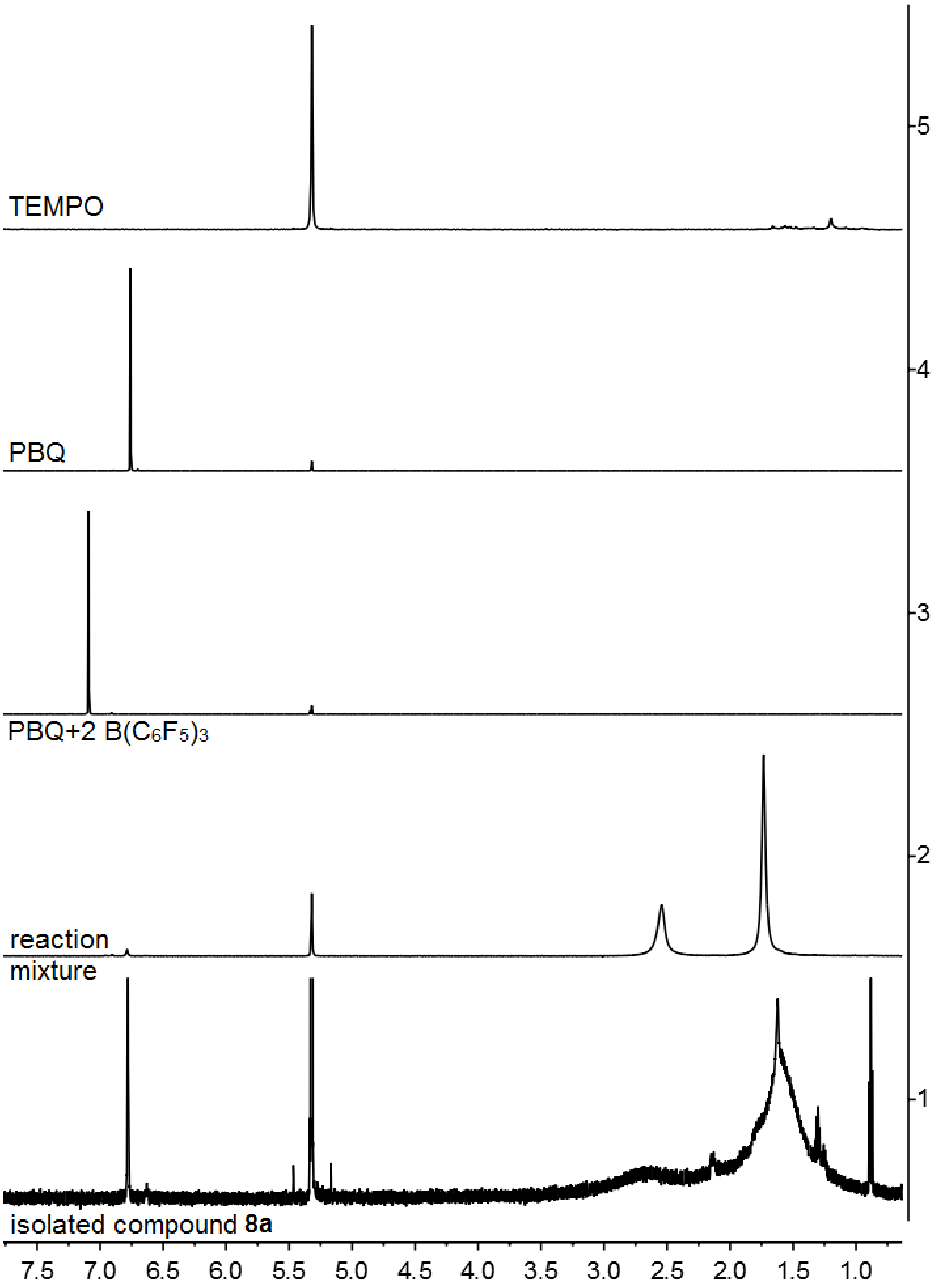

Figure S9. ^{1}H NMR (600 MHz, 299 K, CD_2Cl_2) spectra of (1) isolated compound **8a**, (2) reaction mixture of experiment 2, (3) a mixture of PBQ and 2 equiv. of $B(C_6F_5)_3$, (4) PBQ and (5) TEMPO. [PBQ: *p*-benzoquinone]

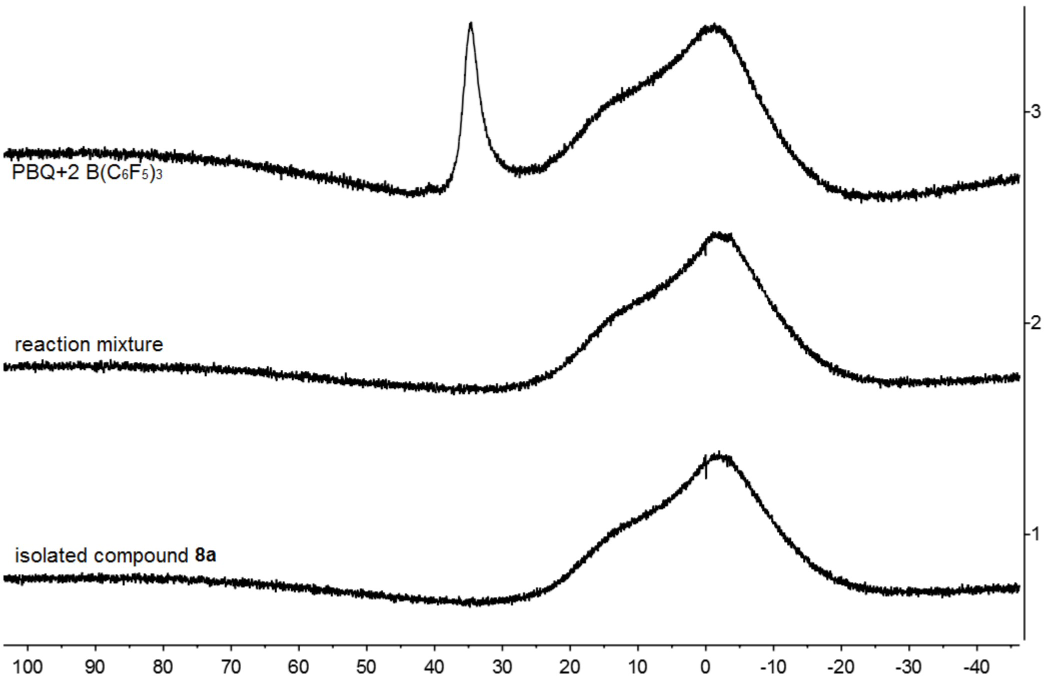

Figure S10. $^{11}B\{^{1}H\}$ NMR (192 MHz, 299 K, CD_2Cl_2) spectra of (1) isolated compound **8a**, (2) reaction mixture of experiment 2 and (3) a mixture of PBQ and 2 equiv. of $B(C_6F_5)_3$.

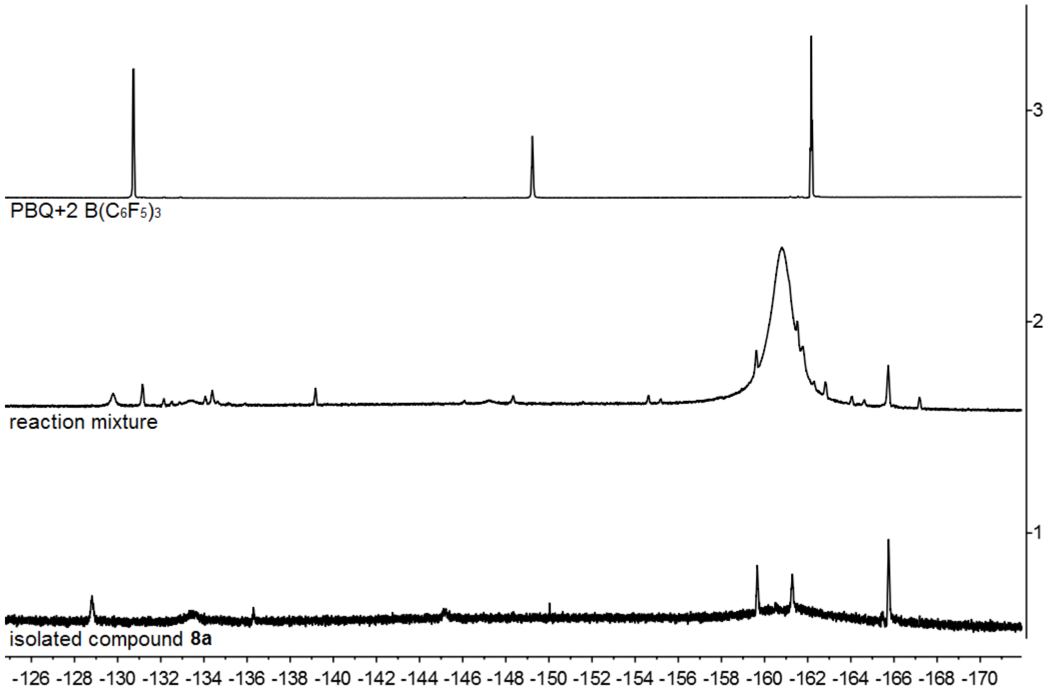

Figure S11. ^{19}F NMR (564 MHz, 299 K, CD_2Cl_2) spectra of (1) isolated compound **8a**, (2) reaction mixture of experiment 2 and (3) a mixture of PBQ and 2 equiv. Of $B(C_6F_5)_3$.

Crystals suitable for the X-ray crystal structure analysis were obtained from diffusion of pentane to a CD_2Cl_2 solution of compound **8a** at -35 ^{o}C.

X-ray crystal structure analysis of 8a (erk8721): formula $C_{51}H_{22}B_2F_{30}NO_3$, *M* = 1288.32, dark orange crystal, 0.20 x 0.16 x 0.02 mm, *a* = 13.8373(3), *b* = 14.8923(3), *c* = 15.4147(3) Å, α = 63.462(1), *β* = 80.689(1), γ = 88.721(2)°, *V* = 2799.9(1) Å^3, ρ_{calc} = 1.528 gcm^{-3}, μ = 0.163 mm^{-1}, empirical absorption correction (0.968 ≤ T ≤ 0.996), *Z* = 2, triclinic, space group $P\bar{1}$ (No. 2), λ = 0.71073 Å, *T* = 173(2) K, ω and ϕ scans, 29995 reflections collected (±*h*, ±*k*, ±*l*), 9622 independent (R_{int} = 0.045) and 7891 observed reflections [$I>2\sigma(I)$], 788 refined parameters, *R* = 0.048, wR^2 = 0.123, max. (min.) residual electron density 0.39 (-0.33) e.Å^{-3}, the position of the hydrogen were calculated and refined as riding atoms.

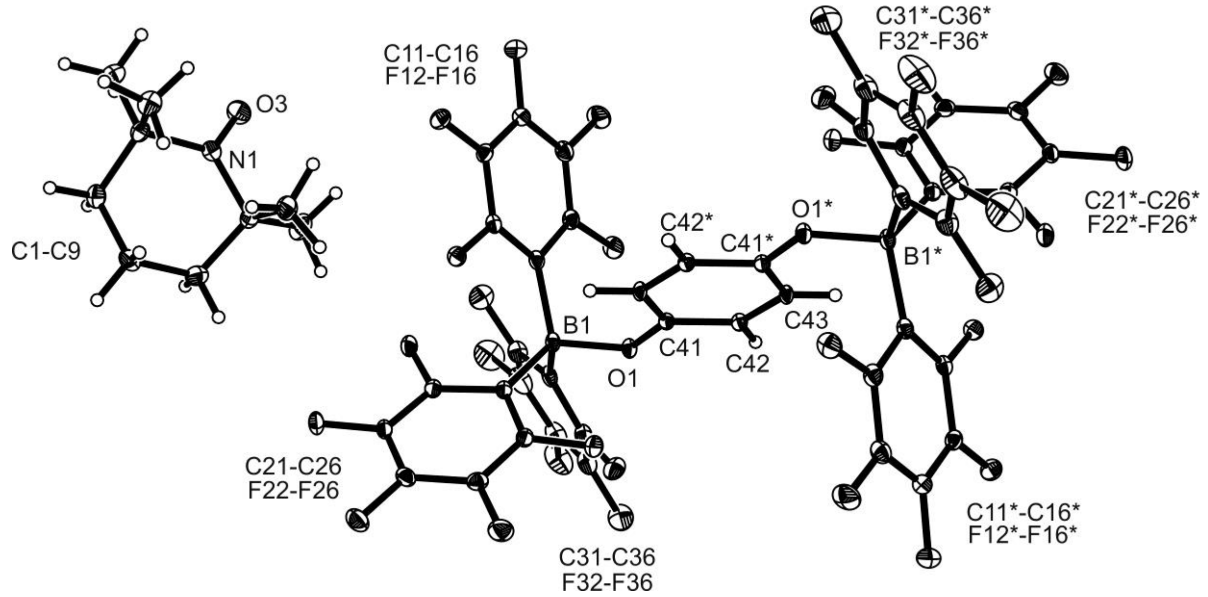

Figure S12. Crystal structure of compound **8a**. Only one half radical anion (completely generated over inversion centre) of two found in the asymmetric unit is shown. (Thermals ellipsoids are shown with 15% probability.)

Experiment 3: (isolation of compound **8a-D**)

Scheme S4

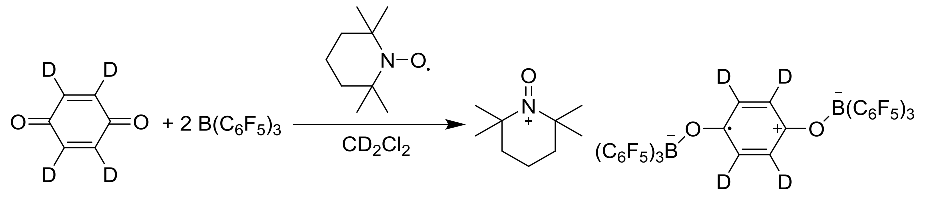

p-Benzoquinone-d$_4$ (22.4 mg, 0.20 mmol) and $B(C_6F_5)_3$ (204 mg, 0.40 mmol) were dissolved in CD_2Cl_2 (1 mL) at room temperature and characterized by NMR experiments. A solution of TEMPO (31.2 mg, 0.20 mmol) in CD_2Cl_2 (1 mL) was added to the reaction mixture at room temperature. After stirring for 10 min at room temperature, the resulting a dark brown solution was characterized by NMR

experiments. Diffusion of pentane to the resulting reaction mixture at –35 °C for 48 h yielded the dark brown crystalline material. Removal of the solution by decantation and drying of the remaining solid in vacuo gave compound **8a-D** (197 mg, 0.15 mmol, 76%) as brown crystalline materials. They were partly used for X-ray structure analysis.

Decomp.: 156 °C

Anal. Calc. for $C_{51}H_{18}D_4B_2F_{30}NO_3$: C, 47.40; H, 2.03; N, 1.08. Found: C, 46.86; H, 1.81; N, 1.05.

X-ray crystal structure analysis of 8a(4D) (erk9094): A orange plate-like specimen of $C_{51}H_{22}B_2F_{30}NO_3$, approximate dimensions 0.042 mm x 0.073 mm x 0.212 mm, was used for the X-ray crystallographic analysis. The X-ray intensity data were measured. A total of 1136 frames were collected. The total exposure time was 21.84 hours. The frames were integrated with the Bruker SAINT software package using a wide-frame algorithm. The integration of the data using a triclinic unit cell yielded a total of 37379 reflections to a maximum θ angle of 66.85° (0.84 Å resolution), of which 8746 were independent (average redundancy 4.274, completeness = 99.1%, R_{int} = 9.46%, R_{sig} = 6.92%) and 6137 (70.17%) were greater than 2σ(F^2). The final cell constants of a = 11.8064(3) Å, b = 14.9813(4) Å, c = 15.3306(4) Å, α = 91.992(2)°, β = 103.172(2)°, γ = 108.4960(10)°, volume = 2487.00(11) $Å^3$, are based upon the refinement of the XYZ-centroids of 9946 reflections above 20 σ(I) with 6.263° < 2θ < 133.6°. Data were corrected for absorption effects using the multi-scan method (SADABS). The ratio of minimum to maximum apparent transmission was 0.878. The calculated minimum and maximum transmission coefficients (based on crystal size) are 0.7190 and 0.9330. The structure was solved and refined using the Bruker SHELXTL Software Package, using the space group $P\bar{1}$, with Z = 2 for the formula unit, $C_{51}H_{22}B_2F_{30}NO_3$.The final anisotropic full-matrix least-squares refinement on F^2 with 788 variables converged at R1 = 5.87%, for the observed data and wR2 = 10.61% for all data. The goodness-of-fit was 1.077. The largest peak in the final difference electron density synthesis was 0.243 $e^-/Å^3$ and the largest hole was -0.287 $e^-/Å^3$ with an RMS deviation of 0.063 $e^-/Å^3$. On the basis of the final model, the calculated density was 1.720 g/cm^3 and F(000), 1278 e^-.

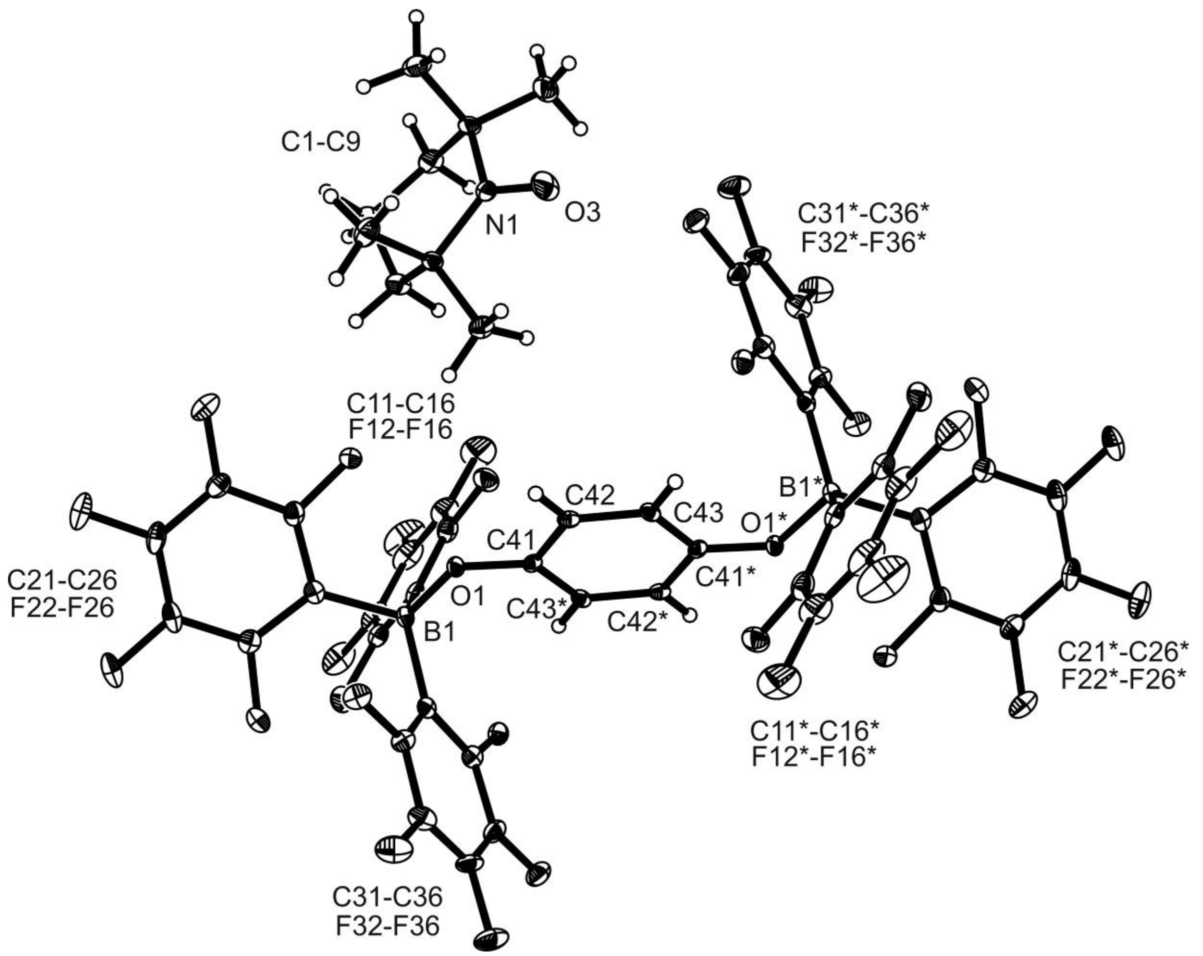

Figure S13. Crystal structure of compound **8a(4D)**. Only one half radical anion (completely generated over inversion centre) of two found in the asymmetric unit is shown. (Thermals ellipsoids are shown with 30% probability.)

C) Synthesis of compound **8b**

Experiment 1: (reaction of Gomberg dimer with *p*-benzoquinone (1:1), isolation of compound **10**)

Scheme S5

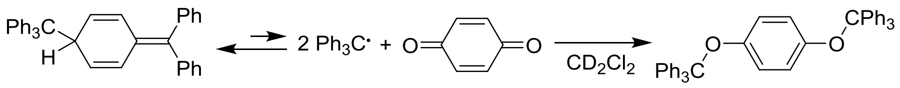

10

p-Benzoquinone (32.4 mg, 0.300 mmol) and Gomberg dimer (146 mg, 0.300 mmol) were dissolved in CD_2Cl_2 (1 mL) at room temperature. After stirring for 10 min., pentane (2 mL) was added to the resulting suspension. The solution was removed by filtration. The remaining white solid was washed with pentane and dried *in vacuo* giving compound **10** (110 mg, 0.186 mmol, 62%) as white powder.

^{1}H NMR (600 MHz, 299 K, CD_2Cl_2): δ ^{1}H: 7.34 (m, 6H, *o*-PhC), 7.23 (m, 9H, *m, p*-PhC), 6.25 (s, 2H, *o*-PhO).

^{13}C{^{1}H} NMR (151 MHz, 299 K, CD_2Cl_2): δ ^{13}C: 151.1 (*i*-PhO), 144.6 (*i*-PhC), 129.3 (*o*-PhC), 127.9 (*p*-PhC), 127.4 (*m*-PhC), 121.8 (*o*-PhO) , 90.8 (CO).

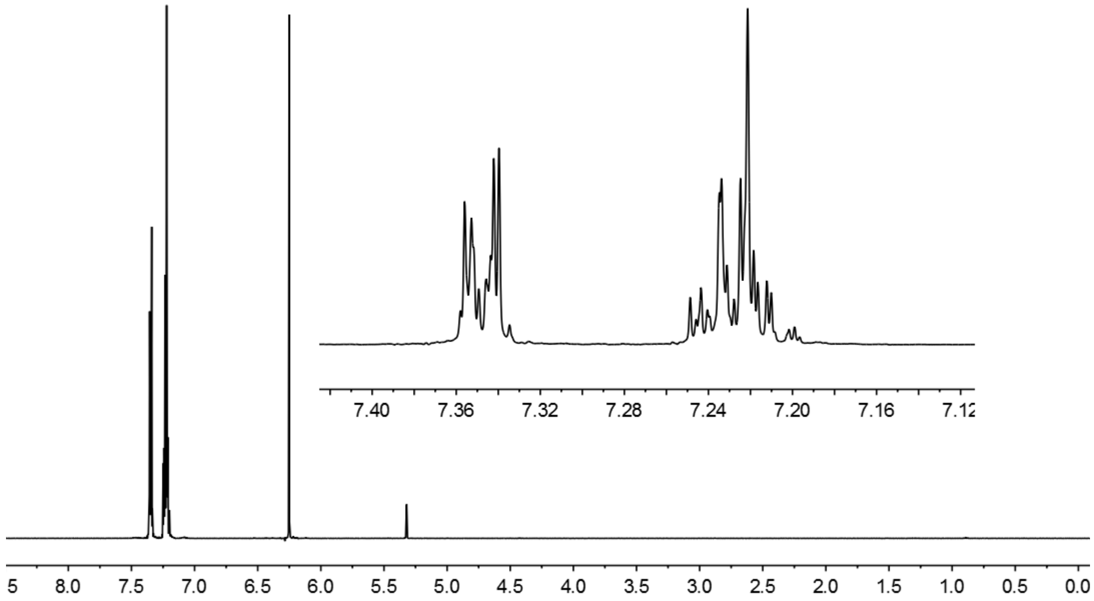

Figure S14. ^{1}H NMR (600 MHz, 299 K, CD_2Cl_2) spectrum of compound **10**.

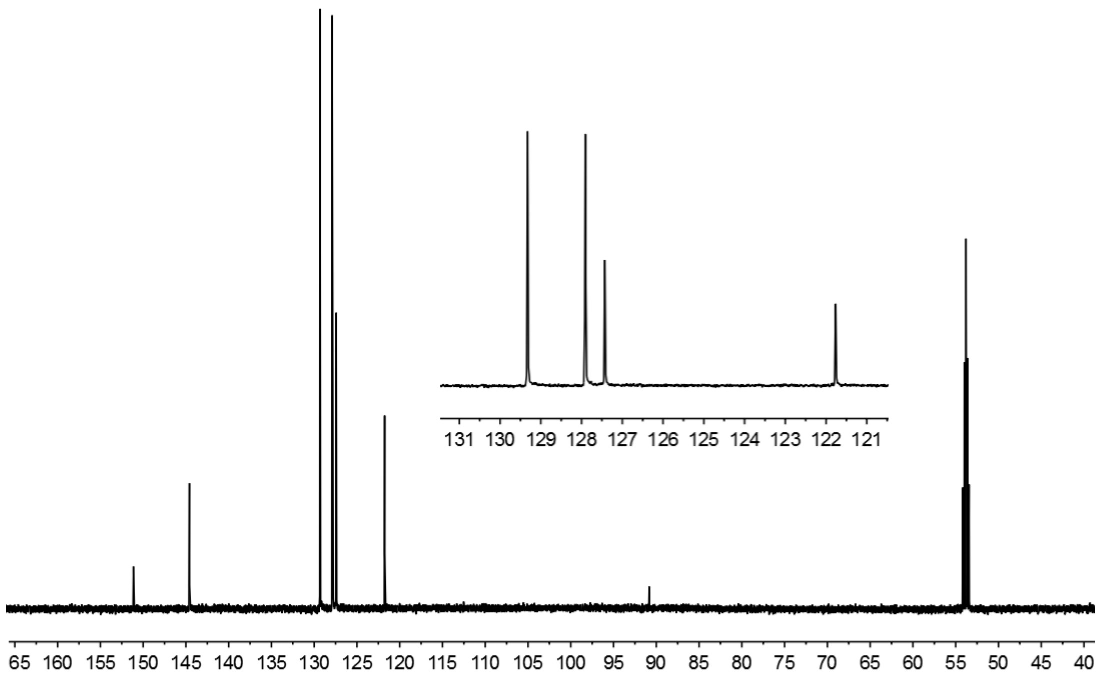

Figure S15. $^{13}C\{^1H\}$ NMR (151 MHz, 299 K, CD_2Cl_2) spetrum of compound **10**.

Experiment 2: [reaction of Gomberg dimer with *p*-benzoquinone (1:2)]

Scheme S6

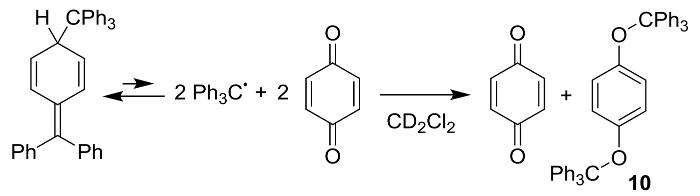

p-Benzoquinone (10.8 mg, 0.100 mmol) and Gomberg dimer (24.3 mg, 0.50 mmol) were dissolved in CD_2Cl_2 (0.5 mL) at room temperature. The resulting reaction mixture was characterized by NMR experiments.

[Comment: A mixture of *p*-benzoquinone (57 mol%, ^{1}H) and compound **10** (43 mol%, ^{1}H) was observed]

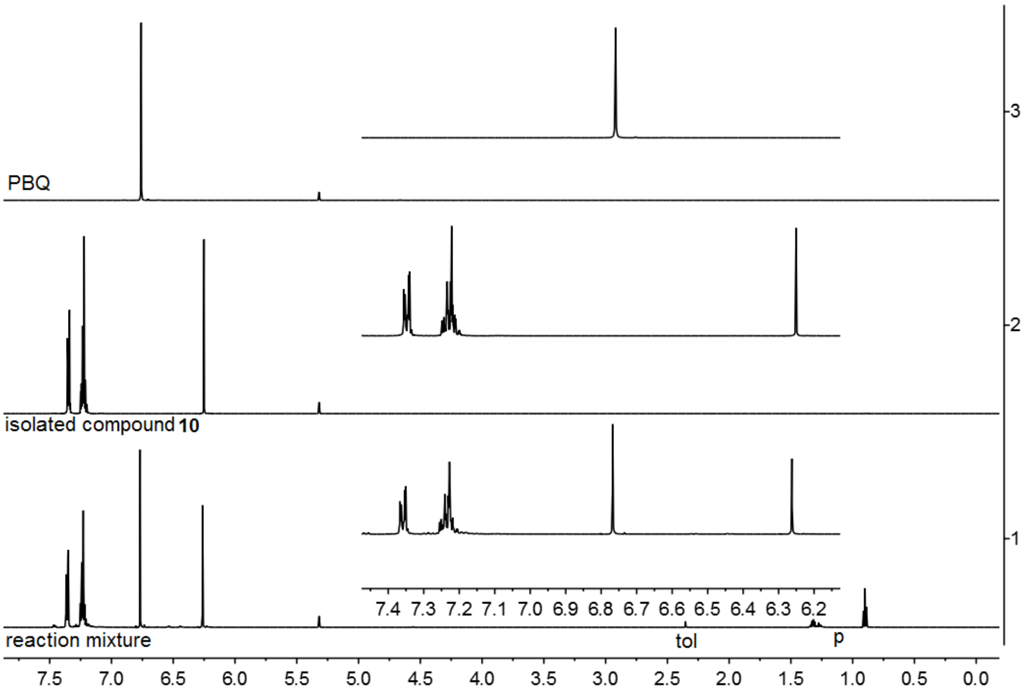

Figure S16. ^{1}H NMR (600 MHz, 299 K, CD_2Cl_2) spectra of (1) reaction mixture, (2) isolated compound **10**, (3) *p*-benzoquinone. [PBQ: *p*-benzoquinone, p: pentane, tol: toluene]

Experiment 3: (reaction of *p*-benzoquinone / 2 $B(C_6F_5)_3$ adduct with Gomberg dimer , isolation of compound **8b**)

Scheme S7

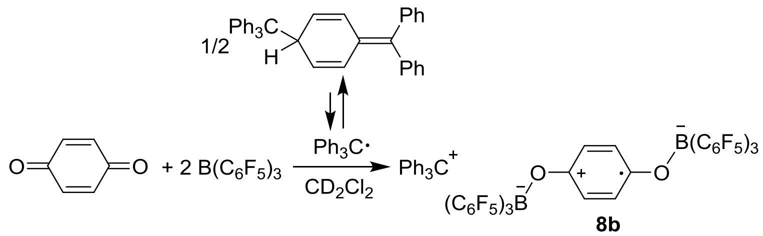

p-Benzoquinone (21.6 mg, 0.200 mmol) and $B(C_6F_5)_3$ (204 mg, 0.400 mmol) were dissolved in CD_2Cl_2 (1.0 mL). Gomberg dimer (48.6 mg, 0.100 mmol) was added to the resulting mixture (red solution). Subsequently, the resulting reaction mixture was characterized by NMR experiments. The resulting (dark red) reaction solution was then layered by pentane (0.5 mL) and stored at –35 °C for one week. The dark red crystals were observed. The solution was removed by decantation and part of the obtained crystalline material was used for the X-ray crystal structure analysis. Drying of the remaining crystals in vacuo gave compound **8b** (178 mg, 0.130 mmol, 65%) as dark red crystalline material.

Decomp.:169 °C

Anal. Calc. for $C_{61}H_{19}B_2F_{30}O_2$: C, 53.27; H, 1.39. Found: C, 53.70; H, 1.87.

NMR data of isolated compound **8b**:

^{1}H NMR (600 MHz, 299 K, CD_2Cl_2): δ ^{1}H: 8.29 (br m, 1H, *p*-Ph), 7.90 (br m, 2H, *m*-Ph), 7.68 (br m, 2H, *o*-Ph).

^{1}H NMR (600 MHz, 253 K, CD_2Cl_2): δ ^{1}H: 8.27 (br, 1H, *p*-Ph), 7.88 (br, 2H, *m*-Ph), 7.66 (br, 2H, *o*-Ph).

^{13}C{^{1}H} NMR (600 MHz, 253 K, CD_2Cl_2): δ ^{13}C: 210.3 (C^+), 143.6 (*p*-Ph), 142.9 (*o*-Ph), 139.7 (*i*-Ph), 130.7 (*m*-Ph).

^{19}F NMR (564 MHz, 299 K, CD_2Cl_2): δ ^{19}F: see Figure S19.

^{11}B{^{1}H} NMR (192 MHz, 299 K, CD_2Cl_2): δ ^{11}B: see Figure S20.

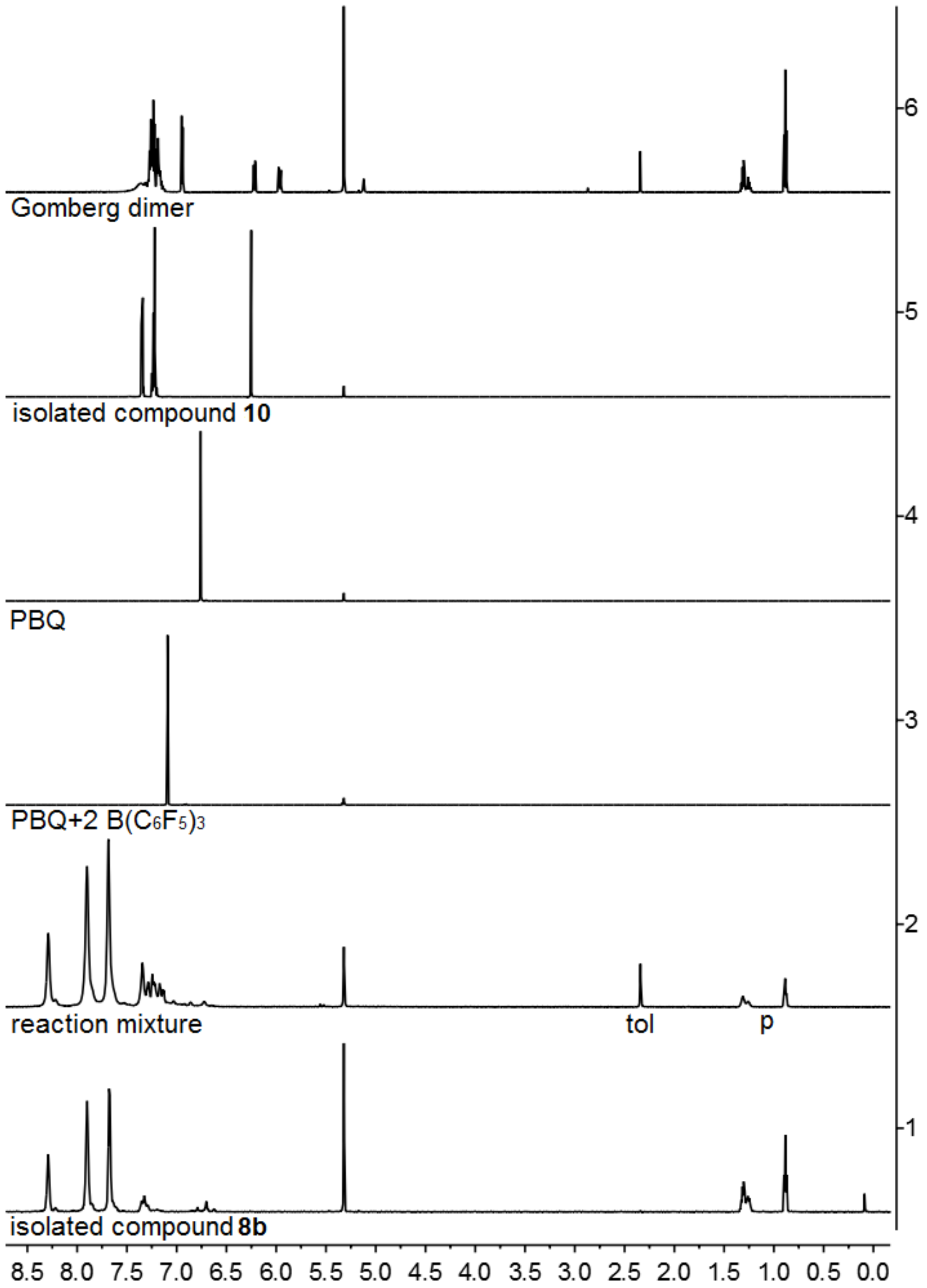

Figure S17. ^{1}H NMR (600 MHz, 299 K, CD_2Cl_2) spectra of (1) isolated compound **8b**, (2) reaction mixture of experiment 3, (3) reaction mixture of *p*-benzoquinone with 2 equiv. of $B(C_6F_5)_3$, (4) *p*-benzoquinone, (5) isolated compound **10** and (6) Gomberg dimer. [PBQ: *p*-benzoquinone, p: pentane, tol: toluene]

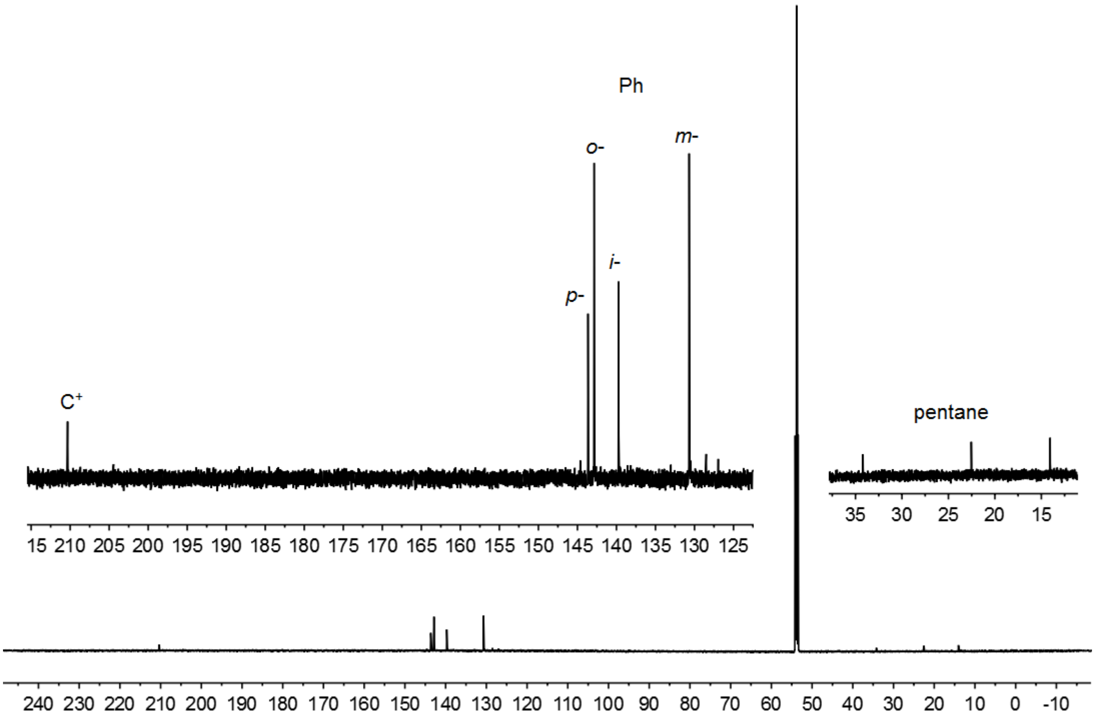

Figure S18. $^{13}C\{^{1}H\}$ NMR (151 MHz, 253 K, CD_2Cl_2) spectrum of isolated compound **8b**.

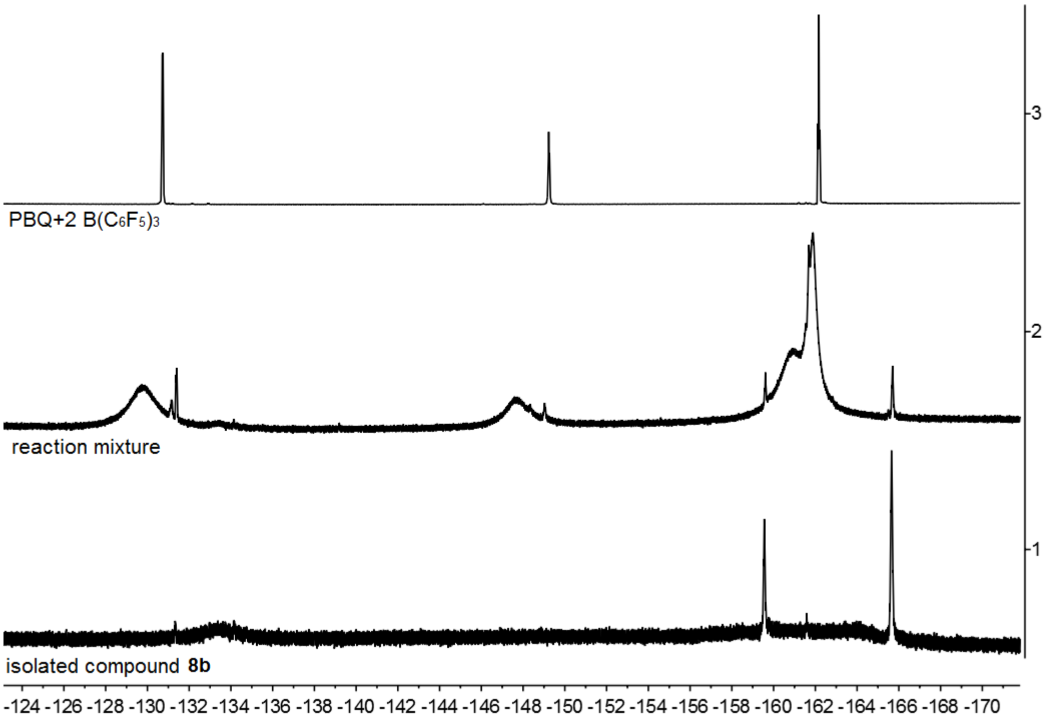

Figure S19. ^{19}F NMR (564 MHz, 299 K, CD_2Cl_2) spectra of (1) isolated compound **8b**, (2) reaction mixture of experiment 3 and (3) reaction mixture of *p*-benzoquinone with 2 equiv. of $B(C_6F_5)_3$.

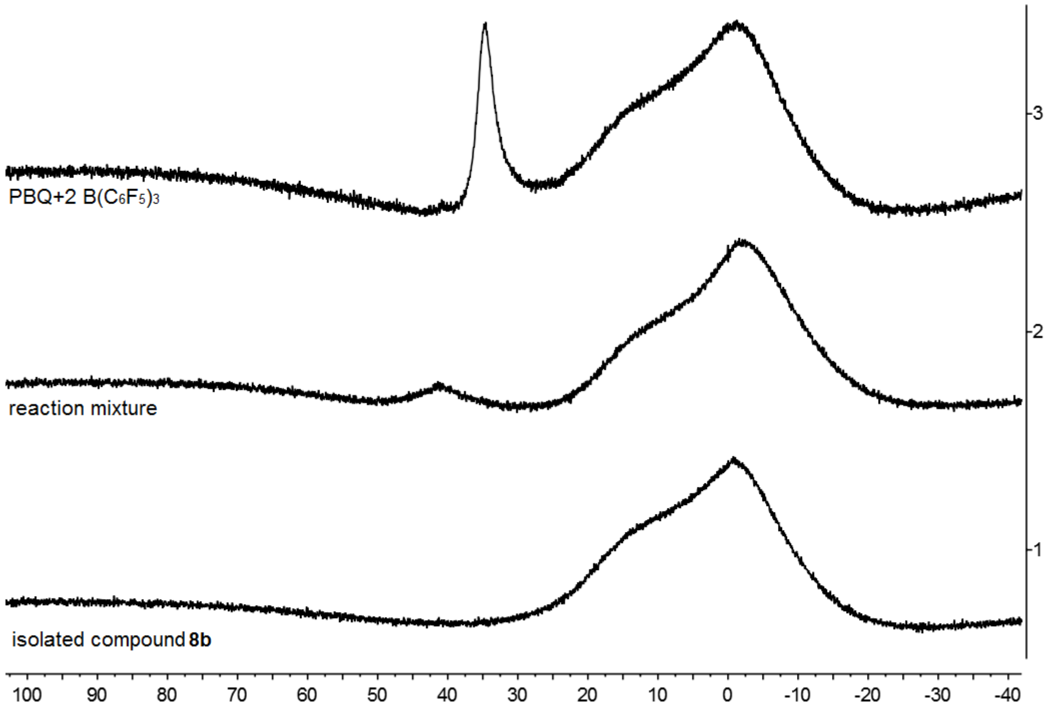

Figure S20. $^{11}B\{^{1}H\}$ NMR (192 MHz, 299 K, CD_2Cl_2) spectra of (1) isolated compound **8b**, (2) reaction mixture of experiment 3 and (3) reaction mixture of *p*-benzoquinone with 2 equiv. of $B(C_6F_5)_3$.

X-ray crystal structure analysis of 8b (erk8901): A orange prism-like specimen of $C_{61}H_{19}B_2F_{30}O_2$, approximate dimensions 0.075 mm x 0.122 mm x 0.124 mm, was used for the X-ray crystallographic analysis. The X-ray intensity data were measured. A total of 505 frames were collected. The total exposure time was 6.42 hours. The frames were integrated with the Bruker SAINT software package using a narrow-frame algorithm. The integration of the data using a triclinic unit cell yielded a total of 46223 reflections to a maximum θ angle of 25.03° (0.84 Å resolution), of which 10896 were independent (average redundancy 4.242, completeness = 99.8%, R_{int} = 16.28%, R_{sig} = 12.02%) and 6131 (56.27%) were greater than 2σ(F^2). The final cell constants of a = 13.7910(12) Å, b = 14.0959(13) Å, c = 18.1102(14) Å, α = 109.569(2)°, β = 109.658(2)°, γ = 90.652(3)°, volume = 3093.2(5) $Å^3$, are based upon the refinement of the XYZ-centroids of 5911 reflections above 20 σ(I) with 4.729° < 2θ < 50.66°. Data were corrected for absorption effects using the multi-scan method (SADABS). The ratio of minimum to maximum apparent transmission was 0.930. The calculated minimum and maximum transmission coefficients (based on crystal size) are 0.9820 and 0.9890. The structure was solved and refined using the Bruker SHELXTL Software Package, using the space group $P\bar{1}$, with Z = 2 for the formula unit, $C_{61}H_{19}B_2F_{30}O_2$. The final anisotropic full-matrix least-squares refinement on F^2 with 856 variables converged at R1 = 6.01%, for the observed data and wR2 = 13.07% for all data. The goodness-of-fit was 1.007. The largest peak in the final difference electron density synthesis was 0.291 $e^-/Å^3$ and the largest hole was -0.321 $e^-/Å^3$ with an RMS deviation of 0.067 $e^-/Å^3$. On the basis of the final model, the calculated density was 1.477 g/cm^3 and F(000), 1362 e^-.

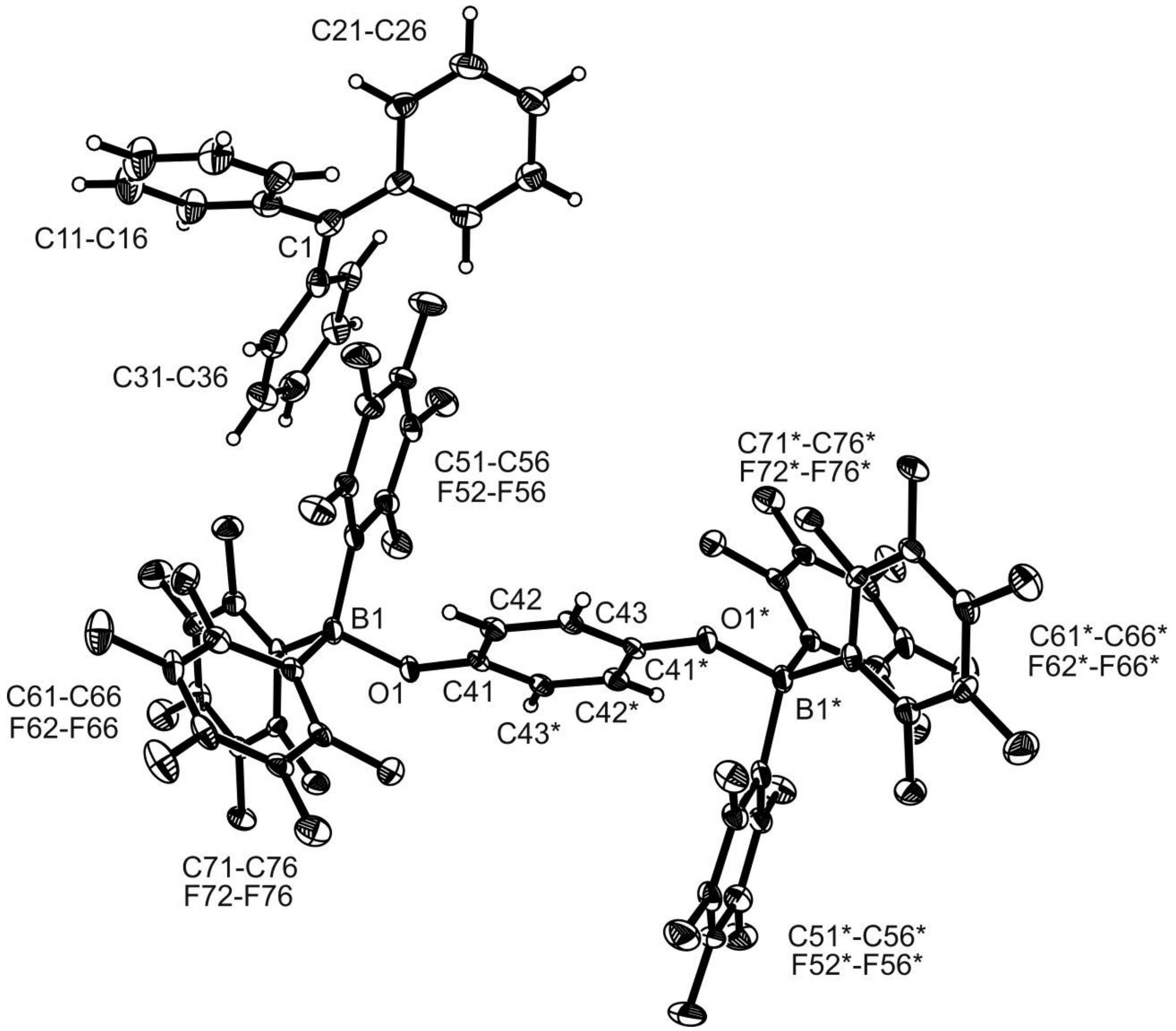

Figure S21. Crystal structure of compound **8b**. Only one half radical anion (completely generated over inversion centre) of two found in the asymmetric unit is shown. (Thermals ellipsoids are shown with 50% probability.)

D) Synthesis of compound **8c** (**8c-D**)

Experiment 1: [reaction of Cp*$_2$Fe with *p*-benzoquinone (1:1)]

Scheme S8

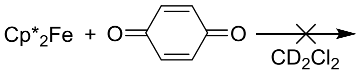

p-Benzoquinone (5.4 mg, 0.050 mmol) and decamethylferrocene (16.3 mg, 0.050 mmol) were dissolved in CD_2Cl_2 (0.5 mL) at room temperature and characterized by NMR experiments.

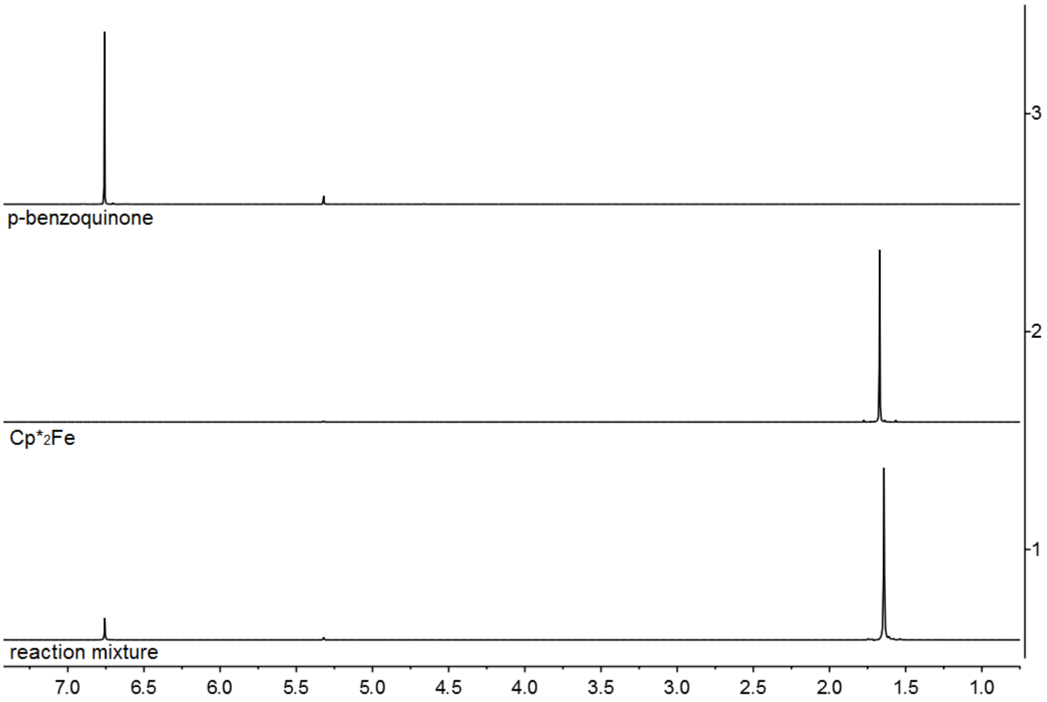

Figure S22. ^{1}H NMR (600 MHz, 299 K, CD_2Cl_2) spectra of (1) reaction mixture, (2) decamethylferrocene and (3) *p*-benzoquinone.

Experiment 2: (reaction of *p*-benzoquinone / 2 $B(C_6F_5)_3$ adduct with decamethylferrocene, isolation of compound **8c**)

Scheme S9

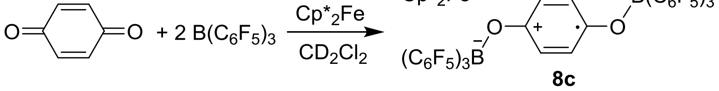

p-Benzoquinone (10.8 mg, 0.100 mmol) and $B(C_6F_5)_3$ (102 mg, 0.200 mmol) were dissolved in CD_2Cl_2 (1.0 mL). Decamethylferrocene (32.6 mg, 0.100 mmol) was added to the resulting mixture (red solution) giving a dark brow solution. After 1 min, dark brown crystals precipitated from the reaction mixture. The crystals was separated by decantation and part of the crystals were used for X-ray structure analysis. The remaining crystals washed with pentane and dried in vacuo giving compound **8c** (92 mg, 0.063 mmol, 63%) as dark red crystalline material.

Decomp.:244 °C

Anal. Calc. for $C_{62}H_{34}B_2F_{30}O_2Fe$: C, 51.06; H, 2.35. Found: C, 50.82; H, 2.48.

NMR data of isolated compound **8c**: (the solubility of **8c** in CD_2Cl_2 is very low)

^{1}H NMR (600 MHz, 299 K, CD_2Cl_2): δ ^{1}H: –36.7 (br s, 30H, $Cp^*{}_2Fe^+$).

^{19}F NMR (564 MHz, 299 K, CD_2Cl_2): δ ^{19}F: see Figure S25.

^{11}B{^{1}H} NMR (192 MHz, 299 K, CD_2Cl_2): δ ^{11}B: see Figure S24.

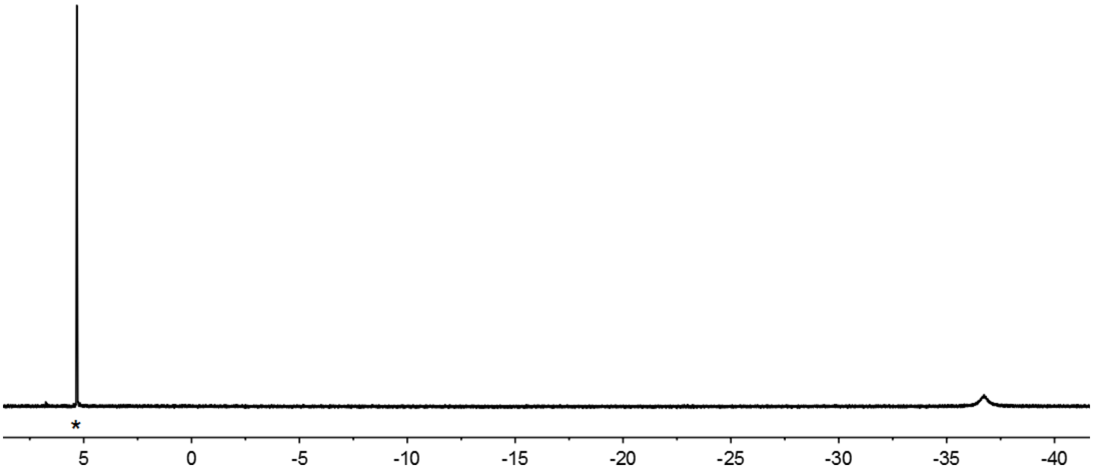

Figure S23. ^{1}H NMR (600 MHz, 299 K, CD_2Cl_2) of isolated compound **8c**.

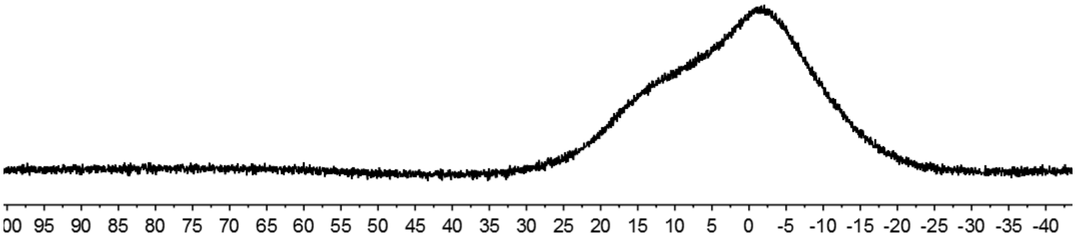

Figure S24. ^{11}B{^{1}H} NMR (192 MHz, 299 K, CD_2Cl_2): of isolated compound **8c**.

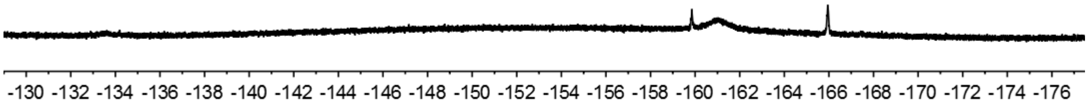

Figure S25. ^{19}F NMR (564 MHz, 299 K, CD_2Cl_2) of isolated compound **8c.**

X-ray crystal structure analysis of 8c (erk8961): formula $C_{62}H_{34}B_2F_{30}FeO_2$, M = 1458.36, green-orange crystal, 0.15 x 0.13 x 0.03 mm, a = 9.5809(3), b = 10.9166(4), c = 14.2783(4) Å, α = 74.722(2), β = 79.532(2), γ = 85.172(2)°, V = 1415.6(1) Å^3, ρ_{calc} = 1.711 gcm^{-3}, μ = 0.413 mm^{-1}, empirical absorption correction (0.940 ≤ T ≤ 0.987), Z = 1, triclinic, space group $P\bar{1}$ (No. 2), λ = 0.71073 Å, T = 173(2) K, ω and ϕ scans, 12519 reflections collected (±h, ±k, ±l), 4845 independent (R_{int} = 0.045) and 4066 observed reflections [I>2σ(I)], 444 refined parameters, R = 0.053, wR^2 = 0.127, max. (min.)

residual electron density 0.36 (-0.35) e.Å^{-3}, the position of the hydrogen were calculated and refined as riding atoms.

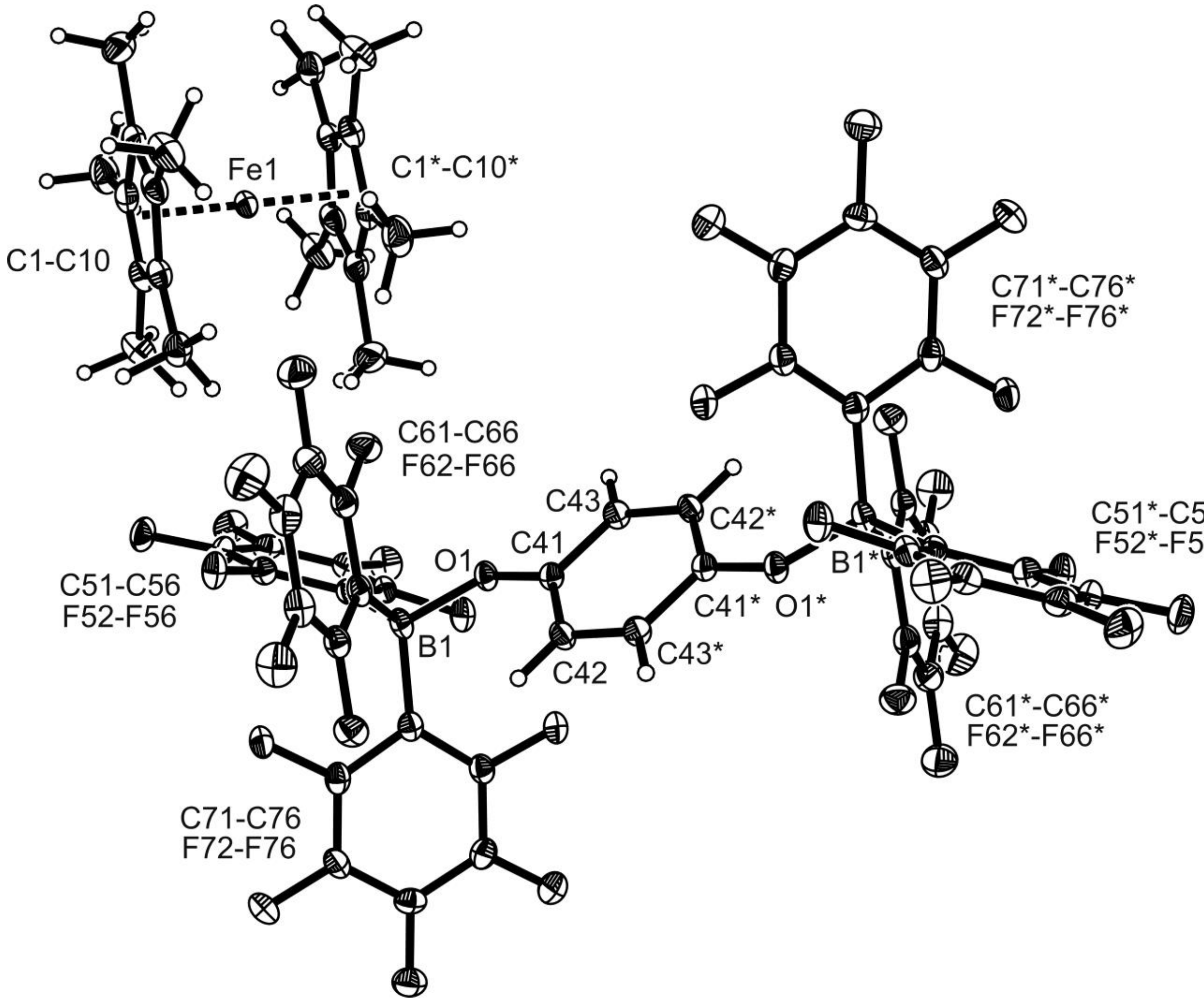

Figure S26. Crystal structure of compound **8c**. The cation and the radical anion are generated over inversion centre. (Thermals ellipsoids are shown with 30% probability.)

Experiment 2: (reaction of *p*-benzoquinone-d$_4$ / 2 $B(C_6F_5)_3$ adduct with decamethylferrocene, isolation of compound **8c-D**)

Scheme S10

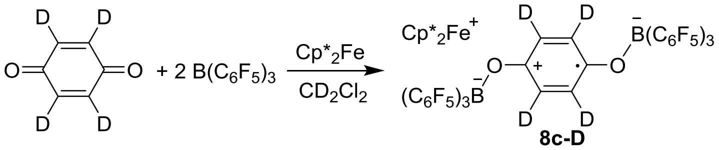

8c-D

p-Benzoquinone (5.6 mg, 0.050 mmol) and $B(C_6F_5)_3$ (102 mg, 0.10 mmol) were dissolved in CD_2Cl_2 (1.0 mL). Decamethylferrocene (32.6 mg, 0.100 mmol) was added to the resulting mixture (red solution) giving a dark brow solution. After 1 min, dark brown crystals precipitated from the reaction mixture. The crystals was separated by decantation and part of the crystals were used for X-ray structure analysis. The remaining crystals washed with pentane and dried in vacuo giving compound **8c-D** (60 mg, 0.041 mmol, 82%) as dark red crystalline material.

[The isolated crystalline material was analyzed by X-ray diffraction, which showed the same cell parameters as compound **8c**]

Decomp.:244 ^{o}C

Anal. Calc. for $C_{62}H_{30}D_4B_2F_{30}O_2Fe$: C, 50.92; H, 2.62. Found: C, 50.05; H, 2.37.

E) Reaction of 9,10-anthraquinone, 2 $B(C_6F_5)_3$ and TEMPO

Scheme S11

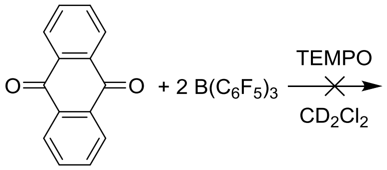

9,10-Anthraquinone (20.8 mg, 0.100 mmol) and $B(C_6F_5)_3$ (102 mg, 0.200 mmol) were mixed in CD_2Cl_2 (1.0 mL). Subsequently, TEMPO (15.6 mg, 0.100 mmol) was added. The resulting yellow solution was characterized by NMR experiments.

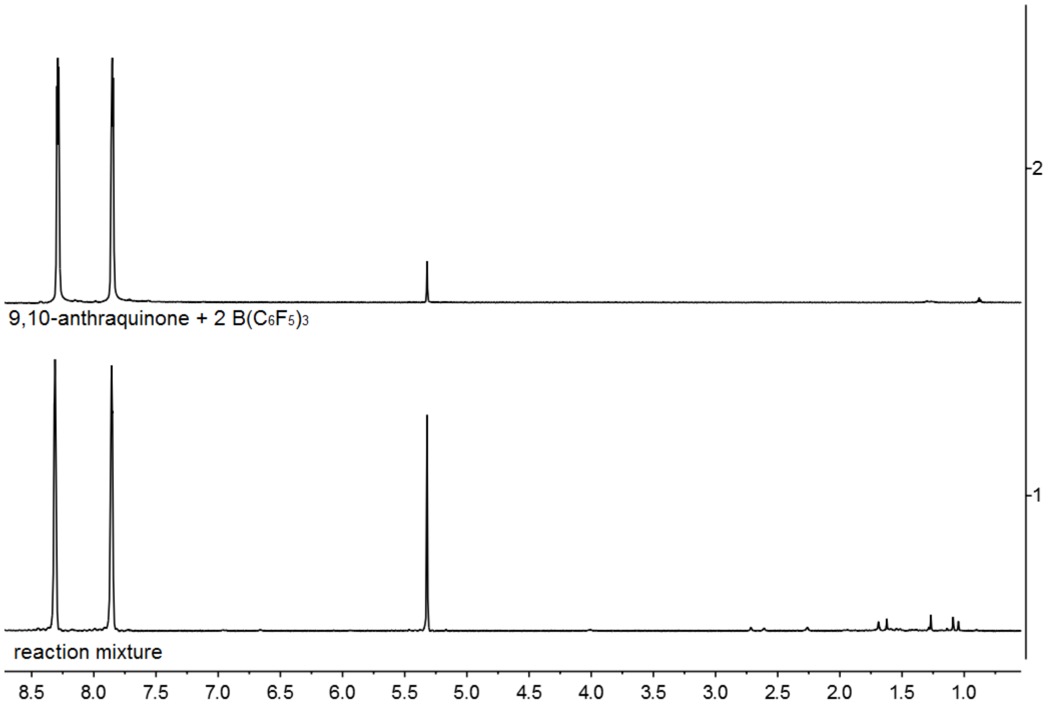

Figure S27. ^1H NMR (600 MHz, 299 K, CD_2Cl_2) spectra of (1) reaction mixture and (2) the mixture of 9,10-anthraquinone with 2 equiv. of $B(C_6F_5)_3$.

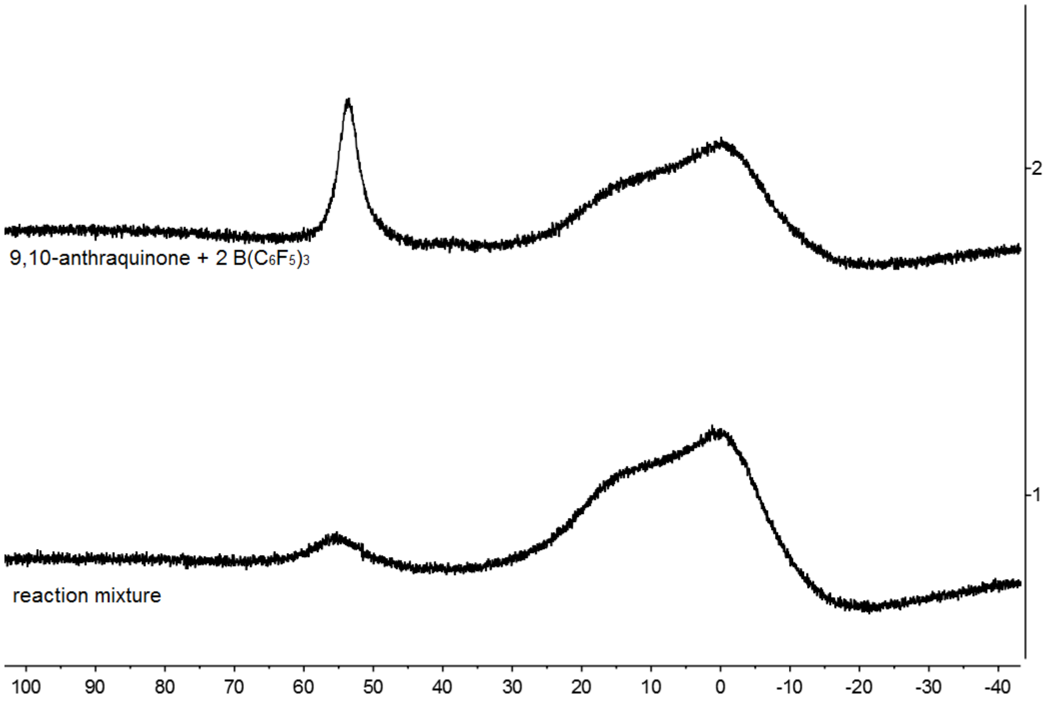

Figure S28. $^{11}B\{^{1}H\}$ NMR (192 MHz, 299 K, CD_2Cl_2) spectra of (1) reaction mixture and (2) the mixture of 9,10-anthraquinone with 2 equiv. of $B(C_6F_5)_3$.

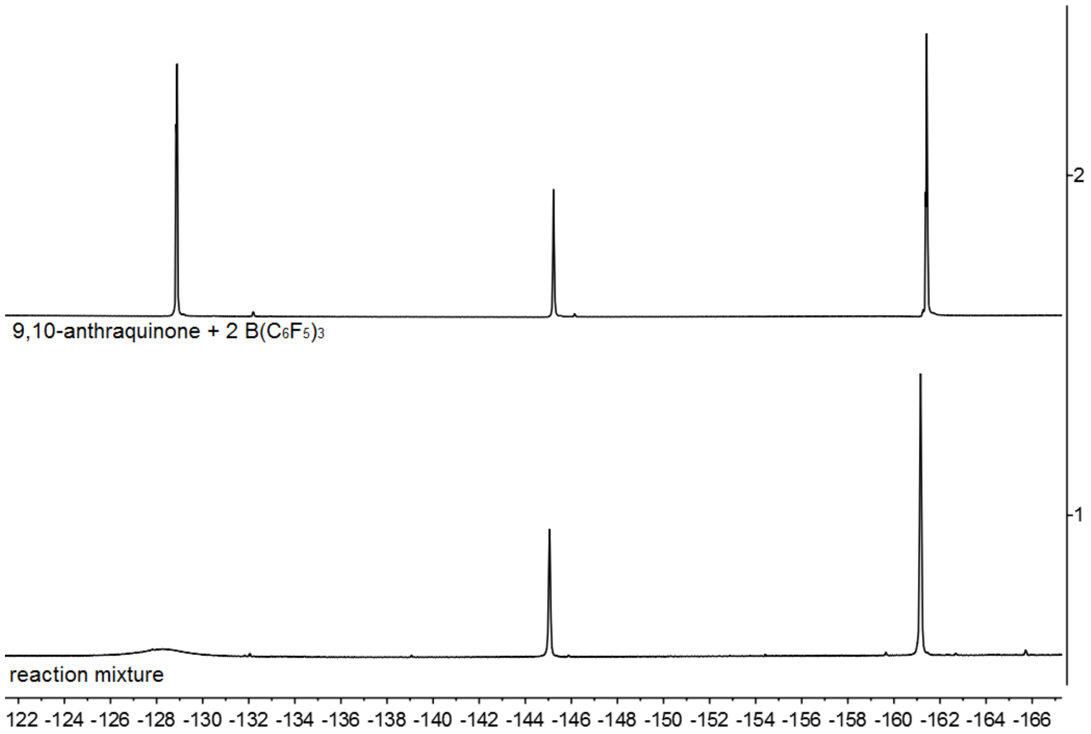

Figure S29. ^{19}F NMR (564 MHz, 299 K, CD_2Cl_2) spectra of (1) reaction mixture and (2) the mixture of 9,10-anthraquinone with 2 equiv. of $B(C_6F_5)_3$.

F) Generation of compound **11b**

Experiment 1: [reaction of Gomberg dimer with 9,10-anthraquinone (1:2)]

Scheme S12

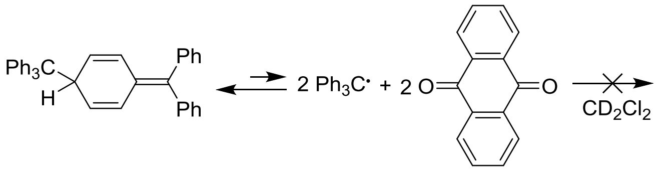

9,10-anthraquinone (20.8 mg, 0.10 mmol) and Gomberg dimer (24.3 mg, 0.050 mmol) were mixed in CD_2Cl_2 (1 mL) at room temperature. After stirring for 1 hour, the resulting reaction mixture (white suspension) was characterized by NMR experiments.

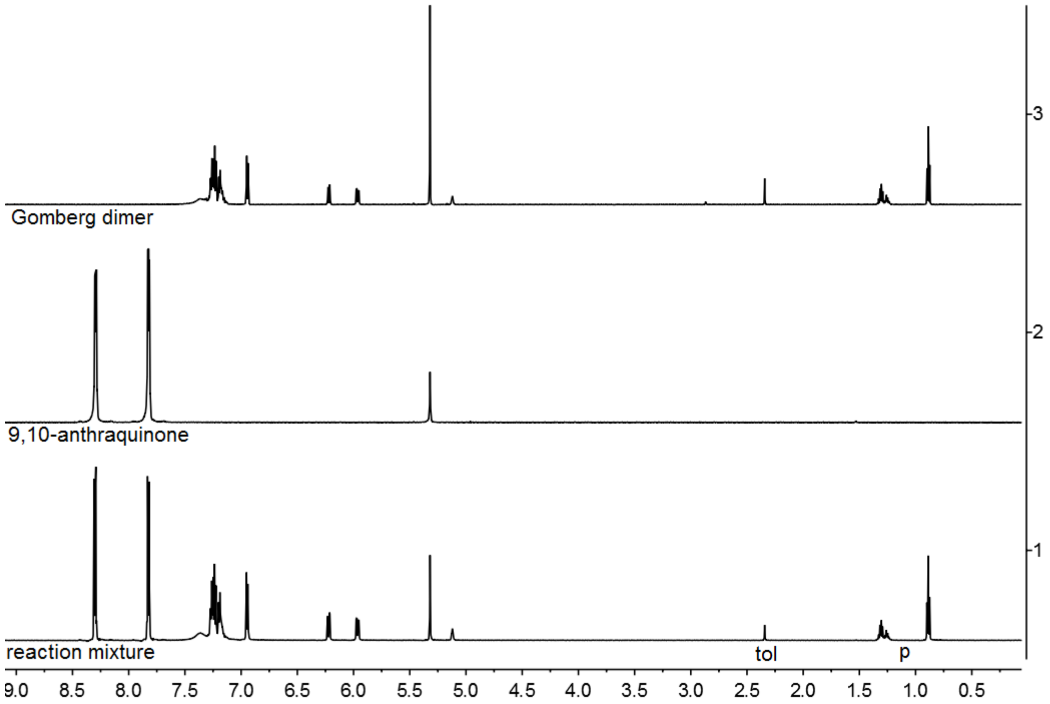

Figure S30. ^1H NMR (600 MHz, 299 K, CD_2Cl_2) spectra of (1) reaction mixture, (2) 9,10-anthraquinone, (3) Gomberg dimer. [p: pentane, tol: toluene]

.

Experiment 2: (reaction of 9,10-anthraquinone with 2 $B(C_6F_5)_3$ and Gomberg dimer)

Scheme S13

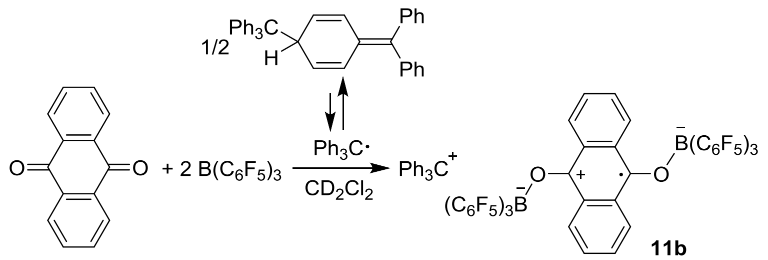

9,10-Anthraquinone (20.8 mg, 0.100 mmol) and $B(C_6F_5)_3$ (102 mg, 0.200 mmol) were mixed in CD_2Cl_2 (1.0 mL). The resulting a yellow solution was characterized by NMR experiments. Subsequently, Gomberg dimer (24.3 mg, 0.050 mmol) was added. The resulting dark green reaction mixture was characterized by NMR experiments again. After diffusion of pentane to the resulting reaction solution and at –35 °C for 48h, the dark green solid precipitated. Removal of the supernatant solution was done by decantation, and part of the obtained crystalline material was used for the X-ray crystal structure analysis (under microscope the crystals with different colors were observed, the dark green crystals were picked for measurement). Drying of the remaining crystals in vacuo gave a mixture (78 mg) of dark green crystalline material mixed with some unknown white crystalline material.

X-ray crystal structure analysis of 11b (erk8933): A dark orange prism-like specimen of $C_{69}H_{23}B_2F_{30}O_2 \cdot 3 \times CH_2Cl_2$, approximate dimensions 0.120 mm x 0.200 mm x 0.250 mm, was used for the X-ray crystallographic analysis. The X-ray intensity data were measured. A total of 1514 frames were collected. The total exposure time was 24.26 hours. The frames were integrated with the Bruker SAINT software package using a wide-frame algorithm. The integration of the data using a triclinic unit cell yielded a total of 52928 reflections to a maximum θ angle of 66.73° (0.84 Å resolution), of which 12023 were independent (average redundancy 4.402, completeness = 99.3%, R_{int} = 5.21%, R_{sig} = 3.98%) and 9693 (80.62%) were greater than $2\sigma(F^2)$. The final cell constants of a = 12.3256(7) Å, b = 16.7558(9) Å, c = 17.1303(9) Å, α = 90.820(3)°, β = 97.535(3)°, γ = 103.218(3)°, volume = 3410.8(3) $Å^3$, are based upon the refinement of the XYZ-centroids of 9938 reflections above 20 σ(I) with 7.347° < 2θ < 133.0°. Data were corrected for absorption effects using the multi-scan method (SADABS). The ratio of minimum to maximum apparent transmission was 0.823. The calculated minimum and maximum transmission coefficients (based on crystal size) are 0.4760 and 0.6790. The structure was solved and refined using the Bruker SHELXTL Software Package, using the space group $P\bar{1}$, with Z = 2 for the formula unit, $C_{69}H_{23}B_2F_{30}O_2 \cdot 3 \times CH_2Cl_2$. The final anisotropic full-matrix least-squares refinement on F^2 with 1056 variables converged at R1 = 3.72%, for the observed data and wR2 = 9.43% for all data. The goodness-of-fit was 1.052. The largest peak in the final difference electron density synthesis was 0.274 $e^-/Å^3$ and the largest hole was -0.435 $e^-/Å^3$ with an RMS deviation of 0.053 $e^-/Å^3$. On the basis of the final model, the calculated density was 1.685

g/cm^3 and F(000), 1718 e$^-$.

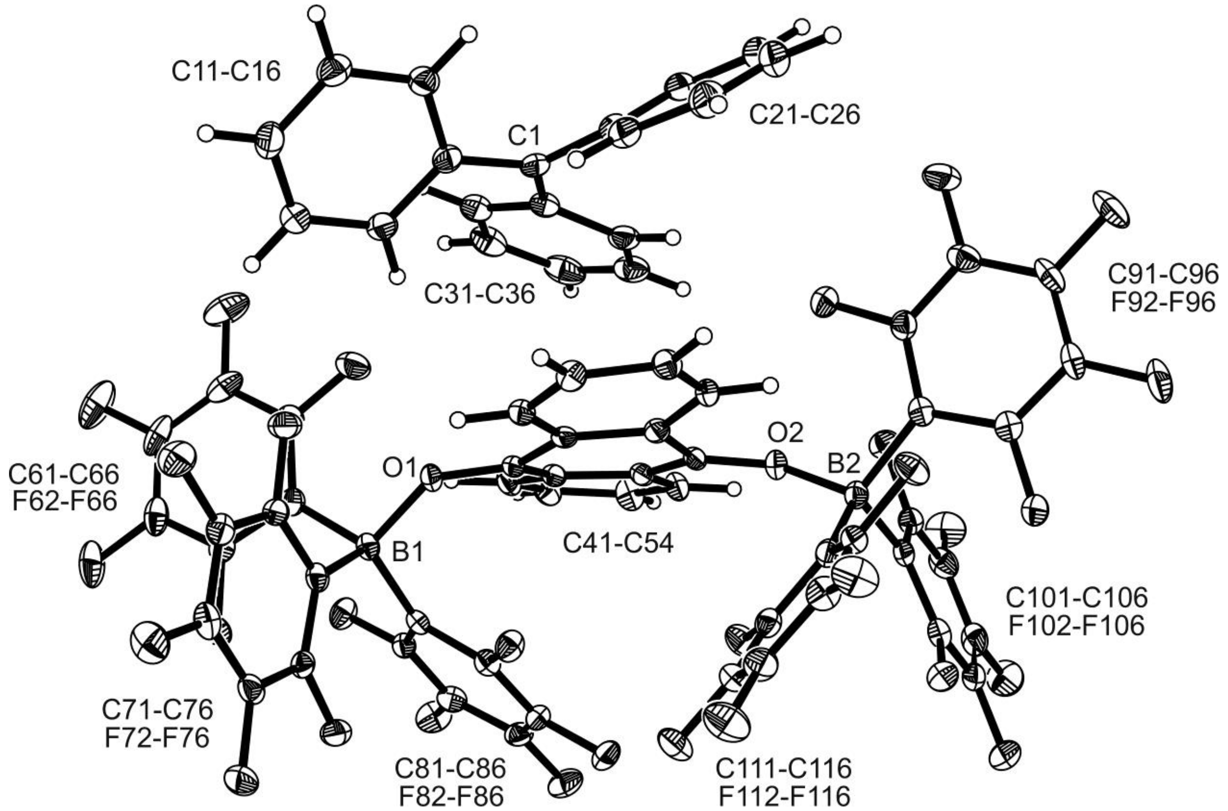

Figure S31. Crystal structure of compound **11b**. (Thermals ellipsoids are shown with 50% probability.)

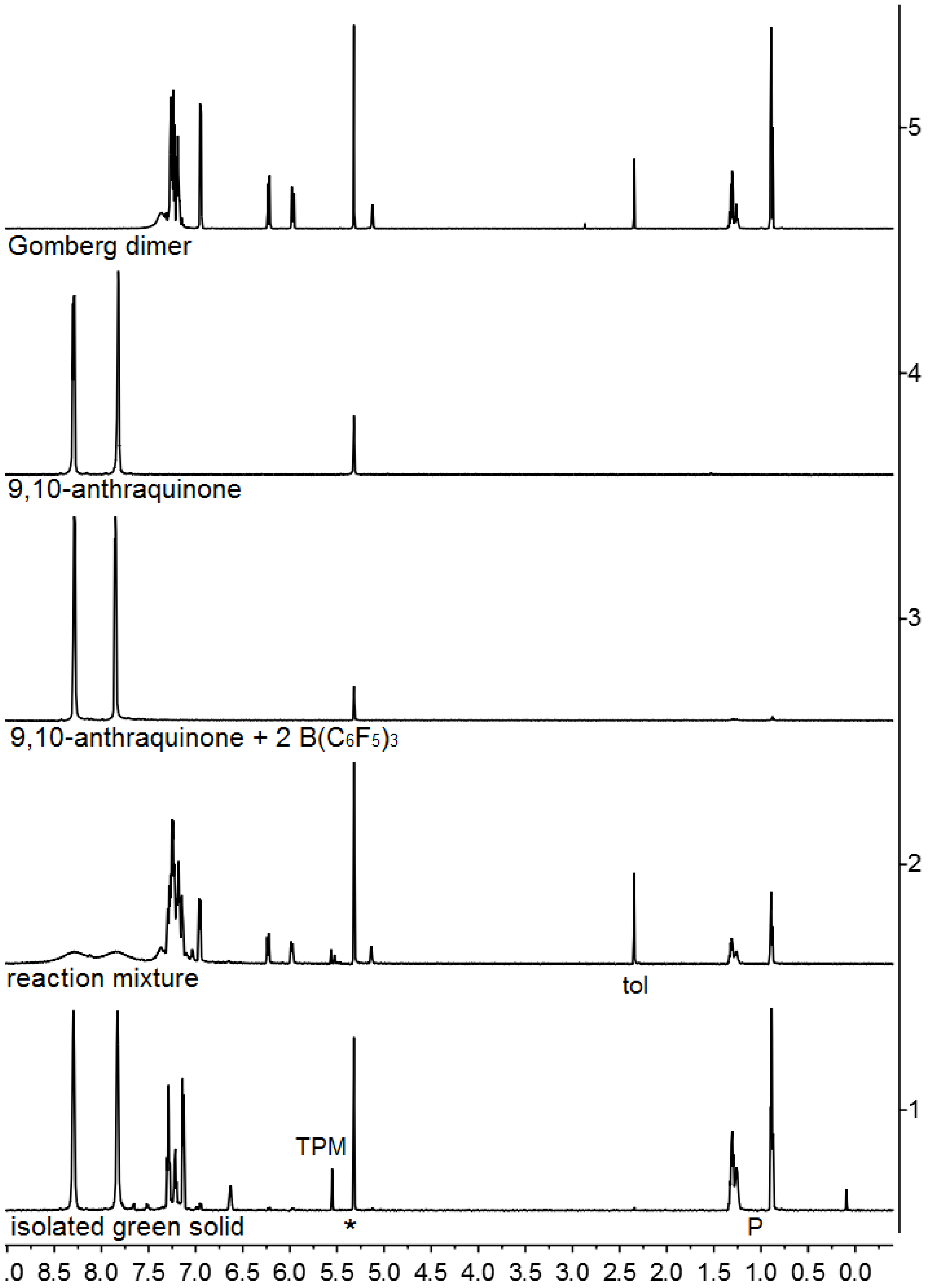

Figure S32. ^{1}H NMR (600 MHz, 299 K, CD_2Cl_2) spectra of (1) isolated green solid, (2) reaction mixture, (3) the mixture of 9,10-anthraquinone with 2 equiv. of $B(C_6F_5)_3$, (4) 9,10-anthraquinone, (5) Gomberg dimer.

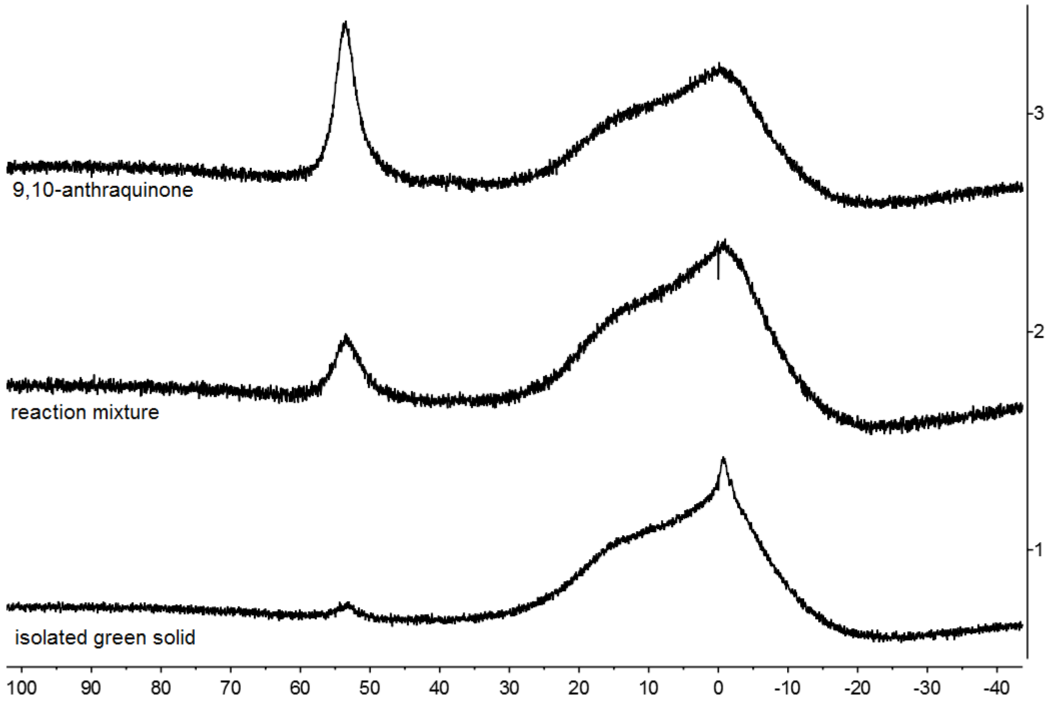

Figure S33. $^{11}B\{^{1}H\}$ NMR (192 MHz, 299 K, CD_2Cl_2) spectra of (1) isolated green solid, (2) reaction mixture, (3) the mixture of 9,10-anthraquinone with 2 equiv. of $B(C_6F_5)_3$.

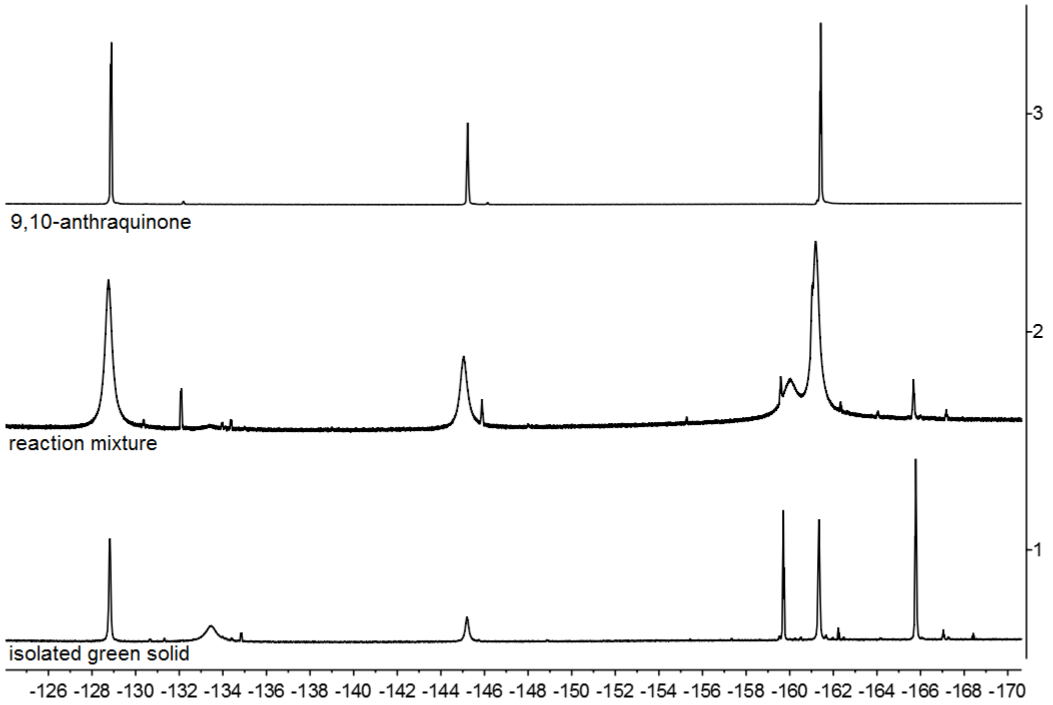

Figure S34. ^{19}F NMR (564 MHz, 299 K, CD_2Cl_2) spectra of (1) isolated green solid, (2) reaction mixture, (3) the mixture of 9,10-anthraquinone with 2 equiv. of $B(C_6F_5)_3$

G) Synthesis of compound **11c**

Experiment 1: [reaction of decamethylferrocene with 9,10-anthraquinone (1:1)]

Scheme S14

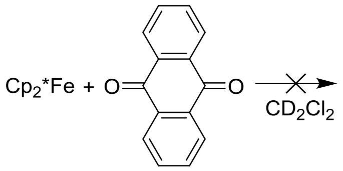

9,10-Anthraquinone (10.4 mg, 0.050 mmol) and decamethyferrocene (16.3 mg, 0.050 mmol) were mixed in CD_2Cl_2 (1 mL) at room temperature. After 1 hour, the resulting reaction mixture was characterized by NMR experiments.

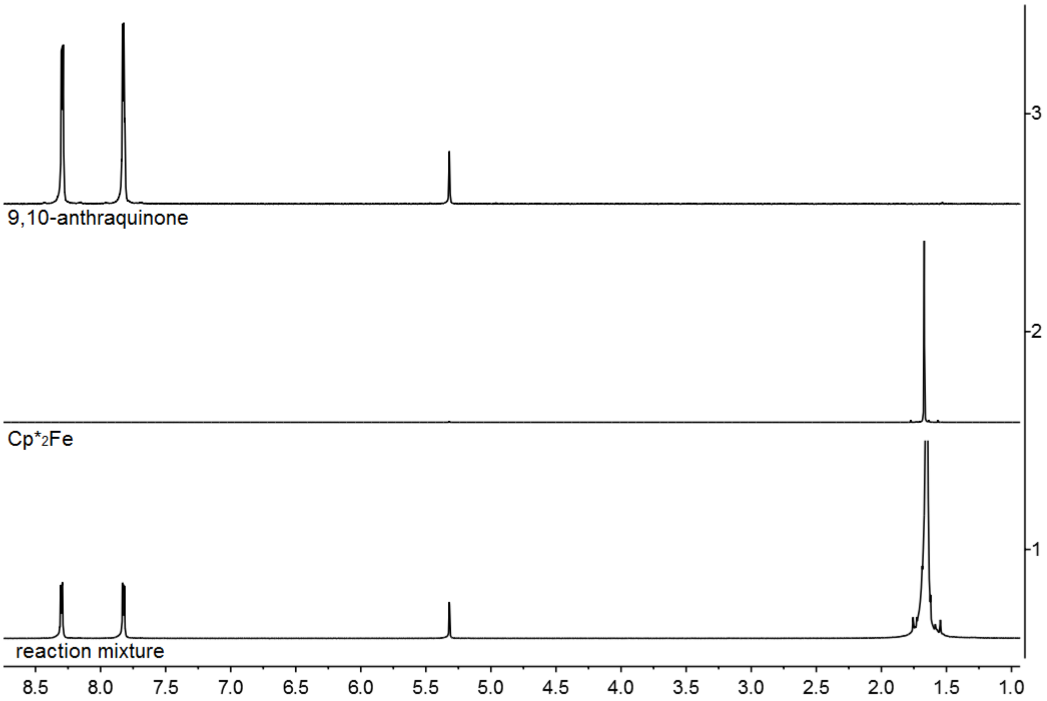

Figure S35. ^1H NMR (600 MHz, 299 K, CD_2Cl_2) spectra of (1) reaction mixture, (2) decamethylferrocene and (3) 9,10-anthraquinone.

.

Experiment 2: [reaction of 9,10-anthraquinone with 2 $B(C_6F_5)_3$ and Cp^*_2Fe, isolation of compound **11c**]

Scheme S15

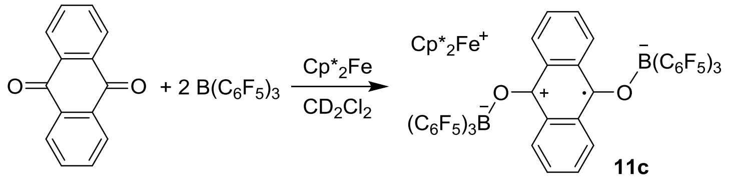

9,10-Anthraquinone (20.8 mg, 0.100 mmol) and $B(C_6F_5)_3$ (102 mg, 0.200 mmol) were mixed in CD_2Cl_2 (1.0 mL) resulting a yellow a solution. Subsequently, decamethylferrocene (32.6 mg, 0.100 mmol) was added. The resulting dark green reaction mixture was characterized by NMR experiments. After two-layer diffusion of pentane to the resulting reaction solution at –35 °C for 48h, the dark green solid precipitated. Removal of the solution was done by decantation, and part of the obtained crystalline material was used for the X-ray crystal structure analysis. Drying of the remaining crystals in vacuo gave compound **11c** (137 mg, 0.088, 88%) as dark green crystalline material.

Melting Point.: 205 °C

Anal. Calc. for $C_{70}H_{38}B_2F_{30}O_2Fe$: C, 53.95; H, 2.46. Found: C, 54.44; H, 2.91.

X-ray crystal structure analysis of 11c (erk8968): A green plate-like specimen of $C_{60}H_{28}B_2F_{30}O_2Fe \cdot 2 \times CH_2Cl_2$, approximate dimensions 0.158 mm x 0.273 mm x 0.373 mm, was used for the X-ray crystallographic analysis. The X-ray intensity data were measured. A total of 470 frames were collected. The total exposure time was 3.92 hours. The frames were integrated with the Bruker SAINT software package using a narrow-frame algorithm. The integration of the data using a triclinic unit cell yielded a total of 27339 reflections to a maximum θ angle of 25.47° (0.83 Å resolution), of which 6313 were independent (average redundancy 4.331, completeness = 99.3%, R_{int} = 6.48%, R_{sig} = 4.80%) and 5173 (81.94%) were greater than $2\sigma(F^2)$. The final cell constants of <u>a</u> = 11.863(4) Å, <u>b</u> = 13.004(5) Å, <u>c</u> = 13.207(5) Å, α = 106.631(10)°, β = 116.366(11)°, γ = 92.518(12)°, volume = 1714.0(11) $Å^3$, are based upon the refinement of the XYZ-centroids of 9546 reflections above 20 σ(I) with 4.798° < 2θ < 50.90°. Data were corrected for absorption effects using the multi-scan method (SADABS). The ratio of minimum to maximum apparent transmission was 0.818. The calculated minimum and maximum transmission coefficients (based on crystal size) are 0.8340 and 0.9250. The structure was solved and refined using the Bruker SHELXTL Software Package, using the space group $P\bar{1}$, with Z = 1 for the formula unit, $C_{60}H_{28}B_2F_{30}O_2Fe \cdot 2 \times CH_2Cl_2$. The final anisotropic full-matrix least-squares refinement on F^2 with 535 variables converged at R1 = 3.85%, for the observed data and wR2 = 9.58% for all data. The goodness-of-fit was 1.029. The largest peak in the final difference electron density synthesis was 0.349 $e^-/Å^3$ and the largest hole was -0.437 $e^-/Å^3$ with an RMS deviation of 0.068 $e^-/Å^3$. On the basis of the final model, the calculated density was 1.620 g/cm^3 and F(000), 832 e^-.

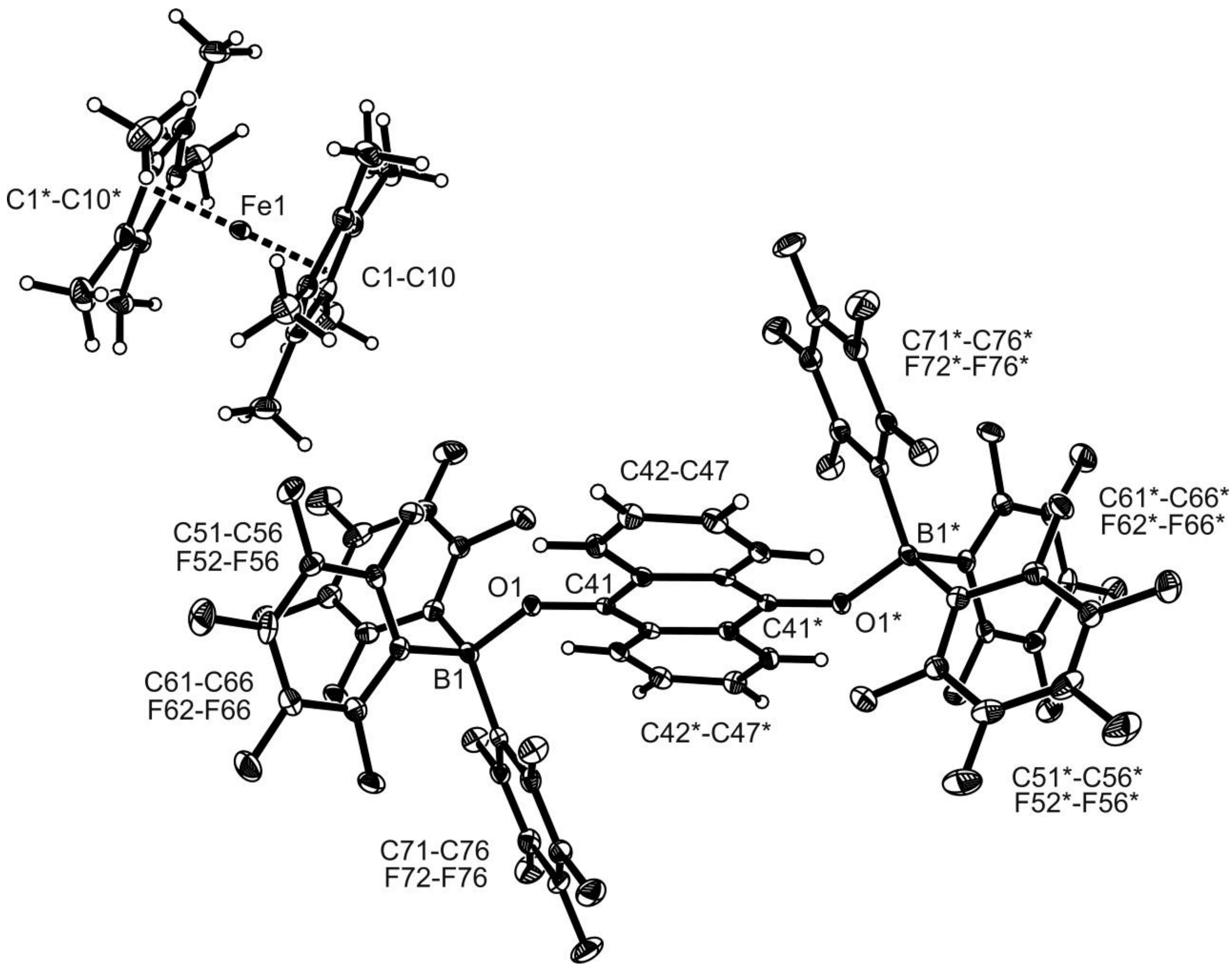

Figure S36. Crystal structure of compound **11c**. The cation and the radical anion are generated over inversion centre. (Thermals ellipsoids are shown with 50% probability.)

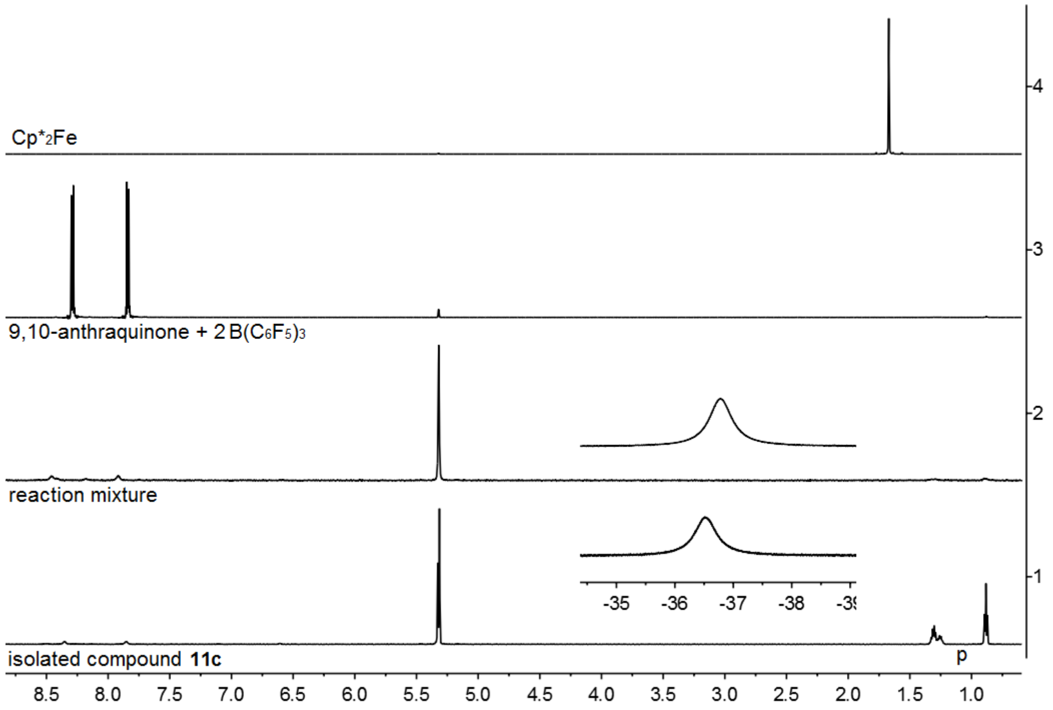

Figure S37. ^{1}H NMR (600 MHz, 299 K, $CD_2Cl_2^*$) spectra of (1) isolated compound **11c**, (2) reaction mixture, (3) decamethylferrocene and (4) a mixture of 9,10-anthraquinone and 2 equiv. of $B(C_6F_5)_3$. [p: pentane]

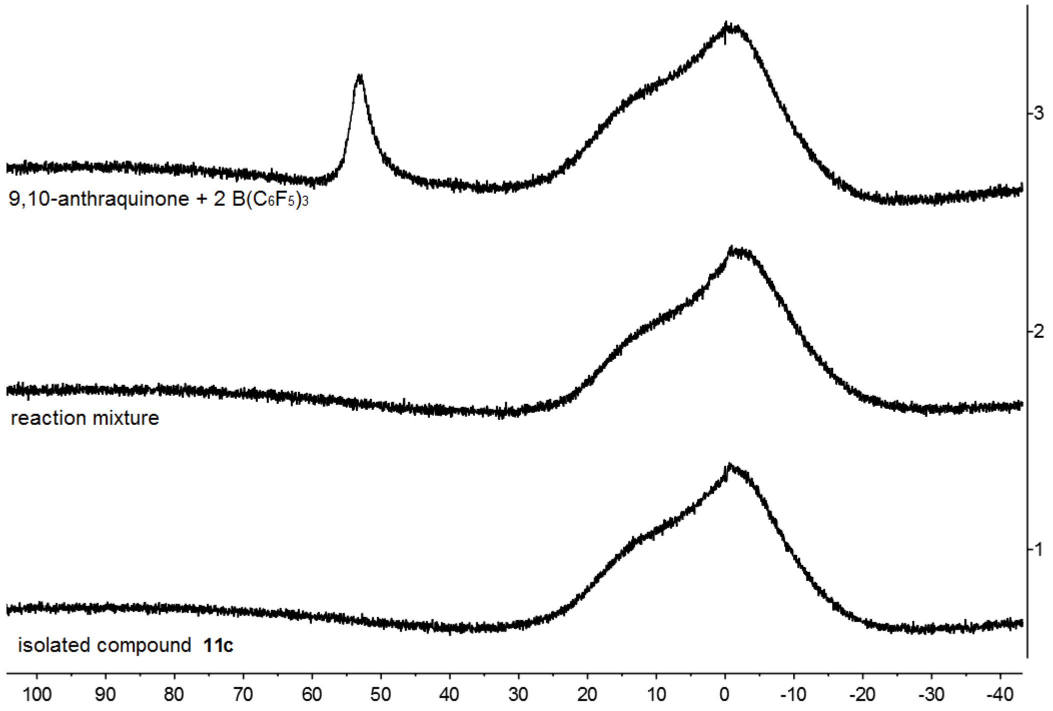

Figure S38. $^{11}B\{^{1}H\}$ NMR (192 MHz, 299 K, CD_2Cl_2) spectra of (1) isolated compound **11c**, (2) reaction mixture and (3) mixture of 9,10-anthraquinone and 2 equiv. of $B(C_6F_5)_3$.

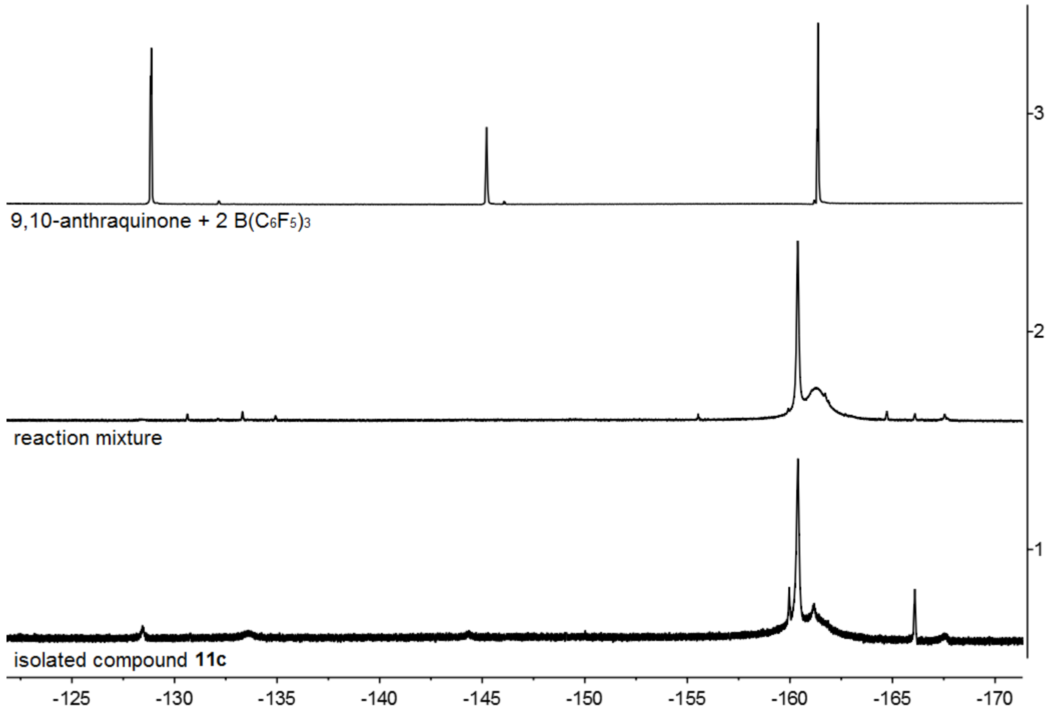

Figure S39. ^{19}F NMR (564 MHz, 299 K, CD_2Cl_2)spectra of (1) isolated compound **11c**, (2) reaction mixture and (3) a mixture of 9,10-anthraquinone and 2 equiv. of $B(C_6F_5)_3$.

H) Reaction of acenaphthenequinone , 2 $B(C_6F_5)_3$ and TEMPO

Scheme S16

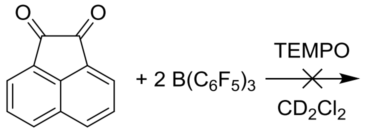

Acenaphthenequinone (18.2 mg, 0.100 mmol) and $B(C_6F_5)_3$ (102 mg, 0.200 mmol) were mixed in CD_2Cl_2 (1.0 mL). Subsequently, TEMPO (15.6 mg, 0.100 mmol) was added. The resulting yellow solution was characterized by NMR experiments.

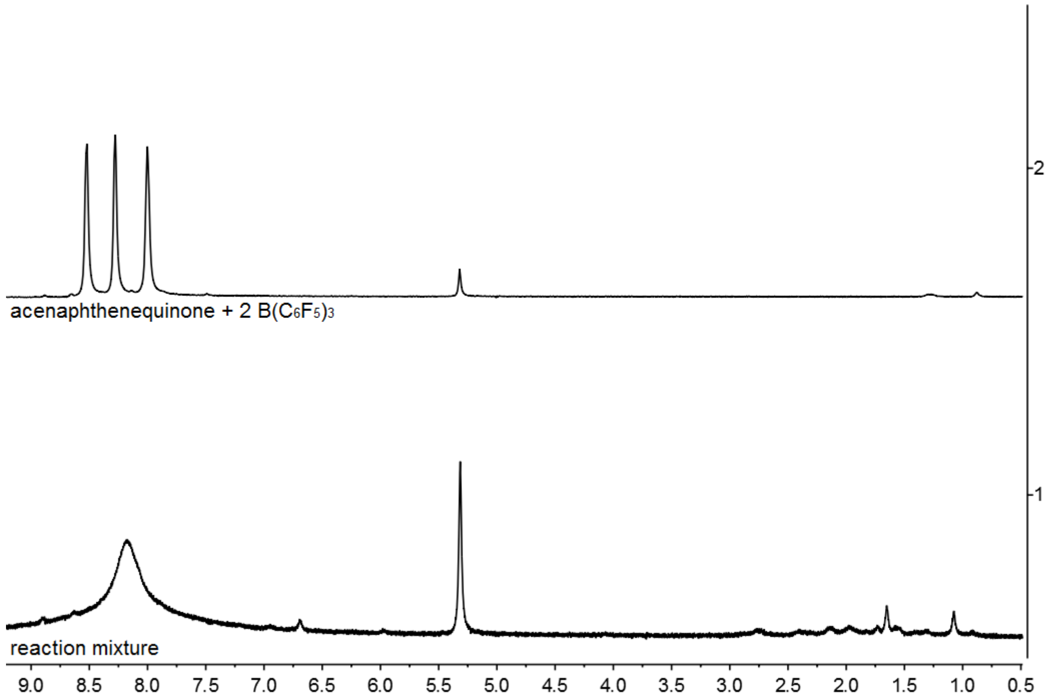

Figure S40. ^{1}H NMR (600 MHz, 299 K, CD_2Cl_2) spectra of (1) reaction mixture and (2) the mixture of acenaphthenequinone with 2 equiv. of $B(C_6F_5)_3$.

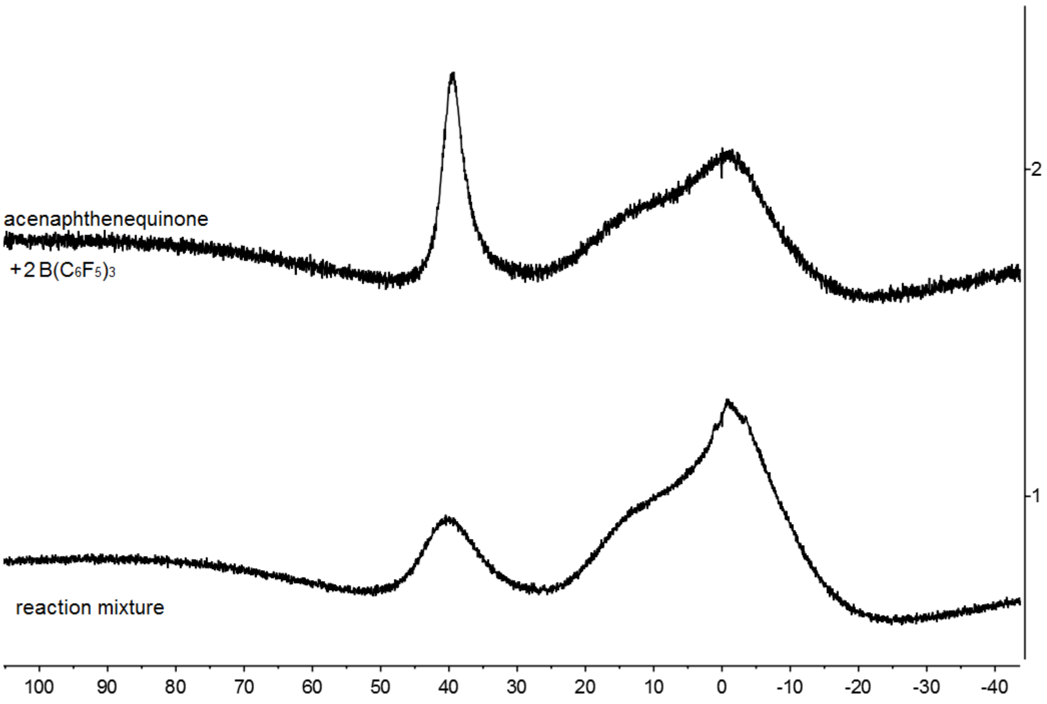

Figure S41. $^{11}B\{^{1}H\}$ NMR (192 MHz, 299 K, CD_2Cl_2) spectra of (1) reaction mixture and (2) the mixture of acenaphthenequinone with 2 equiv. of $B(C_6F_5)_3$.

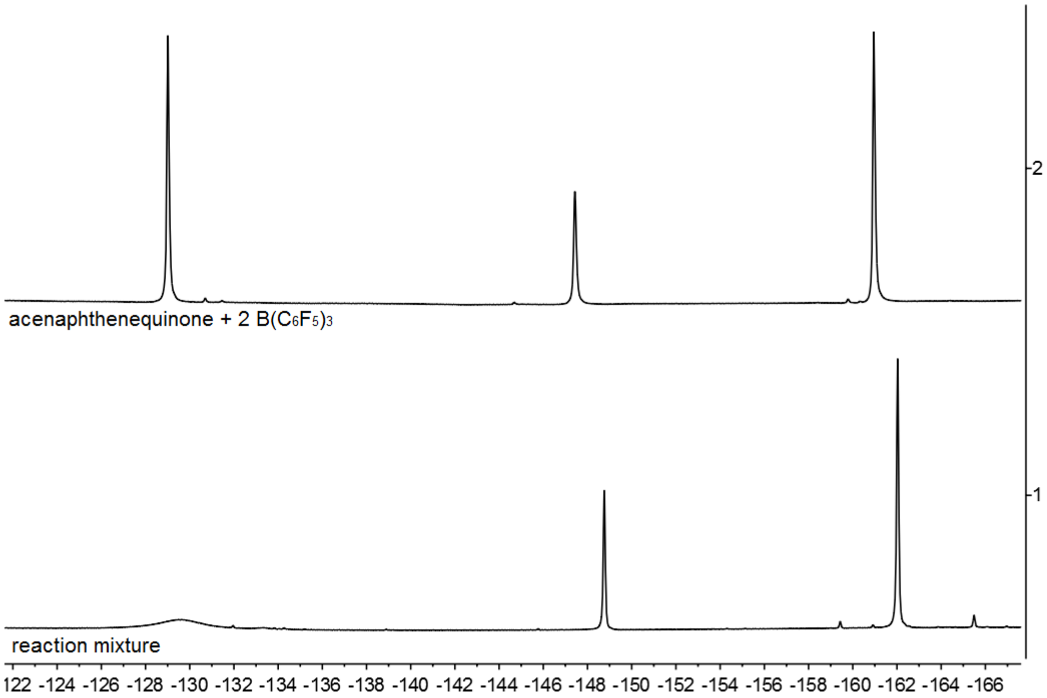

Figure S42. ^{19}F NMR (564 MHz, 299 K, CD_2Cl_2) spectra of (1) reaction mixture and (2) the mixture of acenaphthenequinone with 2 equiv. of $B(C_6F_5)_3$.

I) Generation of compound **12b**

Experiment 1: [reaction of Gomberg dimer with acenaphthenequinone (1:2)]

Scheme S17

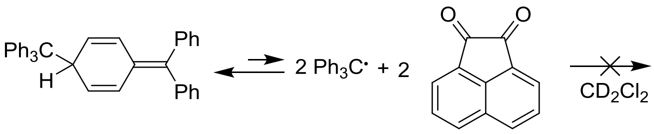

Acenaphthenequinone (18.2 mg, 0.10 mmol) and Gomberg dimer (24.3 mg, 0.050 mmol) were mixed in CD_2Cl_2 (1 mL) at room temperature. After stirring for 1 hour, the resulting reaction mixture (white suspension) was characterized by NMR experiments.

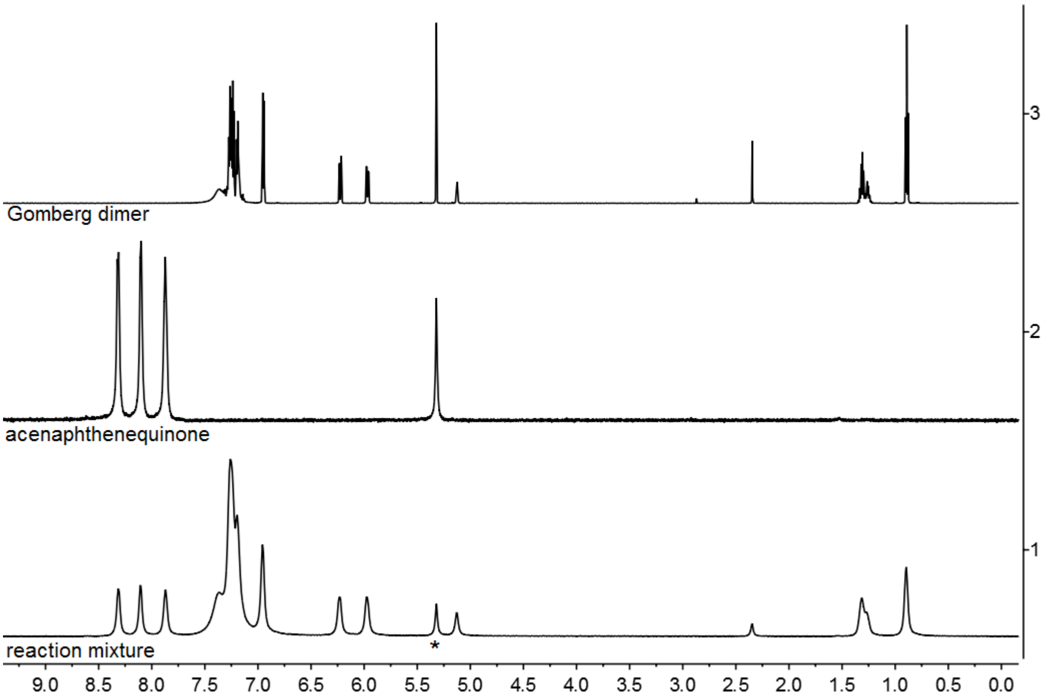

Figure S43. ^{1}H NMR (600 MHz, 299 K, CD_2Cl_2*) spectra of (1) reaction mixture, (2) acenaphthenequinone, (3)Gombreg dimer. [p: pentane, tol: toluene]

.

Experiment 2: (reaction of acenaphthenequinone with 2 $B(C_6F_5)_3$ and Gomberg dimer)

Scheme S18

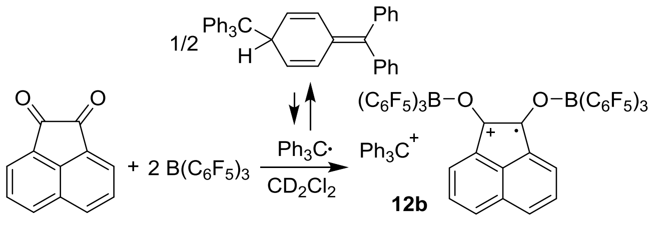

Acenaphthenequinone (18.2 mg, 0.100 mmol) and $B(C_6F_5)_3$ (102 mg, 0.200 mmol) were mixed in CD_2Cl_2 (1.0 mL). The resulting red solution was characterized by NMR experiments. Subsequently, Gomberg dimer (24.3 mg, 0.050 mmol) was added. The resulting dark green reaction mixture was characterized by NMR experiments. After gas phase diffusion of pentane to the resulting reaction solution and at –35 °C for 7 days, a dark green oil precipitated. The solution was separated by decantation. After diffusion of pentane to the separated solution at –35 °C for 7 days, dark green crystals were observed. Removal of the solution by decantation gave several crystals, which were suitable for the X-ray crystal structure analysis (under microscope the crystals with different colors were observed, the dark green crystals were picked for measurement).

X-ray crystal structure analysis of 12b (erk8958): A orange plate-like specimen of $C_{67}H_{21}B_2F_{30}O_2 \cdot 2 \times CH_2Cl_2$, approximate dimensions 0.060 mm x 0.120 mm x 0.220 mm, was used for the X-ray crystallographic analysis. The X-ray intensity data were measured. A total of 1461 frames were collected. The total exposure time was 23.71 hours. The frames were integrated with the Bruker SAINT software package using a wide-frame algorithm. The integration of the data using a triclinic unit cell yielded a total of 44978 reflections to a maximum θ angle of 66.67° (0.84 Å resolution), of which 10958 were independent (average redundancy 4.105, completeness = 99.1%, R_{int} = 8.81%, R_{sig} = 7.45%) and 7134 (65.10%) were greater than $2\sigma(F^2)$. The final cell constants of a = 13.3556(6) Å, b = 14.9486(7) Å, c = 17.5414(8) Å, α = 95.477(3)°, β = 111.222(3)°, γ = 102.632(3)°, volume = 3126.3(3) $Å^3$, are based upon the refinement of the XYZ-centroids of 5198 reflections above 20 σ(I) with 6.174° < 2θ < 132.0°. Data were corrected for absorption effects using the multi-scan method (SADABS). The ratio of minimum to maximum apparent transmission was 0.836. The calculated minimum and maximum transmission coefficients (based on crystal size) are 0.5590 and 0.8410. The structure was solved and refined using the Bruker SHELXTL Software Package, using the space group $P\bar{1}$, with Z = 2 for the formula unit, $C_{67}H_{21}B_2F_{30}O_2 \cdot 2 \times CH_2Cl_2$. The final anisotropic full-matrix least-squares refinement on F^2 with 1020 variables converged at R1 = 5.65%, for the observed data and wR2 = 16.09% for all data. The goodness-of-fit was 1.027. The largest peak in the final difference electron density synthesis was 0.421 $e^-/Å^3$ and the largest hole was -0.560 $e^-/Å^3$ with an RMS deviation of 0.070 $e^-/Å^3$. On the basis of the final model, the calculated density was 1.720 g/cm^3 and F(000), 1606 e^-.

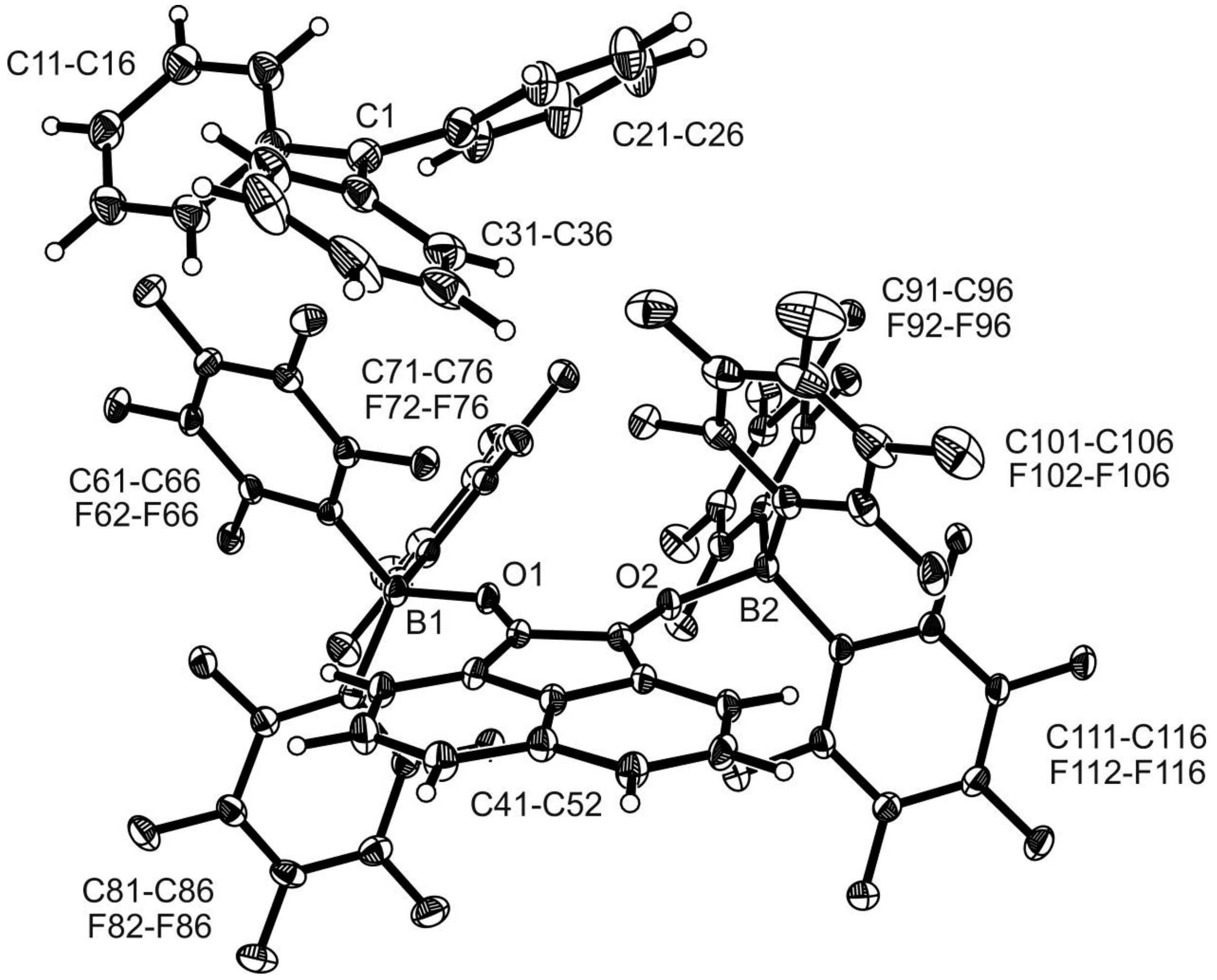

Figure S44. Crystal structure of compound **12b**. (Thermals ellipsoids are shown with 30% probability.)

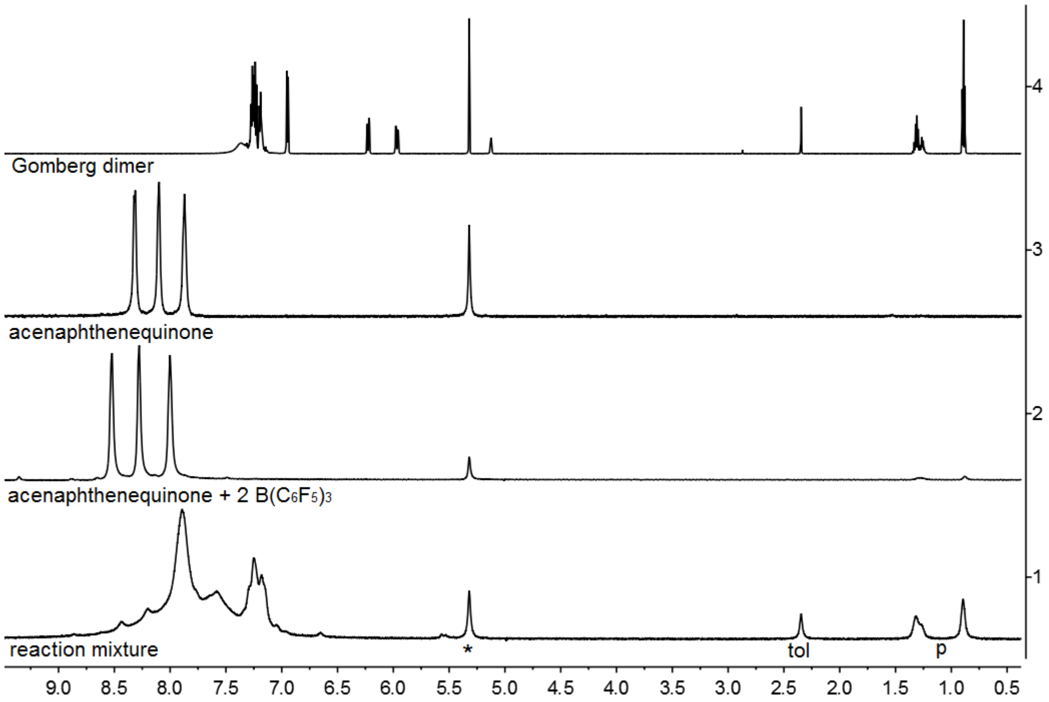

Figure S45. ^{1}H NMR (600 MHz, 299 K, CD_2Cl_2*) spectra of (1) reaction mixture, (2) the mixture of acenaphthenequinone with 2 equiv. of $B(C_6F_5)_3$ and (3) acenaphthenequinone, (4) Gomberg dimer. [tol: toluene, p: pentane]

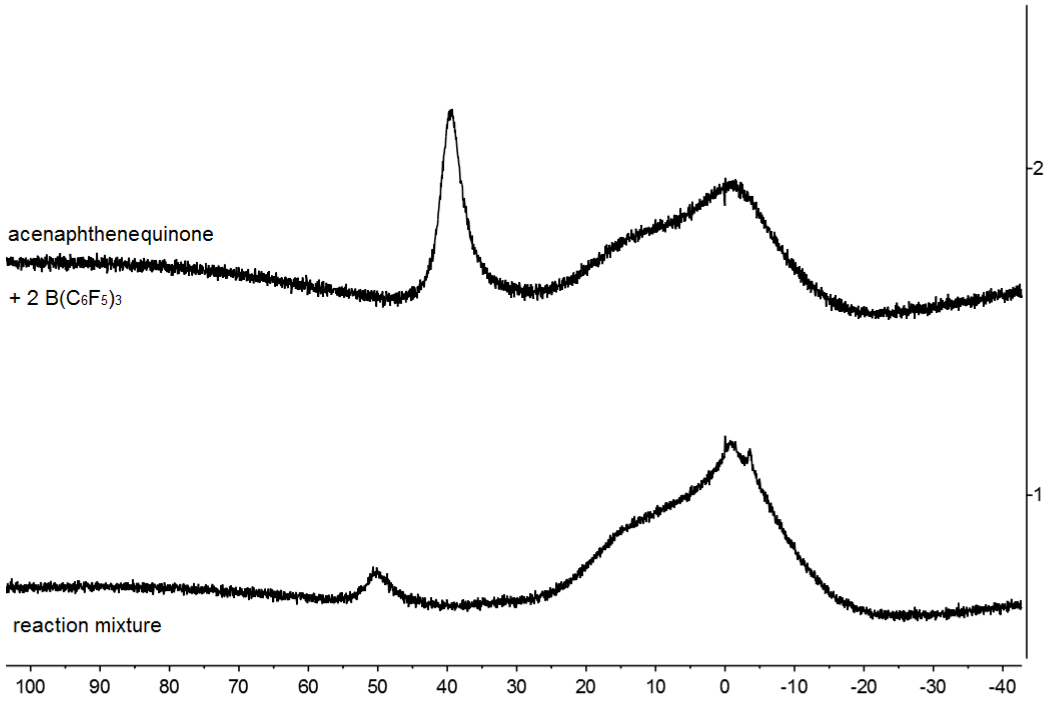

Figure S46. $^{11}B\{^{1}H\}$ NMR (192 MHz, 299 K, CD_2Cl_2) spectra of (1) reaction mixture and (2) the mixture of acenaphthenequinone with 2 equiv. of $B(C_6F_5)_3$.

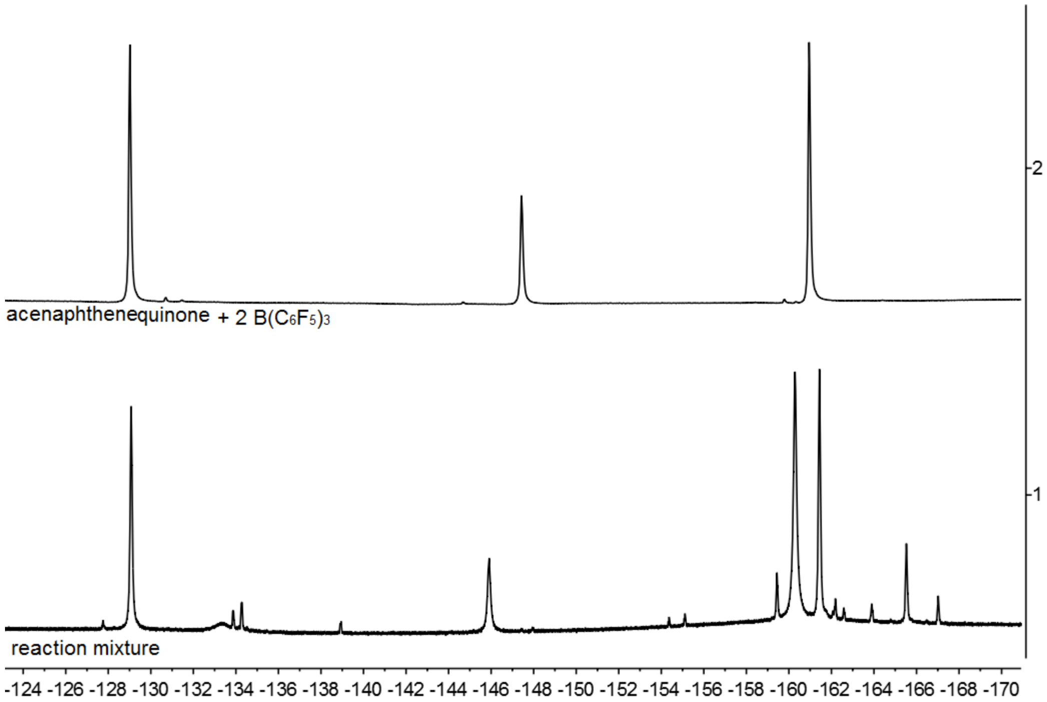

Figure S47. ^{19}F NMR (564 MHz, 299 K, CD_2Cl_2) spectra of (1) reaction mixture and (2) the mixture of acenaphthenequinone with 2 equiv. of $B(C_6F_5)_3$.

J) Synthesis of compound **12c**

Experiment 1: [reaction of decamethylferrocene with acenaphthenequinone (1:1)]

Scheme S19

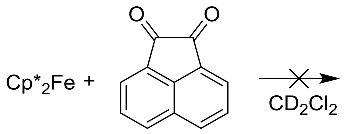

Acenaphthenequinone (9.1 mg, 0.050 mmol) and decamethylferrocene (16.3 mg, 0.050 mmol) were mixed in CD_2Cl_2 (1 mL) at room temperature. After 1 hour, the resulting reaction mixture was characterized by NMR experiments.

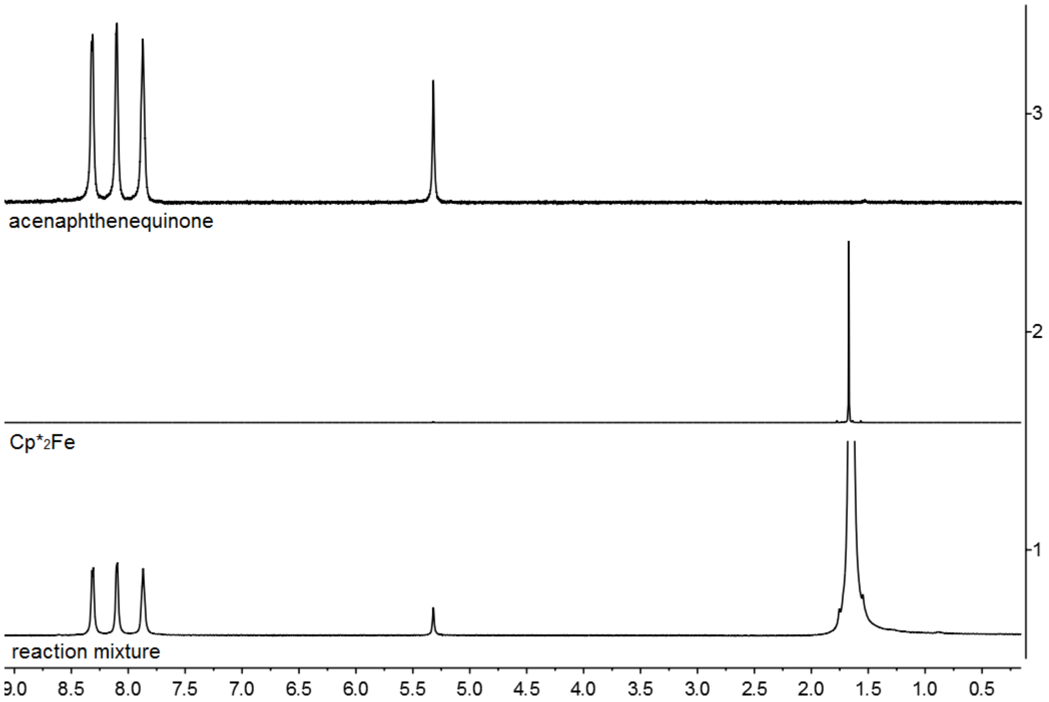

Figure S48. ^{1}H NMR (600 MHz, 299 K, CD_2Cl_2*) spectra of (1) reaction mixture, (2) decamethylferrocene and (3) acenaphthenequinone.

.

Experiment 2: (reaction of acenaphthenequinone with 2 $B(C_6F_5)_3$ and decamethylferrocene, isolation of compound **12c**)

Scheme S20

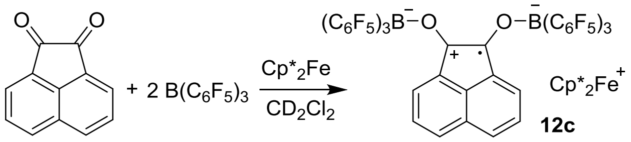

Acenaphthenequinone (18.2 mg, 0.100 mmol) and $B(C_6F_5)_3$ (102 mg, 0.200 mmol) were mixed in CD_2Cl_2 (1.0 mL) resulting a red solution. Subsequently, decamethylferrocene (32.6 mg, 0.100 mmol)

was added. The resulting dark green reaction mixture was characterized by NMR experiments. After storage of the reaction solution and at –35 °C for 24 h, the dark green solid precipitated. Removal of the solution was performed by decantation, and part of the crystals were used for the X-ray crystal structure analysis. Drying of the remaining crystals gave compound **12c** (105 mg, 0.069, 69%) as dark green crystalline material.

Decomp.:242 °C

Anal. Calc. for $C_{68}H_{36}B_2F_{30}O_2Fe$: C, 53.23; H, 2.50. Found: C, 52.89; H, 2.37.

NMR data of isolated compound **12c**:

^{1}H NMR (600 MHz, 299 K, CD_2Cl_2): δ ^{1}H: –36.7 (br s, 30H, $Cp^*{}_2Fe^+$).

^{19}F NMR (564 MHz, 299 K, CD_2Cl_2): δ ^{19}F: see Figure S53.

^{11}B{^{1}H} NMR (192 MHz, 299 K, CD_2Cl_2): δ ^{11}B: see Figure S52.

X-ray crystal structure analysis of 12c (erk8969): formula $C_{68}H_{36}B_2F_{30}FeO_2$, *M* = 1532.44, green-orange crystal, 0.11 x 0.10 x 0.03 mm, *a* = 13.3457(3), *b* = 14.5750(3), *c* = 17.8933(4) Å, α = 91.771(1), *β* = 97.044(1), γ = 102.641(1)°, *V* = 3364.5(1) Å^3, ρ_{calc} = 1.513 gcm^{-3}, μ = 0.352 mm^{-1}, empirical absorption correction (0.962 ≤ T ≤ 0.989), *Z* = 2, triclinic, space group $P\bar{1}$ (No. 2), λ = 0.71073 Å, *T* = 173(2) K, ω and ϕ scans, 35096 reflections collected (±*h*, ±*k*, ±*l*), 11683 independent (R_{int} = 0.048) and 9407 observed reflections [I>2σ(I)], 938 refined parameters, *R* = 0.050, wR^2 = 0.113, max. (min.) residual electron density 0.25 (-0.33) e.Å^{-3}, the position of the hydrogen were calculated and refined as riding atoms.

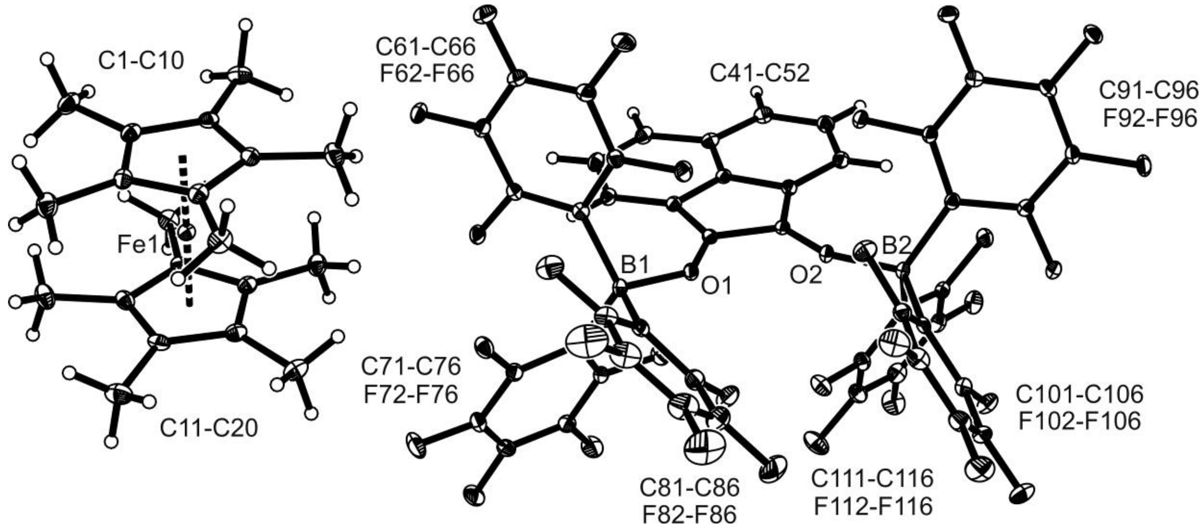

Figure S49. Crystal structure of compound **12c**. (Thermals ellipsoids are shown with 15% probability.)

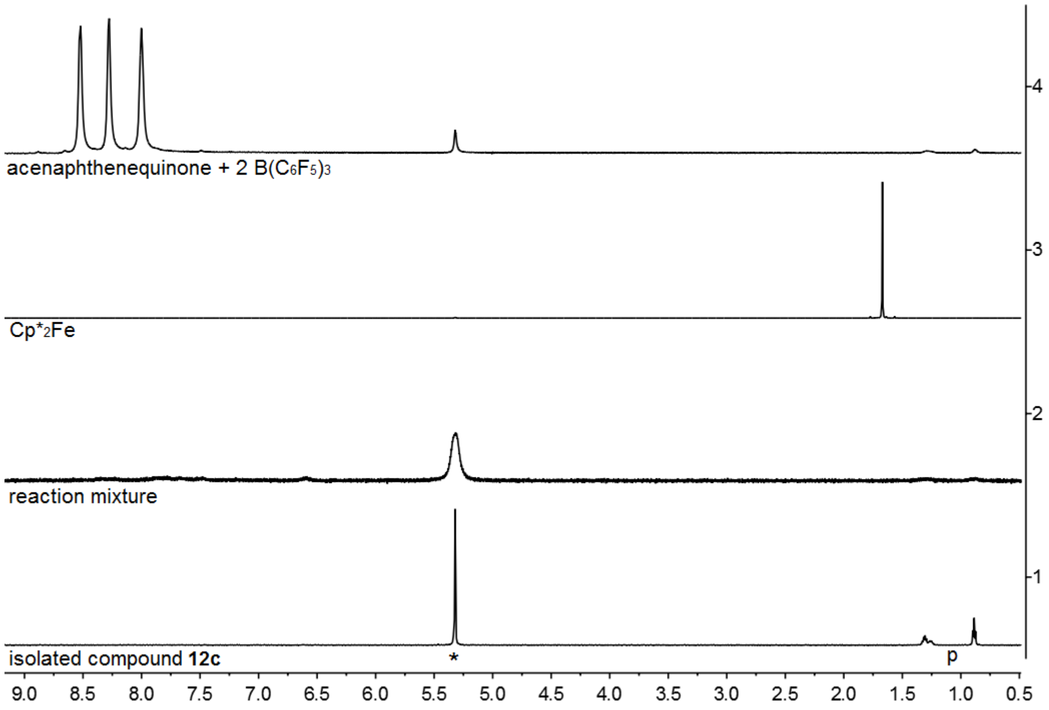

Figure S50. ^{1}H NMR (600 MHz, 299 K, $CD_2Cl_2^*$) spectra of (1) isolated compound **12c**, (2) reaction mixture, (3) decamethylferrocene and (4) a mixture of acenaphthenequinone and 2 equiv. of $B(C_6F_5)_3$. [p: pentane]

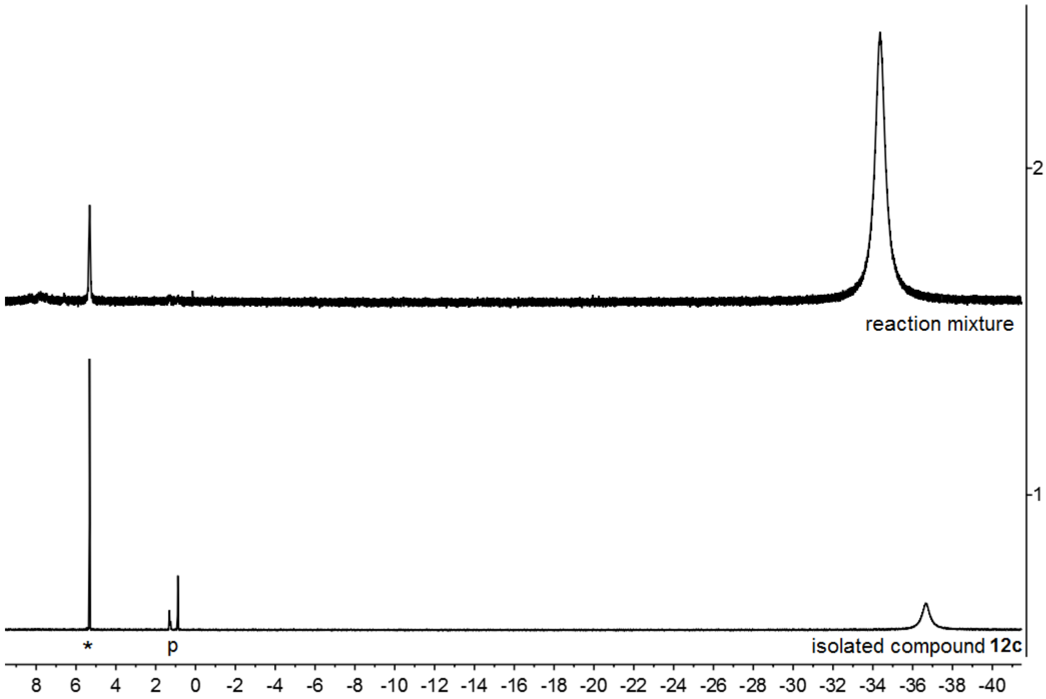

Figure S51. ^{1}H NMR (600 MHz, 299 K, $CD_2Cl_2^*$) spectra of (1) isolated compound **12c** and (2) reaction mixture.

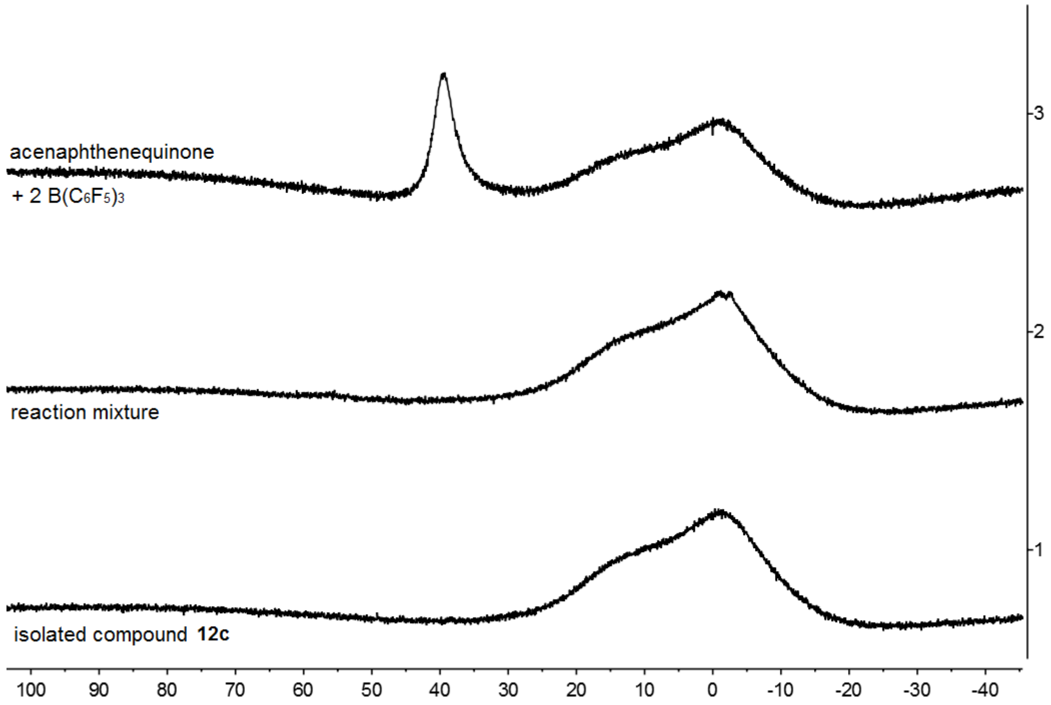

Figure S52. $^{11}B\{^1H\}$ NMR (192 MHz, 299 K, CD_2Cl_2) spectra of (1) isolated compound **12c**, (2) reaction mixture and (3) a mixture of acenaphthenequinone and 2 equiv. of $B(C_6F_5)_3$.

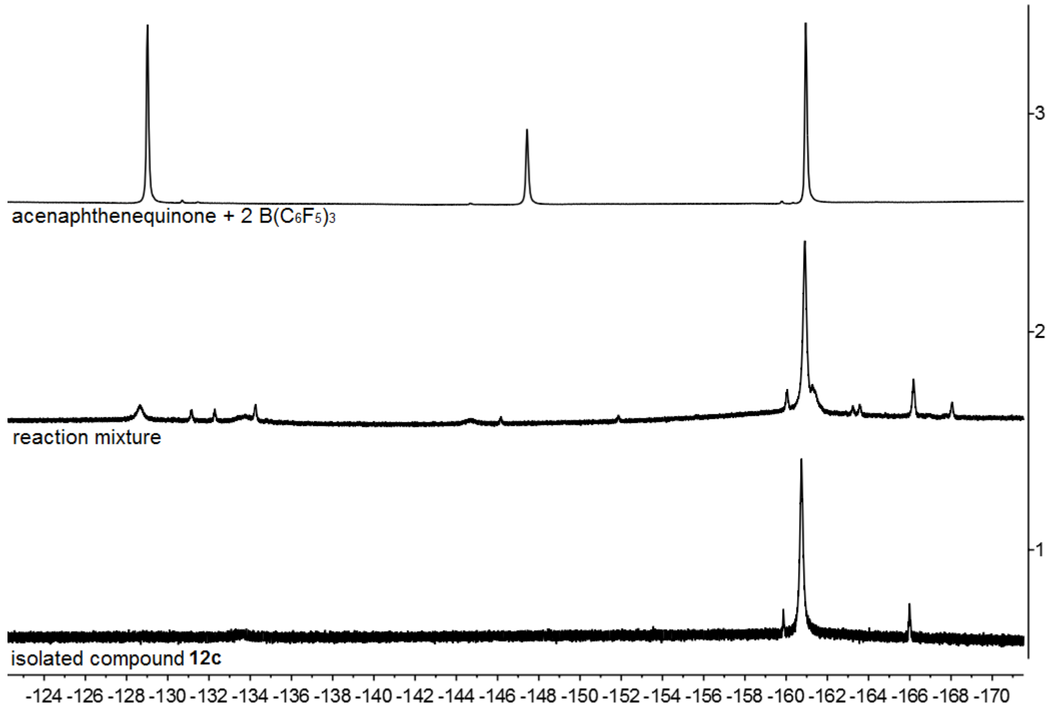

Figure S53. ^{19}F NMR (564 MHz, 299 K, CD_2Cl_2) spectra of (1) isolated compound **12c**, (2) reaction mixture and (3) a mixture of acenaphthenequinone and 2 equiv. of $B(C_6F_5)_3$.

K) Reaction of 9,10-phenanthrenequinone, 2 $B(C_6F_5)_3$ and TEMPO

Scheme S21

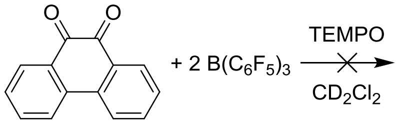

9,10-Phenanthrenequinone (20.8 mg, 0.100 mmol) and $B(C_6F_5)_3$ (102 mg, 0.200 mmol) were mixed in CD_2Cl_2 (1.0 mL). Subsequently, TEMPO (15.6 mg, 0.100 mmol) was added. The resulting dark brown solution was characterized by NMR experiments.

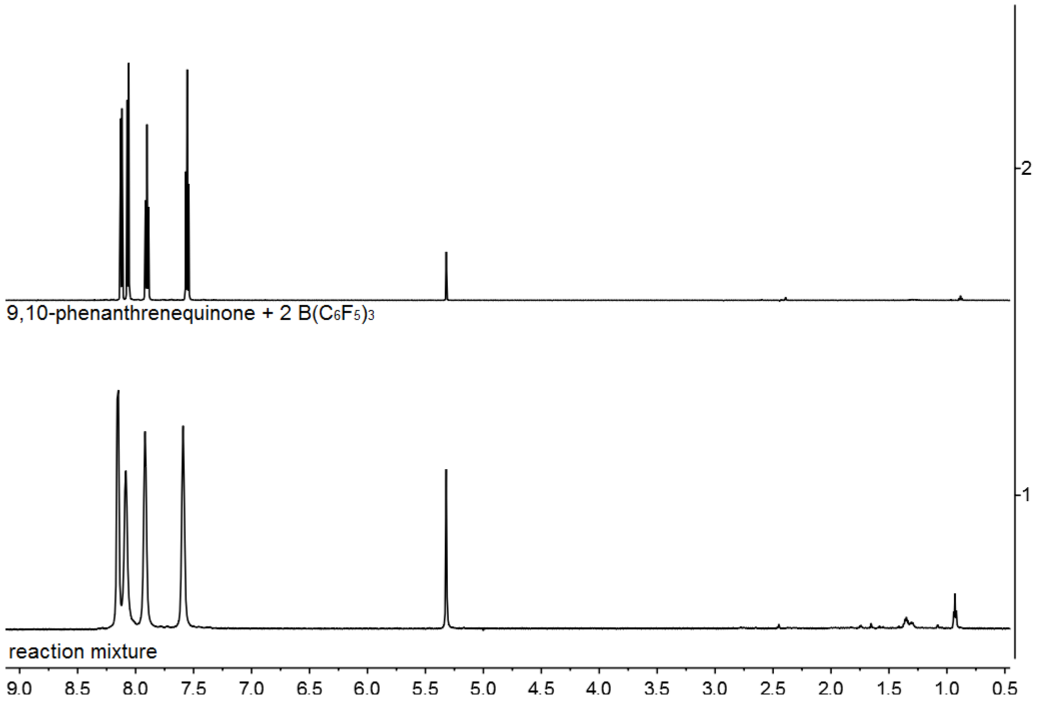

Figure S54. ^1H NMR (600 MHz, 299 K, CD_2Cl_2) spectra of (1) reaction mixture and (2) the mixture of 9,10-phenanthrenequinone with 2 equiv. of $B(C_6F_5)_3$.

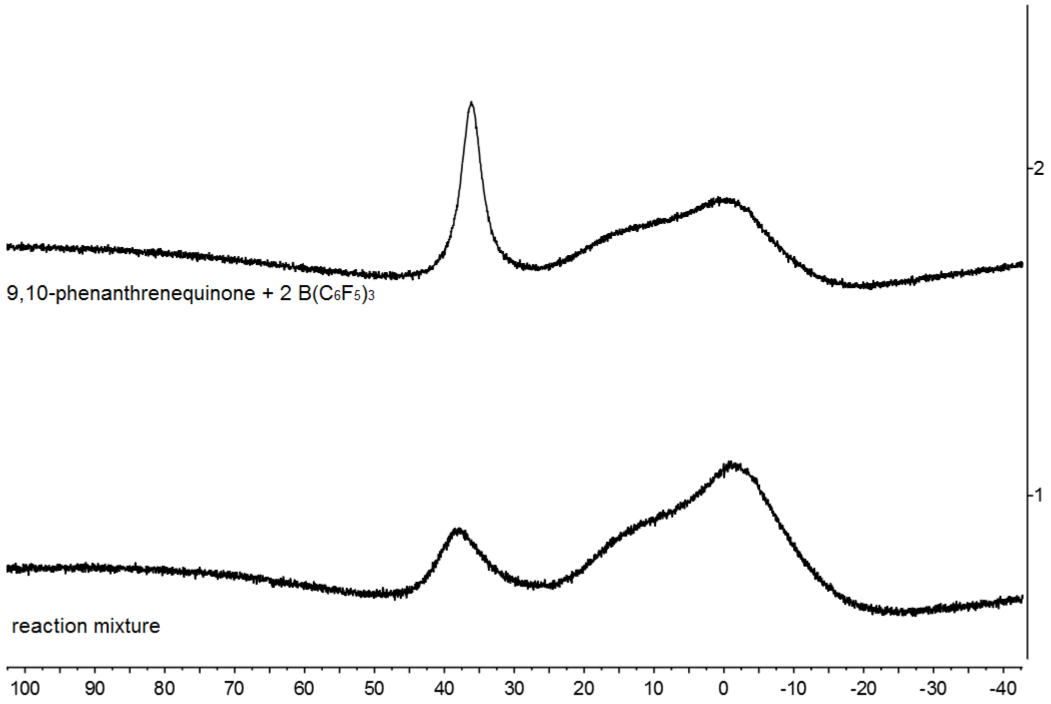

Figure S55. $^{11}B\{^{1}H\}$ NMR (192 MHz, 299 K, CD_2Cl_2) spectra of (1) reaction mixture and (2) the mixture of 9,10-phenanthrenequinone with 2 equiv. of $B(C_6F_5)_3$.

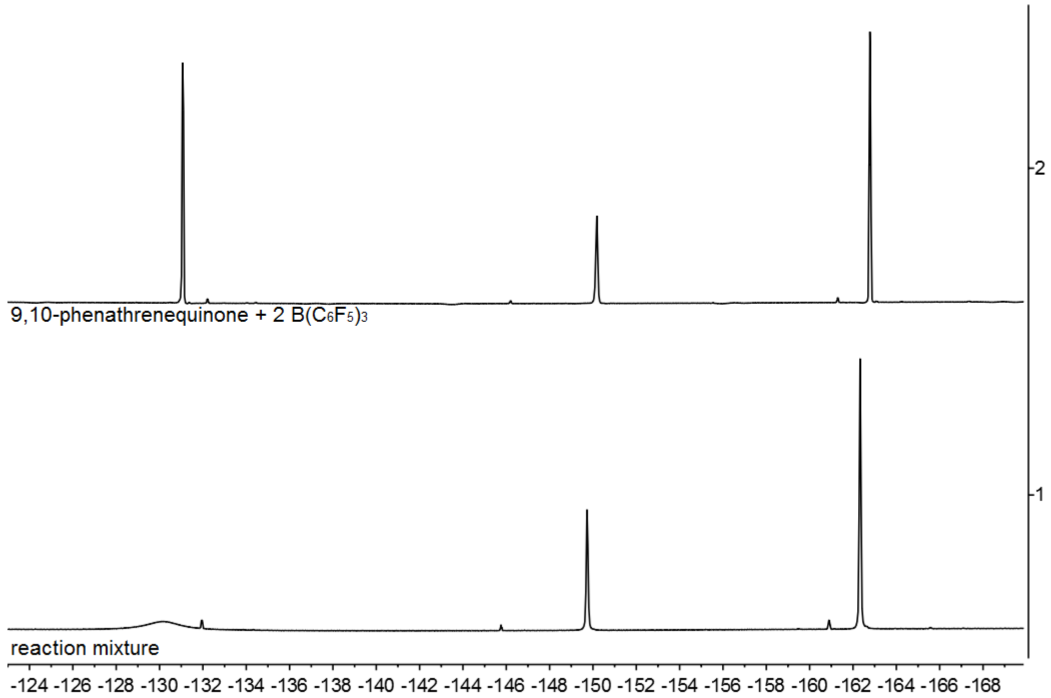

Figure S56. ^{19}F NMR (564 MHz, 299 K, CD_2Cl_2) spectra of (1) reaction mixture and (2) the mixture of 9,10-phenanthrenequinone with 2 equiv. of $B(C_6F_5)_3$.

L) Synthesis of compound **13c**

Experiment 1: [reaction of decamethylferrocene with 9,10-phenanthrenequinone (1:1)]

Scheme S22

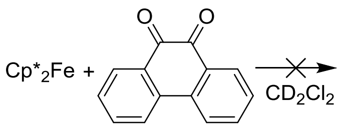

9,10-Phenanthrenequinone (20.8 mg, 0.100 mmol) and decamethyferrocene (32.6 mg, 0.100 mmol) were mixed in CD_2Cl_2 (1 mL) at room temperature. After 1 hour, the resulting reaction mixture was characterized by NMR experiments.

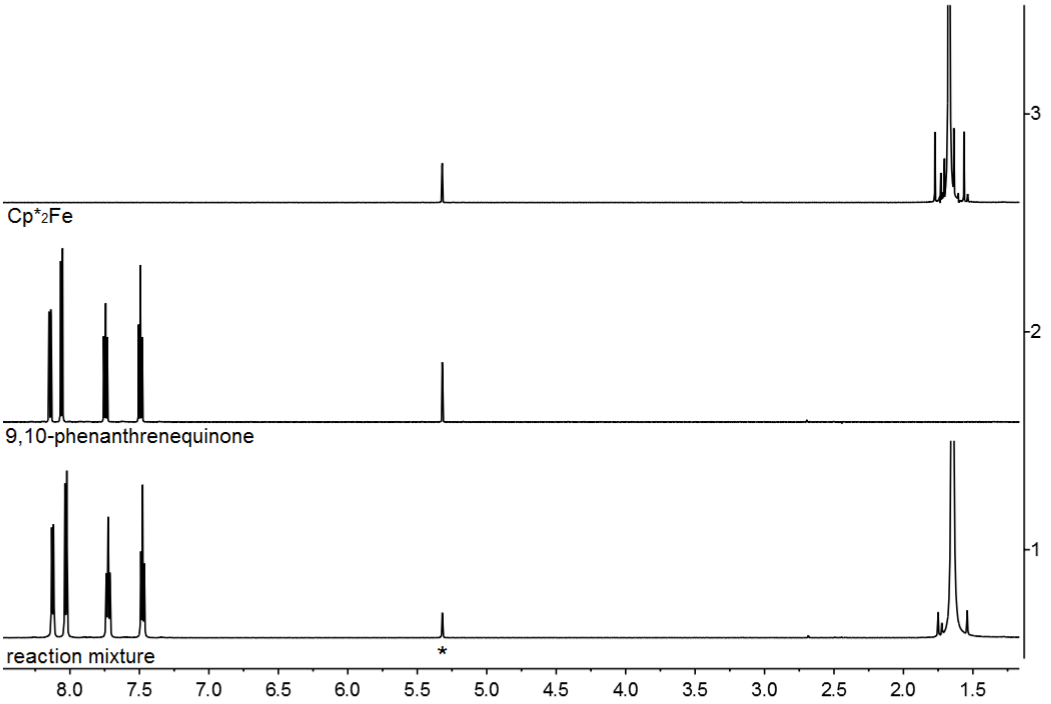

Figure S57. ^{1}H NMR (600 MHz, 299 K, CD_2Cl_2*) spectra of (1) reaction mixture, (2) 9,10-phenanthrenequinone and (3) decamethylferrocene.

.

Experiment 2: [reaction of 9,10-phenanthrenequinone with 2 $B(C_6F_5)_3$ and Cp^*_2Fe, isolation of compound **13c**]

Scheme S23

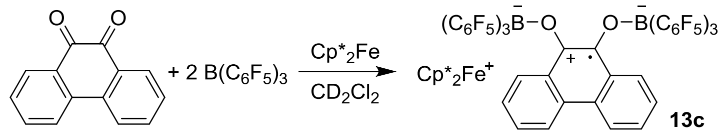

13c

9,10-Phenanthrenequinone (20.8 mg, 0.100 mmol) and $B(C_6F_5)_3$ (102 mg, 0.200 mmol) were mixed in CD_2Cl_2 (1.0 mL) to give a dark brown solution. Subsequently, decamethylferrocene (32.6 mg, 0.100 mmol) was added. The resulting dark brown reaction mixture was characterized by NMR experiments. After storage of the resulting reaction solution at –35 °C for 24h, the dark brown solid precipitated. Removal of the solution was carried out by decantation and part of the obtained crystalline material was used for the X-ray crystal structure analysis. Drying of the remaining crystals in vacuo gave compound **13c** (126 mg, 0.081 mmol, 81%) as dark brown crystalline material.

Decomp.: 158 °C

Anal. Calc. for $C_{70}H_{38}B_2F_{30}O_2Fe{\cdot}CH_2Cl_2$: C, 51.86; H, 2.51. Found: C, 51.39; H, 2.44.

NMR data of isolated compound **13c**:

^{1}H NMR (600 MHz, 299 K, CD_2Cl_2): δ ^{1}H: -36.7 (br s, 20H, $Cp^*_2Fe^+$). See Figure S59.

^{19}F NMR (564 MHz, 299 K, CD_2Cl_2): δ ^{19}F: see Figure S61.

^{11}B{^{1}H} NMR (192 MHz, 299 K, CD_2Cl_2): δ ^{11}B: see Figure S60.

X-ray crystal structure analysis of 13c (erk8997): A pale orange prism-like specimen of $C_{70}H_{38}B_2F_{30}FeO_2 \cdot CH_2Cl_2$, approximate dimensions 0.072 mm x 0.121 mm x 0.248 mm, was used for the X-ray crystallographic analysis. The X-ray intensity data were measured. A total of 2083 frames were collected. The total exposure time was 41.26 hours. The frames were integrated with the Bruker SAINT software package using a wide-frame algorithm. The integration of the data using a monoclinic unit cell yielded a total of 54819 reflections to a maximum θ angle of 66.59° (0.84 Å resolution), of which 11948 were independent (average redundancy 4.588, completeness = 99.9%, R_{int} = 9.31%, R_{sig} = 8.23%) and 9709 (81.26%) were greater than 2σ(F^2). The final cell constants of $\underline{a}$ = 12.6071(3) Å, $\underline{b}$ = 13.6253(4) Å, $\underline{c}$ = 20.3659(6) Å, β = 100.362(2)°, volume = 3441.31(17) Å^3, are based upon the refinement of the XYZ-centroids of 9852 reflections above 20 σ(I) with 7.128° < 2θ < 136.4°. Data were corrected for absorption effects using the multi-scan method (SADABS). The ratio of minimum to maximum apparent transmission was 0.775. The calculated minimum and maximum transmission coefficients (based on crystal size) are 0.4660 and 0.7800. The structure was solved and refined using the Bruker SHELXTL Software Package, using the space group $P2_1$, with Z = 2 for the formula unit, $C_{70}H_{38}B_2F_{30}FeO_2 \cdot CH_2Cl_2$. The final anisotropic full-matrix least-squares refinement on F^2 with 1011 variables converged at R1 = 4.92%, for the observed data and wR2 = 9.69% for all data. The goodness-of-fit was 1.027. The largest peak in the final difference electron density

synthesis was 0.601 $e^-/Å^3$ and the largest hole was -0.312 $e^-/Å^3$ with an RMS deviation of 0.056 $e^-/Å^3$. On the basis of the final model, the calculated density was 1.586 g/cm^3 and F(000), 1644 e^-.

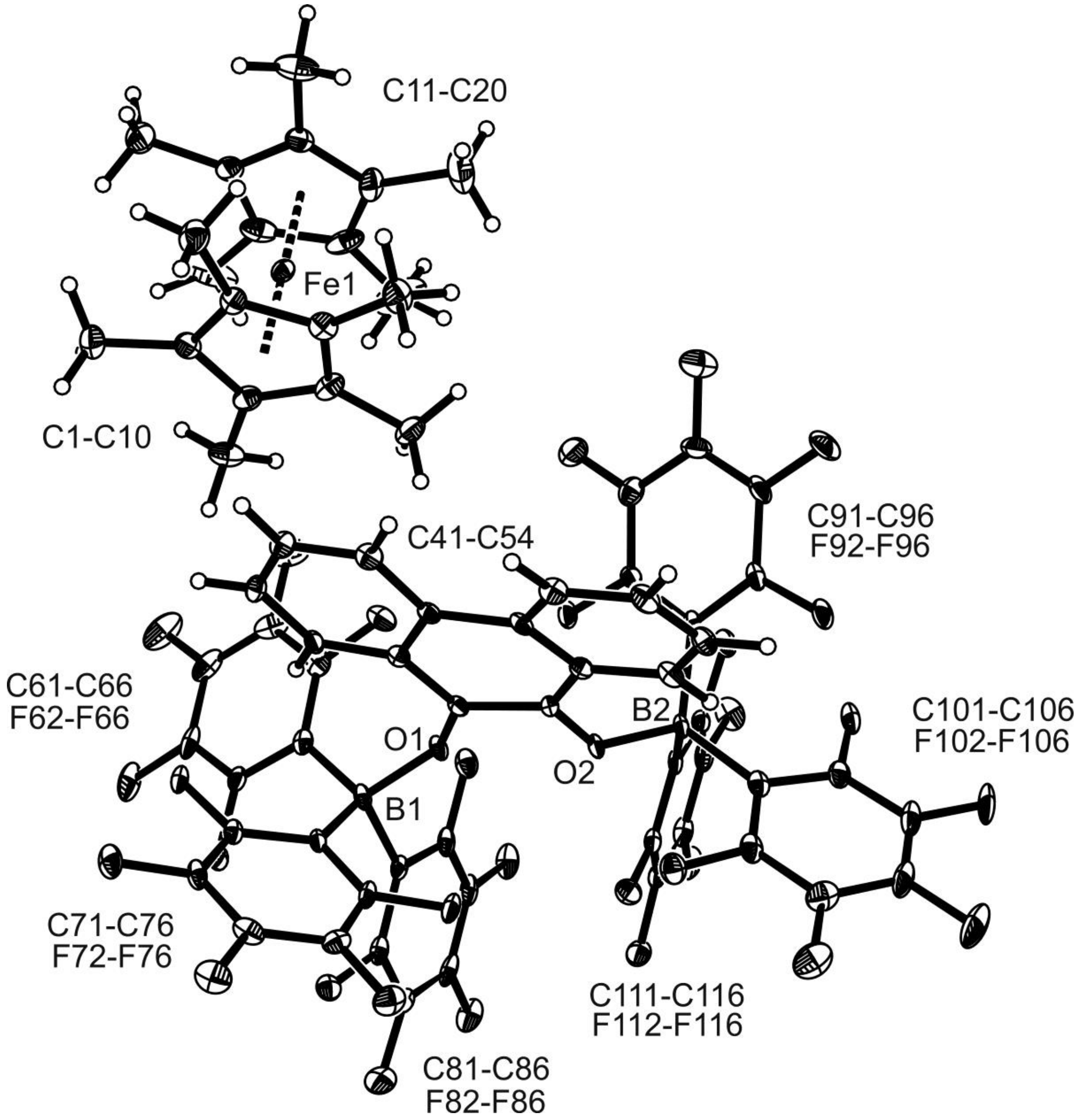

Figure S58. Crystal structure of compound **13c**. (Thermals ellipsoids are shown with 30% probability.)

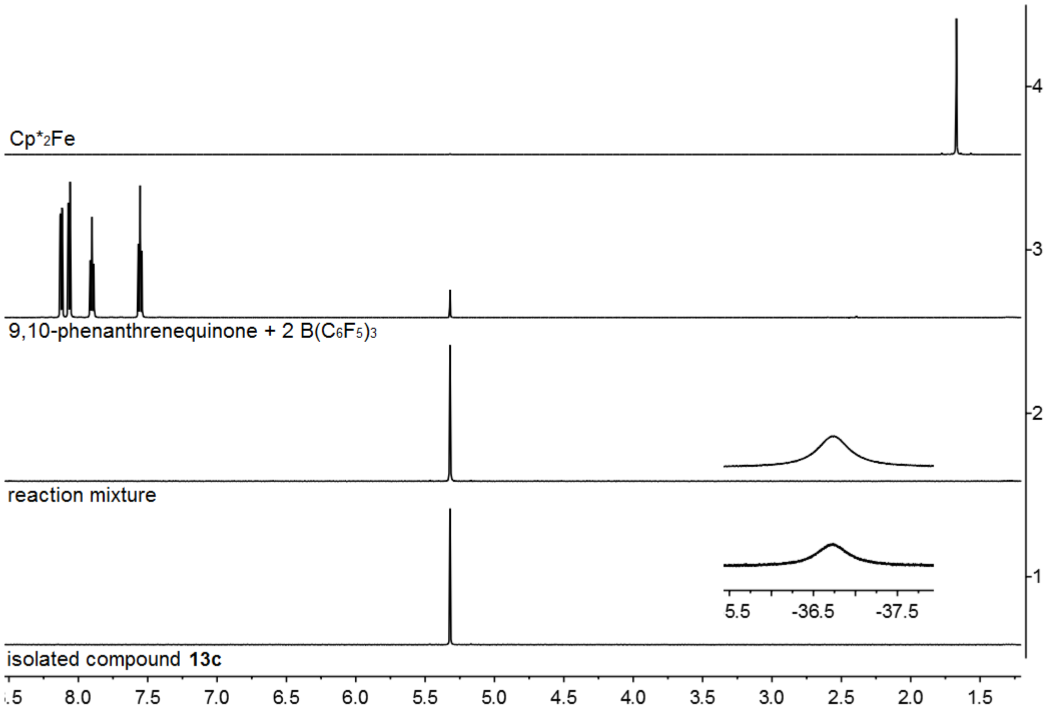

Figure S59. ^{1}H NMR (600 MHz, 299 K, $CD_2Cl_2^{*}$) spectra of (1) isolated compound **13c**, (2) reaction mixture, (3) decamethylferrocene and (4) a mixture of 9,10-phenanthrenequinone and 2 equiv. of $B(C_6F_5)_3$.

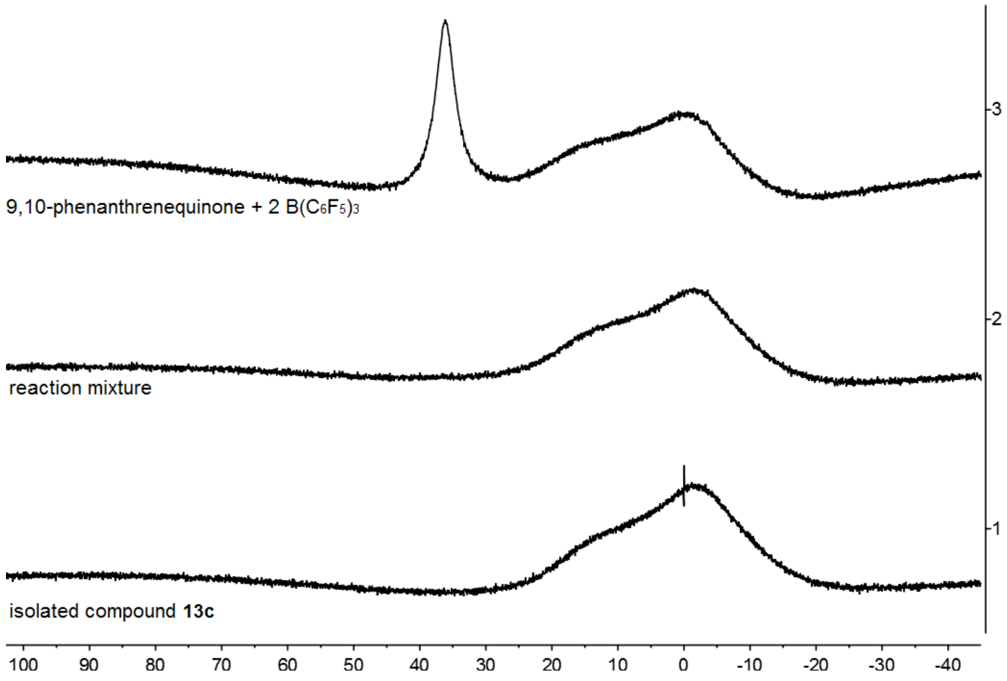

Figure S60. $^{11}B\{^{1}H\}$ NMR (192 MHz, 299 K, CD_2Cl_2) spectra of (1) isolated compound **13c**, (2) reaction mixture, and (3) a mixture of 9,10-phenanthrenequinone and 2 equiv. of $B(C_6F_5)_3$.

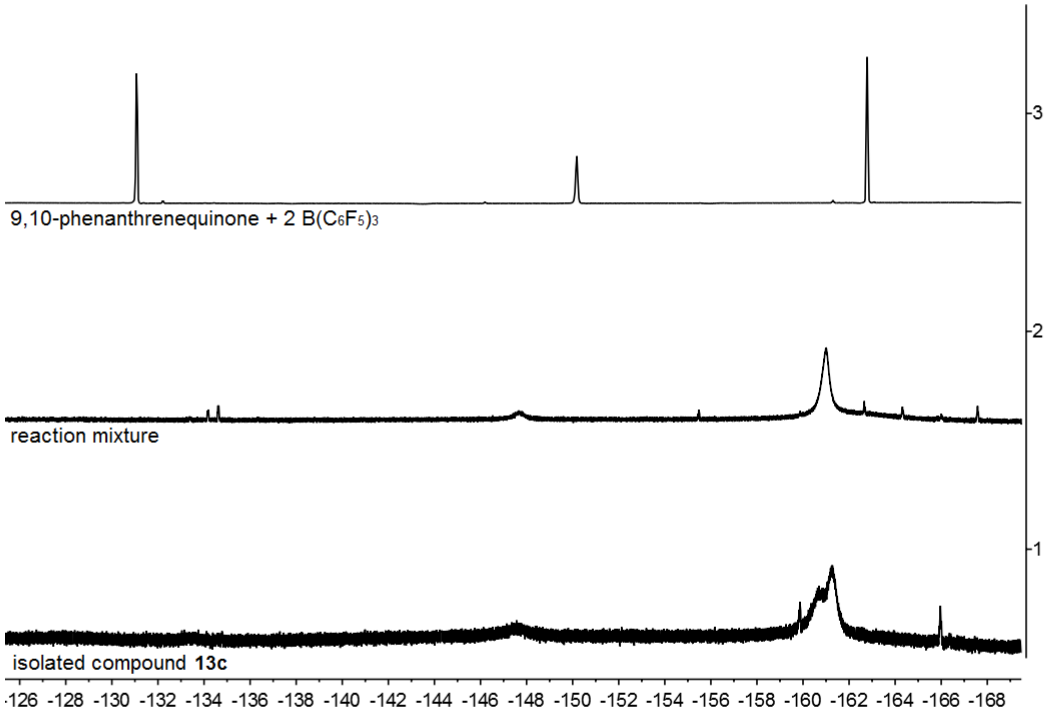

Figure S61. ^{19}F NMR (564 MHz, 299 K, CD_2Cl_2) spectra of (1) isolated compound **13c**, (2) reaction mixture, and (3) a mixture of 9,10-phenanthrenequinone and 2 equiv. of $B(C_6F_5)_3$.

M) Reaction of compound **8c** with Cp*$_2$Fe

Scheme S24

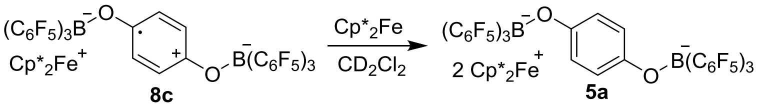

Compound **8c** (14.6 mg, 0,010) and decamethylferrocene (3.3 mg, 0.010 mmol) were dissolved in CD_2Cl_2 (0.5 mL) at room temperature. The resulting green solution was characterized by NMR experiments after 5 min.

Compound **5a** was observed as the main component in the reaction solution.

The NMR data of dianion in compound **5a** are comparable with those cited in the literature [L. Liu, L. L. Cao, Y. Shao, D. W. Stephan, *J. Am. Chem. Soc.* **2017**, *139*, 10062–10071].

NMR data of the reaction mixture:

^{1}H NMR (600 MHz, 299 K, CD_2Cl_2): δ ^{1}H: 6.81 (br s, PhO), -33.27 (br s, 30H, Cp*$_2$Fe$^+$).

^{19}F NMR (564 MHz, 299 K, CD_2Cl_2): δ ^{19}F: -134.1 (m, 2F, *o*-C_6F_5), -163.8 (t, $^3J_{FF}$ = 17.7 Hz, 1F, *p*-C_6F_5), -168.1 (m, 2F, *m*-C_6F_5).

^{11}B{^{1}H} NMR (192 MHz, 299 K, CD_2Cl_2): δ ^{11}B: -3.3.

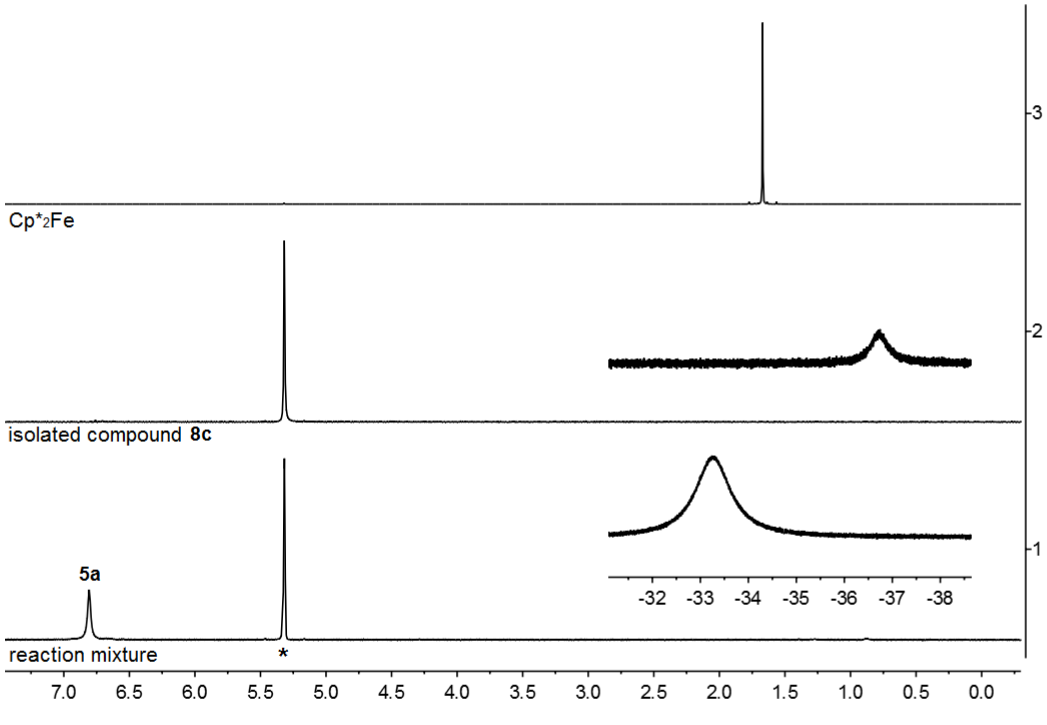

Figure S62. ^{1}H NMR (600 MHz, 299 K, CD_2Cl_2*) spectra of (1) reaction mixture, (2) isolated compound **8c** and (3) decamethylferrocene.

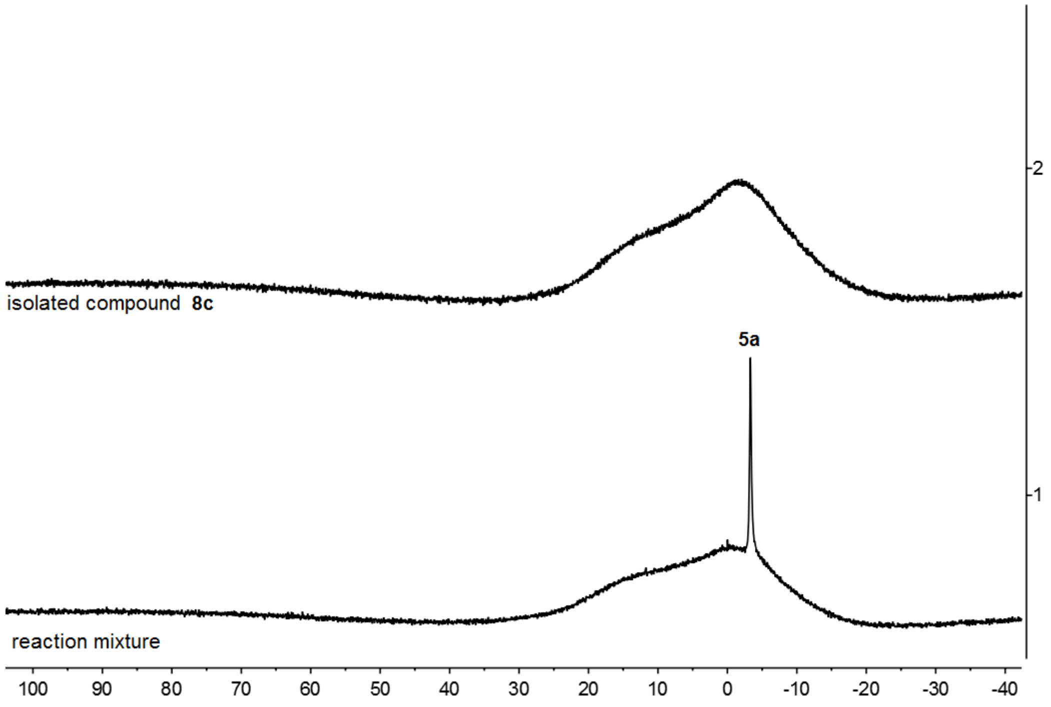

Figure S63. $^{11}B\{^{1}H\}$ NMR (192 MHz, 299 K, CD_2Cl_2) spectra of (1) reaction mixture and (2) isolated compound **8c**.

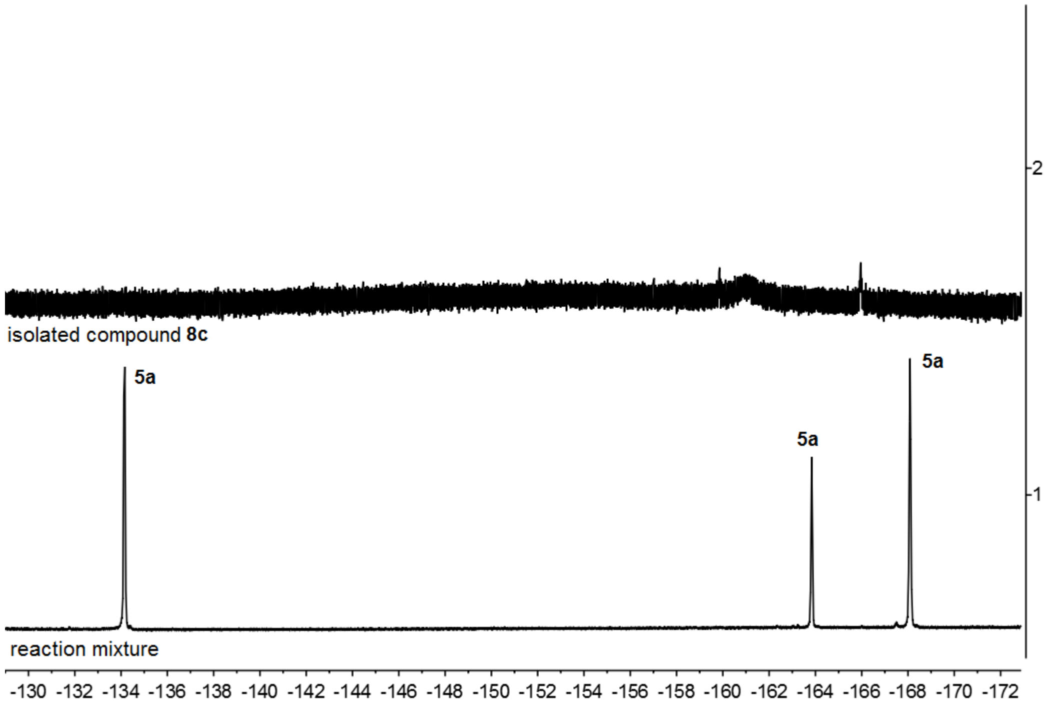

Figure S64. ^{19}F NMR (564 MHz, 299 K, CD_2Cl_2) spectra of (1) reaction mixture and (2) isolated compound **8c**.

N) Reaction of compound **11c** with Cp^*_2Fe

Experiment 1: (reaction of 9,10-anthraquinone, 2 $B(C_6F_5)_3$ and 2 Cp^*_2Fe)

Scheme S25

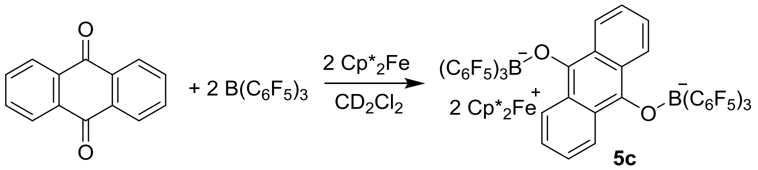

5c

Decamethylferrocene (32.6 mg, 0.100 mmol) was added to a mixture of 9,10-anthraquinone (10.4 mg, 0.050 mmol) and $B(C_6F_5)_3$ (51.1 mg, 0.100 mmol) in CD_2Cl_2 (0.5 mL). The green crystalline solid precipitated from the reaction mixture. The solution was removed by decantation and the part of the remaining crystals were used for X-ray structure analysis. The rest crystals were washed with pentane and dried in vacuo giving compound **5c** (55 mg, 0.029 mmol, 58%) as green crystalline material.

Decomp.: 260 °C

Anal. Calc. for $C_{90}H_{68}B_2F_{30}O_2Fe$: C, 57.35; H, 3.64. Found: C, 56.86; H, 3.42.

X-ray crystal structure analysis of 5c (erk8962): formula $C_{35}H_{21}B_2F_{20}NO \cdot CH_2Cl_2$, M = 958.07, colourless crystal, 0.18 x 0.07 x 0.03 mm, a = 12.5201(1), b = 12.2872(3), c = 25.1183(6) Å, β = 97.597(2)°, V = 3830.2(1) Å^3, ρ_{calc} = 1.661 gcm^{-3}, μ = 0.301 mm^{-1}, empirical absorption correction (0.947 ≤ T ≤ 0.991), Z = 4, monoclinic, space group $P2_1/c$ (No. 14), λ = 0.71073 Å, T = 173(2) K, ω and ϕ scans, 37236 reflections collected (±h, ±k, ±l), 6675 independent (R_{int} = 0.050) and 5289 observed reflections [I>2σ(I)], 664 refined parameters, R = 0.051, wR^2 = 0.126, max. (min.) residual electron density 0.40 (-0.37) e.Å^{-3}, the hydrogen atoms were calculated and refined as riding atoms.

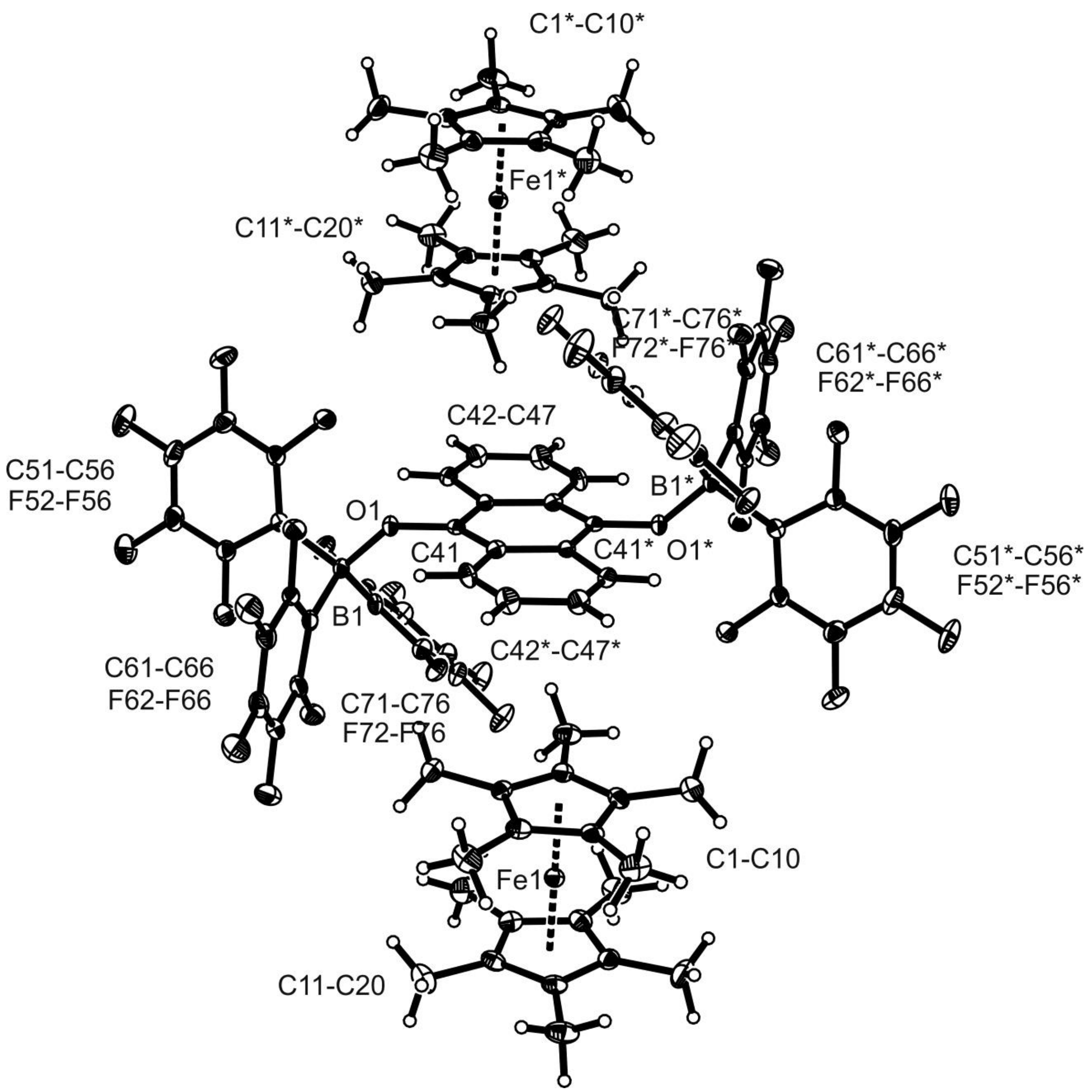

Figure S65. Crystal structure of compound **5c**. The half anion and the second cation are generated over inversion centre. Hydrogen atoms were omitted for clarity. (Thermals ellipsoids are shown with 30% probability.)

Experiment 2: (reaction of isolated compound **11c** with Cp^*_2Fe)

Scheme S26

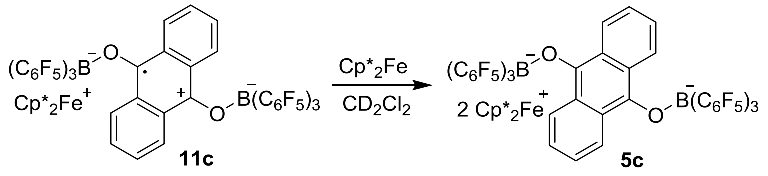

Compound **11c** (15.6 mg, 0,010) and decamethylferrocene (3.3 mg, 0.010 mmol) were mixed in CD_2Cl_2 (0.5 mL) at room temperature. The green crystalline solid precipitated from the reaction mixture. The obtained crystals (ca. 10 mg) were used for X-ray structure analysis.
[Comment: the cell parameters of these crystals are comparable to those obtained in exp.1]

O) Reaction of compound **12c** with Cp^*_2Fe

Scheme S27

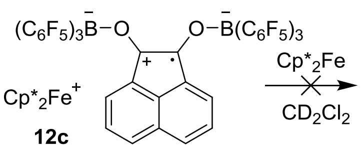

Compound **12c** (15.3 mg, 0,010) and decamethylferrocene (3.3 mg, 0.010 mmol) were dissolved in CD_2Cl_2 (0.5 mL) at room temperature. The resulting green solution was characterized by NMR experiments.

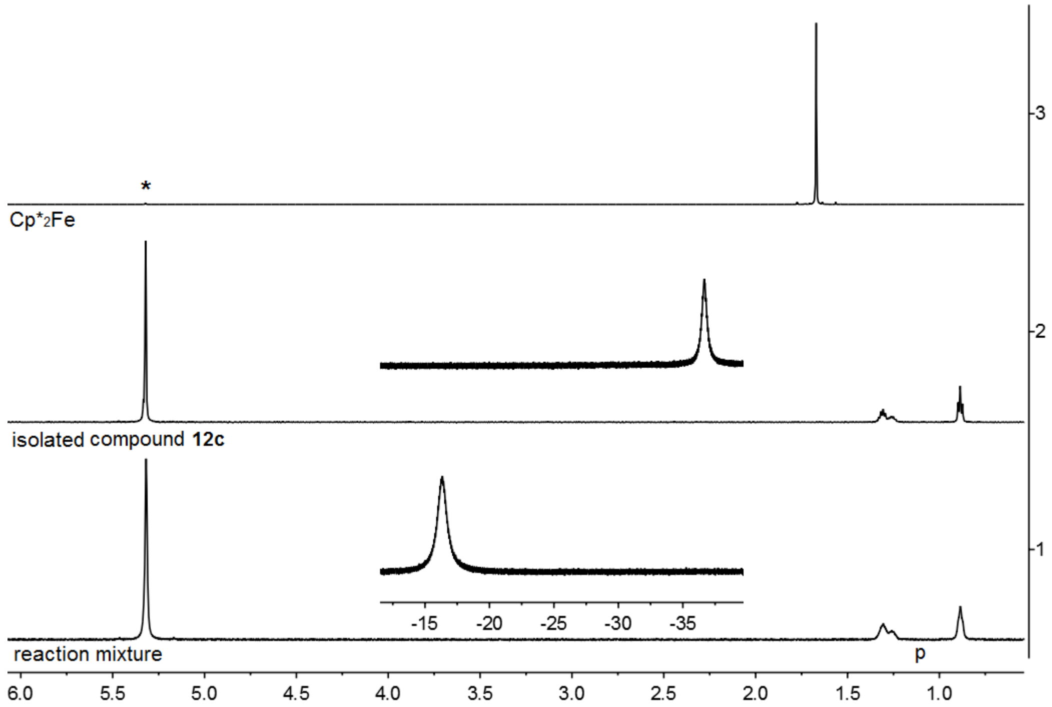

Figure S66. ^1H NMR (600 MHz, 299 K, $CD_2Cl_2^*$) spectra of (1) reaction mixture, (2) isolated compound **12c** and (3) decamethylferrocene.[p: pentane]

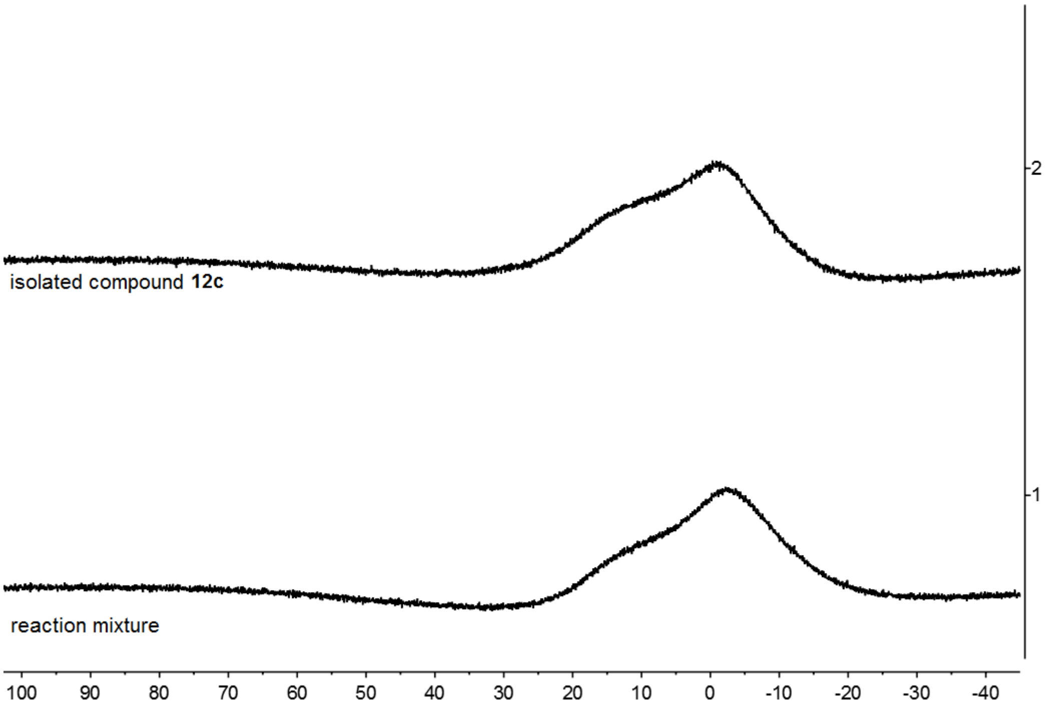

Figure S67. $^{11}B\{^{1}H\}$ NMR (192 MHz, 299 K, CD_2Cl_2) spectra of (1) reaction mixture and (2) isolated compound **12c**.

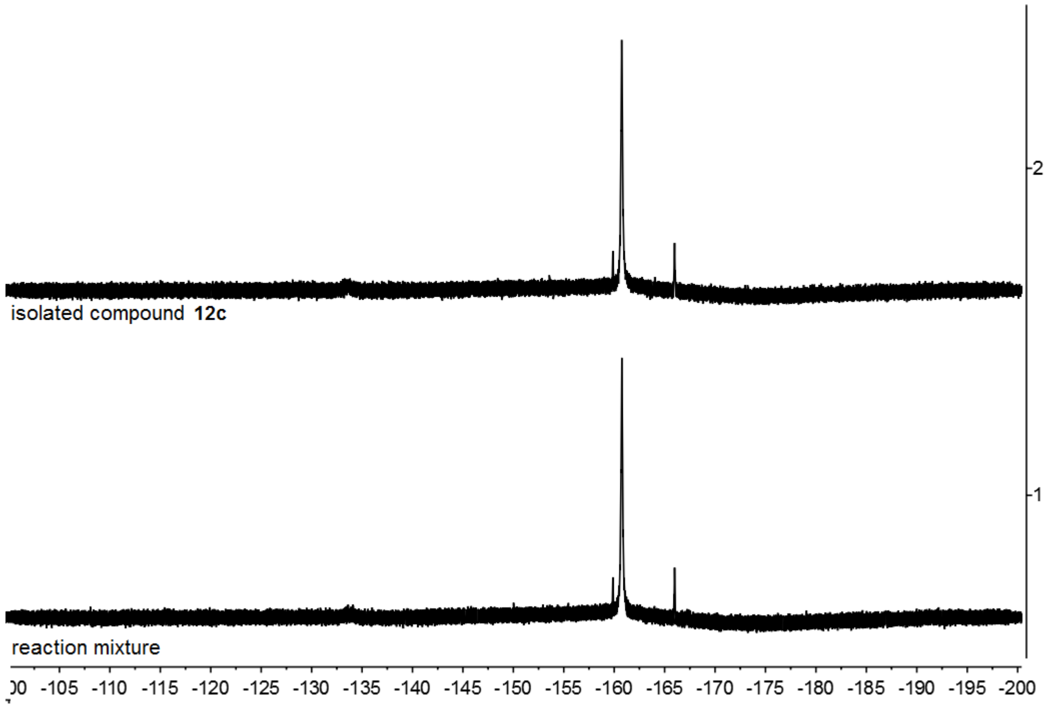

Figure S68. ^{19}F NMR (564 MHz, 299 K, CD_2Cl_2) spectra of (1) reaction mixture and (2) isolated compound **12c**.

P) Reaction of compound **13c** with $Cp*_2Fe$

Experiment 1: (reaction of 9,10-phenanthrenequinone, 2 $B(C_6F_5)_3$ and 2 $Cp*_2Fe$)

Scheme S28

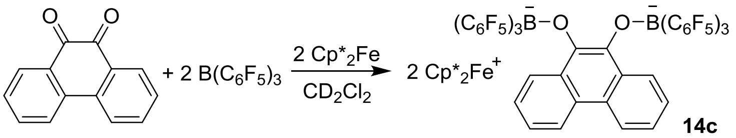

14c

Decamethylferrocene (65.2 mg, 0.200 mmol) was added to a mixture of 9,10-phenanthrenequinone (20.8 mg, 0.100 mmol) and $B(C_6F_5)_3$ (102 mg, 0.200 mmol) in CD_2Cl_2 (1.0 mL). The resulting green solution was then layered with pentane (1.0 mL) and stored at room temperature for 24 h. Then the green crystalline material precipitated. The solution was removed by decantation and the part of the remaining crystals were used for X-ray structure analysis. The remaining crystals were washed with pentane and dried in vacuo giving compound **14c** (179 mg, 0.095 mmol, 95%) as green crystalline material.

Decomp.:163 ^oC

Anal. Calc. for $C_{90}H_{68}B_2F_{30}O_2Fe$: C, 57.35; H, 3.64. Found: C, 56.94; H, 3.90.

NMR data of isolated compound **14c**: (tentatively assigned)

^{1}H NMR (600 MHz, 299 K, CD_2Cl_2): δ ^{1}H: 8.84, 7.98, 7.57 (each br s, 8H, dianion), –36.5 (br s, 20H, $Cp*_2Fe^+$).

^{1}H NMR (600 MHz, 233 K, CD_2Cl_2): δ ^{1}H: 9.21, 8.06, 8.01, 7.78 (each br s, 1:1:1:1, dianion), –50.3 (br s, 20H, $Cp*_2Fe^+$).

^{13}C{^{1}H} NMR (151 MHz, 233 K, CD_2Cl_2): δ ^{13}C: 144.1, 134.0, 125.6, 123.8, 123.3, 121.8, 121.0 (each s, dianion). [C_6F_5 not listed]

^{19}F NMR (564 MHz, 299 K, CD_2Cl_2): δ ^{19}F: –127.7, –132.5, –134.9 (each br s, 2F, o-C_6F_5), –165.8 (br s, 1F, p-C_6F_5), –169.6, –171.2 (each br s, 2 F, m-C_6F_5).

^{19}F NMR (564 MHz, 233 K, CD_2Cl_2): δ ^{19}F: –128.2, –131.1, –133.7, –134.1, –134.4, –135.8 (each br m, each 1F, o-C_6F_5), –164.2, –165.2, –166.3 (each br s, each 1F, p-C_6F_5), –167.5, –168.4, –169.6, –170.5, –170.9, –171.6 (each br s, 2 F, m-C_6F_5).

^{11}B{^{1}H} NMR (192 MHz, 299 K, CD_2Cl_2): δ ^{11}B: –2.4 ($\nu_{1/2} \approx$ 180 Hz).

^{11}B{^{1}H} NMR (192 MHz, 233 K, CD_2Cl_2): δ ^{11}B: –2.7 ($\nu_{1/2} \approx$ 250 Hz).

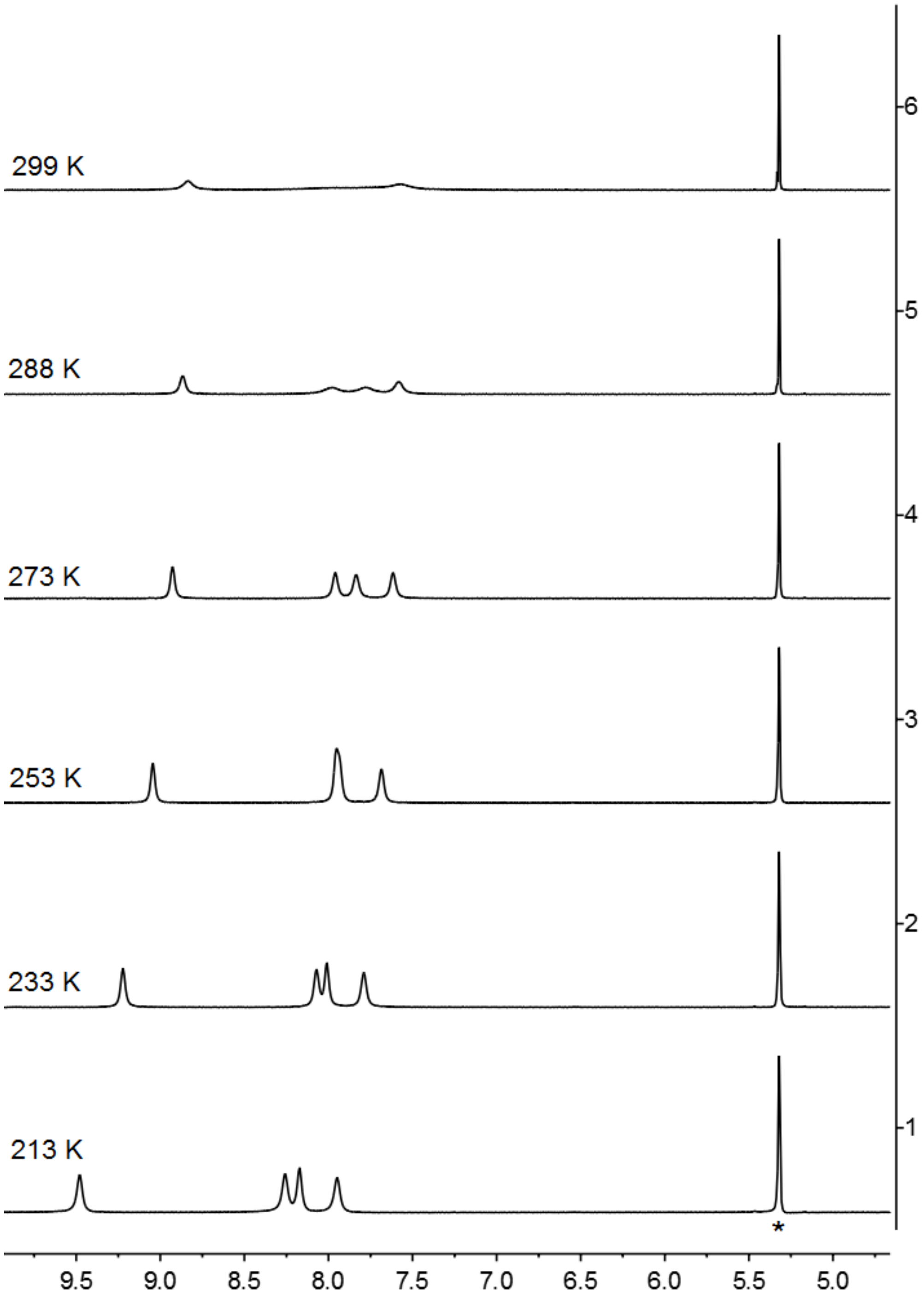

Figure S69. ^{1}H NMR (600 MHz, CD_2Cl_2*) spectra of isolated compound **14c** at variable temperature.

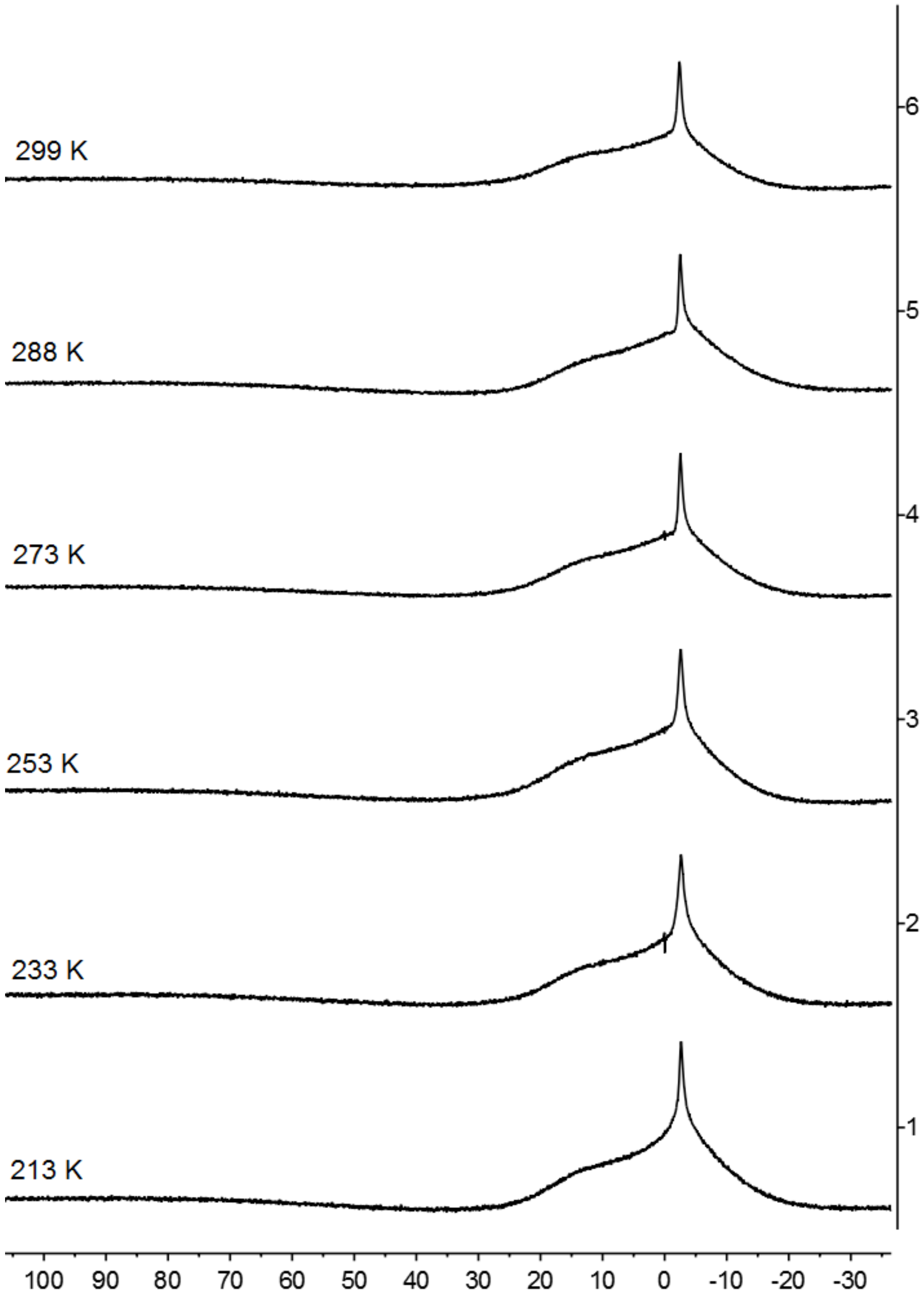

Figure S70. $^{11}B\{^{1}H\}$ NMR (192 MHz, CD_2Cl_2) spectra of isolated compound **14c** at variable temperature.

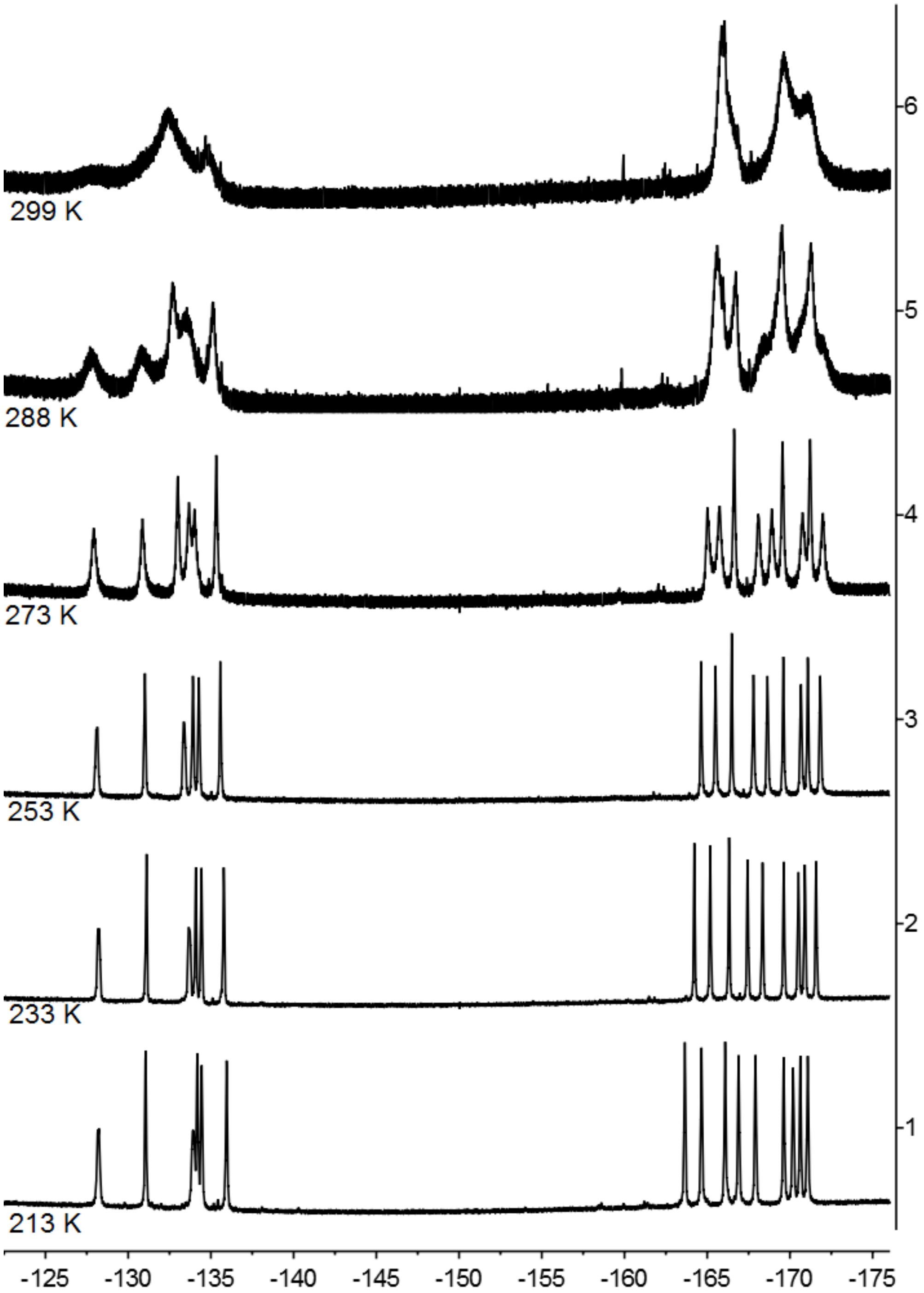

Figure S71. ^{19}F NMR (564 MHz, CD_2Cl_2) spectra of isolated compound **14c** at variable temperature.

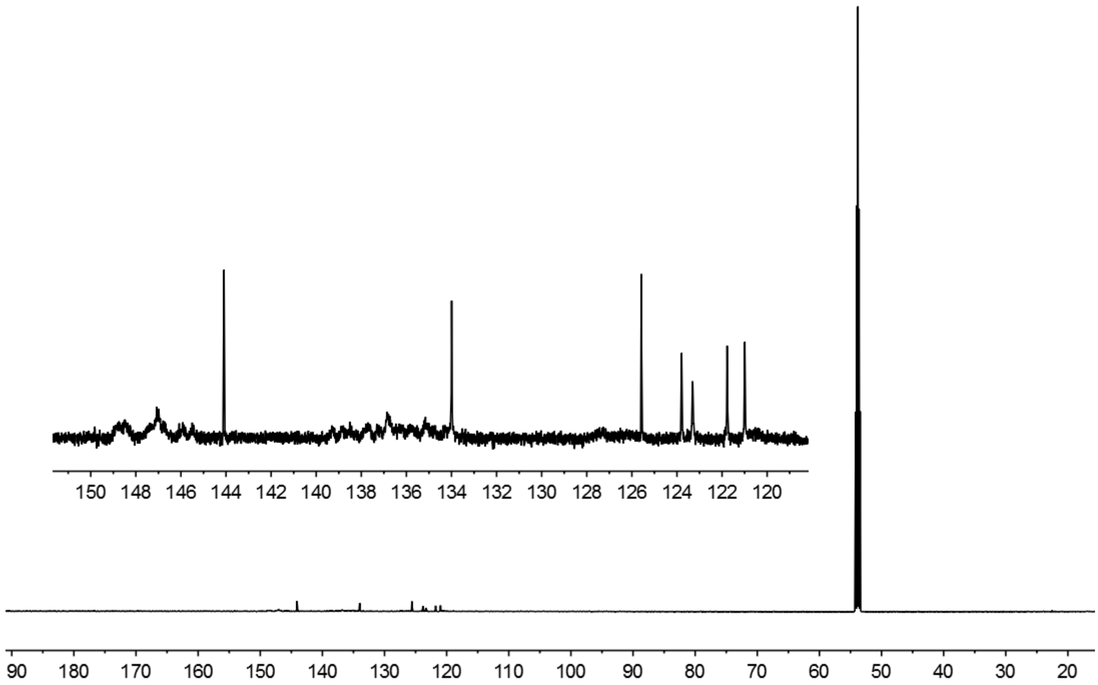

Figure S72. $^{13}C\{^{1}H\}$ NMR (151 MHz, 233K, CD_2Cl_2) spectra of isolated compound **14c**.

X-ray crystal structure analysis of 14c (erk8998): A green plate-like specimen of $C_{90}H_{68}B_2F_{30}Fe_2O_2$, approximate dimensions 0.030 mm x 0.160 mm x 0.180 mm, was used for the X-ray crystallographic analysis. The X-ray intensity data were measured. A total of 2183 frames were collected. The total exposure time was 32.07 hours. The frames were integrated with the Bruker SAINT software package using a wide-frame algorithm. The integration of the data using a monoclinic unit cell yielded a total of 157095 reflections to a maximum θ angle of 65.08° (0.85 Å resolution), of which 14672 were independent (average redundancy 10.707, completeness = 99.9%, R_{int} = 20.01%, R_{sig} = 10.06%) and 9269 (63.17%) were greater than 2σ(F^2). The final cell constants of a = 26.8002(8) Å, b = 12.9058(4) Å, c = 27.8292(8) Å, β = 116.473(2)°, volume = 8616.2(5) $Å^3$, are based upon the refinement of the XYZ-centroids of 7056 reflections above 20 σ(I) with 7.369° < 2θ < 113.0°. Data were corrected for absorption effects using the multi-scan method (SADABS). The ratio of minimum to maximum apparent transmission was 0.759. The calculated minimum and maximum transmission coefficients (based on crystal size) are 0.5550 and 0.8970. The structure was solved and refined using the Bruker SHELXTL Software Package, using the space group $P2_1/n$, with Z = 4 for the formula unit, $C_{90}H_{68}B_2F_{30}Fe_2O_2$. The final anisotropic full-matrix least-squares refinement on F^2 with 1197 variables converged at R1 = 5.99%, for the observed data and wR2 = 16.46% for all data. The goodness-of-fit was 1.018. The largest peak in the final difference electron density synthesis was 0.469 $e^-/Å^3$ and the largest hole was -0.358 $e^-/Å^3$ with an RMS deviation of 0.080 $e^-/Å^3$. On the basis of the final model, the calculated density was 1.453 g/cm^3 and F(000), 3824 e^-.

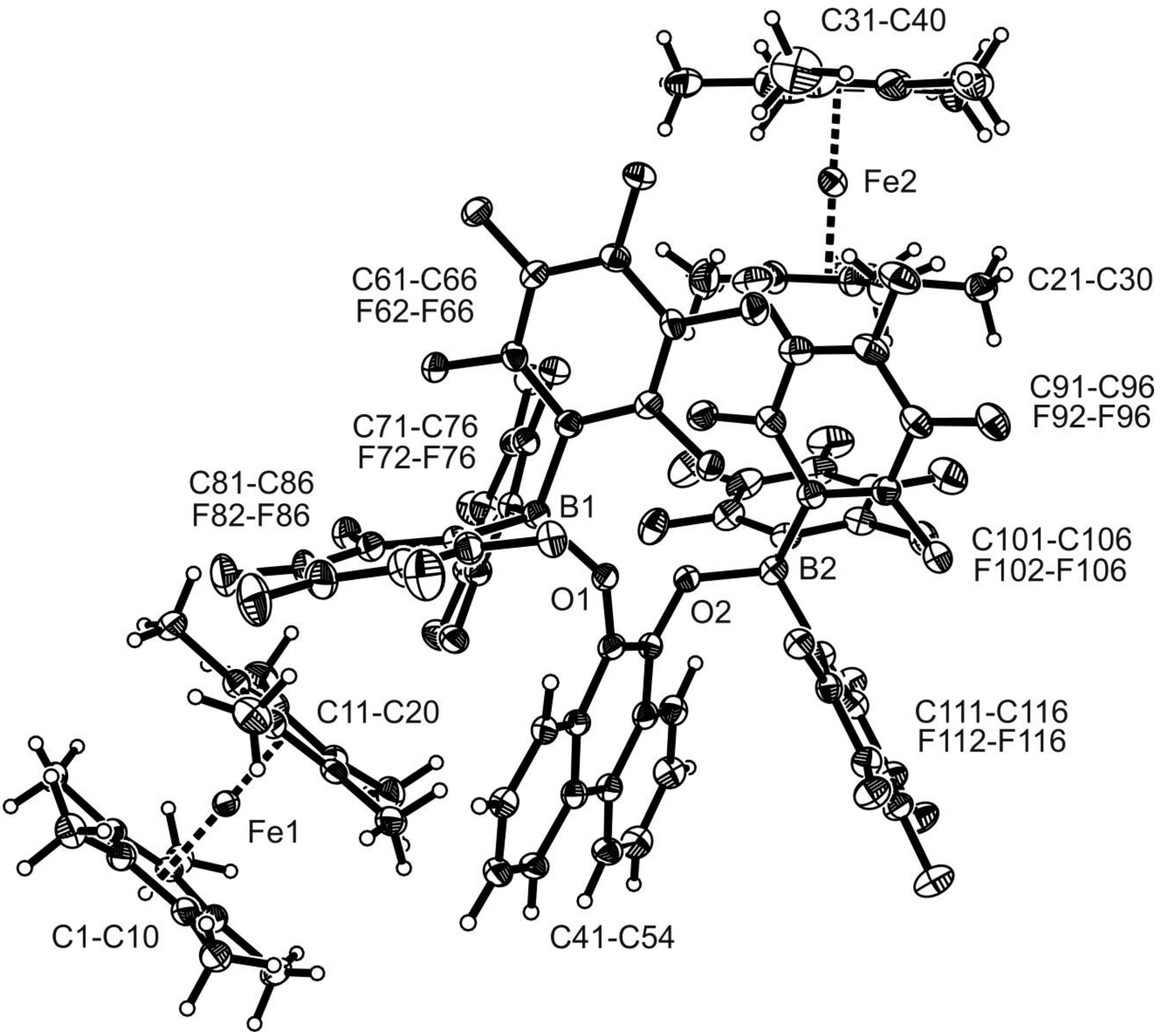

Figure S73. Crystal structure of compound **14c**. (Thermals ellipsoids are shown with 30% probability.)

Experiment 2: (reaction of isolated compound **13c** with Cp^*_2Fe)

Scheme S29

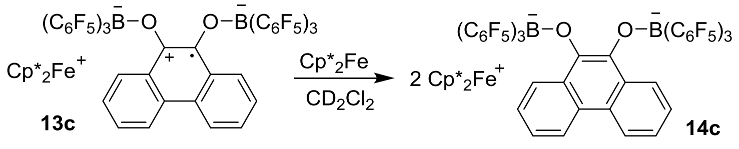

Compound **13c** (15.6 mg, 0.010 mmol) and decamethylferrocene (3.3 mg, 0.010 mmol) were mixed in CD_2Cl_2 (0.5 mL) at room temperature. The resulting green solution was characterized by NMR experiments.

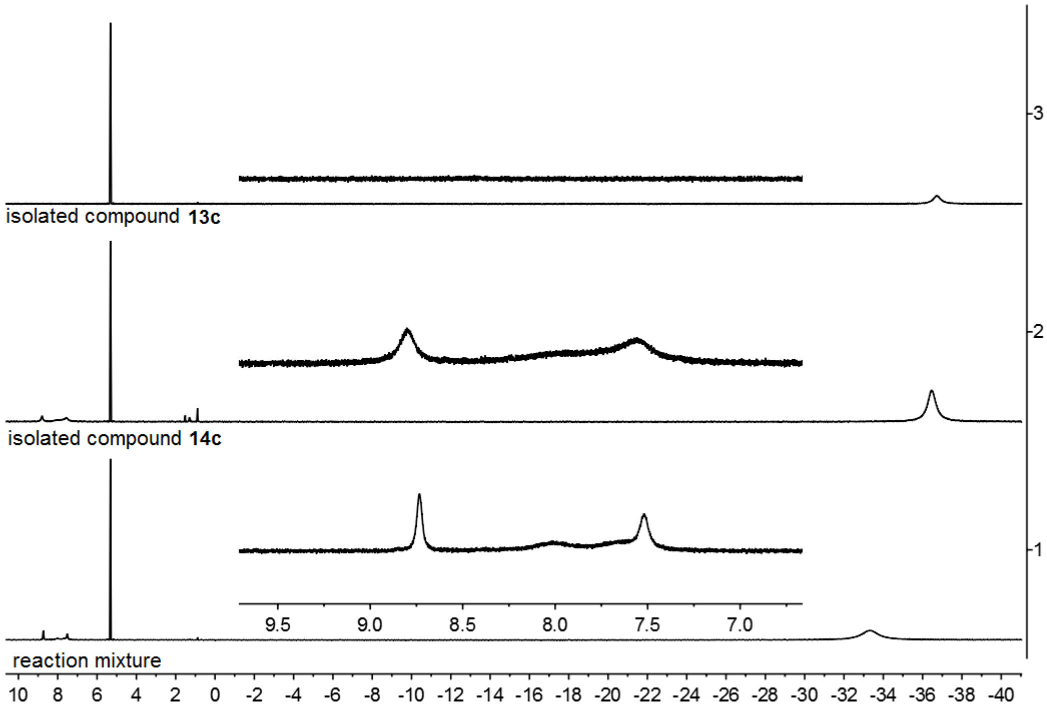

Figure S74. ^{1}H NMR (600 MHz, 299 K, CD_2Cl_2) spectra of (1) the reaction mixture, (2) isolated compound **14c** and (3) isolated compound **13c**.

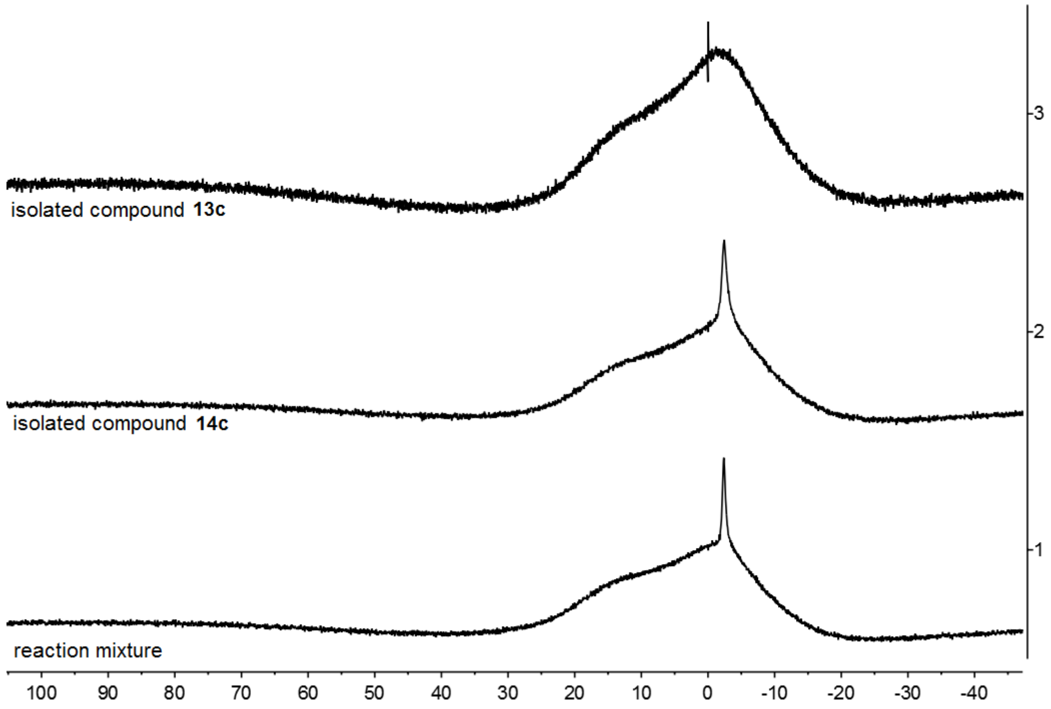

Figure S75. $^{11}B\{^{1}H\}$ NMR (192 MHz, 299 K, CD_2Cl_2) spectra of (1) he reaction mixture, (2) isolated compound **14c** and (3) isolated compound **13c**.

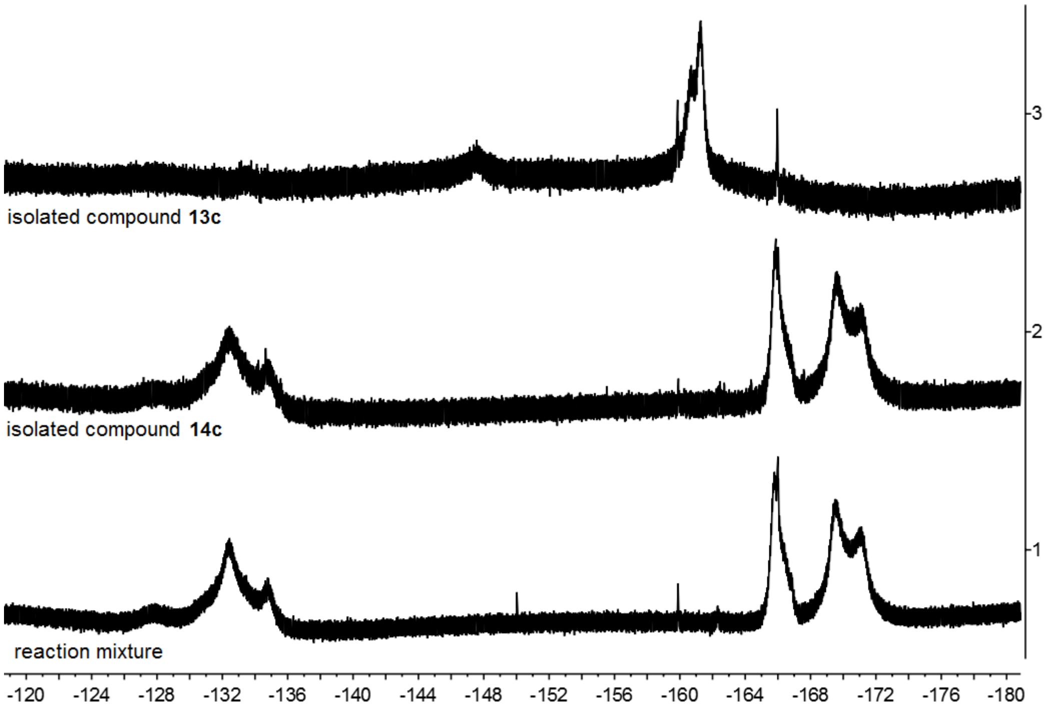

Figure S76. ^{19}F NMR (564 MHz, 299 K, CD_2Cl_2) spectra of (1) the reaction mixture, (2) isolated compound **14c** and (3) isolated compound **13c**.

Q) Reaction of compound **8a** with THF and DMSO, respectively

Experiment 1: (NMR scale, reaction of compound **8a** with THF in CD_2Cl_2)

Scheme S30

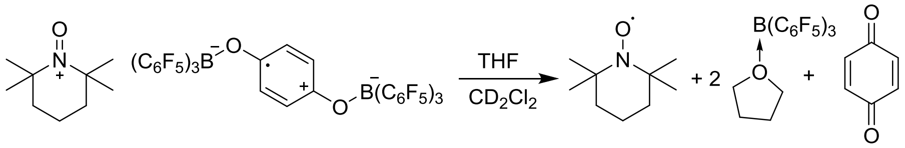

One drop of THF was added to a solution of compound **8a** (30 mg, 0.023 mmol) in CD_2Cl_2 (0.5 mL) and the resulting solution was characterized by NMR and EPR experiments.

[Comment: The THF/B(C_6F_5)$_3$ adduct and p-benzoquinone was observed by NMR experiments. The TEMPO radical was observed as a product by EPR spectroscopy (see EPR section, page S108, Figure S124).]

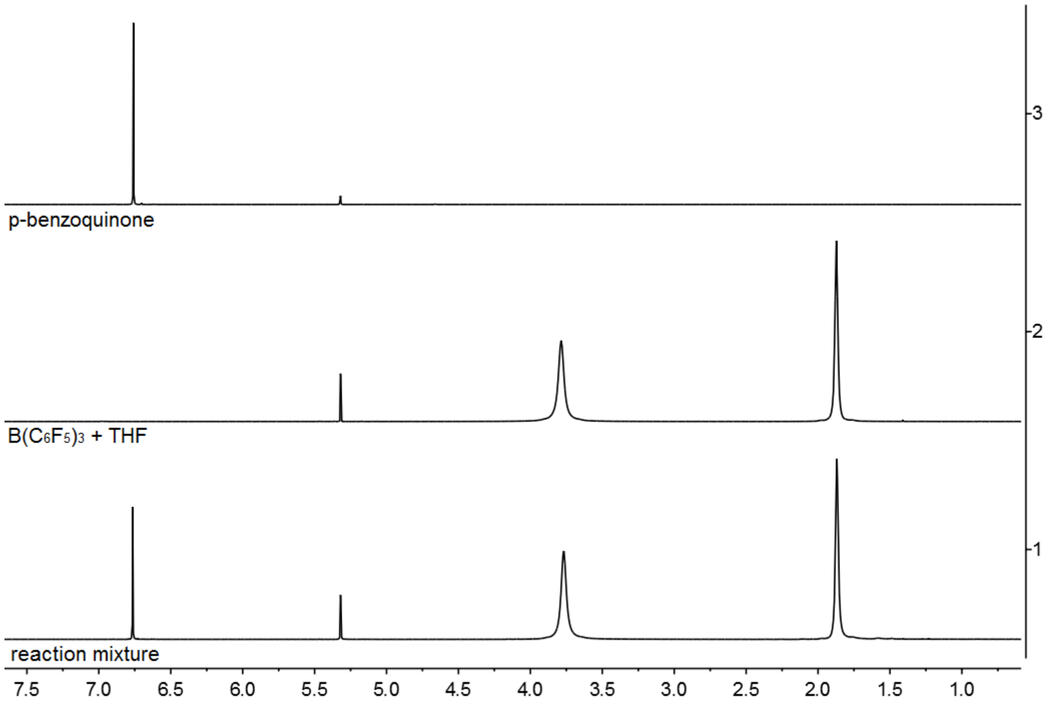

Figure S77. ^{1}H NMR (600 MHz, 299 K, CD_2Cl_2) spectra of (1) the reaction mixture of experiment 1, (2) the mixture of B(C_6F_5)$_3$ and one drop of THF and (3) p-benzoquinone for comparison .

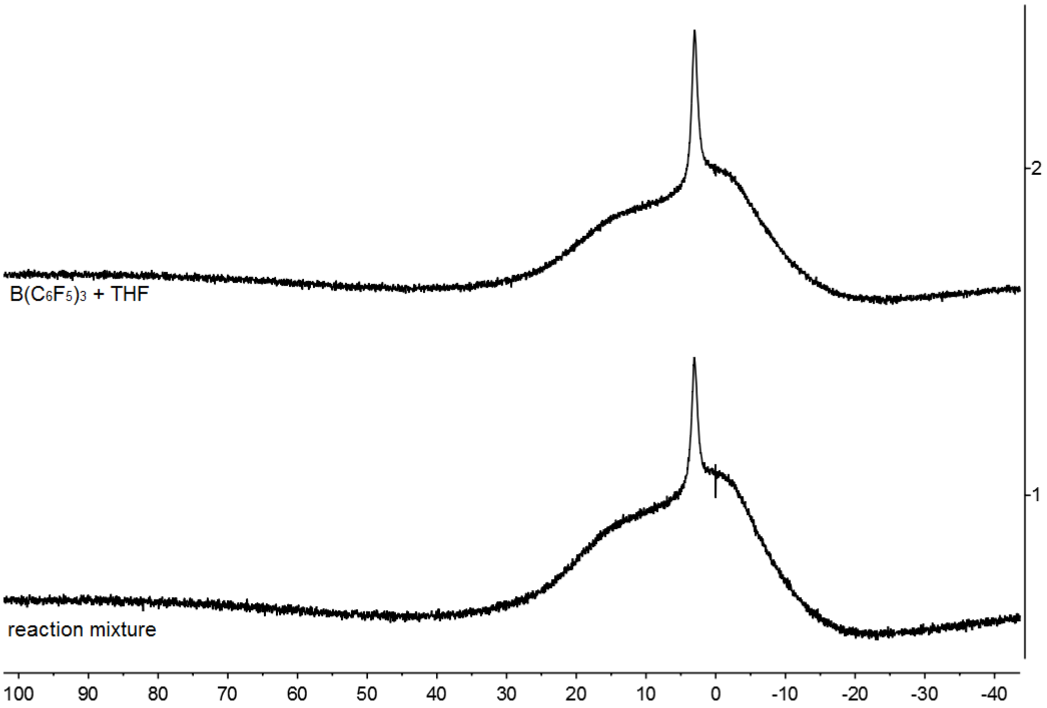

Figure S78. $^{11}B\{^1H\}$ NMR (192 MHz, 299 K, CD_2Cl_2) spectra of (1) he reaction mixture of experiment 1 and (2) the mixture of $B(C_6F_5)_3$ and one drop of THF for comparison .

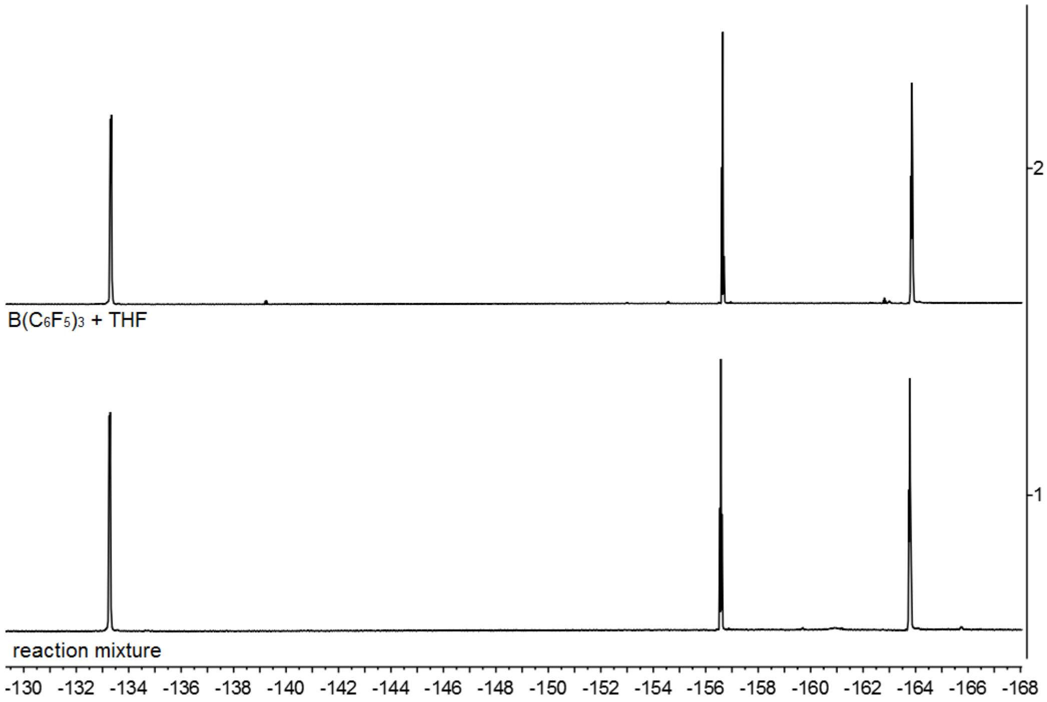

Figure S79. ^{19}F NMR (564 MHz, 299 K, CD_2Cl_2) spectra of (1) the reaction mixture of experiment 1 and (2) the mixture of $B(C_6F_5)_3$ and one drop of THF for comparison .

Experiment 2: (NMR scale, reaction of compound **8a** with DMSO in CD_2Cl_2)

Scheme S31

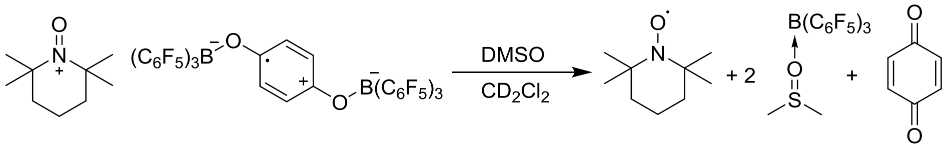

One drop of THF was added to a solution of compound **8a** (30 mg, 0.023 mmol) in CD_2Cl_2 (0.5 mL) and the resulting solution was characterized by NMR and EPR experiments.

[Comment: The DMSO/B(C_6F_5)$_3$ adduct and p-benzoquinone were observed by NMR experiments. The TEMPO radical was observed as a product by EPR spectroscopy (see EPR section, page S108, Figure S124).]

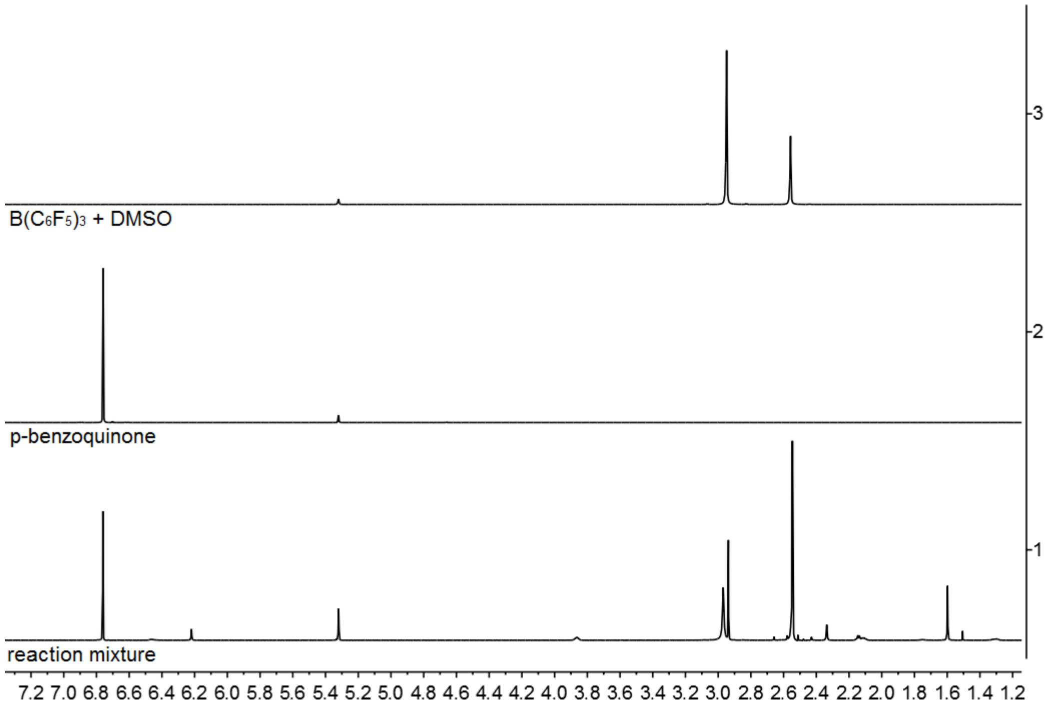

Figure S80. ^1H NMR (600 MHz, 299 K, CD_2Cl_2) spectra of (1) the reaction mixture of experiment 2, (2) p-benzoquinone and (3) the mixture of B(C_6F_5)$_3$ and one drop of DMSO for comparison.

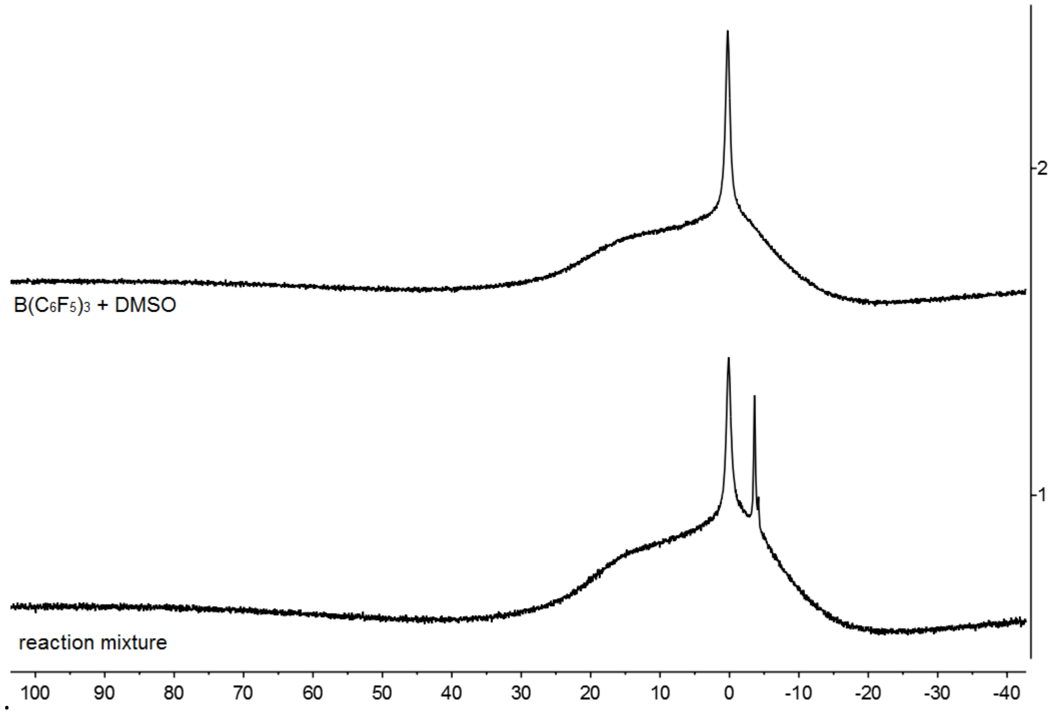

Figure S81. $^{11}B\{^1H\}$ NMR (192 MHz, 299 K, CD_2Cl_2) spectra of (1) the reaction mixture of experiment 2 and (2) the mixture of $B(C_6F_5)_3$ and one drop of DMSO for comparison .

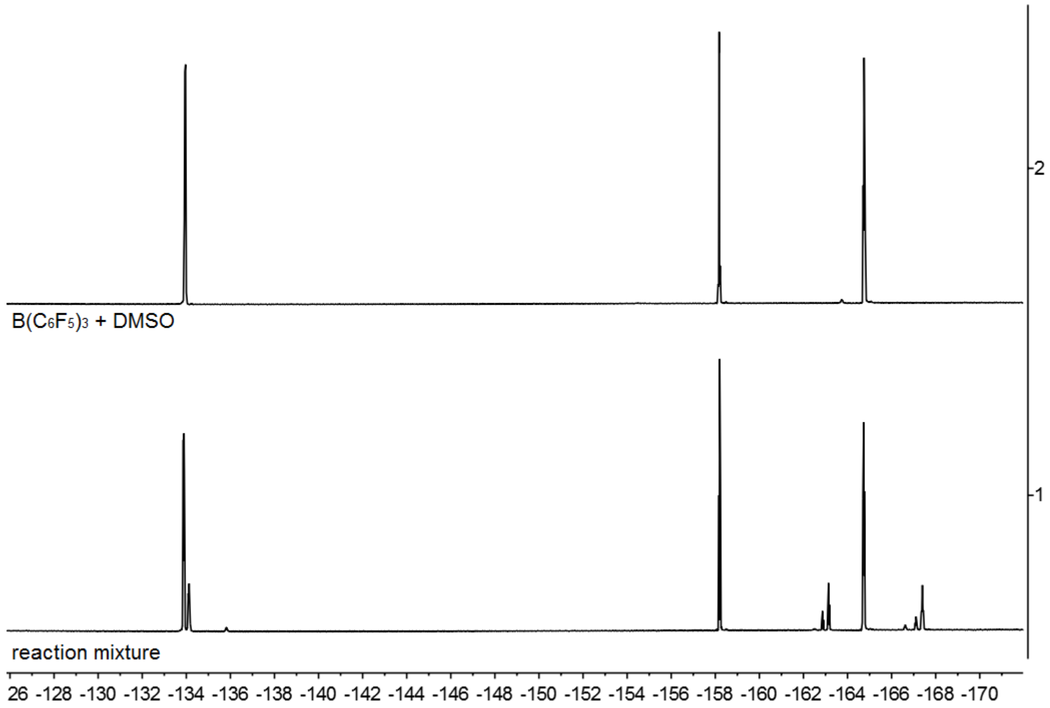

Figure S82. ^{19}F NMR (564 MHz, 299 K, CD_2Cl_2) spectra of (1) the reaction mixture of experiment 2 and (2) the mixture of $B(C_6F_5)_3$ and one drop of DMSO for comparison .

R) Reaction of compound **8b** with THF and DMSO, respectively

Experiment 1: (NMR scale, reaction of compound **6**with THF in CD_2Cl_2)

Scheme S32

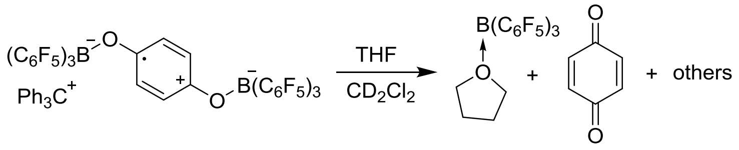

One drop of THF was added to a solution of compound **8b** (30 mg, 0.023 mmol) in CD_2Cl_2 (0.5 mL) and the resulting solution was characterized by NMR experiments.

[Comment: The THF/B(C_6F_5)$_3$ adduct, p-benzoquinone and triphenylmethane were observed by NMR experiments.]

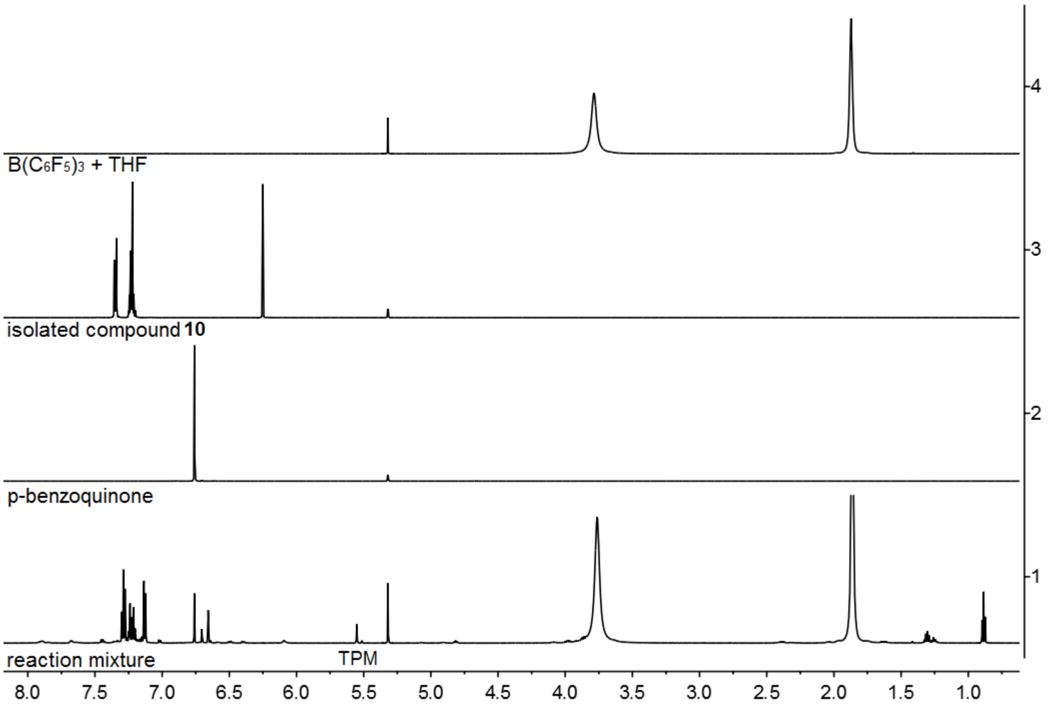

Figure S83. ^{1}H NMR (600 MHz, 299 K, CD_2Cl_2) spectra of (1) the reaction mixture of experiment 1, (2) p-benzoquinone, (3) isolated compound **10** and (4) the mixture of B(C_6F_5)$_3$ and one drop of THF for comparison . [TPM: triphenylmethane]

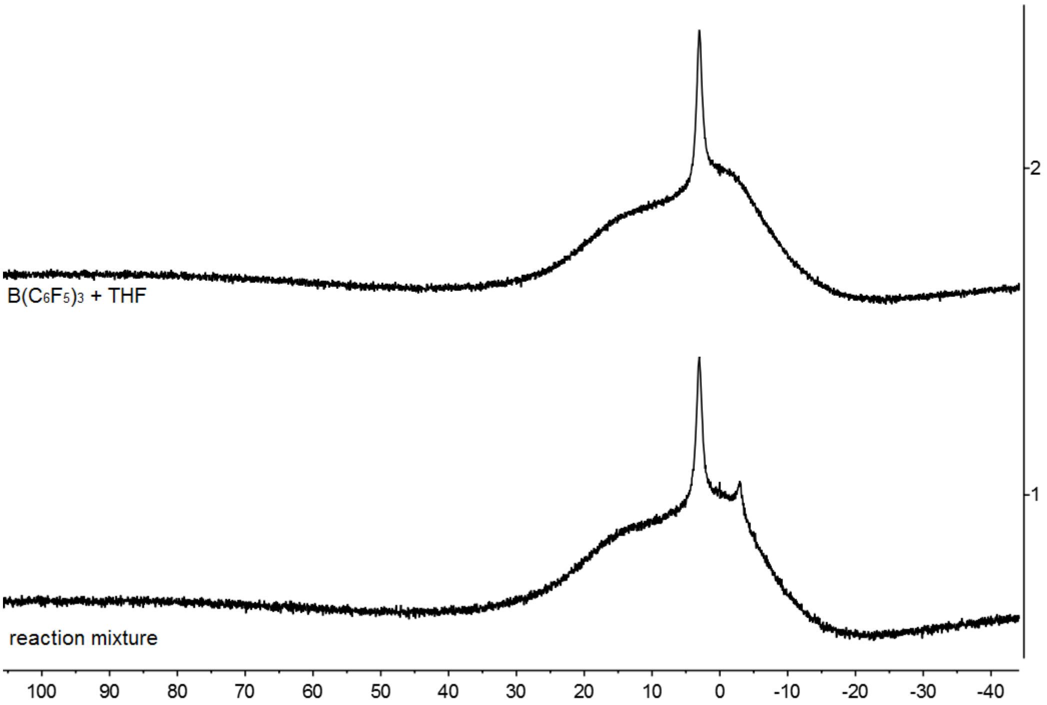

Figure S84. $^{11}B\{^{1}H\}$ NMR (192 MHz, 299 K, CD_2Cl_2) spectra of (1) the reaction mixture of experiment 1 and (2) the mixture of $B(C_6F_5)_3$ and one drop of THF for comparison .

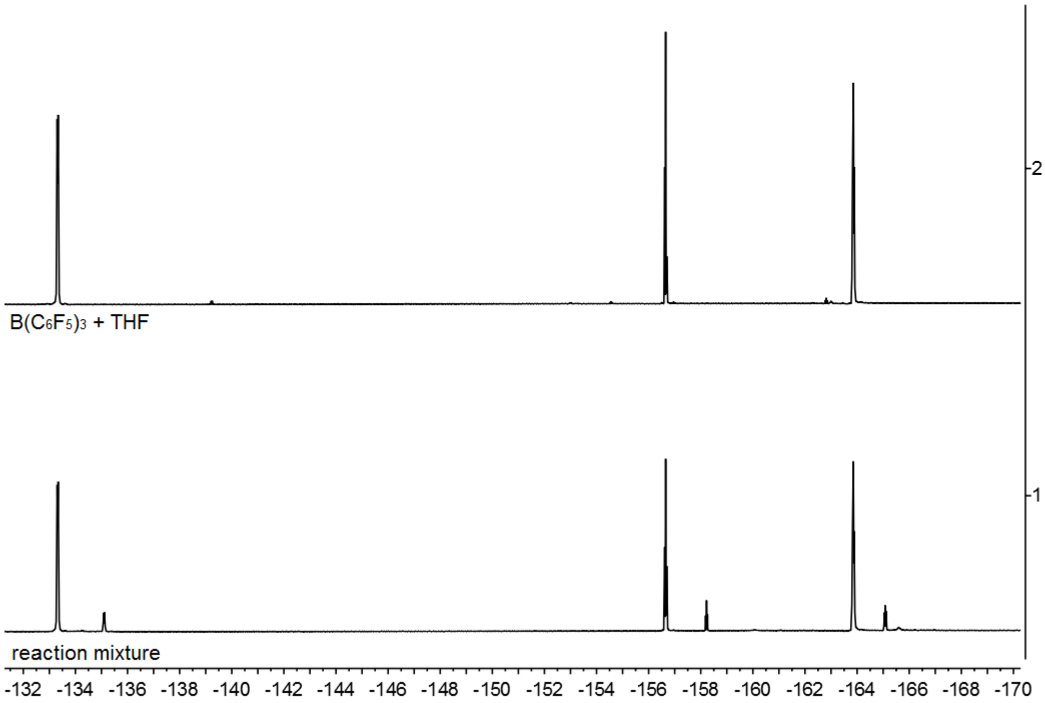

Figure S85. ^{19}F NMR (564 MHz, 299 K, CD_2Cl_2) spectra of (1) the reaction mixture of experiment 1 and (2) the mixture of $B(C_6F_5)_3$ and one drop of THF for comparison .

Experiment 2: (NMR scale, reaction of compound **8b** with DMSO in CD_2Cl_2)

Scheme S33

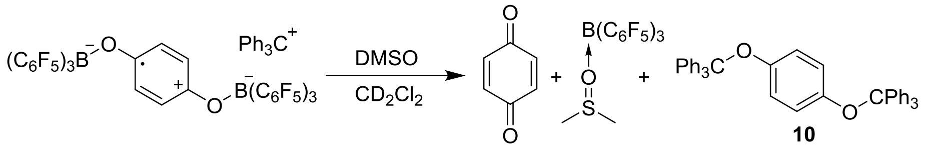

One drop of DMSO was added to a solution of compound **8b** (30 mg, 0.023 mmol) in CD_2Cl_2 (0.5 mL) and the resulting solution was characterized by NMR and EPR experiments.

[Comment: The DMSO/B(C_6F_5)$_3$ adduct, p-benzoquinone and compound ***10*** *were observed by NMR experiments.]*

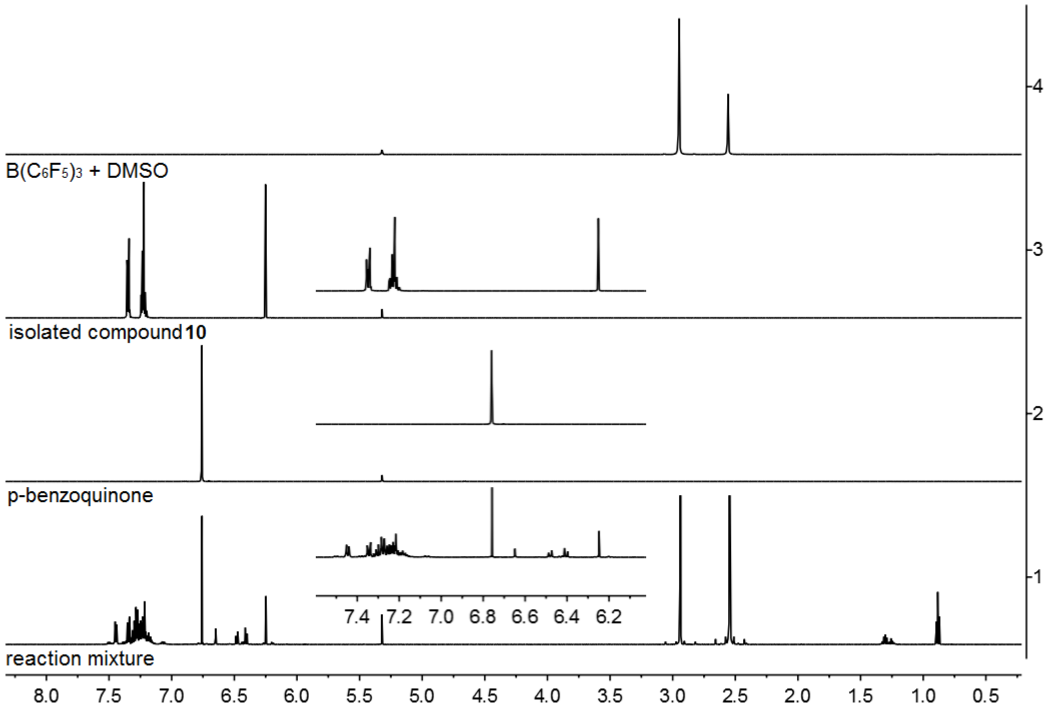

Figure S86. ^{1}H NMR (600 MHz, 299 K, CD_2Cl_2) spectra of (1) the reaction mixture of experiment 2, (2) p-benzoquinone, (3) isolated compound **10** and (4) the mixture of B(C_6F_5)$_3$ and one drop of DMSO for comparison.

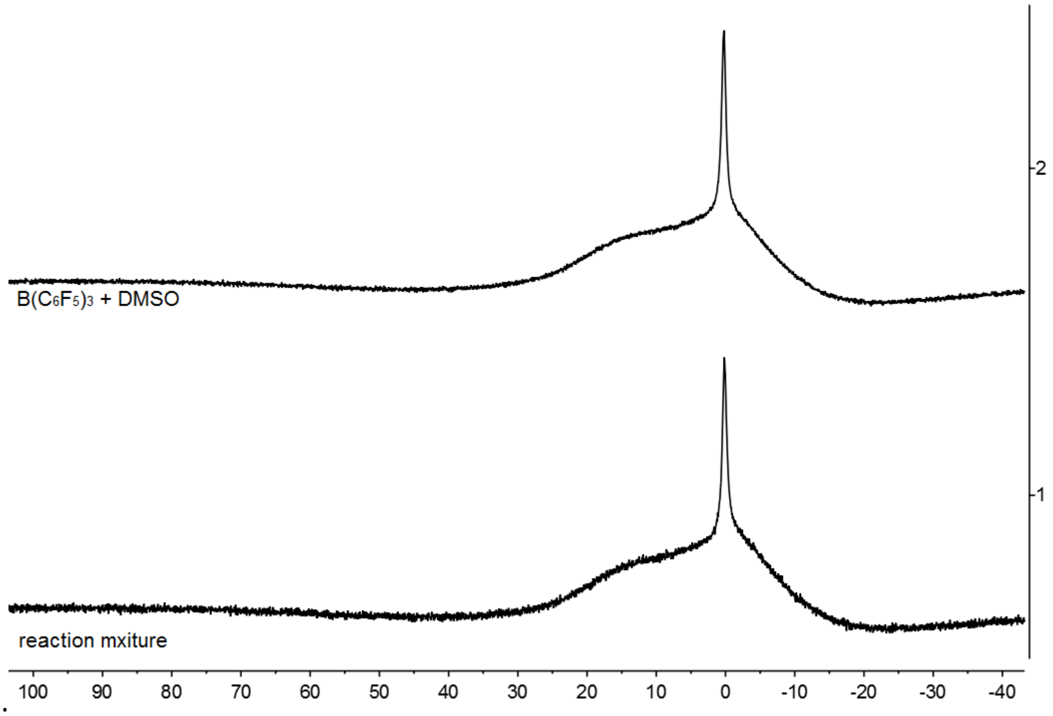

Figure S87. $^{11}B\{^{1}H\}$ NMR (192 MHz, 299 K, CD_2Cl_2) spectra of (1) the reaction mixture of experiment 2 and (2) the mixture of $B(C_6F_5)_3$ and one drop of DMSO for comparison .

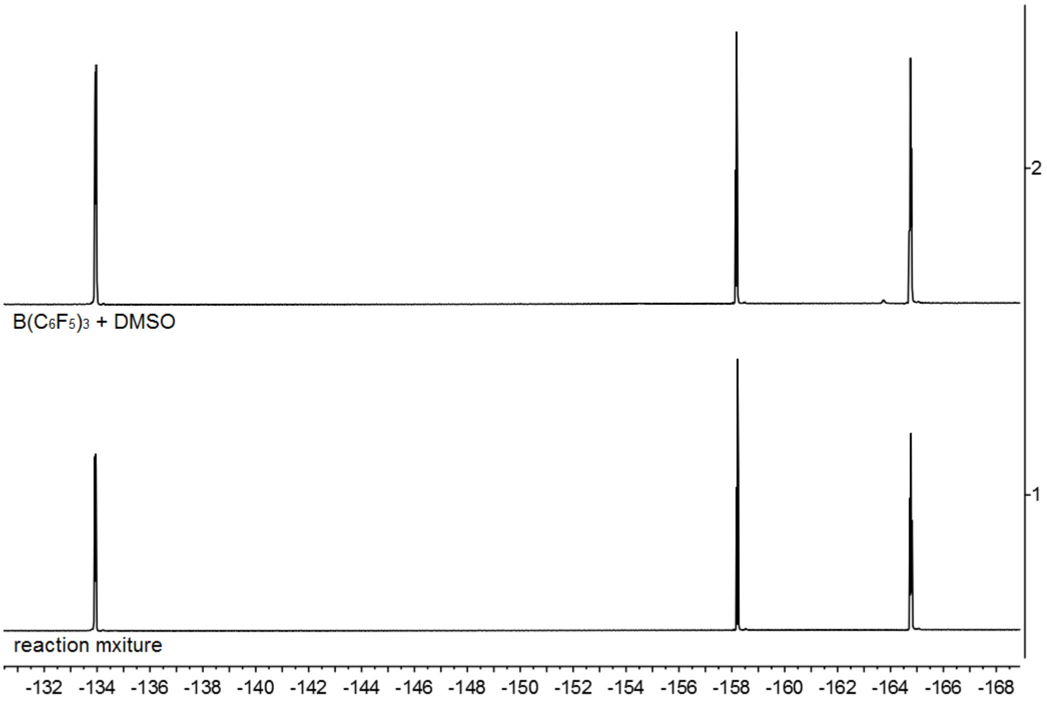

Figure S88. ^{19}F NMR (564 MHz, 299 K, CD_2Cl_2) spectra of (1) the reaction mixture of experiment 2 and (2) the mixture of $B(C_6F_5)_3$ and one drop of DMSO for comparison .

S) Reaction of compound **8c** with THF and DMSO, respectively

Experiment 1: (NMR scale, reaction of compound **8c** with THF in CD_2Cl_2)

Scheme S34

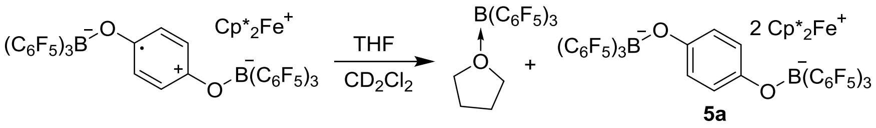

One drop of THF was added to a solution of compound **8c** (10 mg, 0.007 mmol) in CD_2Cl_2 (0.5 mL) and the resulting suspension was characterized by NMR experiments after 72 h at room temperature.

[Comment: The reaction was very slow. The THF/$B(C_6F_5)_3$ adduct and trace amounts of compound **5a** were observed by NMR experiments. The NMR data of dianion part of compound **5a** in the reaction mixture are comparable with those cited in the literature [L. Liu, L. L. Cao, Y. Shao, D. W. Stephan, *J. Am. Chem. Soc.* **2017**, *139*, 10062–10071].

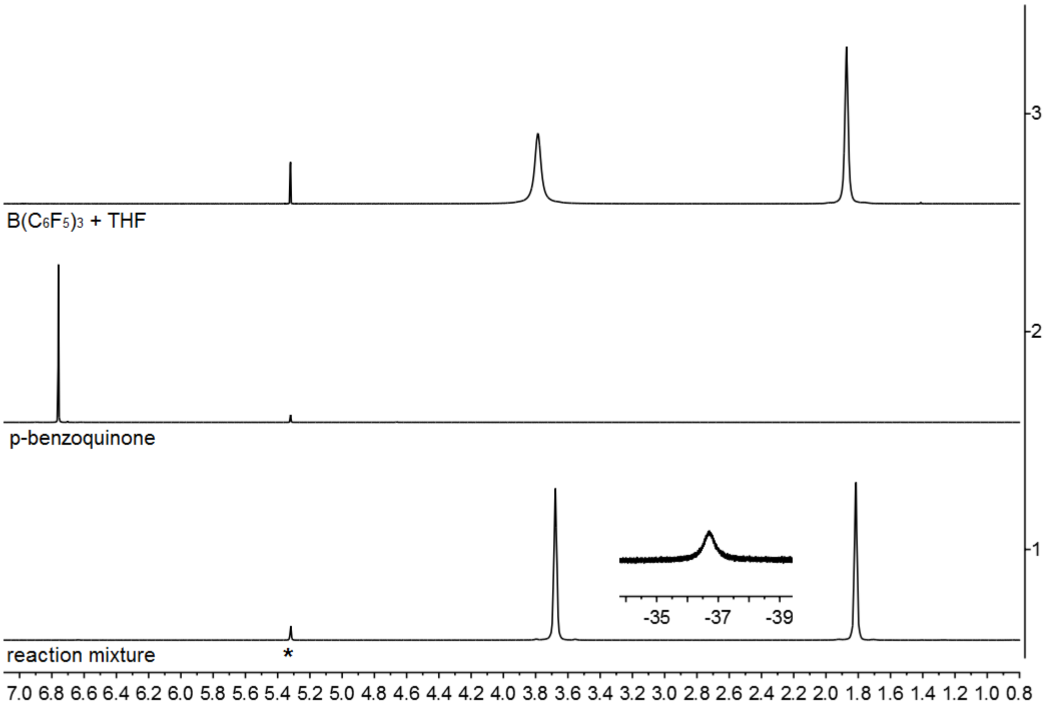

Figure S89. ^1H NMR (600 MHz, 299 K, CD_2Cl_2) spectra of (1) the reaction mixture of experiment 1, (2) p-benzoquinone, (3) the mixture of $B(C_6F_5)_3$ and one drop of THF for comparison.

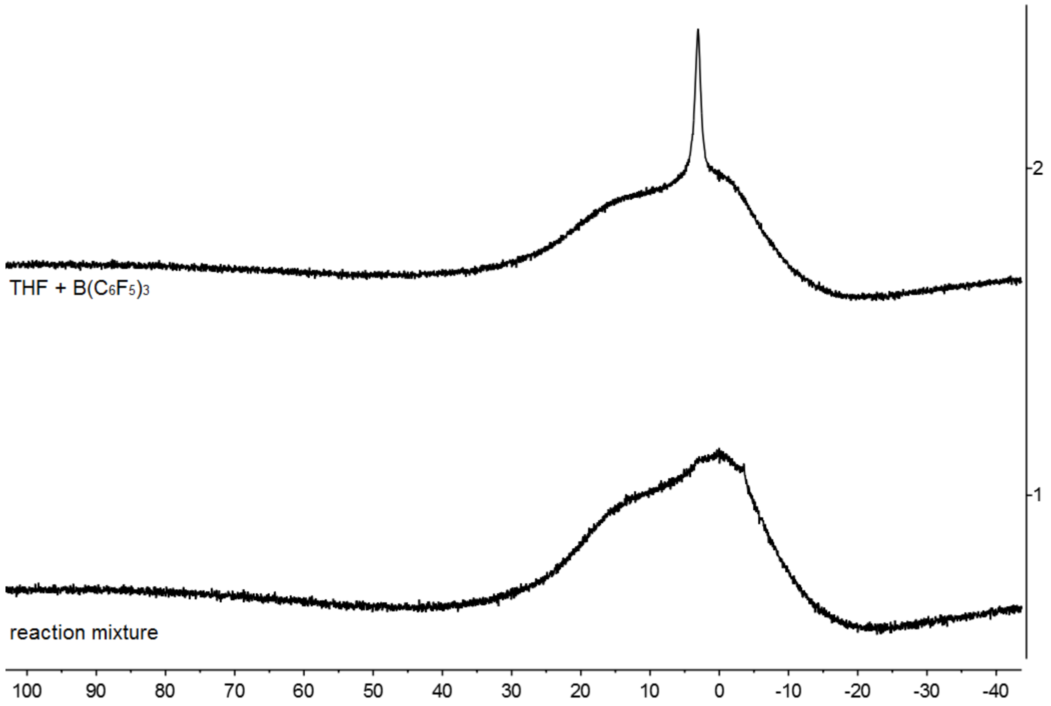

Figure S90. $^{11}B\{^{1}H\}$ NMR (192 MHz, 299 K, CD_2Cl_2) spectra of (1) the reaction mixture of experiment 1 and (2) the mixture of $B(C_6F_5)_3$ and one drop of THF for comparison .

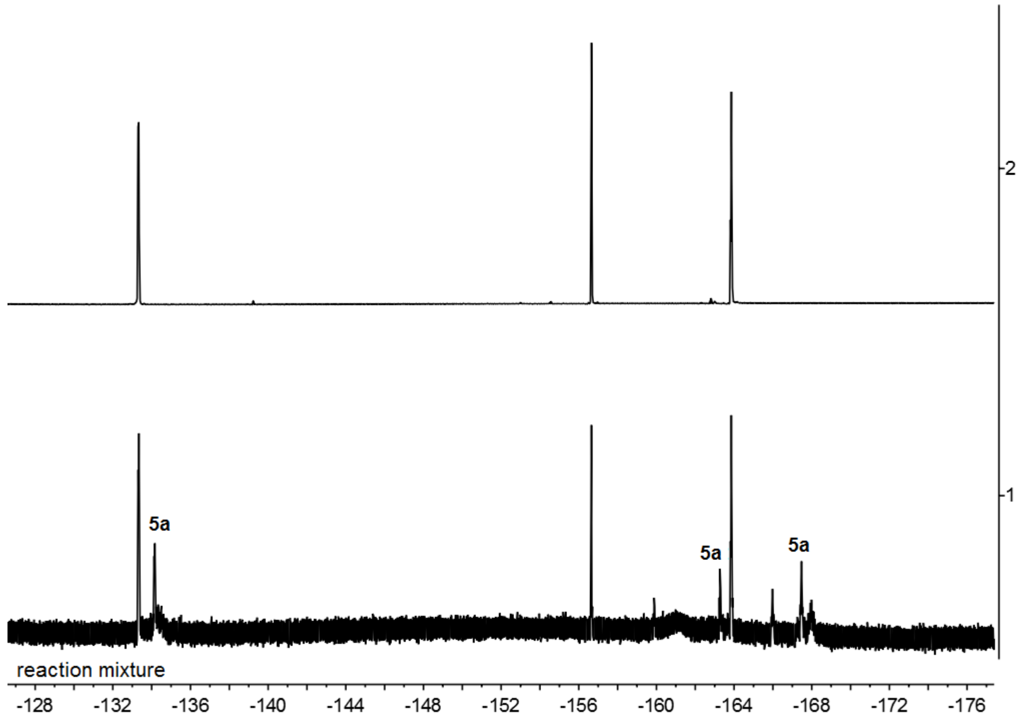

Figure S91. ^{19}F NMR (564 MHz, 299 K, CD_2Cl_2) spectra of (1) the reaction mixture of experiment 1 and (2) the mixture of $B(C_6F_5)_3$ and one drop of THF for comparison .

Experiment 2: (NMR scale, reaction of compound **8c** with DMSO in CD_2Cl_2)

Scheme S35

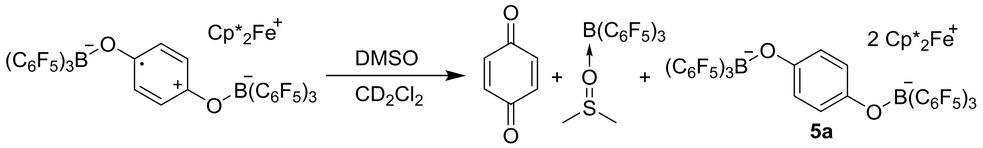

One drop of DMSO was added to a solution of compound **8c** (10 mg, 0.007 mmol) in CD_2Cl_2 (0.5 mL) and the resulting green solution was characterized by NMR after 24 h at room temperature. [Comment: The DMSO/$B(C_6F_5)_3$ adduct, p-benzoquinone and compound **5a** were observed by NMR experiments. The NMR data of dianion part of compound **5a** in the reaction mixture are comparable with those cited in the literature [L. Liu, L. L. Cao, Y. Shao, D. W. Stephan, *J. Am. Chem. Soc.* **2017**, *139*, 10062–10071].

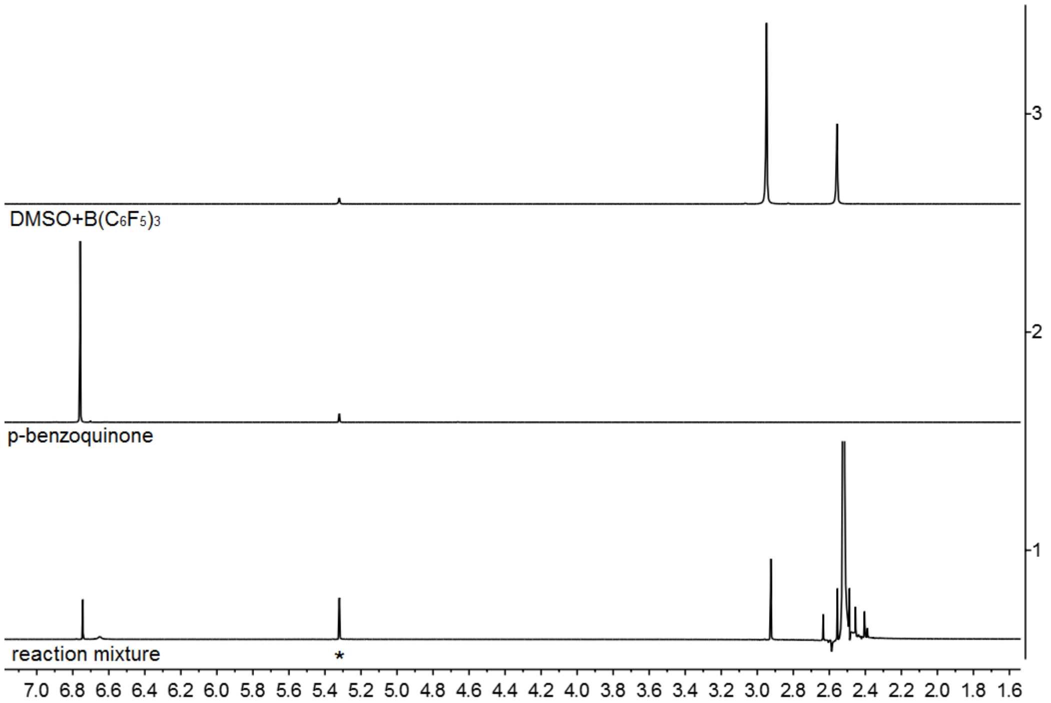

Figure S92. ^{1}H NMR (600 MHz, 299 K, CD_2Cl_2) spectra of (1) the reaction mixture of experiment 2, (2) p-benzoquinone and (3) the mixture of $B(C_6F_5)_3$ and one drop of DMSO for comparison.

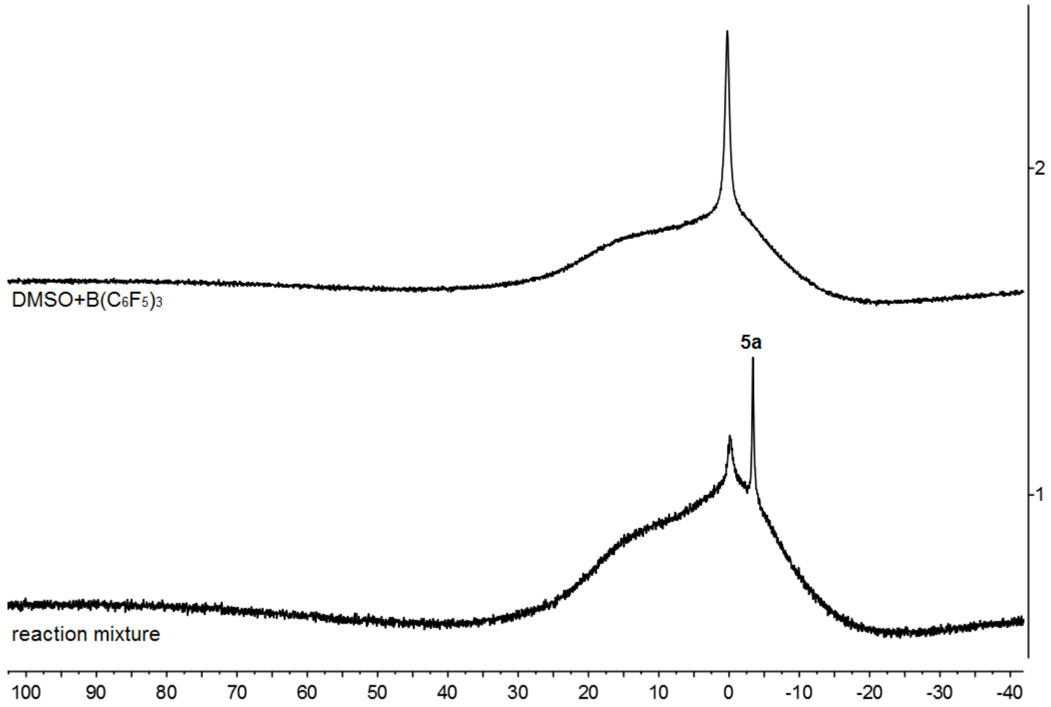

Figure S93. $^{11}B\{^{1}H\}$ NMR (192 MHz, 299 K, CD_2Cl_2) spectra of (1) the reaction mixture of experiment 2 and (2) the mixture of $B(C_6F_5)_3$ and one drop of DMSO for comparison .

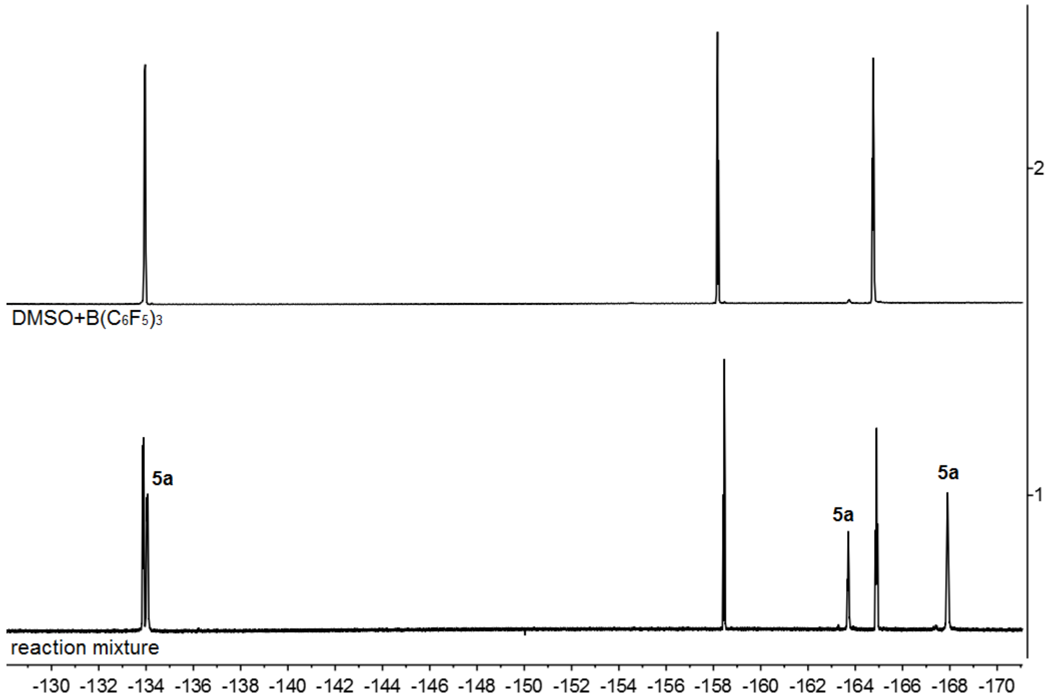

Figure S94. ^{19}F NMR (564 MHz, 299 K, CD_2Cl_2) spectra of (1) the reaction mixture of experiment 2 and (2) the mixture of $B(C_6F_5)_3$ and one drop of DMSO for comparison .

T) Reaction of compound **11c** with THF and DMSO, respectively

Experiment 1: (NMR scale, reaction of compound **11c** with THF in CD_2Cl_2)

Scheme S36

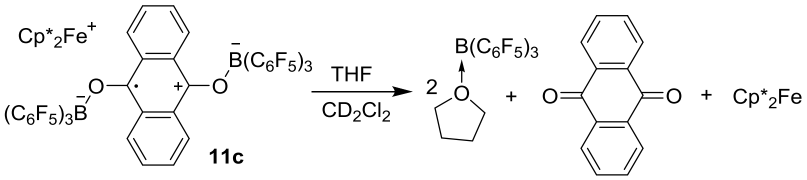

One drop of THF was added to a solution of Compound **11c** (10 mg, 0.007 mmol) in CD_2Cl_2 (0.5 mL) at room temperature. A pale yellow solution obtained in a few minutes, which was subsequently characterized by NMR experiments.

[Comment: The THF/$B(C_6F_5)_3$ adduct, 9,10-anthraquinone and decamethylferrocene were observed by NMR experiments]

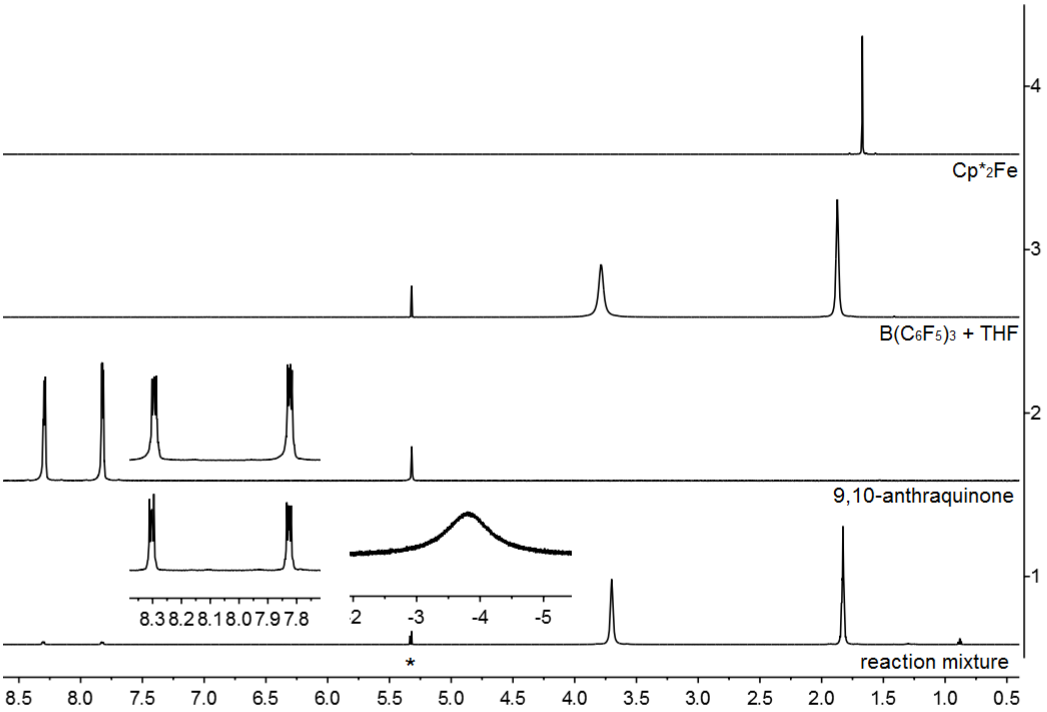

Figure S95. ^1H NMR (600 MHz, 299 K, CD_2Cl_2) spectra of (1) the reaction mixture, (2) 9,10-anthraquinone, (3) the mixture of $B(C_6F_5)_3$ and one drop of THF and (4) decamethylferrocene for comparison.

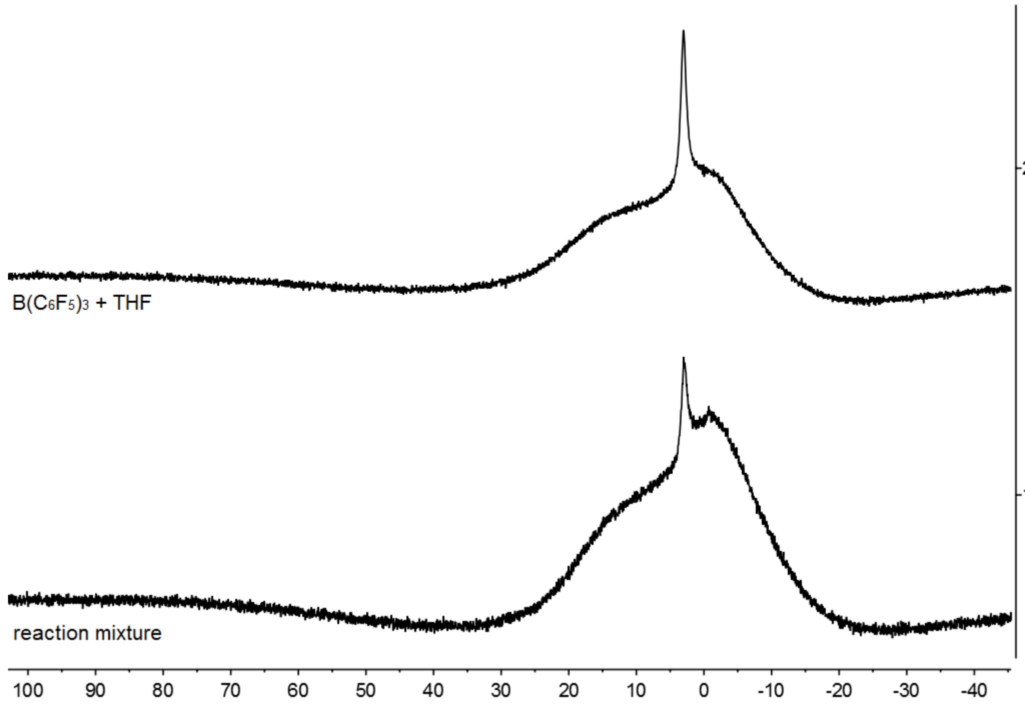

Figure S96. $^{11}B\{^{1}H\}$ NMR (192 MHz, 299 K, CD_2Cl_2) spectra of (1) the reaction mixture and (2) the mixture of $B(C_6F_5)_3$ and one drop of THF for comparison .

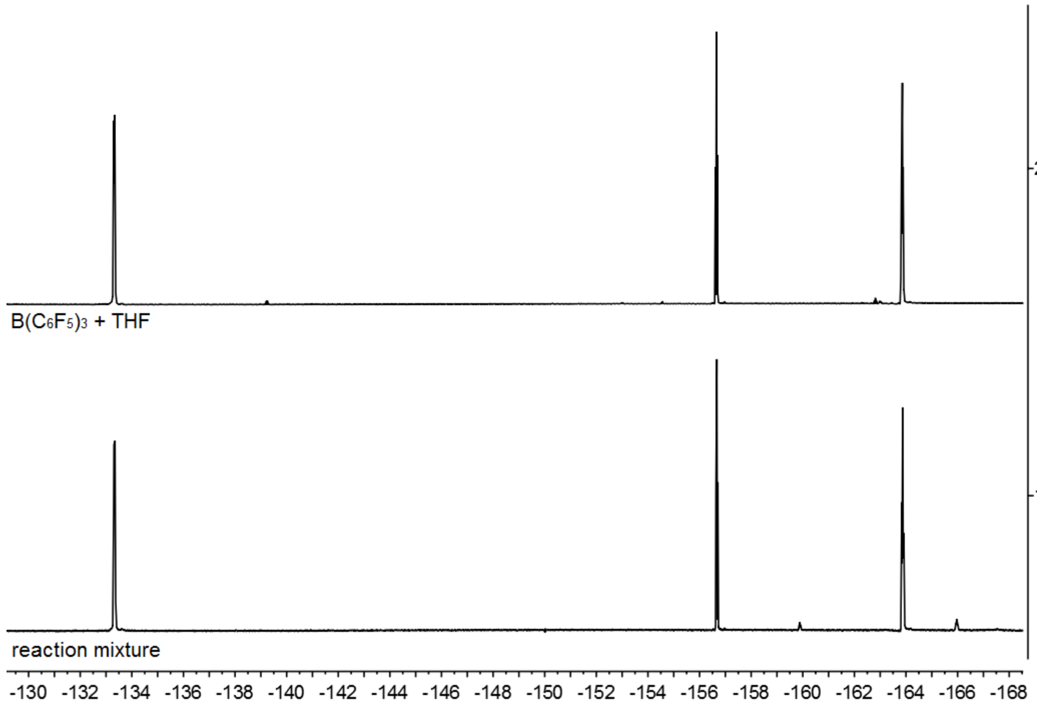

Figure S97. ^{19}F NMR (564 MHz, 299 K, CD_2Cl_2) spectra of (1) the reaction mixture and (2) the mixture of $B(C_6F_5)_3$ and one drop of THF for comparison .

Experiment 2: (NMR scale, reaction of compound **11c** with DMSO in CD_2Cl_2)

Scheme S37

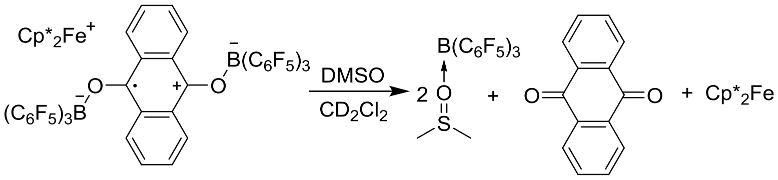

One drop of DMSO was added to a solution of compound **11c** (10 mg, 0.007 mmol) in CD_2Cl_2 (0.5 mL) at room temperature. A pale yellow solution was obtained in a few seconds, which was subsequently characterized by NMR experiments.

[Comment: The DMSO/$B(C_6F_5)_3$ adduct, 9,10-anthraquinone and decamethylferrocene (a broad signal) were observed by NMR experiments]

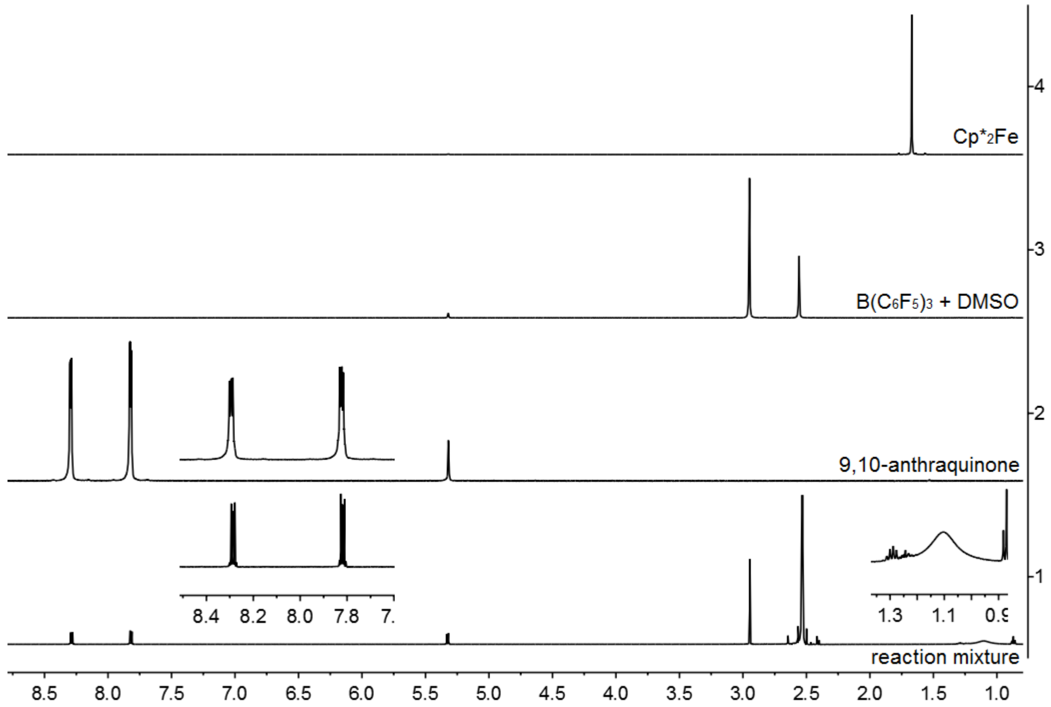

Figure S98. ^1H NMR (600 MHz, 299 K, CD_2Cl_2) spectra of (1) the reaction mixture, (2) 9,10-anthraquinone, (3) the mixture of $B(C_6F_5)_3$ and one drop of DMSO and (4) decamethylferrocene for comparison.

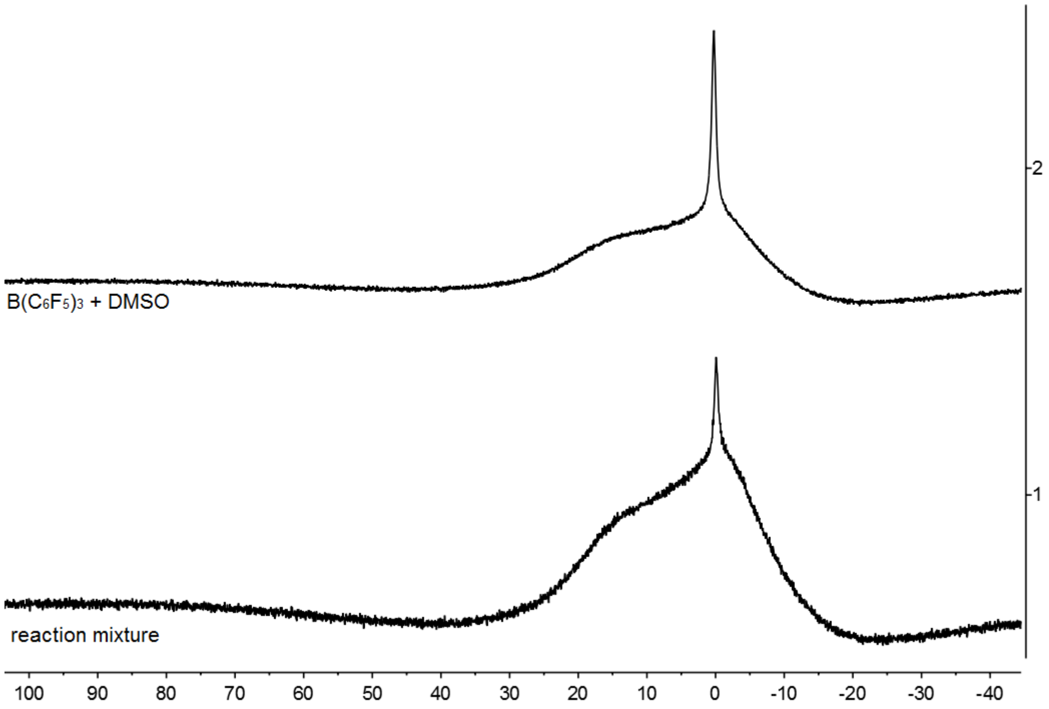

Figure S99. $^{11}B\{^{1}H\}$ NMR (192 MHz, 299 K, CD_2Cl_2) spectra of (1) the reaction mixture and (2) the mixture of $B(C_6F_5)_3$ and one drop of DMSO for comparison .

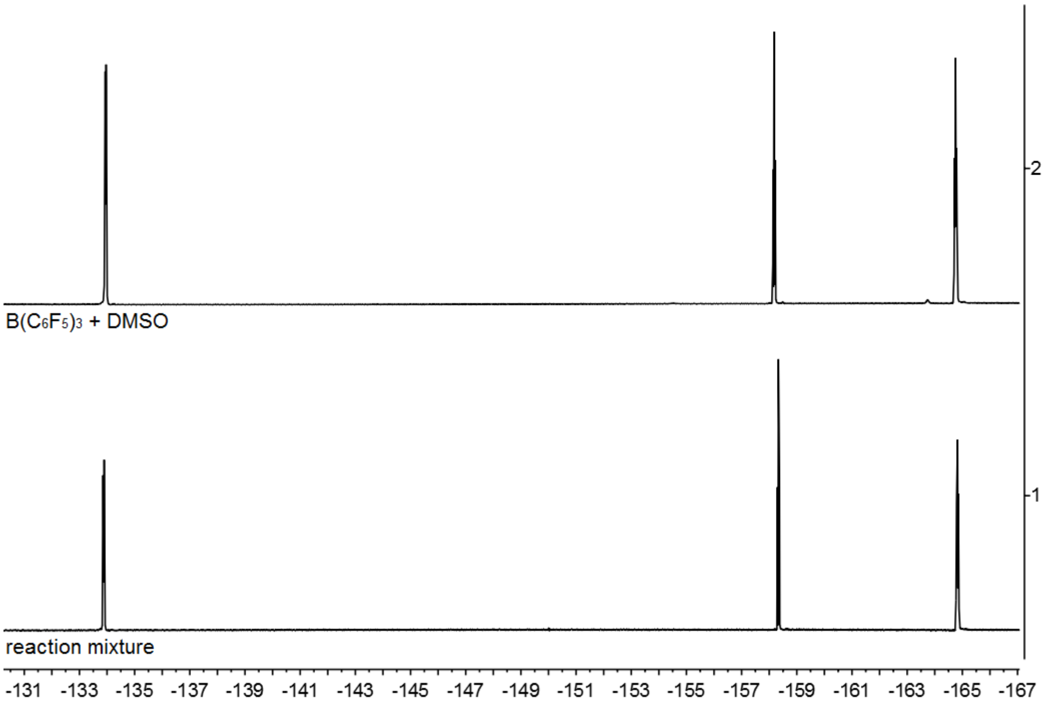

Figure S100. ^{19}F NMR (564 MHz, 299 K, CD_2Cl_2) spectra of (1) the reaction mixture and (2) the mixture of $B(C_6F_5)_3$ and one drop of DMSO for comparison .

U) Reaction of compound **12c** with THF and DMSO, respectively

Experiment 1: (NMR scale, reaction of compound **12c** with THF in CD_2Cl_2)

Scheme S38

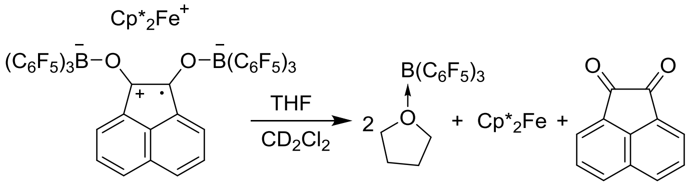

One drop of THF was added to a solution of compound **12c** (15.0 mg, 0.010 mmol) in CD_2Cl_2 (0.5 mL) and the resulting solution was characterized by NMR experiments after 72 h at room temperature. [Comment: The THF/$B(C_6F_5)_3$ adduct, decamethylferrocene and acenaphthenequinone were observed by NMR experiments]

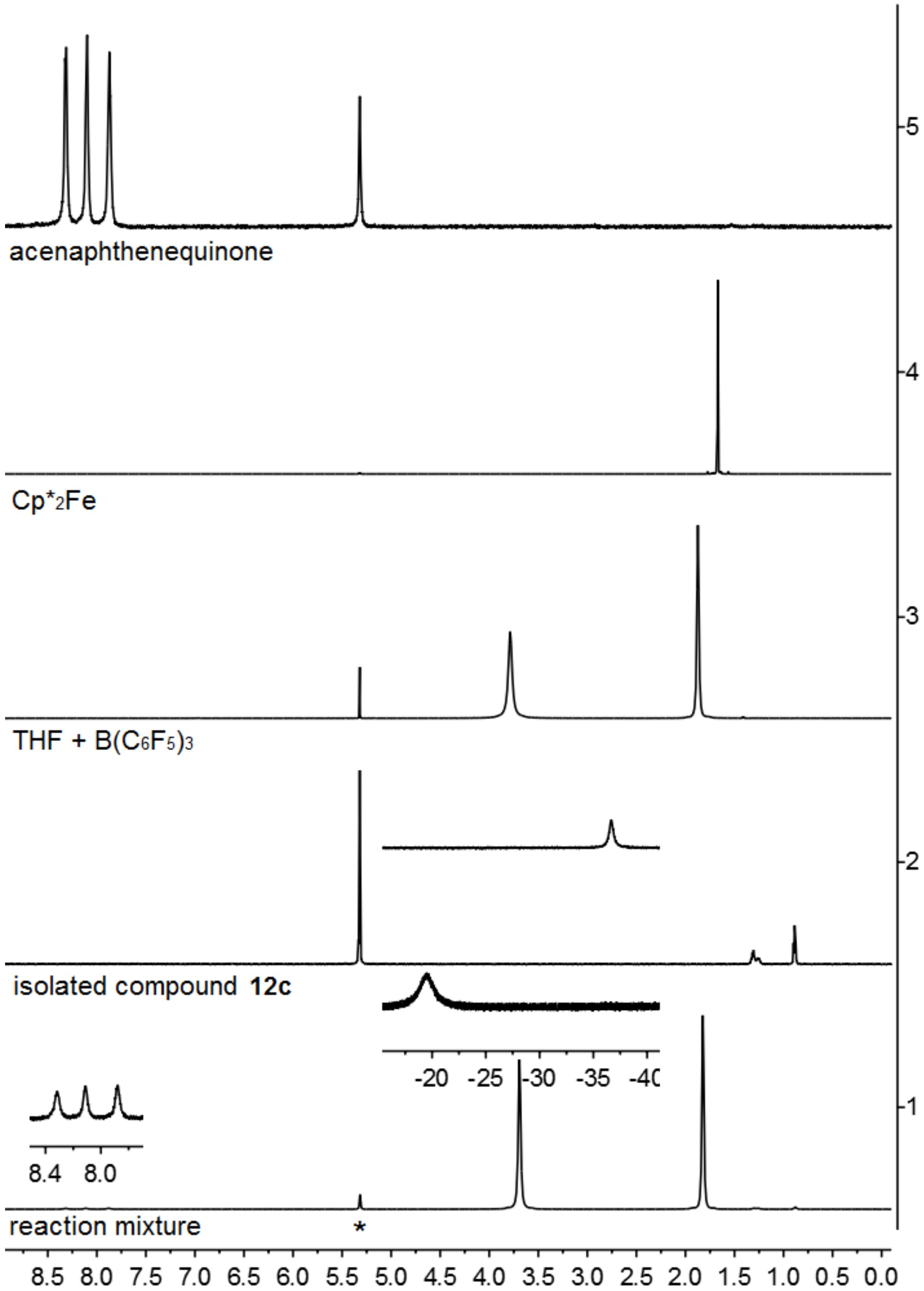

Figure S101. ^{1}H NMR (600 MHz, 299 K, CD_2Cl_2) spectra of (1) the reaction mixture, (2) acenaphthenequinone, (3) isolated compound **12c** and (4) the mixture of $B(C_6F_5)_3$ and one drop of THF for comparison.

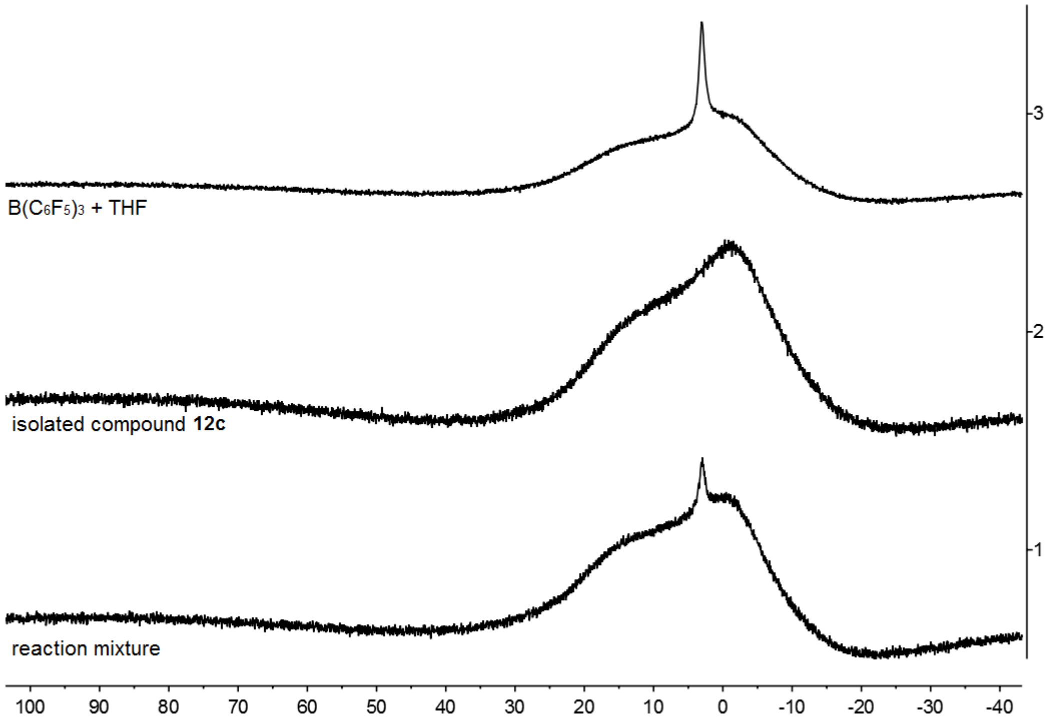

Figure S102. $^{11}B\{^1H\}$ NMR (192 MHz, 299 K, CD_2Cl_2) spectra of (1) the reaction mixture of experiment 1 and (2) the mixture of $B(C_6F_5)_3$ and one drop of THF for comparison .

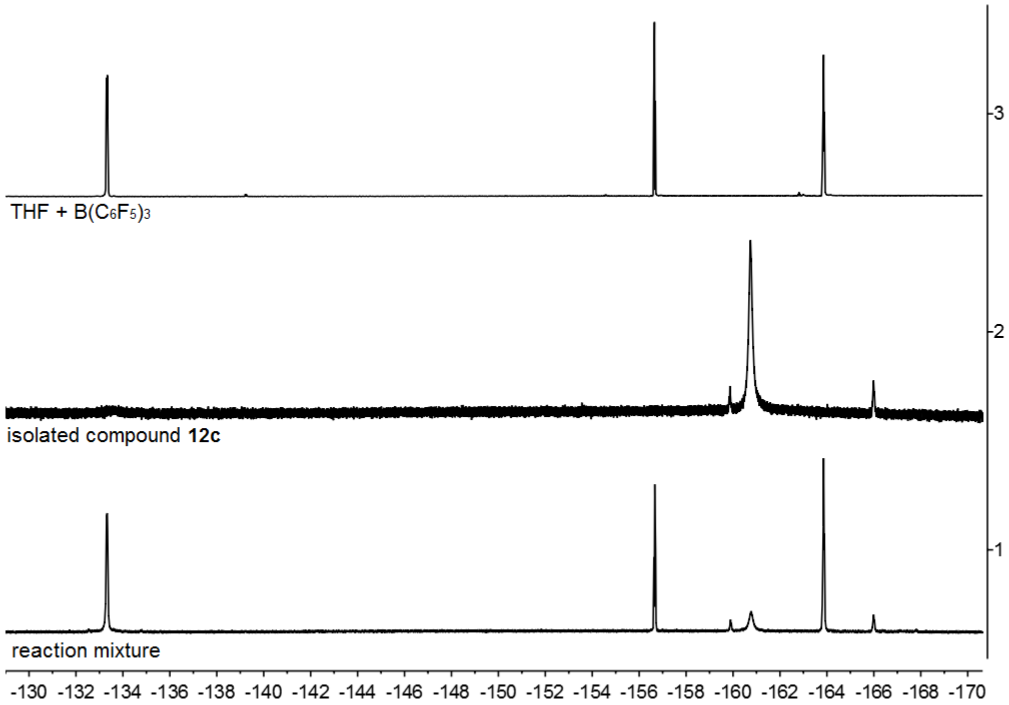

Figure S103. ^{19}F NMR (564 MHz, 299 K, CD_2Cl_2) spectra of (1) the reaction mixture of experiment 1 and (2) the mixture of $B(C_6F_5)_3$ and one drop of THF for comparison .

Experiment 2: (NMR scale, reaction of compound **12c** with DMSO in CD_2Cl_2)

Scheme S39

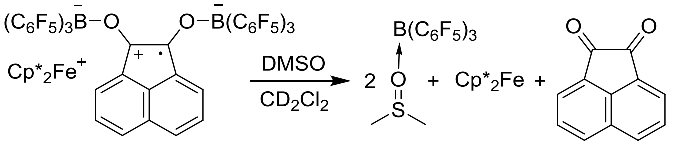

One drop of DMSO was added to a solution of compound **12c** (10 mg, 0.007 mmol) in CD_2Cl_2 (0.5 mL) and the resulting green solution was characterized by NMR after 1 h at room temperature. [Comment: The DMSO/$B(C_6F_5)_3$ adduct, decamethylferrocene and acenaphthenequinone were observed by NMR experiments]

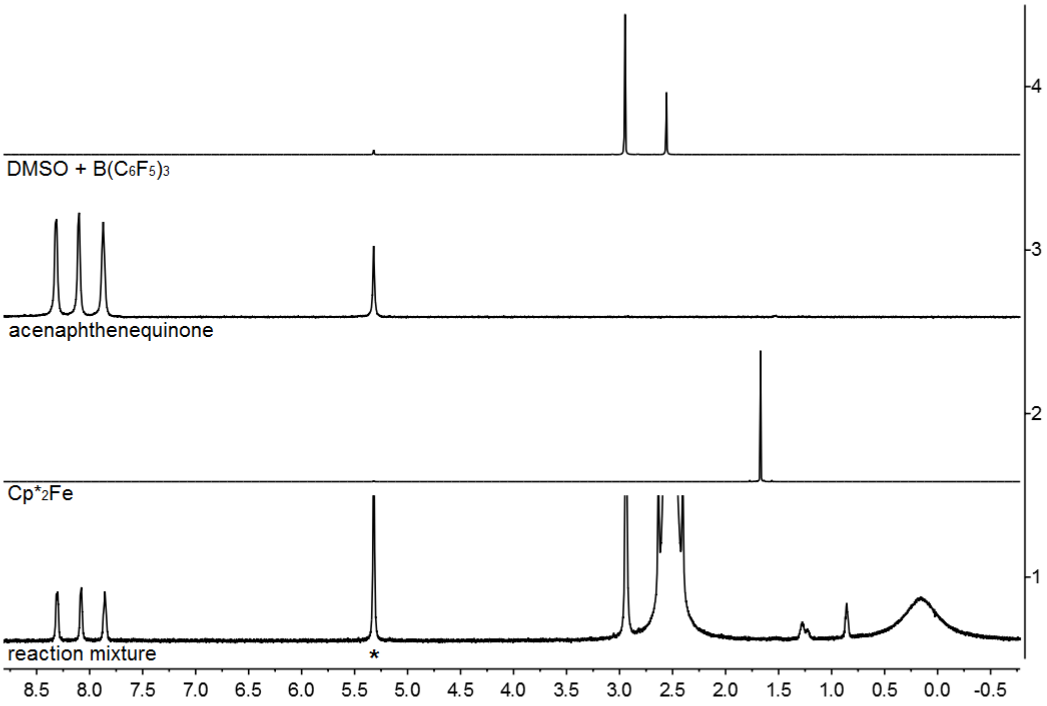

Figure S104. ^{1}H NMR (600 MHz, 299 K, CD_2Cl_2) spectra of (1) the reaction mixture of experiment 2, (2) acenphthenequinone, (3) decamethylferrocene and (4) the mixture of $B(C_6F_5)_3$ and one drop of DMSO for comparison.

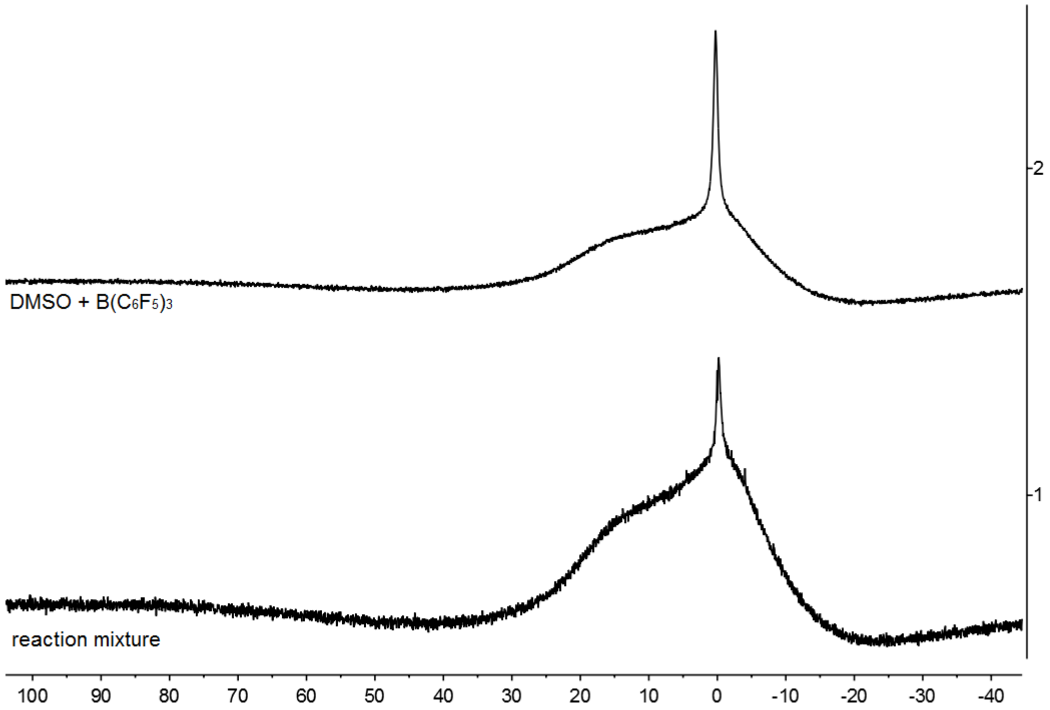

Figure S105. $^{11}B\{^{1}H\}$ NMR (192 MHz, 299 K, CD_2Cl_2) spectra of (1) the reaction mixture of experiment 2 and (2) the mixture of $B(C_6F_5)_3$ and one drop of DMSO for comparison .

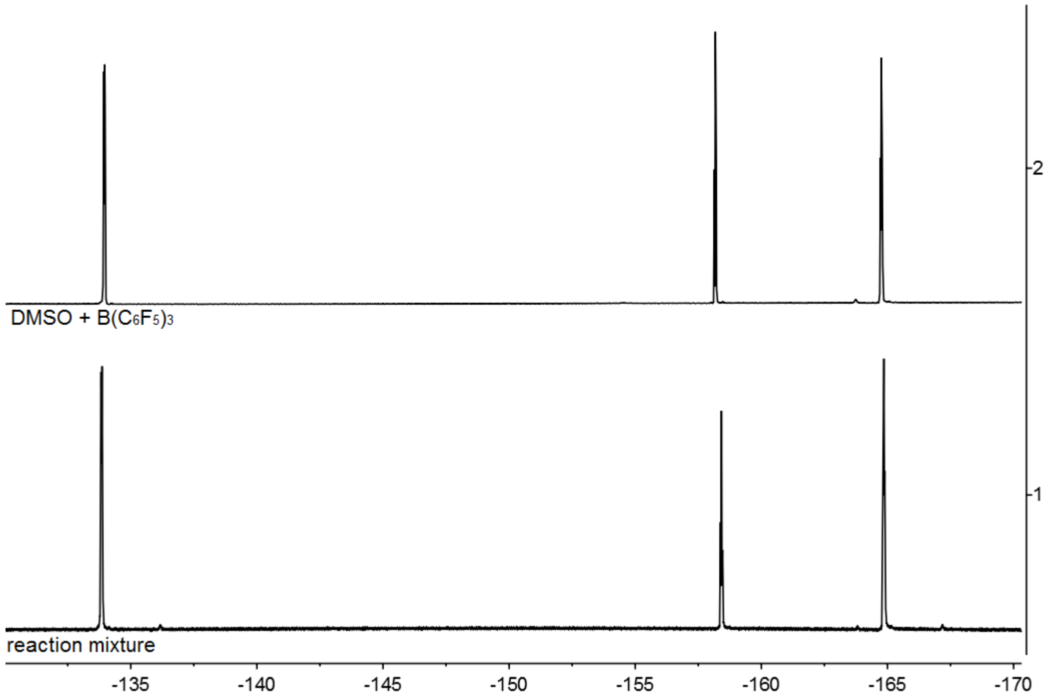

Figure S106. ^{19}F NMR (564 MHz, 299 K, CD_2Cl_2) spectra of (1) the reaction mixture of experiment 2 and (2) the mixture of $B(C_6F_5)_3$ and one drop of DMSO for comparison.

Cyclic voltammetry

Maximilian Lübbesmeyer, Armido Studer
Organisch-Chemisches Institut, Westfälische Wilhelm-Universität Münster, Corrensstraße 40, 48149 Münster, Germany

General: experiments were conducted in a *TSC 1600 closed* measuring cell that was connected to a *Metrohm Autolab PGSTAT204* potentiostat. The cell consists of the front-end of a platinum wire as the working electrode, a platinum crucible counter electrode and a silver wire pseudo-reference electrode. The examined substance (approx. 10 mM) was dissolved in a 0.1 M solution of tetrabutylammonium hexafluorophosphate in the specified solvent. The applied voltage was internally referenced to the redox potential of ferrocene or decamethylferrocene (-0.59 V vs. Fc/Fc^+ in dichloromethane[1], -0.505 V vs. Fc/Fc^+ in acetonitrile[2], -0.465 V vs. Fc/Fc^+ in dimethylsulfoxide[2], -0.48 V vs. Fc/Fc^+ in benzotrifluoride) and for reasons of comparability converted to potentials versus the ferrocene/ferrocenium redox couple. A scan rate of 100 mV/s was applied.

Comments

The diborylated semiquinone decamethylferrocenium salt **8c** is reversibly reduced to the O-borylated hydroquinone dianion at a redox potential of -0.03 V vs. Fc/Fc^+ in DCM and 0.04 V vs. Fc/Fc^+ in benzotrifluoride (Figures S107 and S108). The potentials were verified by examination of the decamethylferrocenium/hydroquinone dianion **5a** (Figure S110 and S111). Further electrochemical studies were complicated by the fast decomposition of the semiquinones under the experimental conditions. Despite several attempts no redox potentials for the semiquinones **11c**, **12c** and **13c** could be determined, neither in DCM nor in benzotrifluoride. Also, oxidation potentials of the dianions **5c** and **14c** could not be obtained. Measurements in solvents that can act as Lewis base donor ligands are not feasible due to fast decomposition of the semiquinones (see Scheme 3 in the manuscript). A solution of compound **8c** in DMSO and acetonitrile is, therefore, expected to contain *p*-benzoquinone, diborylated hydroquinone dianion **5a** and adducts of the respective solvent and $B(C_6F_5)_3$. Indeed, redox features corresponding to the reduction of the uncomplexed *p*-benzoquinone **3a** to the semiquinone were observed (see Figures S112 and S114) in these solvents. The measured redox potentials are in accordance with independent measurements of *p*-benzoquinone **3a** and decamethylferrocene in the respective solvent (Figure S113 and S115).

Reduction of the quinones in the presence of an excess of tris(pentafluorophenyl)borane did not lead to reproducible and sensible results. Hence, the reactivity of the borylated quinones was correlated to the redox potentials of the uncomplexed quinones. p-Benzoquinone is reduced to the semiquinone and dianion at potentials of -0.99 V and -1.8 V vs. Fc/Fc^+ in DCM (0.1 M Bu_4NPF_6, Figure S107), 9,10-anthraquinone **3b** is reduced at -1.49 V and -2.0 V vs. Fc/Fc^+, respectively (Figure S109). The reduction to the dianion could not be observed for acenaphthenequinone **3c** under the

described experimental conditions and is, therefore, expected to occur at <-2.1 V vs. Fc/Fc^+. The first reduction step takes place at -1.43 V vs. Fc/Fc^+ (Figure S117). Reduction of 9,10-phenanthrenequinone **3d** to the semiquinone and dianion occurs at -1.13 V and -1.8 V vs. Fc/Fc^+ in DCM (0.1 M Bu_4NPF_6, Figure S118).

The redox potentials of the reducing agents (decamethylferrocene: -0.59 V vs. Fc/Fc^+; trityl radical: -0.18 V vs. Fc/Fc^+; TEMPO: 0.24 V vs. Fc/Fc^+) were determined previously under the same experimental conditions.[3]

Tris(pentafluorophenyl)borane is irreversibly reduced at -1.6 V vs. Fc/Fc^+ in DCM (0.1 M Bu_4NPF_6, Figure 119).

The results show that precomplexation of the quinone with the borane Lewis acid is necessary in any of the examined reductions since neither the uncomplexed quinone nor the Lewis acid itself can be reduced by the employed reducing agents. Assuming similar trends in the redox potentials determined for the uncomplexed quinones with the complexed quinones some of the reactivity patterns observed in the course of the study can be explained. The most positive redox potential for the reduction of the quinone to the semiquinone was observed for *p*-benzoquinone. This is in accordance with the observation that only this quinone could be reduced by TEMPO in the presence of two equivalents of tris(pentafluorophenyl)borane. Diborylated acenaphthenesemiquinone **12c** could not further be reduced to the dianion by addition of decamethylferrocene. Accordingly, the reduction potential of the uncomplexed semiquinone appeared to be very low (<2.1 V vs. Fc/Fc^+).

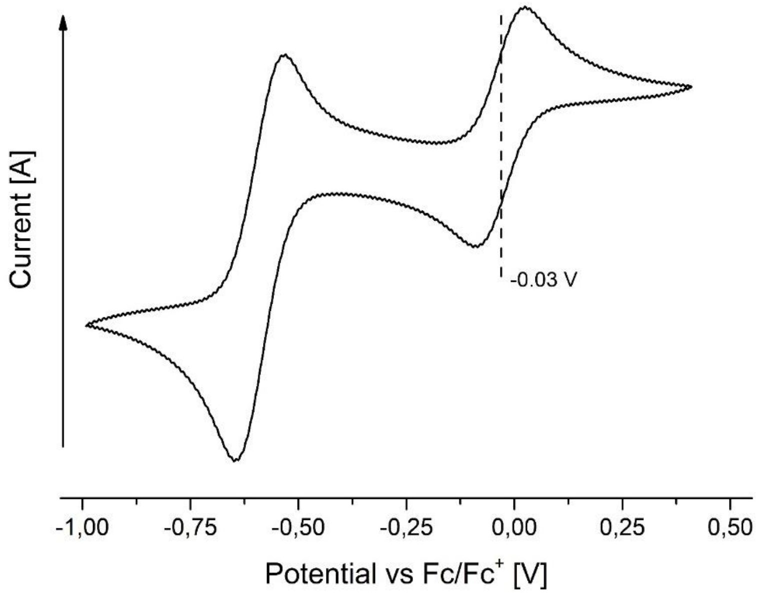

Figure S107. Cyclic voltammogram of decamethylferrocenium/semiquinone salt **8c** in DCM (0.1 M Bu_4NPF_6).

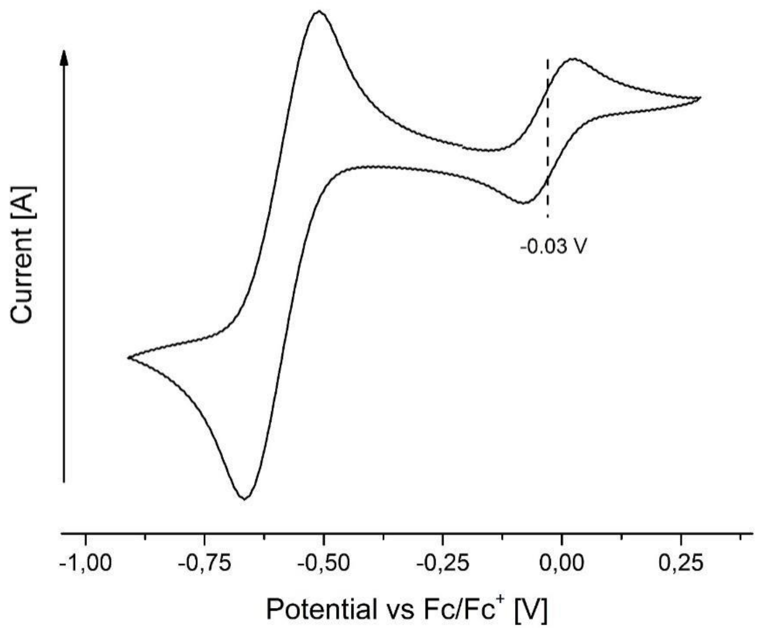

Figure S108. Cyclic voltammogram of decamethylferrocenium/hydroquinone dianion **5a** in DCM (0.1 M Bu_4NPF_6).

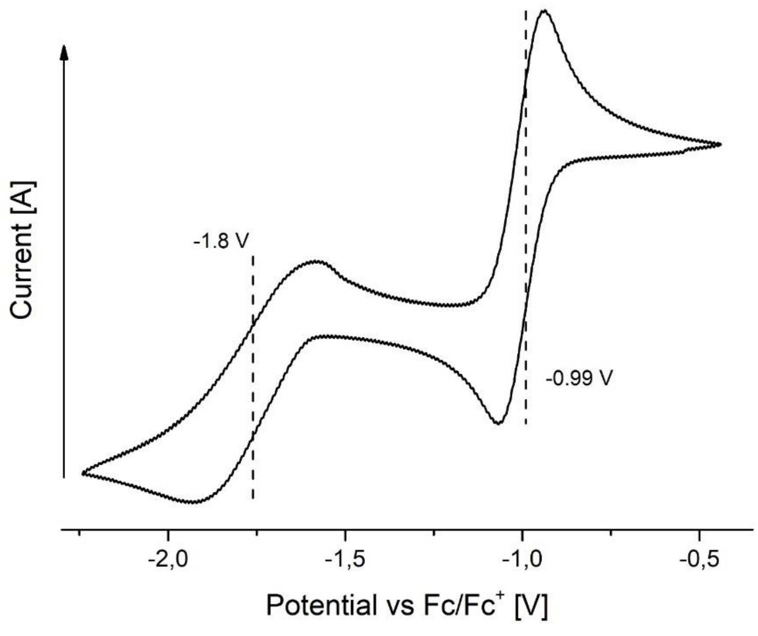

Figure S109. Cyclic voltammogram of *p*-benzoquinone **3a** in DCM (0.1 M Bu_4NPF_6).

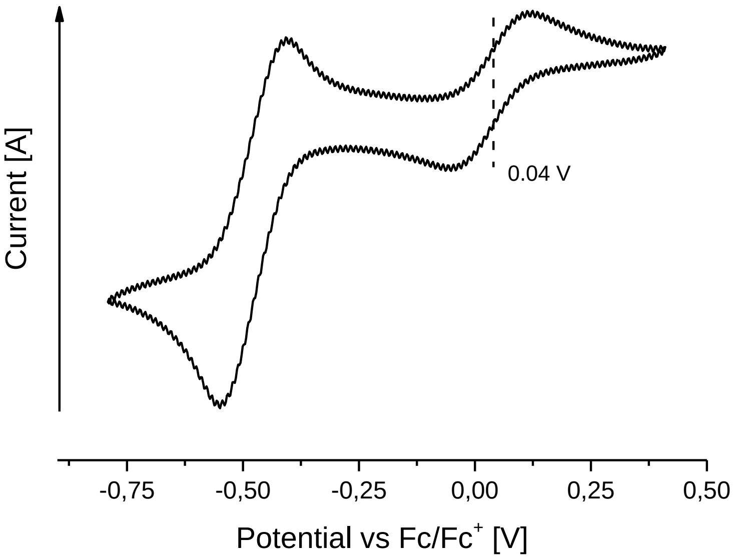

Figure S110. Cyclic voltammogram of decamethylferrocenium/semiquinone salt **8c** in benzotrifluoride (0.1 M Bu_4NPF_6).

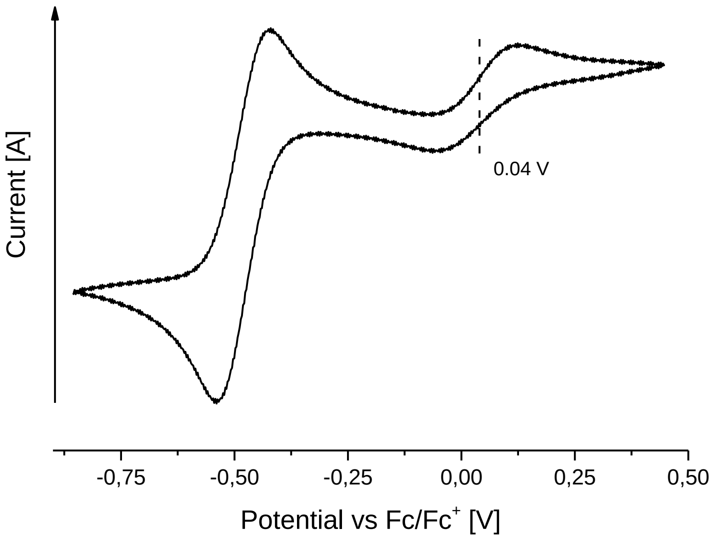

Figure S111. Cyclic voltammogram of decamethylferrocenium/hydroquinone dianion **5a** in benzotrifluoride (0.1 M Bu_4NPF_6).

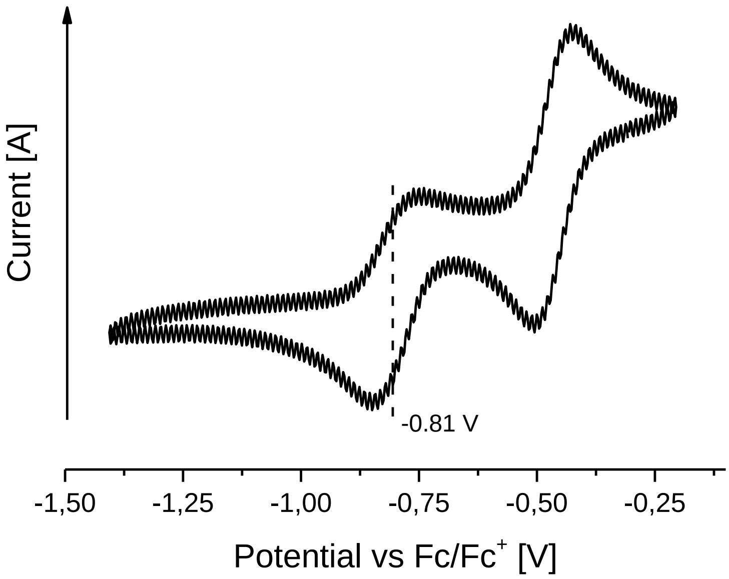

Figure S112. Cyclic voltammogram of decamethylferrocenium/semiquinone salt **8c** in DMSO (0.1 M Bu_4NPF_6). (Note that decomposition of the semiquinone according to the reaction presented in Scheme 3 has occurred.)

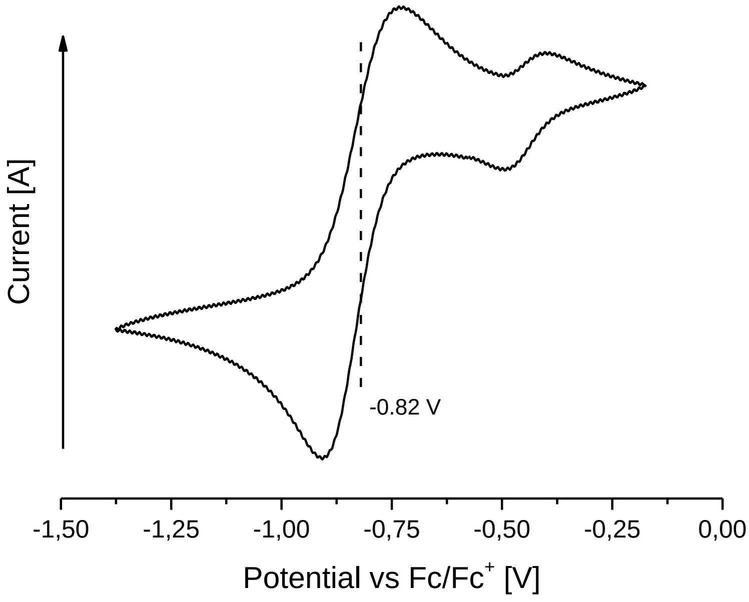

Figure S113. Cyclic voltammogram of p-benzoquinone **3a** and decamethylferrocene in DMSO (0.1 M Bu_4NPF_6).

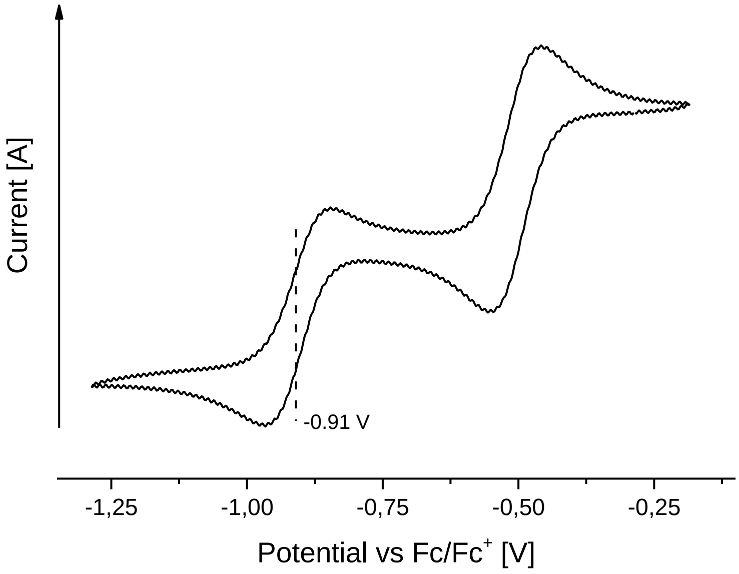

Figure S114. Cyclic voltammogram of decamethylferrocenium/semiquinone salt **8c** in acetonitrile (0.1 M Bu_4NPF_6). (Note that decomposition of the semiquinone likely according to the reaction presented in Scheme 3 has occurred.)

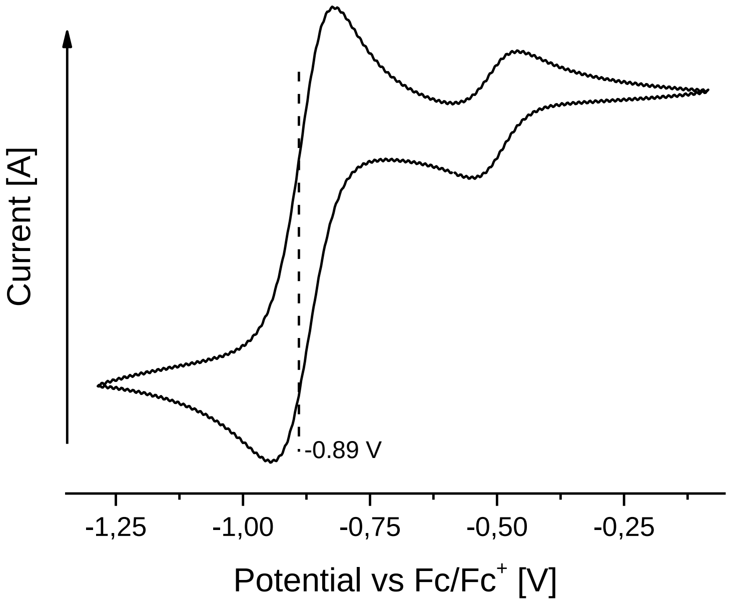

Figure S115. Cyclic voltammogram of p-benzoquinone **3a** and decamethylferrocene in acetonitrile (0.1 M Bu_4NPF_6).

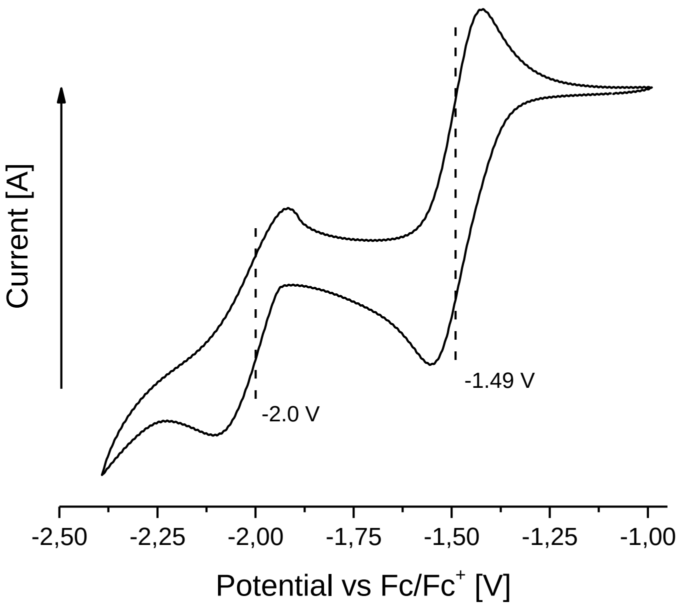

Figure S116. Cyclic voltammogram of 9,10-anthraquinone **3b** in DCM (0.1 M Bu_4NPF_6).

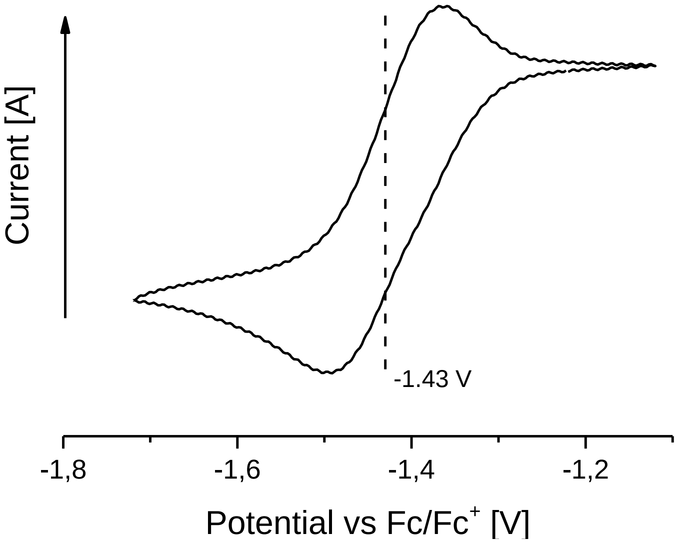

Figure S117. Cyclic voltammogram of acenaphthenequinone **3c** in DCM (0.1 M Bu_4NPF_6).

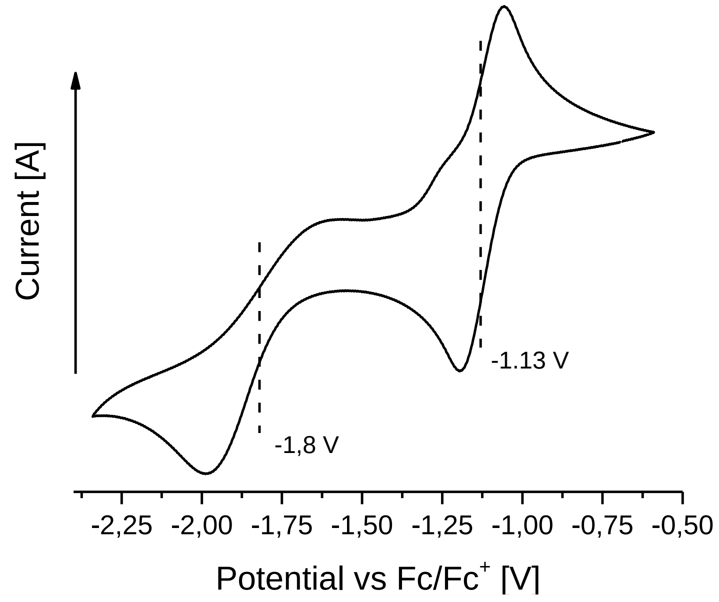

Figure S118. Cyclic voltammogram of 9,10-phenanthrenequinone **3d** in DCM (0.1 M Bu_4NPF_6).

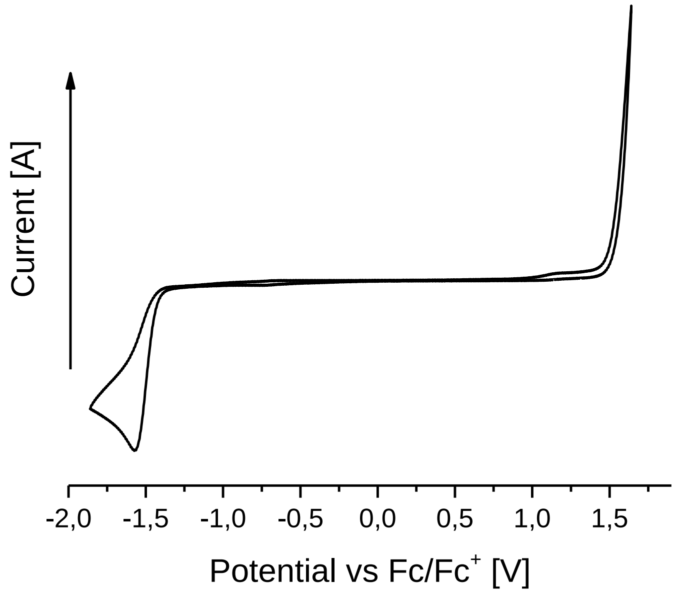

Figure S119. Cyclic voltammogram of tris(pentafluorophenyl)borane **1** in DCM (0.1 M Bu_4NPF_6).

References:

[1] N. G. Connelly, W. E. Geiger *Chem. Rev.*, **1996**, 96, 877–910.

[2] J. R. Aranzaes, M.-C. Daniel D. Astruc *Can. J. Chem.*, **2006**, 84, 288–299.

[3] X. Tao, C. G. Daniliuc, O. Janka, R. Pöttgen, R. Knitsch, M. R. Hansen, H. Eckert, M. Lübbesmeyer, A. Studer, G. Kehr, G. Erker *Angew. Chem.*, **2017**, 129, 16868–16871.

cw-EPR spectroscopy on tris(pentafluorophenyl)borane (BCF) stabilized semiquinones

Robert Knitsch,[b] Michael Ryan Hansen,[b] Hellmut Eckert,[b,c]
[b]Institut für Physikalische Chemie der Universität Münster, Correnssstraße 28, 48149 Münster, Germany, [c]Instituto de Fisica, Sáo Carlos, Universidade de Sáo Paulo, CP 369, 13560-970, Sáo Carlos, S.P. Brasil.

Experimental:

The *cw*-EPR experiments were carried out on carefully degassed solutions of the respective radical in DCM using a BRUKER EMXnano spectrometer operating at X-band frequencies with a microwave power of 0.32 mW. A modulation frequency for the magnetic field sweep of 100 kHz proved to be sufficient. The samples were sealed in glass capillaries in argon atmosphere and transferred to the spectrometer in liquid nitrogen. The measurement temperature was set to 180 K, slightly above the melting point of DCM resulting in improved resolution compared to elevated temperatures. For data acquisition the Software *XENON_nano* (version 1.3) was applied, while line shape simulations were carried out using the Easyspin package for Matlab[1]. The isotopic distribution of ^{10}B (I=3) and ^{11}B (I=3⁄2) was included in each simulation. For 2 equivalent boron atoms this leads to a natural distribution of 64% ^{11}B, ^{11}B; 32% ^{11}B, ^{10}B and 4% ^{10}B, ^{10}B. However hyperfine interaction couplings are only listed for ^{11}B, while the coupling to ^{10}B was set according to the gyromagnetic ratios (γ(^{11}B)/ γ(^{11}B) ~3).

Table S1: Experimental parameters used for *cw*-EPR spectroscopy for the spectra depicted in Figures S120 and S121**.**

Sample	concentration /(mol/L	center field/ G	sweep width/ G	conv. time/ms	microwave frequency/GHz	modulation amplitude/G	time constant/ ms	temp./K
**8a**	10^{-3}-10^{-4}	3432	45.3	110	9.634115	0.25	1.28	180
**8b**	10^{-3}-10^{-4}	3432	45.3	55.2	9.638988	0.25	1.28	180
**8c**	10^{-3}-10^{-4}	3432	45.3	55.2	9.630232	0.25	1.28	180
**8c** deut.	10^{-3}-10^{-4}	3432	45.3	55.2	9.640627	0.25	1.28	180
**11c**	10^{-3}-10^{-4}	3431	30.0	55.2	9.626514	0.25	1.28	180
**12c**	10^{-3}-10^{-4}	3431	30.0	55.2	9.630023	0.25	1.28	180
**13c**	10^{-3}-10^{-4}	3432	45.3	55.2	9.638831	0.25	1.28	180

DFT experimental

For DFT calculation of EPR parameters in a first step gas phase geometry optimization of the radical's molecular structure obtained via x-ray crystallography was performed with GAUSSIAN (version GAUSSIAN09)[2] using B3LYP[3, 4] as DFT-functional and the EPR-II basis set. Afterwards hyperfine interaction constants were calculated using the same set of parameters.

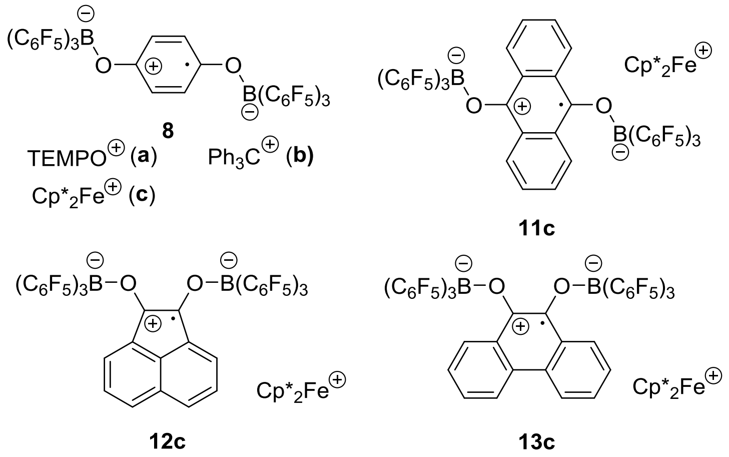

Scheme S40: Schematic representations of investigated radicals.

Table S2: Parameters used for line shape simulation of the *cw*-EPR spectra in Figure S120 and S121 and DFT-EPR parameters (in parentheses) calculated based on x-ray structures. Values in red had no influence on the quality of the fitted simulations and thus were set to the calculated parameters. See Figure S122 for the molecular geometries that form the basis for the DFT-calculated values and for the assignment of the coupling constants to the individual hydrogen species.

sample	g-factor	A (^{11}B)/ MHz	A_1 (^{1}H)/ MHz	A_2 (^{1}H)/ MHz	A_3 (^{1}H)/ MHz	A_4 (^{1}H)/ MHz	Gaussian LW/mT	Lorentzian LW/mT
**8a**	2.00494	6.3 (4.2)	8.1 (7.1)	3.4 (4.7)	---	---	0.09	0.05
**8b**	2.00490	6.4 (4.2)	8.2 (7.1)	3.4 (4.7)	---	---	0.01	0.12
**8c**	2.00486	6.3 (4.2)	8.2 (7.1)	3.5 (4.7)	---	---	0.07	0.07
**8c** deut.	2.00496	6.2 (4.2)	---	---	---	---	0.29	0.00
**11c**	2.00434	0.4 (0.4)	3.7 (3.7)	3.3(3.3)	2.9(2.9)	2.3(2.3)	0.37	0.03
**11c** no coup.	2.00434	---	---	---	---	---	0.50	0.02
**12c**	2.00459	3.2(3.2)	4.3(4.3)	3.3(3.3)	1.5(1.5)	---	0.15	0.05
**12c** no coup.	2.00459	---	---	---	---	---	0.51	0.02
**13c**	2.00468	1.3 (2.0)	7.9 (6.0)	5.4(1.7)	7.0(5.8)	1.3(1.3)	0.05	0.05

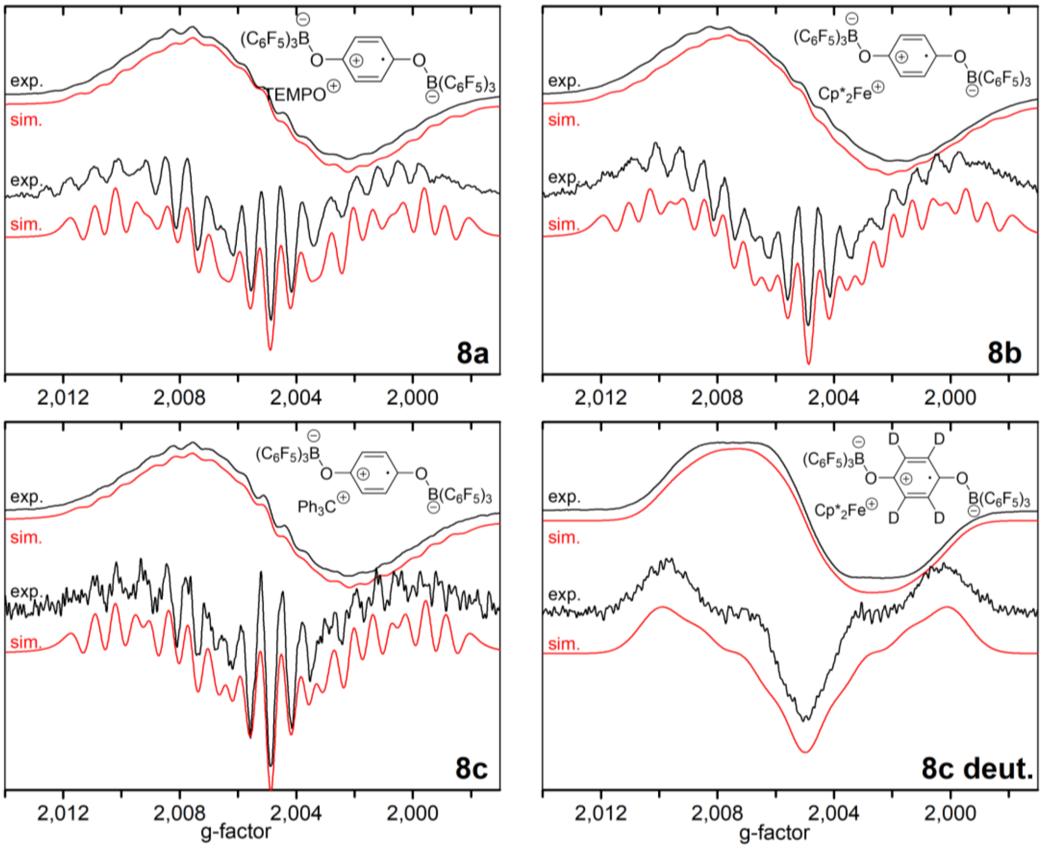

Figure S120: Experimental EPR spectra (first derivatives) and respective line-shape simulation (top) and second derivatives of the spectra (bottom) for structures **8a**, **8b**, **8c** and deuterated **8c** (optimized EPR parameters see Table S2; experimental parameters see Table S1).

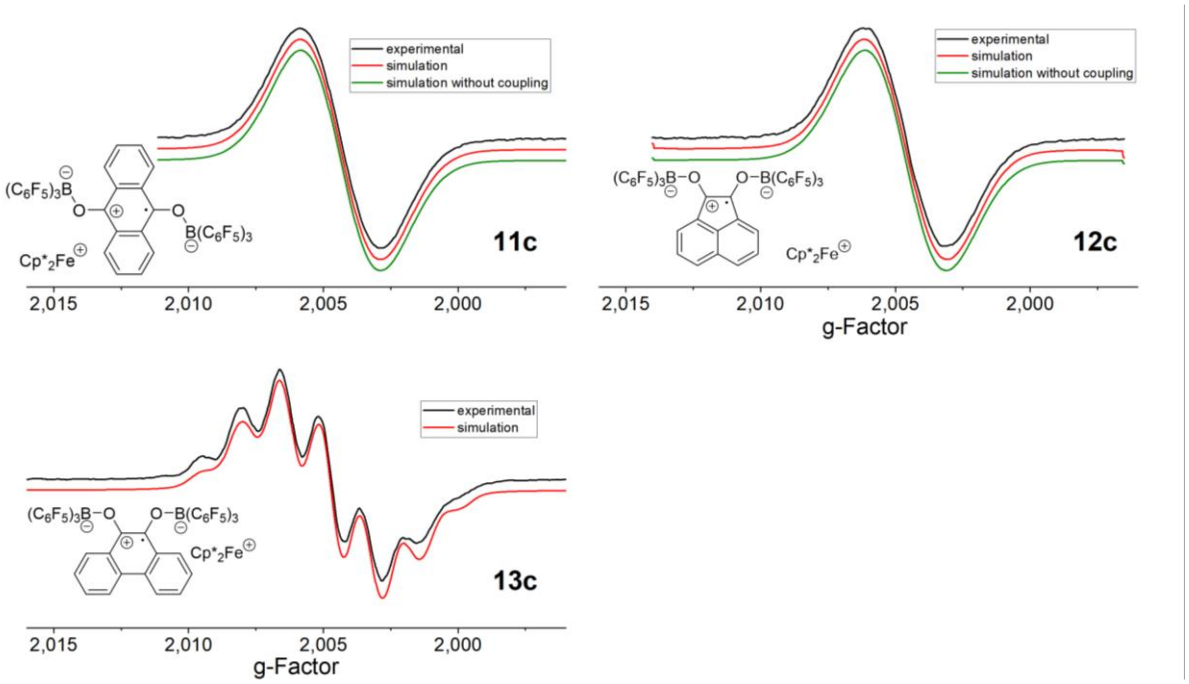

Figure S121: Experimental EPR spectra and respective line-shape simulation for structures **11c**, **12c** and **13c** (optimized EPR parameters see Table S2; experimental parameters see Table S1).

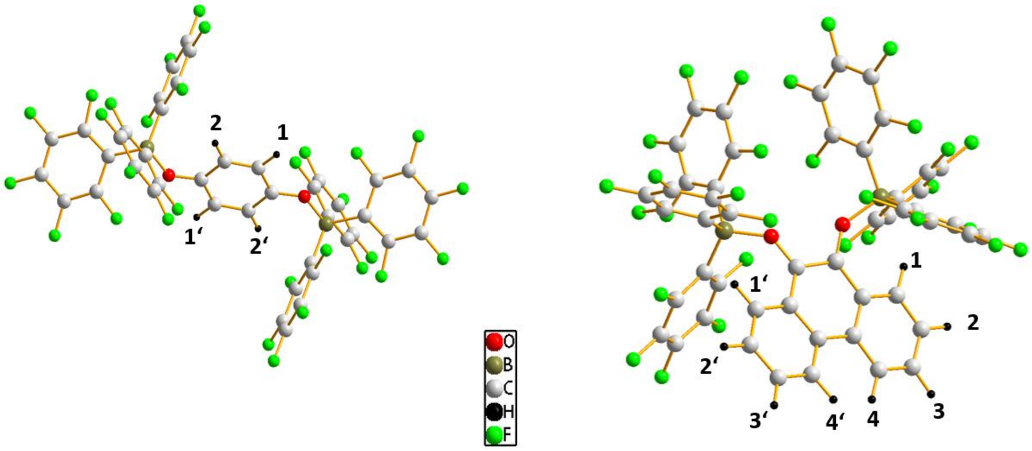

8a-8c

A_1=7.1 A_2=4.7

13c

A_1=6.0 A_2=1.7

A_3=5.8 A_4=1.3

Figure S122: Illustration of geometry optimized structures for compound **8a-8c** and **13c** and assignment of calculated ^{1}H hyperfine couplings (values given in MHz).

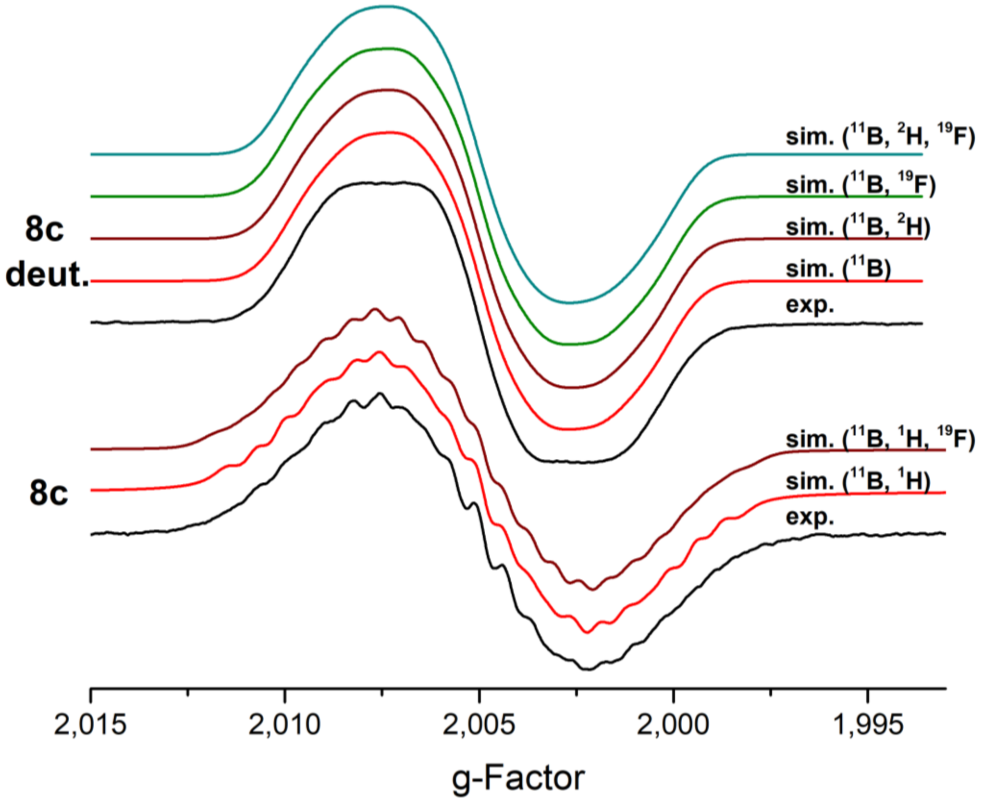

Figure S123: Experimental EPR spectra of **8c** and deuterated **8c** (black) and their respective line-shape simulation considering different interacting nuclei (for parameters see Table S3).

Table S3: Parameters used for line shape simulation of the cw-EPR spectra of **8c** depicted in Figure S123 taking different interacting nuclei (in parentheses) into account.

**Sample** (add. computed nuclei)	**A (^{11}B)/ MHz**	**A_1 (^{1}H/^{2}H)/ MHz**	**A_2 (^{1}H/^{2}H) / MHz**	**A_3 (^{19}F)/ MHz**	**A_4 (^{19}F)/ MHz**	**A_5 (^{19}F)/ MHz**	**Gaussian LW/mT**	**Lorentzian LW/mT**
**8c**	6.3	8.2/ 0	3.5/ 0	---	---	---	0.07	0.07
**8c** (^{19}F)	6.3	8.5/ 0	3.6/ 0	1.1	1.0	0.7	0.03	0.03
**8c** deut.	6.3	---	---	---	---	---	0.29	0.00
**8c** deut. (^{2}H)	6.3	0/ 1.3	0/ 0.6	---	---	---	0.25	0.01
**8c** deut. (^{19}F)	6.3	---	---	1.1	1.0	0.8	0.25	0.00
**8c** deut. (^{2}H, ^{19}F)	6.3	0/ 1.3	0/ 0.7	1.1	0.9	0.9	0.20	0.03

Results and Discussion:

All of the here investigated radicals gave EPR spectra with intrinsically broad EPR line-shapes, which could be decreased by neither decreased radical concentrations (10^{-3} to 10^{-4} M), enhanced O_2 removal procedures (freeze-pump-thaw method) nor by adjustment of measurement conditions (modulation amplitude (0.25 G), microwave power). We attribute this behaviour to the aggregation of several negatively charged radical anions together with their counter cations within the relatively unipolar solvent DCM. Furthermore, lowering the measurement temperature improves the resolution of the line-shapes observed. In some cases, partially resolved lines were observed at measurement temperatures near 180 K, close to the melting point of DCM. Attempts to break up the aggregates using solvents with a higher dielectric constant (such as 1,2-difluorobenzene or an ionic liquid (1-Butyl-1-methylpyrrolidinium tris(pentafluoroethyl)-trifluorophosphate) were unsuccessful and did not lead to improved resolution. Other more common solvents as DMSO or THF had to be excluded as they induce decomposition of the radicals by Lewis-pair formation with BCF (Tris(pentafluorophenyl)borane) leading to back electron transfer to the cation. This is demonstrated in Figure S124 showing the EPR spectrum of TEMPO after addition of THF and DMSO to **8a**. However, no line-shape improvement was achieved with any tested solvent. The measurement temperature, which had to be set higher for polar solvents due to their elevated melting points, appears to be more crucial for an EPR experiment on our radicals than the solvent.

The compounds **8a-8c** contain the same radical component and differ only in the choice of the

cation. Thus, we acquired very similar EPR spectra for all three compounds. To obtain EPR parameters we performed simulations for first and second derivative of the EPR spectra, minimizing deviations for both spectra shown in Figure S120, while Table S2 summarizes the interaction parameters. To decrease the number of fitting parameters we also investigated a fully deuterated sample of **8c**. As the coupling to ^{2}H nuclei can be neglected, the line-shape of the deuterated compound mainly reflects the electron-boron hyperfine interaction determined (6.4 MHz). Table S2 shows, that neglecting the coupling to ^{2}H leads to an increased Gaussian line width parameter. Simulations for EPR spectra acquired on **8a-8c** taking into consideration the natural isotope distribution starting from the ^{11}B hyperfine coupling of 6.4 MHz lead to similar coupling constants in the final simulation (6.2-6.4 MHz) as those obtained by simulating only the $^{11}B_2$ isotopologue. In isotropic solution, the four hydrogen ring species are expected to be equivalent if the conformational equilibration occurs rapidly on the EPR timescale. In contrast, for achieving a satisfactory simulation of the 180 K spectra two different coupling constants had to be assumed, reflecting two pairs of equivalent ^{1}H atoms, indicating approach to the slow-exchange limit. This finding could be a consequence of restricted conformational mobility of the oxygen-BCF units owing to aggregate formation. As illustrated in the literature, slow-exchange phenomena involving semiquinone radicals are not uncommon in low-temperature EPR spectra of radical ion solutions.[5]
As a starting point for spectrum fitting, we used the calculated EPR parameters for the gas phase optimized structure depicted in Figure S122 (left), implicitly assuming that this conformation is the dominant one in solution. Hyperfine coupling to the ^{19}F nuclei of the C_6F_5 residues had no influence on the simulations and were thus included in an overall linebroadening parameter. Justification for this approach comes from Figure S123, which shows the experimental spectrum of **8c** and deuterated **8c** and their simulations with and without hyperfine coupling to fluorine and deuterium. In these simulations the starting values for the ^{2}H coupling constants were set to 1⁄6.51 times the experimental ^{1}H hyperfine coupling constants of **8c** while starting values for the ^{19}F hyperfine coupling constants were set to those measured with exceptional resolution for a very similar uncharged molecule.[6] These simulations were based on the three strongest couplings (starting at 1.06, 0.92 and 0.78 MHz) present for sets of 6 equivalent fluorine-atoms each of the $B(C_6F_5)_3$ substituent groups. In comparison with our calculations based on Gaussian09 our experimentally determined parameters are in the same order of magnitude as calculated hyperfine coupling constants. We attribute the greater deviations compared to Gaussian09 calculations of uncharged molecules to the more challenging calculation of a charged radical. Moreover, the structure of the gas-phase optimized molecular structure of the radical ion might differ from the average geometry in solution leading to the experimental spectra.
For the radicals of compound **11c** and **12c** even at low temperature we did not observe any hyperfine splitting (cf. Figure S121). In this material, the unpaired electron probability density is distributed over a larger aromatic system leading to decreased hyperfine coupling constants to both the boron and the hydrogen nuclei, as also predicted by our DFT calculations. We therefore were

only able to safely determine the g-factor of these two radicals (comp. Table S2). Test simulations based on theoretically computed hyperfine coupling constants showed, however, that these unresolved couplings may contribute to the line-width of the EPR spectrum.

Although compound **13c** also exhibits a large aromatic system as **11c** and **12c**, we were able to acquire a resolved EPR spectrum in this case, whose structure could be simulated on the basis of nuclear magnetic hyperfine interactions (cf. Figure S121). The simplest simulation might be envisioned by assuming coupling to two equivalent ^{11}B (I = 3/2 nuclei), however, as shown in Figure S125, such a simulation poorly reproduces the intensity distribution of the experimental seven-peak pattern. The best simulation is based on the parameters depicted in Table S2, with hyperfine coupling constants in reasonable agreement with DFT calculations. The results suggest that three pairs of protons show hyperfine coupling constants in the 5-8 MHz range, whereas the hyperfine coupling to the fourth set of protons is significantly weaker. Furthermore, the hyperfine coupling to ^{11}B and ^{10}B is significantly weaker than in compounds **8a** and **8c**, as also confirmed by the DFT calculation.

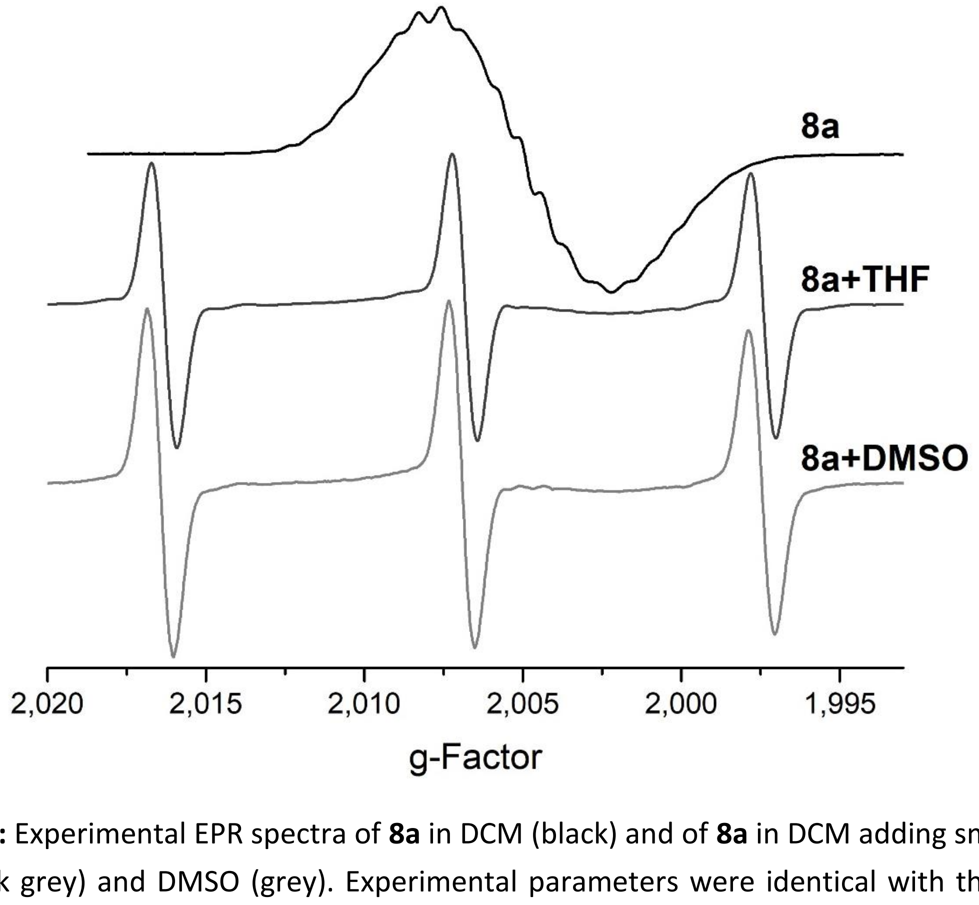

Figure S124: Experimental EPR spectra of **8a** in DCM (black) and of **8a** in DCM adding small amounts of THF (dark grey) and DMSO (grey). Experimental parameters were identical with those of **8a** in Table S1.

Table S4: Parameters used for line-shape simulation of the *cw*-EPR spectrum of **13c** depicted in Figure S125 performed for varying hyperfine coupling constants for the boron and hydrogen nuclei.

sample	A (^{11}B)/ MHz	A_1 (^{1}H)/ MHz	A_2 (^{1}H)/ MHz	A_3 (^{1}H)/ MHz	A_4 (^{1}H)/ MHz	Gaussian LW/mT	Lorentzian LW/mT
**13c sim. A**	1.3	7.9	5.4	7.0	1.3	0.05	0.05
**13c sim B1**	6.9	---	---	---	---	0.05	0.05
**13c sim B2**	6.9	2.1	1.05	1.7	0.14	0.05	0.05

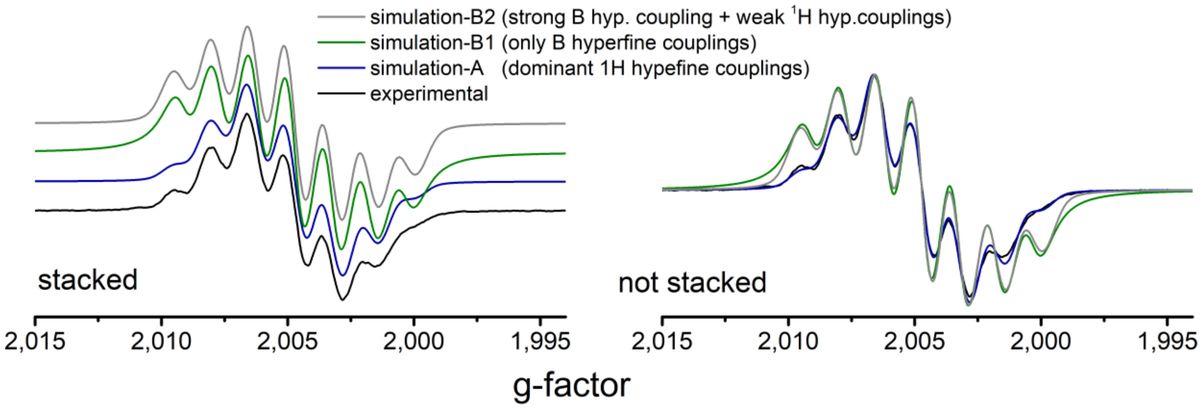

Figure S125: Experimental *cw*-EPR spectrum of **13c** and corresponding simulations assuming dominant ^{1}H hyperfine couplings (A, blue), coupling only to two boron nuclei (B1, green) and strong coupling to the boron atoms with weak optimized couplings to the set of ^{1}H nuclei (B2, grey) (for parameters see Table S4).

References:

[1] S. Stoll, A. Schweiger *J. Magn. Reson.,* **2006**, 178, 42-55
[2] M. J. Frisch *et al*. Gaussian 09; Gaussian, Inc., Wallingford, CT, 2009.
[3] Becke, A. D. *J. Chem. Phys.* **1993**, 98, 5648-565.
[4] P. J. Stephens, F. J. Devlin, C. F. Chabalowski, M. Frisch, *J. Phys. Chem.* **1994**, 98, 11623.
[5] J. M. Lü, S. V. Rosokha, I. S. Neretin, J. K. Kochi, *J. Am. Chem. Soc.* **2006**, 128, 16708-16719.
[6] S. S. Eaton *et al. J. Magn. Res.* **2017**, 276, 7-13.
